# Drugs to reduce bleeding and transfusion in major open vascular or endovascular surgery: a systematic review and network meta‐analysis

**DOI:** 10.1002/14651858.CD013649.pub2

**Published:** 2023-02-17

**Authors:** Anair Beverly, Giok Ong, Catherine Kimber, Josie Sandercock, Carolyn Dorée, Nicky J Welton, Peter Wicks, Lise J Estcourt

**Affiliations:** Systematic Review InitiativeNHS Blood and TransplantOxfordUK; Population Health Sciences, Bristol Medical SchoolUniversity of BristolBristolUK; Cardiac Anaesthesia and Intensive CareUniversity Hospital SouthamptonSouthamptonUK; Haematology/Transfusion MedicineNHS Blood and TransplantOxfordUK

**Keywords:** Adult, Humans, Aprotinin, Blood Transfusion, Deamino Arginine Vasopressin, Deamino Arginine Vasopressin/therapeutic use, Fibrin Tissue Adhesive, Hemorrhage, Hemorrhage/etiology, Hemorrhage/prevention & control, Tranexamic Acid, Tranexamic Acid/therapeutic use

## Abstract

**Background:**

Vascular surgery may be followed by internal bleeding due to inadequate surgical haemostasis, abnormal clotting, or surgical complications. Bleeding ranges from minor, with no transfusion requirement, to massive, requiring multiple blood product transfusions. There are a number of drugs, given systemically or applied locally, which may reduce the need for blood transfusion.

**Objectives:**

To assess the effectiveness and safety of anti‐fibrinolytic and haemostatic drugs and agents in reducing bleeding and the need for blood transfusion in people undergoing major vascular surgery or vascular procedures with a risk of moderate or severe (> 500 mL) blood loss.

**Search methods:**

We searched: Cochrane Central Register of Controlled Trials; MEDLINE; Embase; CINAHL, and Transfusion Evidence Library. We also searched the WHO ICTRP and ClinicalTrials.gov trial registries for ongoing and unpublished trials. Searches used a combination of MeSH and free text terms from database inception to 31 March 2022, without restriction on language or publication status.

**Selection criteria:**

We included randomised controlled trials (RCTs) in adults of drug treatments to reduce bleeding due to major vascular surgery or vascular procedures with a risk of moderate or severe blood loss, which used placebo, usual care or another drug regimen as control.

**Data collection and analysis:**

We used standard Cochrane methods. Our primary outcomes were units of red cells transfused and all‐cause mortality. Our secondary outcomes included risk of receiving an allogeneic blood product, risk of reoperation or repeat procedure due to bleeding, risk of a thromboembolic event, risk of a serious adverse event and length of hospital stay. We used GRADE to assess certainty of evidence.

**Main results:**

We included 22 RCTs with 3393 participants analysed, of which one RCT with 69 participants was reported only in abstract form, with no usable data. Seven RCTs evaluated systemic drug treatments (three aprotinin, two desmopressin, two tranexamic acid) and 15 RCTs evaluated topical drug treatments (drug‐containing bioabsorbable dressings or glues), including fibrin, thrombin, collagen, gelatin, synthetic sealants and one investigational new agent. Most trials were conducted in high‐income countries and the majority of the trials only included participants undergoing elective surgery. We also identified two ongoing RCTs.

We were unable to perform the planned network meta‐analysis due to the sparse reporting of outcomes relevant to this review.

**Systemic drug treatments**

We identified seven trials of three systemic drugs: aprotinin, desmopressin and tranexamic acid, all with placebo controls. The trials of aprotinin and desmopressin were small with very low‐certainty evidence for all of our outcomes. Tranexamic acid versus placebo was the systemic drug comparison with the largest number of participants (2 trials; 1460 participants), both at low risk of bias. The largest of these included a total of 9535 individuals undergoing a number of different higher risk surgeries and reported limited information on the vascular subgroup (1399 participants).

Neither trial reported the number of units of red cells transfused per participant up to 30 days. Three outcomes were associated with very low‐certainty evidence due to the very wide confidence intervals (CIs) resulting from small study sizes and low number of events. These were: all‐cause mortality up to 30 days; number of participants requiring an allogeneic blood transfusion up to 30 days; and risk of requiring a repeat procedure or operation due to bleeding.

Tranexamic acid may have no effect on the risk of thromboembolic events up to 30 days (risk ratio (RR) 1.10, 95% CI 0.88 to 1.36; 1 trial, 1360 participants; low‐certainty evidence due to imprecision).

There is one large ongoing trial (8320 participants) comparing tranexamic acid versus placebo in people undergoing non‐cardiac surgery who are at high risk of requiring a red cell transfusion. This aims to complete recruitment in April 2023. This trial has primary outcomes of proportion of participants transfused with red blood cells and incidence of venous thromboembolism (DVT or PE).

**Topical drug treatments**

Most trials of topical drug treatments were at high risk of bias due to their open‐label design (compared with usual care, or liquids were compared with sponges). All of the trials were small, most were very small, and few reported clinically relevant outcomes in the postoperative period. Fibrin sealant versus usual care was the topical drug comparison with the largest number of participants (5 trials, 784 participants).

The five trials that compared fibrin sealant with usual care were all at high risk of bias, due to the open‐label trial design with no measures put in place to minimise reporting bias. All of the trials were funded by pharmaceutical companies.

None of the five trials reported the number of red cells transfused per participant up to 30 days or the number of participants requiring an allogeneic blood transfusion up to 30 days.

The other three outcomes were associated with very low‐certainty evidence with wide confidence intervals due to small sample sizes and the low number of events, these were: all‐cause mortality up to 30 days; risk of requiring a repeat procedure due to bleeding; and risk of thromboembolic disease up to 30 days.

We identified one large trial (500 participants) comparing fibrin sealant versus usual care in participants undergoing abdominal aortic aneurysm repair, which has not yet started recruitment. This trial lists death due to arterial disease and reintervention rates as primary outcomes.

**Authors' conclusions:**

Because of a lack of data, we are uncertain whether any systemic or topical treatments used to reduce bleeding due to major vascular surgery have an effect on: all‐cause mortality up to 30 days; risk of requiring a repeat procedure or operation due to bleeding; number of red cells transfused per participant up to 30 days or the number of participants requiring an allogeneic blood transfusion up to 30 days.

There may be no effect of tranexamic acid on the risk of thromboembolic events up to 30 days, this is important as there has been concern that this risk may be increased.

Trials with sample size targets of thousands of participants and clinically relevant outcomes are needed, and we look forward to seeing the results of the ongoing trials in the future.

## Plain language summary

**Are there any drugs which help reduce bleeding after surgery on blood vessels?**

**• Key messages**

We do not yet know what the best drugs are to reduce bleeding and blood transfusions during vascular surgery.

**• What is vascular surgery?**

Vascular surgery is when a surgeon operates on blood vessels, to repair leaks and areas of weakness, or to clear blockages. This review focused on the types of vascular surgery that are more likely to lead to severe bleeding.

**• Why is it important to reduce blood transfusions during vascular surgery?**

If people bleed a lot during or after this type of surgery they may need blood transfusions to replace the blood they have lost. It is better to avoid receiving a blood transfusion, if possible, because blood transfusions can cause harm. This is especially important when health services have limited blood supplies.

**• What did we want to find out?**

We wanted to find out which drug treatments help reduce bleeding and the need for blood transfusion. We also wanted to find out if these treatments increase the risk of side effects, such as blood clots.

**• What did we do?**

We searched electronic libraries for reports of the most reliable studies (called randomised controlled trials) of drugs to prevent bleeding after surgery on blood vessels.

**• What did we find?**

We found seven trials of drugs injected before surgery to try to reduce the amount of bleeding. We found 15 trials of dressings or glues with drugs in them. These are used to stop bleeding during surgery and are left inside after the operation. We did not find enough information to be sure which drugs are best for reducing bleeding and transfusions during vascular surgery. Often, the people having surgery were not followed up for very long once they left the operating theatre, so we could not find out whether they needed a blood transfusion afterwards.

One trial, of 9535 people having surgery, tested a drug called tranexamic acid injected before surgery. This included 1399 people who had surgery on their blood vessels, but it did not report much information for this group on its own. The one outcome it did report on, specifically for vascular surgery, was whether there was an increased risk of developing a blood clot if tranexamic acid is given. We found that there may be no difference in the risk of developing a blood clot between tranexamic acid and a placebo.

**• What are the limitations of the evidence?**

Most of the trials we found were small, with fewer than 100 people included. This is not enough to be sure if any of these treatments might help people. We need to have trials with many hundreds or even thousands of people included to find out if these drugs helped them recover from surgery.

Most of the trials we found did not collect information about blood transfusions after surgery. We think this might be for a few different reasons. Surgeons may transfer the care of patients they have operated on to the care of other healthcare professionals if they require prolonged postoperative care, e.g. physicians. It is more complicated to do a study if they need to work with other healthcare professionals to find out what happened afterwards.

Also, most of the trials were run by the companies that make the treatments. It is cheaper and easier for them to only look at what happens during surgery, especially if people will accept their product without any information about what happens after surgery.

**• How up to date is this evidence?**

We found all the published trials on this topic up to 31 March 2022. We also found all the trials which have started or are going to be starting soon. The good news is that there is one very large trial on drugs to prevent bleeding in surgery that is already in progress. It is testing tranexamic acid injected before surgery. It plans to recruit 8320 people undergoing a range of surgeries, including surgery on blood vessels, and will report the number of people needing blood transfusions. This will be an important result when the trial is completed, and we hope it will inspire other surgeons to do trials of this sort in future.

## Summary of findings

**Summary of findings 1:** Summary of findings: Tranexamic acid versus placebo

**Drugs to reduce transfusion after major open vascular or endovascular surgery**						
**Patient or population:** people aged 18 and over undergoing vascular surgery **Settings:** surgical department **Intervention:** tranexamic acid (TXA) **Comparison:** control (placebo, usual care or active comparator)						
**Outcomes**	Anticipated absolute effects* (95% CI)		**Relative effect** **(95% CI)**	**No. participants (RCTs)**	**Certainty of the evidence (GRADE)**	**Comments**
	**Risk with placebo**	**Risk with TXA**				
**Red cell transfusions (units per participant)** up to 30 days post surgery	There were no data for red cell transfusions (units per participant) in this comparison.					
**All‐cause mortality** up to 30 days	No deaths occurred in either study arm			100 (1 RCT)	⊕⊝⊝⊝^a^ VERY LOW	We are uncertain whether TXA has any effect on all‐cause mortality up to 30 days after surgery.
**Risk of receiving any allogeneic blood product** (data only available for intraoperative blood product use)	60 per 1000	**40 per 1000** (53 fewer to 177 more)	**RR 0.66** (0.11, 3.95)	100 (1 RCT)	⊕⊝⊝⊝^b^ VERY LOW	We are uncertain whether TXA has any effect on the risk of receiving any allogeneic blood product within 30 days of surgery.
**Risk of reoperation or repeat procedure for bleeding** within 7 days	20 per 1000	**7 per 1000** (20 fewer to 153 more)	**RR 0.33** (0.01, 7.99)	100 (1 RCT)	⊕⊝⊝⊝^c^ VERY LOW	We are uncertain whether TXA has any effect on the risk of reoperation or repeat procedure for bleeding.
**Risk of a thrombotic/thromboembolic event (MI, CVA, DVT, PE)** 30‐day follow‐up	186 per 1000	**205 per 1000** (22 fewer to 67 more)	**RR 1.10** (0.88, 1.36)	1360 (1 RCT)	⊕⊕⊝⊝^d^ LOW	TXA may have little to no effect on the risk of experiencing a thrombotic or thromboembolic event.
*The risk in the intervention group (and its 95% CI) is based on the assumed risk in the comparison group and the **relative effect** of the intervention (and its 95% CI). **CI**: confidence interval; **CVA**: cerebrovascular attack; **DVT**: deep vein thrombosis; **MI**: myocardial infarction; **PE**: pulmonary embolus; **RCT**: randomised controlled trial; **RR**: risk ratio; **TXA**: tranexamic acid						
**GRADE Working Group grades of evidence** **High certainty:** we are very confident that the true effect lies close to that of the estimate of the effect. **Moderate certainty:** we are moderately confident in the effect estimate. The true effect is likely to be close to the estimate of the effect, but there is a possibility that it is substantially different. **Low certainty:** our confidence in the effect estimate is limited. The true effect may be substantially different from the estimate of the effect. **Very low certainty:** we have very little confidence in the effect estimate. The true effect is likely to be substantially different from the estimate of effect.						

^a^ We downgraded the evidence three times for imprecision because of very wide confidence intervals resulting from small study sizes and low event rate. ^b^ We downgraded the evidence once for indirectness because data were only available for the intraoperative time period, and twice for imprecision because of very wide confidence intervals resulting from small study sizes and low event rate. ^c^ We downgraded the evidence three times for imprecision because of very wide confidence intervals resulting from small study sizes and low event rate. ^d^ We downgraded the evidence twice for imprecision because of the wide confidence intervals resulting from small study sizes and low event rate.

**Summary of findings 2:** Summary of findings: Fibrin sealant vs usual care

**Drugs to reduce transfusion after major open vascular or endovascular surgery**						
**Patient or population:** people aged 18 and over undergoing vascular surgery **Settings:** surgical department **Intervention:** fibrin sealant **Comparison:** usual care						
**Outcomes**	Anticipated absolute effects* (95% CI)		**Relative effect** **(95% CI)**	**No. participants (RCTs)**	**Certainty of the evidence (GRADE)**	**Comments**
	**Risk with usual care**	**Risk with fibrin sealant**				
**Red cell transfusions (units per participant)** up to 30 days post surgery	There were no data for red cell transfusions (units per participant) in this comparison					
**All‐cause mortality** up to 30 days	29 per 1000	**13 per 1000** (26 fewer to 22 more)	**RR 0.44** (0.11, 1.76)	585 (3 RCTs)	⊕⊝⊝⊝^a^ VERY LOW	We are uncertain whether fibrin sealant has any impact on all‐cause mortality at 30 days.
**Risk of receiving any allogeneic blood product** up to 30 days	There were no data for the risk of receiving any allogeneic blood product in this comparison					
**Risk of reoperation or repeat procedure for bleeding** within 7 days	62 per 1000	**64 per 1000** (41 fewer to 148 more)	**RR 1.03** (0.31, 3.40)	160 (1 RCT)	⊕⊝⊝⊝^a^ VERY LOW	We are uncertain whether fibrin sealant has any impact on risk of reoperation or repeat procedure for bleeding within 7 days.
**Risk of a thrombotic/thromboembolic event (MI, CVA, DVT, PE)** 30‐day follow‐up	56 per 1000	**7 per 1000** (56 fewer to 269 more)	**RR 0.11** (0.00, 5.84)	39 (1 RCT)	⊕⊝⊝⊝^b^ VERY LOW	We are uncertain whether fibrin sealant has any impact on the risk of experiencing a thrombotic/thromboembolic event within 30 days.
*The risk in the intervention group (and its 95% confidence interval) is based on the assumed risk in the comparison group and the **relative effect** of the intervention (and its 95% CI). **CI**: confidence interval; **CVA**: cerebrovascular attack; **DVT**: deep vein thrombosis; **MI**: myocardial infarction; **PE**: pulmonary embolus; **RCT**: randomised controlled trial; **RR**: risk ratio						
**GRADE Working Group grades of evidence** **High certainty:** we are very confident that the true effect lies close to that of the estimate of the effect **Moderate certainty:** we are moderately confident in the effect estimate: The true effect is likely to be close to the estimate of the effect, but there is a possibility that it is substantially different **Low certainty:** our confidence in the effect estimate is limited: The true effect may be substantially different from the estimate of the effect **Very low certainty:** we have very little confidence in the effect estimate: The true effect is likely to be substantially different from the estimate of effect						

^a^ We downgraded the evidence once for risk of bias in the domains of blinding of participants and personnel, and we downgraded twice for imprecision due to very wide confidence intervals resulting from small study sizes and low event rate. ^b^ We downgraded the evidence once for risk of bias in the domain of blinding and selective outcome reporting, and we downgraded three times for imprecision due to extremely wide confidence intervals resulting from small study sizes and low event rate.

## Background

### Description of the condition

Vascular surgery treats diseases of arteries, veins or lymph vessels, except for those in the heart or brain. The major types of arterial disease are arterial aneurysms, arterial dissections and arterial occlusive disease. These conditions may be treated either with open surgery by vascular surgeons, or with endovascular procedures conducted by vascular surgeons or interventional radiologists (Hirsch 2006). The availability of interventional radiology procedures varies regionally and globally, depending on the availability of trained staff and equipment (Beck 2016; Benson 2020; Boyle 2021; Kline 2017).

#### Aneurysms

Aneurysms are abnormal dilations in an artery which can progressively enlarge and weaken, with risk of rupture and severe internal bleeding. Vascular services manage aneurysms found in the chest (thoracic aortic aneurysm, TAA), chest and abdomen (thoraco‐abdominal aortic aneurysm, TAAA) or abdomen (abdominal aortic aneurysm, AAA) as well as aneurysms found in peripheral arteries, including iliac, popliteal or femoral arteries. Aneurysms can remain asymptomatic, but rupture can be fatal or life‐threatening. Aneurysm repair can be conducted as an emergency in the case of leak or rupture, or electively to prevent rupture and other complications. Elective repair of AAA is associated with significantly reduced mortality of 2% compared to emergency repair mortality of 20% to 30% (Majd 2016). AAA is the most common type of aneurysm to require repair, whether with open surgery or endovascular repair (Sampson 2014). Risk factors for AAA include older age, male gender, European ancestry, smoking and high blood pressure (Altobelli 2018). Ultrasound screening programmes estimate AAA prevalence at 2.7% in 65 to 74‐year‐olds and 7.3% in 75 to 85‐year‐olds (Makrygiannis 2016). The Centers for Disease Control and Prevention (CDC) ranks AAA as one of the top 15 causes of mortality in the USA for those between 85 and 89 years old (CDC 2015).

#### Arterial dissection

Arterial dissection is a process in which blood tracks between the layers of an artery wall, forcing them apart. This can be an acute or chronic process, initiated by a defect in the vessel due to shear stress, inflammation, trauma or at the site of an aneurysm. Seventy per cent of major vessel dissections occur in the ascending thoracic aorta (Stanford type A), 7% in the thoracic arch, 20% in the descending thoracic or thoraco‐abdominal aorta (Stanford type B) and 2% in the abdominal aorta (Howard 2014; Roberts 1991). Stanford type A requires urgent or emergency open surgical or endovascular repair, whilst Stanford type B may also be managed by reducing blood pressure with medications (Bannazadeh 2016; Cooper 2016; Elsayed 2017; Ulug 2012). Risk factors for aortic dissection include inherited or acquired connective tissue disorders, high blood pressure and aortic aneurysm (Paterick 2013). Acute aortic dissection has an estimated incidence of 52 per 100,000 per year, 60% occur in men, and it has a high risk of death (approximately 73% 30‐day mortality if Stanford type A, and 13% mortality if Stanford Type B) (Howard 2013). If the aortic valve is involved in thoracic aortic aneurysm or dissection, surgical repair may require cardiac surgeons.

#### Occlusive arterial disease

Occlusive arterial disease (OAD) is caused by atherosclerosis, in which fat and cholesterol deposition cause inflammation, thickening and hardening of vessel walls, with eventual narrowing or blockage of the artery (Rahman 2017). This can cause inadequate blood flow through the vessel, with poor oxygen delivery to tissues beyond it (ischaemia). Increased blood flow through other (collateral) vessels may compensate to some degree (McDermott 2017). Atherosclerotic deposits (plaques) can also rupture, suddenly blocking blood flow with blood clot (thrombus) and debris, causing sudden and severe (critical) ischaemia and resultant tissue, organ or limb death (Gilliland 2017). OAD risk factors include being male, older age, personal or family history, cardiovascular disease, diabetes, stroke, high cholesterol, high blood pressure, smoking, obesity and inactivity. OAD can occur in many locations, but vascular services typically treat blockage or narrowing (stenosis) of arteries in the neck (carotid arteries), abdomen (aorta), pelvis and legs (iliac, femoral or popliteal arteries). Carotid stenoses or occlusions cause 15% to 25% of strokes (Saw 2014). Carotid OAD can be managed with open surgery (endarterectomy) or endovascular stents (Noiphithak 2017). Occlusive disease in the aorta is classified as above (supra‐renal) or below (infra‐renal) the artery to the kidneys and can also be managed with open bypass grafting surgery or endovascular stenting.

OAD most commonly affects the lower limbs, where it is also known as peripheral arterial disease (PAD) (Fowkes 2017). It is defined by ankle to brachial (upper arm) blood pressure index (ABPI) of less than 0.9. The prevalence of asymptomatic PAD in the middle‐aged to elderly population is estimated at 7% to 15%, and affects over eight million Americans (Swaminathan 2014). The PERART study (a Spanish primary care population) found a PAD prevalence of 10.2% in males and 5.3% in females (Alzamora 2010). The National Health and Nutrition Examination Survey (NHANES, 1999 to 2000) reported a symptomatic PAD prevalence of 4.3% in adults aged over 40 years old and 14.5% in adults over 70 years old (Selvin 2004). However, the British Regional Heart Study, using femoral artery ultrasound assessment, found 64% of subjects aged 56 to 77 years had significant femoral atherosclerosis, of which only 10% were symptomatic (Leng 2000). When vascular disease causes ischaemia with resultant tissue death, vascular surgeons may perform an amputation at the lowest unaffected level, for example below‐knee, above‐knee or hind‐quarter amputation. Retrospective studies show that non‐traumatic amputations are nearly all caused by vascular disease, which may or may not be complicated by diabetes, and have a high risk of death (mortality of 30% at 30 days and 54% at one year) (Kristensen 2012).

#### Open vascular and endovascular procedures

Open aneurysm or bypass surgery, particularly in the chest, abdomen and pelvis, are invasive major operations associated with complications including bleeding, stroke, cardiac and kidney injury and spinal cord ischaemia, with relatively long recovery and length of hospital stay, and high readmission rates (Fry 2018; Hobson 2018). They may also require periods of aortic cross‐clamping, which adds to complication rates (Zammert 2016). Where cost‐effective and feasible, procedures are conducted endovascularly with stents and grafts, guided by contrast dye and radiological imaging. Endovascular procedures avoid large incisions, cause less postoperative pain, and may have lower mortality and complication rates with reduced hospital length of stay and costs. Intraoperative or postoperative bleeding can occur in endovascular surgery from the vascular access site, around the graft (endoleak), or from vessel rupture. Conversion to open surgery or repeat endovascular procedures are sometimes necessary. Endoleaks (Types I to V) are defined as a persistent blood flow outside the lumen of an endoluminal graft but within the aneurysm sac or the adjacent vascular segments (Society for Vascular Surgery). They are caused by incomplete sealing or exclusion of the aneurysm sac. Endovascular procedures are now feasible in most elective and some emergency settings, particularly in high‐income countries, but remain inappropriate for some complex procedures and require expertise and equipment that may not be available in some settings (Buck 2014).

A 2014 Cochrane Review found that for elective AAA repair, endovascular aneurysm repair (EVAR) was associated with lower short‐term mortality than open surgical repair (OSR), particularly with regard to respiratory complications. At intermediate and long‐term follow‐up, however, they performed comparably. Additionally, individuals undergoing EVAR had a higher re‐intervention rate to manage endoleaks, but these were mostly catheter‐based interventions associated with low mortality and were not associated with any difference in terms of 30‐day mortality (Paravastu 2014). Elective AAA repair may also be conducted with laparoscopic (keyhole) surgery (Robertson 2017). A 2017 Cochrane Review found that for urgent or emergency repair of ruptured AAA, EVAR and OSR had similar 30‐day mortality rates, but did not find a difference in complication rates (Badger 2017). A 2016 Cochrane Review found no randomised controlled evidence to support thoracic endovascular aneurysm repair (TEVAR) compared to open surgical thoracic aorta repair; observational studies, however, support the use of TEVAR (Abraha 2016). A 2017 Cochrane Review found that endovascular treatment (percutaneous transluminal angioplasty) for chronic limb ischaemia was associated with fewer early complications and shorter hospital stay compared with bypass grafting surgery. However, open surgical treatment had better flow in vessels one year on. Endovascular treatment of lower limb occlusive arterial disease may therefore be particularly beneficial in people with significant comorbidities which make them high‐risk surgical candidates (Antoniou 2017). In general, surgical or interventional radiologist experience and anatomy of the defect determine whether endovascular or open surgery is preferable.

#### Bleeding and transfusion in vascular procedures

Internal bleeding may occur before surgery (in the case of arterial dissection or rupture), during an intervention, or after an intervention, due to inadequate surgical haemostasis, abnormal clotting, graft failure, migration or endoleaks. Bleeding ranges from minor, with no transfusion requirement, to massive, requiring multiple blood product transfusions.

Operations or procedures with a risk of moderate or severe blood loss (at least 500 mL) include: open or endovascular emergency repair of AAA, TAAA or TAA; open or endovascular repair of thoracic aortic dissection; complex lower limb bypass surgery; and major lower limb amputation. Studies show transfusion rates of 38% in people undergoing elective open AAA repair, 27% in lower limb bypass surgery, 15% in open thromboendarterectomy (removal of blood clot and atherosclerotic plaque), and vary from 17% to 64% in lower limb amputation (D'Ayala 2010; O'Keeffe 2010; Tan 2015).

Some procedures, for example elective endovascular AAA repair or endovascular lower limb stenting, have a low risk of bleeding (Obi 2015). However, NICE 2020 has recommended that open surgical repair of unruptured AAAs should be performed unless it is contraindicated, due to the increase in medium‐ and long‐term harms of EVAR that outweigh its short‐term benefits. EVAR is associated with fewer perioperative deaths, and less time in hospital in general (and critical care in particular). But it has worse long‐term survival than open surgical repair, and more long‐term complications, leading to further procedures (NICE 2020). Other procedures very rarely cause people to experience bleeding or require transfusion; for example, a transfusion rate of less than 1% in elective carotid endarterectomy has been reported (Rubinstein 2013). Importantly, transfusion rates and transfusion practices vary between centres and care providers (Osborne 2018).

Various surgical factors can increase risk of bleeding, including emergency procedures, for example for aneurysm rupture, dissection or critical limb ischaemia, revision or repeat surgery or complex or branching anatomy (Obi 2015). Perioperative factors include systemic anticoagulation with heparin to prevent graft thrombosis or clot extension, pre‐existing use of anticoagulants or antiplatelet drugs, intraoperative hypothermia, cross‐clamp position, acute coagulopathy in the setting of trauma, and systemic inflammatory response in the setting of an infectious disease, for example aortic mycotic abscess (Obi 2015; Samoila 2017). Procedure‐specific models have been developed to predict bleeding for certain vascular procedures (Kapma 2017; Mahmood 2018). For example, using data from a large multicentre quality improvement database, the transfusion rate within 24 hours of EVAR was predicted at 3.2%, with the following risk factors associated with transfusion: haematocrit less than 36%, increased aortic diameter, functional status and chronic obstructive pulmonary disease (O'Donnell 2018).

#### Interventions to reduce bleeding and allogeneic transfusion

Cell salvage can be used to collect blood from the surgical field for autologous transfusion, and meta‐analyses of vascular surgery randomised controlled trials suggest cell salvage reduces perioperative transfusions by up to 37% (Ashworth 2010; Takagi 2009). However, 30% to 50% of surgical blood loss is absorbed into swabs, therefore swab washing can increase blood available for autotransfusion (Haynes 2005). Using a range of techniques to reduce bleeding may be more useful than cell salvage alone; moreover, cell salvage is not used during endovascular procedures. The ratio of different blood products transfused also appears to be important to people's outcomes, as well as the overall amount of blood product transfused (using a higher or lower transfusion trigger) (Mesar 2017). Strategies to reduce use of any allogeneic blood products include techniques such as arterial cross‐clamping, medications to reduce blood pressure and thus reduce bleeding, and limiting use of crystalloid or other fluid infusions, which can compound bleeding by diluting clotting factors present in the circulation (Chee 2016). In addition, point‐of‐care viscoelastic testing (rotational thromboelastometry (ROTEM) or thromboelastography (TEG)) quantifies coagulation and fibrinolysis parameters and their use can guide and reduce autologous transfusion, though most evidence is in the context of cardiac surgery (Wikkelsø 2016). Finally, various haemostatic drugs, which alter coagulation and fibrinolysis, are an important part of management to reduce bleeding and transfusion risk.

### Description of the intervention

When an injury occurs, the formation of a blood clot (normal haemostasis) stops excessive bleeding. Blood clot formation is initiated by tissue injury, endothelial and collagen exposure and release of factors which cause blood vessel constriction (vasoconstriction) and platelet activation (Blanco 2017). Activated platelets stick together, forming a weak plug (Mackman 2007). Multiple enzyme pathways are also activated and amplified, finally producing thrombin, an enzyme that converts fibrinogen to fibrin. Fibrin rapidly polymerises and cross‐links with platelets to form an insoluble, stable blood clot. The clot is further stabilised and contracted by cross‐linking between the fibrin strands by factor XIII (Chapin 2015).

To prevent harmful, unregulated clot extension beyond the injury, blood clots are subsequently contained and broken down by fibrinolysis (Blanco 2017). The enzyme plasmin, a protease, cuts through the fibrin mesh, releasing soluble fragments that are metabolised in the liver and kidneys (Hudson 2017). Plasmin is activated locally from its precursor, plasminogen, as part of the normal clotting process. Plasmin formation and fibrinolytic processes normally occur more slowly than coagulation, such that clot breakdown occurs well after clot formation and tissue remodelling ‐ that is, after bleeding has stopped (Chapin 2015). To prevent plasmin digesting non‐clot tissue or proteins, plasminogen is predominantly converted to plasmin at the site of and within the blood clot, creating bound, rather than free plasmin. Free plasmin will indiscriminately digest plasma proteins, including clotting factors and is normally kept in check and neutralised by circulating alpha‐2‐plasmin inhibitor (Madurska 2018). This reduces pathological, rather than physiological fibrinolysis (Makar 2010).

Antifibrinolytic drugs inhibit the activity of plasmin and thus reduce the breakdown of fibrin within blood clots, resulting in greater early and persistent clot strength (Okamoto 1997). Haemostatic drugs are a broad class of drugs which each act on distinct parts of the coagulation cascade to replace or enhance missing or poorly functioning pro‐coagulant enzymes, substrates or factors. These could be deficient due to inherited conditions, such as haemophilia, or acquired conditions, such as prolonged bleeding (consumption of clotting factors), liver failure, autoimmune disease or drug therapy.

#### Antifibrinolytic drugs

##### Tranexamic acid (TXA)

TXA is a synthetic analogue of the amino acid lysine. It binds reversibly to lysine receptor sites on plasminogen, prevents activation of plasminogen into plasmin, and reduces fibrin breakdown. This improves clot formation, stability and duration. TXA has been well validated for use in perioperative, obstetric and trauma care, as well as in cardiac surgery (Henry 2011; Ker 2015; Shakur 2018). A systematic review and network meta‐analysis of antifibrinolytic adverse drugs effects in the setting of cardiac surgery suggests TXA use reduces mortality compared to placebo or aprotinin. In addition, it does not increase myocardial infarction (MI), cerebrovascular attack (CVA) or renal failure or dysfunction (Hutton 2012). In high doses, however, TXA has been associated with seizures in the cardiac surgery setting (Murkin 2010).

##### ε‐aminocaproic acid (EACA)

EACA is another synthetic lysine analogue, with a similar mechanism of action to tranexamic acid. Comparative potency of EACA and TXA estimates vary but suggest EACA is 7 to 10 times less potent than tranexamic acid (Thomsen 2006). There is no known association with seizures.

Antifibrinolytic drugs such as EACA and TXA are usually administered intravenously after induction of anaesthesia. Usually, a loading dose is given followed by continuous infusion. High doses appear to be more effective than low doses (Henry 2011). Neither TXA nor EACA has been associated with increased risks of adverse effects (Hutton 2012).

##### Aprotinin

Aprotinin is an enzyme inhibitor with complex effects on haemostasis. It is a competitive inhibitor of various serine proteases, including plasmin and kallikrein (McCarthy 1994). Plasmin inhibition slows the rate of fibrinolysis. Aprotinin exerts a much greater effect on free plasmin, however, with much less effect on bound plasmin. This improves the haemostatic problems caused by excessive or unregulated free plasmin activity, such as consumption of clotting factors. This reduces pathological rather than physiological fibrinolysis (Royston 2015). Kallikrein inhibition reduces factor XIIa activity, which inhibits intrinsic coagulation pathways leading to the formation of thrombin and fibrin. On balance, aprotinin is frequently classed as antifibrinolytic, as it has a net clot‐stabilising effect which outweighs its kallikrein‐mediated anticoagulant effects.

Aprotinin has been associated with a higher rate of adverse effects than the lysine analogues (Henry 2009). Evidence from three observational studies and from a single randomised study, in adults undergoing cardiac surgery, showed an increased risk of renal dysfunction, cardiovascular events, pulmonary embolism and death with aprotinin (Bremerich 2006; Cooper 2006; Mangano 2007; Royston 2015). This led to its withdrawal from many national markets in 2007 (FDA 2007). These data have, however, been revisited and reanalysed, questioning the validity of the conclusions of the four studies (Howell 2013). Despite this, aprotinin remains unavailable or on a restricted license, for example for myocardial revascularisation only, in some countries (Henry 2011).

#### Other haemostatic drugs

##### Desmopressin (DDVAP)

Desmopressin is a synthetic analogue of the human anti‐diuretic hormone, vasopressin. It increases the plasma levels of von Willebrand factor (vWF) two‐ to three‐fold by stimulating vWF release from endothelial cells. vWF plays an important role in platelet adhesion to wound sites, and thus early clot formation, so deficiency of vWF leads to bleeding tendencies. vWF also increases the availability of factor VIII, because factor VIII degrades rapidly if not complexed to vWF. Activated factor VIII is required in the enzyme cascade, which produces thrombin and fibrin. vWF deficiency is the most common clotting disorder and is present in about 1% of the population. Desmopressin is mainly used to treat coagulopathy caused either by deficiency of vWF or factor VIII (haemophilia A), but may also be used before procedures to treat reduced platelet adhesiveness due to drugs like aspirin, or from raised serum urea in the setting of severe renal impairment (Kim 2015).

Desmopressin is typically administered at a dose of 0.3 μg per kg subcutaneously or intravenously and takes approximately 30 minutes to reach peak effectiveness, and this effect lasts up to six to eight hours (Franchini 2007). Increases in vWF, factor VIII levels and in tissue plasminogen activator (tPA) if recurrent dosing is used can potentially increase the risk of arterial or venous thrombotic events; this is an important safety consideration (Franchini 2007; Kaufmann 2003). Desmopressin also results in release of nitric oxide from endothelial cells, which can cause vasodilation with symptoms of facial flushing, tachycardia, and hypotension (Kaufmann 2003). In rare cases, desmopressin administration may be associated with hyponatraemia and seizures, especially in young children (Smith 1989).

##### Prothrombin complex concentrate (PCC)

There are two main types of PCC. 3‐factor PCC contains blood clotting factors II, IX and X, whereas 4‐factor PCC also contains blood clotting factor VII, protein C, and protein S. PCC is a powder concentrate, extracted from human plasma and reconstituted prior to use, dosed at 25 to 50 units per kg. It is used for perioperative prophylaxis or treatment of severe bleeding in people treated with vitamin K antagonists, like warfarin, or in people with clotting factor deficiencies, whether inherited, for example haemophilia, or acquired, such as in severe liver disease (BNF 2019). Side effects include fever, high blood pressure and thromboembolism (migrating blood clots).

##### Recombinant factor VIIa (rFVIIa)

rFVIIa, also called NovoSeven, is a serine protease which catalyses conversion of factors IX and thrombin (X) into active forms. This increases the conversion of fibrinogen to fibrin by thrombin and promotes clot formation and propagation. It is currently licensed only for bleeding in people with a diagnosis of haemophilia, or severe uncontrolled haemorrhage, but is also used for prevention of haemorrhage in haemophiliacs undergoing invasive procedures like surgery (Simpson 2012). Studies have suggested an association with rFVIIa and arterial thromboembolic events (Levi 2010; Simpson 2012).

##### Factor XIII (FXIII)

FXIII, is a transglutaminase enzyme which cross‐links fibrin monomers between adjacent fibrin polymer strands to stabilise and strengthen the clot. It also acts to contract the clot into a more dense and insoluble unit (Ariëns 2002). FXIII treatment is currently indicated for congenital or acquired factor XIII deficiencies, identified with quantitative methods, and has been studied as an agent that can reduce bleeding in cardiac surgery (Muszbek 2008).

##### Fibrinogen concentrate

Fibrinogen is a plasma glycoprotein synthesised by the liver. Fibrinogen is the precursor to fibrin, but also helps platelets activate and aggregate by binding to the platelet’s GPIIb/IIIa receptor. Fibrinogen substitution is believed to normalise and improve the environment for clot formation by providing sufficient amounts of substrate and by enhancing the strength and speed of clot generation in people with depleted or dysfunctional fibrinogen (Nielsen 2005a; Nielsen 2005b). Within the context of cardiac surgery, systemic fibrinogen replacement is currently indicated for prophylaxis or treatment of bleeding in congenital and acquired deficiencies of fibrinogen that have been identified with quantitative methods (Bracey 2017). It has, however, been associated with small reduction in transfusions in a Cochrane Review of people with bleeding in elective and cardiac surgery, though without survival benefit (Wikkelsø 2013).

#### Internal topical agents (excludes surface dressings)

Internal topical application of drugs or biomaterials can be used as an adjunct to surgical control of bleeding, particularly where there are many microscopic bleeding vessels or raw tissue which cannot be surgically closed (Gabay 2013). A biomaterial is any substance that has been engineered to physically interact with biological tissue for a specific purpose (Park 2007). Topical agents include active drugs or clotting factors applied directly as a liquid, paste, foam or gel, or impregnated into biomaterials, or application of passive biomaterials which promote clotting through physical means (Vyas 2013). There are many agents available, and these have been classified as active, passive and combined haemostatic agents (Bracey 2017). They can also be classified as flowable, or non‐flowable, or fibrin and non‐fibrin sealant.

Active agents enhance enzyme pathways in clotting and include antifibrinolytic drugs, fibrin sealants or topical thrombin. Passive materials include collagens, porcine gelatins, regenerated oxidised cellulose and polysaccharide spheres. Passive synthetic sealants include cyanoacrylate, polyethylene glycol, and bovine serum albumin with glutaraldehyde. Combination agents include liquid gelatins with thrombin, and fibrin sealants with equine collagens. These diverse groups have the advantage of acting locally at the site of bleeding, potentially avoiding systemic side effects (Seyednejad 2008). The passive biomaterial and sealants may have the advantage of promoting clotting even in hypothermia or with deficits in normal clotting factors, as they operate independently of enzymatic biological clotting processes.

### How the intervention might work

#### Antifibrinolytic drugs

Hyperfibrinolysis can contribute to catastrophic bleeding by preventing new clots forming as well as degrading formed clots. This is because fibrin degradation products interfere with platelet activation, adhesion and normal fibrin polymerisation, inhibiting normal coagulation. Additionally, the high level of free plasmin associated with hyperfibrinolysis also causes degradation of the fibrin precursor fibrinogen, reducing the substrate available for fibrin polymerisation. Prophylactic antifibrinolytic use is recommended for all surgery expected to have moderate or severe blood loss (often defined as at least 500 ml blood loss), unless there are specific contraindications (Chee 2016; Kozek‐Langenecker 2017; NICE 2015; WHO 2021).

#### Other haemostatic drugs

Other haemostatic drugs are currently only recommended where a pre‐existing clotting factor deficiency has been identified with quantitative testing. There is a lack of well‐conducted studies to assess the impact of haemostatic drugs in people who may acquire perioperative deficits in clotting factors or have platelet function deficits due to perioperative medications. DDVAP may be of particular benefit in people with bleeding stemming from GPIIb/IIIa inhibitors and other antiplatelet medications (Raja 2006). rFVIIa is used off‐label for a variety of major surgeries, occasionally as prophylaxis, or more frequently in catastrophic haemorrhage after other options have failed to arrest bleeding. Its usefulness in reducing bleeding in surgery remains unproven (Simpson 2012). Analysis of rFVIIa usage in intractable bleeding in cardiothoracic surgery demonstrated a reduction in transfusion requirement, at the expense of a higher thrombotic event rate; it has not been determined, however, whether this translates into more favourable clinical outcomes (Omar 2015). Fibrinogen may be used during massive transfusion, or acquired hypofibrinoginaemia during major bleeding, but is not routinely used (Chee 2016; Kozek‐Langenecker 2017). Therefore, FXIII, rFVIIa and fibrinogen concentration may be used as a rescue treatment in severe bleeding rather than as prophylaxis due to their cost and risk profile.

#### Internal topical agents

Several trials have shown improved local haemostasis and reductions in overall blood use with topical agents, and there are theoretical advantages of localised treatments in terms of avoiding unwanted side effects (Vyas 2013). In people with abnormal clotting, however, local active treatments which rely on coagulation pathways to work may also have limited effect due to systemic coagulation derangement.

### Why it is important to do this review

Bleeding and reoperation for bleeding are serious adverse outcomes, which are associated with increased mortality, complications, and risk of transfusion (Shaw 2013). Bleeding and the need for a red blood cell transfusion have also been shown to increase the duration of hospital stay and the costs associated with surgery, after taking into consideration confounding factors (Stokes 2011; Zbrozek 2015). The negative impact on outcomes associated with allogeneic transfusion is observed even when a person only receives a transfusion of one or two units of red blood cells (Paone 2014; Paone 2018). These findings have recently been replicated in studies of major vascular surgery: after adjustment of major covariates, perioperative transfusion was associated with increased 30‐day mortality and morbidity (specifically myocardial infarction and pneumonia) in people undergoing major vascular surgery (Obi 2015). In lower limb bypass surgery, transfusion was associated with increased perioperative wound infection and graft thrombosis in a dose‐dependent fashion (Tan 2015). This has also been demonstrated in amputation surgery (Tan 2013). The particular blood product components transfused (red cells, platelet, fresh frozen plasma) may also impact outcome in AAA rupture surgery (Henriksson 2013). This study showed that the ratio of platelets and fresh frozen plasma to red cells increased from 0.8 to 0.9 during the study (1992 to 1999 versus 2000 to 2008), which was associated with improved survival.

Why even a small transfusion of red cells may be associated with poorer outcomes is not fully understood. It may be due to a mixture of pro‐inflammatory and anti‐inflammatory molecules within the transfusion, called transfusion‐related immunomodulation (TRIM) (Karsten 2018; Muszynski 2017; Youssef 2017). Other transfusion‐related adverse effects include incompatibility reactions, transfusion‐related acute lung injury (TRALI), and transfusion‐associated circulatory overload (TACO) (Harvey 2015; Maxwell 2006). In addition, transmission of infectious diseases (e.g. HIV, Hepatitis C, prion disease) remains a concern (Kiely 2017; Rerambiah 2014). This is particularly a concern in countries with higher prevalence of infectious diseases, or less robust screening capabilities, or both (Seo 2015; WHO 2017). Blood components, particularly platelets, can also have bacterial contamination that may cause sepsis in the recipient (Benjamin 2016; Makuni 2015; Morel 2013).

Adjuncts to reduce bleeding include prophylactic haemostatic drugs which alter coagulation and fibrinolysis. Tranexamic acid is probably the most frequently used at present, though aprotinin is re‐emerging after its withdrawal in the late 2000s (Royston 2015). These drugs may be given as a single dose, multiple doses or infusion, and before, during or after surgery, or by various different routes (e.g. topically onto a bleeding internal tissue, subcutaneously or intravenously). Audits of elective surgery show that there is poor uptake of pharmacological adjuncts to reduce bleeding (NCABTPG 2017). Barriers to optimal use may include not knowing which drug, drug combination, dose or timing is most effective. These factors are also important for establishing minimum effective doses and appropriate duration of exposure, so that other drug side effects are minimised. In order to select the most appropriate drug (or drug combination), dose, timing and route, the many different ways of giving these drugs should be compared; this requires clarification and review of available evidence. Finally, this review will investigate the effect of antiplatelet/anticoagulant drug use and compare drug efficacy and safety in open and endovascular procedures, to establish any different performance of drugs in different circumstances (Berger 2012).

## Objectives

To assess the effectiveness and safety of anti‐fibrinolytic and haemostatic drugs and agents in reducing bleeding and the need for blood transfusion in people undergoing major vascular surgery or vascular procedures with a risk of moderate or severe (> 500 mL) blood loss.

## Methods

### Criteria for considering studies for this review

#### Types of studies

We prespecified our methods for conducting this review in the review protocol (Beverly 2020). We included randomised controlled trials (RCTs) and cluster‐RCTs if the analyses accounted for clustering, or if we were able to adequately adjust for clustering (McKenzie 2016). We included all studies regardless of their language or publication status. We excluded studies with purely experimental laboratory outcomes (for example blood tests for inflammatory markers).

#### Types of participants

We included adults (18 years or over) undergoing the following emergency, urgent and elective procedures.

- Open surgical repair (OSR) of aneurysm of the:
  - abdominal aorta (AAA);
  - thoracic aorta (TAA);
  - thoraco‐abdominal aorta (TAAA);
  - iliac artery;
  - femoral artery; or
  - popliteal artery.
- OSR or endovascular repair of dissection of the:
  - abdominal aorta;
  - thoracic aorta; or
  - thoraco‐abdominal aorta.
- Open bypass surgery for peripheral arterial disease of the:
  - aortic artery;
  - iliac artery;
  - femoral artery; or
  - popliteal artery.
- Major lower limb amputation for vascular disease:
  - below knee;
  - above knee; or
  - hindquarter.

We included adults (18 years or over) undergoing the following emergency or urgent procedures.

- Endovascular aneurysm repair (EVAR) of the:
  - abdominal aorta (AAA);
  - thoracic aorta (TAA);
  - thoraco‐abdominal aorta (TAAA);
  - iliac artery;
  - femoral artery; or
  - popliteal artery.
- Endovascular stenting for peripheral arterial disease of the:
  - aortic artery;
  - iliac artery;
  - femoral artery; or
  - popliteal artery

We included participants undergoing surgery with or without aortic cross clamping and with or without use of hypothermia. We included participants undergoing open, modifications of open, and minimally invasive, e.g. laparoscopic, surgical approaches.

We excluded procedures typically performed by or in conjunction with cardiac surgeons, such as those on the ascending aorta and aortic root, or those using coronary artery bypass grafting. These are the topic of a separate ongoing Cochrane Review entitled *Drugs to reduce bleeding and transfusion in adults undergoing cardiac surgery; a systematic review and network meta‐analysis* (Beverly 2019).

We excluded studies involving elective endovascular procedures. We excluded procedures associated with minimal bleeding and transfusion, such as carotid procedures, arterio‐venous fistulae formation for dialysis, varicose vein surgery and upper limb or digit amputations. We also excluded procedures typically performed by neurosurgeons, such as repair of aneurysms or dissection of arteries in the head or neck.

We excluded people with known inherited coagulation disorders, such as von Willebrand factor deficiency, haemophilia or hypofibrinogenaemia. This is because the clotting mechanisms that the drugs promote or interact with may be genetically absent, making response atypical.

For trials consisting of mixed populations of participants (e.g. including children, or including procedures other than those specified), we only used data from participants 18 years or over undergoing the specified procedures, without clotting disorders. If the subgroup data required were not provided, we excluded the trial if less than 80% of participants were eligible to be included.

#### Types of interventions

We included RCTs of the following interventions, compared to usual care, placebo, or each other.

- Tranexamic acid (TXA)
- ε‐aminocaproic acid (EACA)
- Aprotinin
- Desmopressin
- Prothrombin complex concentrate (PCC)
- Recombinant factor VII (rFVII)
- Factor XIII (FXIII)
- Fibrinogen concentrate
- Other topical agents, categorised as:
  - fibrin‐based agents;
  - thrombin‐based agents;
  - synthetic sealants;
  - passive biomaterials; and
  - combination agents.

We included RCTs that compared one or more of the interventions listed above. We included studies using a combination of the above drugs. We did not exclude trials on the basis of the route, dose, timing, or frequency of drug administration. The comparison groups were as defined by the study, which could be a control group using placebo, standard care, or one of the included drugs, if a second additional drug was being investigated.

#### Types of outcome measures

We were primarily interested in postsurgical outcomes, and especially the need for blood transfusion. We did not include intraoperative outcomes, such as time to haemostasis, because these outcomes are prone to measurement bias and are of limited clinical relevance or interest to health services.

##### Primary outcomes

Our primary outcomes were:

- Red cell transfusions (units per participant*) at up to 30 days post surgery
- All‐cause mortality at up to 30 days; and between 31 and 90 days

*If the red cell transfusion outcome was reported in mL, we converted that into units, according to any local mean unit volume data given in the study, or as per the *Guidelines for the Blood Transfusion Services in the UK* mean stated volume per unit of red cells of 280 ± 60 mLs (JPAC 2013).

##### Secondary outcomes

Our secondary outcomes were as follows.

- Risk of receiving any allogeneic blood product at up to 30 days post surgery
  - Composite: packed red cells (PRC), fresh frozen plasma (FFP), platelets (PLTs)
  - Components: PRC, FFP, PLTs
- Risk of reoperation or repeat procedure for bleeding within 7 days
- Risk of a thrombotic/thromboembolic event
  - Composite: myocardial Infarction (MI), cerebrovascular attack (CVA), deep vein thrombosis (DVT), pulmonary embolus (PE) at up to 30 days and between 31 and 90 days
  - Components: MI at up to 30 days, CVA at up to 30 days, DVT at up to 90 days, PE at up to 90 days
- Risk of a serious adverse event (SAE) at up to 30 days postsurgery
- Length of hospital stay (days)

We commented on any cost data, if presented, in a narrative form (Ryan 2016). Cost information was provided as useful additional information, but was not intended to be a formal economic evaluation.

### Search methods for identification of studies

We searched bibliographic databases and checked the references of included studies.

#### Electronic searches

Searches used a combination of MeSH and free text terms and were carried out from database inception to 31 March 2022, without language restriction or publication status.

The Information Specialist (CD) searched the following databases for relevant trials:

- Cochrane Central Register of Controlled Trials (CENTRAL) (Wiley, The Cochrane Library, 2022, Issue 3);
- MEDLINE (Ovid, 1946 to 31 March 2022);
- Embase (Ovid, 1974 to 31 March 2022);
- CINAHL (EBSCOhost, 1982 to 31 March 2022);
- Transfusion Evidence Library (Evidentia Publishing, 1950 to 31 March 2022).

The Information Specialist (CD) also searched the following trials registries.

- World Health Organization International Clinical Trials Registry Platform (ICTRP).
- ClinicalTrials.gov.

The Cochrane sensitivity‐ and precision‐maximising RCT filter (Lefebvre 2011) was applied to Ovid MEDLINE, and adaptations of it to Ovid Embase and CINAHL, in combination with a systematic review filter (to include systematic reviews to allow manual screening for additional citations, see Searching other resources), based on the Scottish Intercollegiate Guidelines Network (SIGN) filter (www.sign.ac.uk/methodology/filters.html). Search strategies are displayed in full in Appendix 1.

#### Searching other resources

We checked the reference lists of all included studies for additional references to trials using SpiderCite. We also examined any relevant retraction statements and errata for included studies.

### Data collection and analysis

We conducted and reported the review in accordance with the *Cochrane Handbook for Systematic Reviews of Interventions* (Higgins 2021) and PRISMA.

#### Selection of studies

Four review authors (AB, PW, CK, JS) used Covidence to screen abstracts of citations identified by the search strategy. Two review authors retrieved and screened the full text of all potentially eligible citations. We translated studies reported in non‐English language journals before assessment. Studies that were ineligible because we could not identify a vascular subgroup are detailed in the Excluded studies table.

Disagreements during screening were resolved by consensus, in consultation with review author LJE where necessary. We recorded the reasons for excluding studies at full text screening.

#### Data extraction and management

Two of four review authors (AB, GO, CK, JS) independently undertook data extraction from included studies. Data extraction forms were designed by AB and GO, and were piloted and modified before use.

We extracted the following data from each study.

- **General information:** country of study, single or multi‐centre, funding source, publication type (abstract/full text/protocol), trial registration and timing (prospective or retrospective), year of publication.
- **Trial details:** trial design, aims of the trial, funding, location, setting, number of centres, number of treatment arms, intention‐to‐treat analysis, power calculation and whether reached, treatment allocation method, randomisation, blinding, total number recruited, total number randomised, total number analysed in each study group, dropout rate, participant inclusion and exclusion criteria, antiplatelet and anticoagulant cessation protocol, transfusion strategy, comparability of groups according to participants' characteristics, length of follow‐up, stopping rules, thrombotic event definition, SAE definition.
- **Characteristics of participants:** age, sex, weight, preoperative antiplatelet and anticoagulant medication (including washout period).
- **Characteristics of surgery:** type of vascular operation, risk stratification, urgency of surgery (e.g. elective, non‐elective, mixed, not stated), surgical duration, aortic cross‐clamp use, aortic cross‐clamp duration, use of hypothermia, mean minimum temperature, percentage in each arm dropping out (with reasons), percentage in each arm lost to follow‐up.
- **Characteristics of intervention:** number of arms, description of intervention and comparison arms, description of control arms (including placebo, usual care etc.), intervention(s) given, route of administration of intervention, timing of intervention, methods of dosing (e.g. standard, dose/kg, dose categories), dose, dose delivery (single bolus, multiple bolus, infusion).

##### Grouping interventions into treatment nodes for data synthesis

The included studies used a range of different interventions and control treatments, and we grouped them by type of intervention and comparator. There were not enough network connections, or data, to perform a network meta‐analysis, so we have presented these as pairwise comparisons, grouped by systemic drugs (all of which were compared to placebo) and topical dressings or glues (with a number of different comparators, including some placebo sponges).

#### Assessment of risk of bias in included studies

Two of four review authors (GO, AB, CK, JS) independently assessed the risk of bias using the Cochrane risk of bias 1 tool (RoB 1) (Higgins 2017). We resolved any disagreements by discussion.

We had planned to use the Confidence in Network Meta Analysis (CiNeMA 2017) tool, but this was not done because no network meta‐analysis was performed. We used the GRADE criteria to summarise the certainty of evidence for pairwise meta‐analysis.

This and other deviations from the published protocol are described in the Differences between protocol and review section.

#### Measures of treatment effect

We expressed measures of treatment effect using the criteria laid out by Cochrane for dichotomous outcomes and continuous outcomes (Higgins 2022).

For dichotomous outcomes, we recorded the number of events and total number of participants in treatment and control groups. For continuous outcomes, we recorded the mean, standard deviation and total number of participants in both the treatment and control groups, and median, range or interquartile range.

For dichotomous variables, we expressed the results as risk ratio (RR) with 95% confidence intervals (CI). Where the number of observed events was small (less than 5% of sample per group) and the trials had balanced treatment groups, we reported Peto's OR with 95% CI (Deeks 2017).

Where outcomes, for example red cell transfusions, were reported with different units (mLs, mL/kg, units) we converted these to the desired units (e.g. units of packed red cells) where possible.

Where we could not synthesise the data, we provided a descriptive narrative summary and tables with the available information. When we could not report available data in any of the formats described above, we provided a narrative report and, when appropriate, presented the data in tables.

#### Unit of analysis issues

We considered participants as the unit of analysis (McKenzie 2016).

We did not find any cluster‐randomised trials, or any trials with more than two eligible arms.

#### Dealing with missing data

We did not contact authors for missing data because most of the missing data was due to the trials not collecting information on the outcomes of relevance to this review.

#### Assessment of heterogeneity

Where the clinical and methodological characteristics of individual studies were sufficiently homogenous, we combined the data to perform a meta‐analysis (Deeks 2017). In standard pairwise meta‐analyses, we estimated the heterogeneity variances for each pairwise comparison.

##### Measures and tests for heterogeneity

During initial data extraction, we assessed if clinical and methodological heterogeneity were present by looking at trial and person characteristics across all included trials.

We summarised statistical heterogeneity using Tau^2^ and I^2^.

#### Assessment of reporting biases

We recorded the prespecified outcomes for each trial, where available, and compared them to reported outcomes. There were not enough data to explore small‐study biases; most of the included studies were very small.

#### Data synthesis

We had planned to perform a network meta‐analysis (NMA) but could not do so due to the large number of treatments with very little data to populate the network. The planned NMA methods were outlined in the protocol (Beverly 2020), and we will undertake this planned analysis should sufficient data be available in future updates.

##### Methods for direct treatment comparisons

We used RevMan Web to perform pairwise meta‐analysis (RevMan Web 2020), pooling data with a random‐effects model unless there were rare events, in which case we used Peto's OR (if the arms were balanced), which is only available using a fixed‐effect model. We presented the results as the pooled treatment effect with its 95% CI, alongside estimates of Tau^2^ and I^2^, and reported all data that could not be included in meta‐analyses in the characteristics of Included studies section.

#### Subgroup analysis and investigation of heterogeneity

##### Treatment effect modifiers

We planned to investigate potential effect modifiers by carrying out the following subgroup analyses. However, we were unable to perform any of these analyses due to a lack of data.

- Endovascular versus open surgery
- Perioperative antiplatelet and anticoagulant therapy
- Aortic cross‐clamp use
- Hypothermia use

#### Sensitivity analysis

We planned to do sensitivity analyses based on risk of bias and broad versus narrow treatment groupings, and to explore the impact of missing data, but there were insufficient data for any sensitivity analysis to be informative.

#### Summary of findings and assessment of the certainty of the evidence

We were unable to do a network meta‐analysis, so we have not used CiNeMA as specified in the protocol. We have detailed this deviation from the protocol in Differences between protocol and review.

We created summary of findings tables using GRADEPro (Schünemann 2021), and used RevMan Web 2020 to present the main findings of this review. We included the following outcomes in the summary of findings tables.

- Red cell transfusions (units per participant) up to 30 days post surgery
- All‐cause mortality at up to 30 days
- Risk of receiving any allogeneic blood product up to 30 days post surgery
- Risk of reoperation or repeat procedure for bleeding within 7 days
- Risk of a thrombotic/thromboembolic event

We used the five GRADE considerations (risk of bias, inconsistency, imprecision, indirectness, and publication bias) to assess the certainty of the evidence as related to the studies reporting on the prespecified outcome (Atkins 2004). We used the methods described in the *Cochrane Handbook for Systematic Reviews of Interventions* (Higgins 2021; Schünemann 2021). When we downgraded the certainty of evidence we explained our decisions using footnotes, and added comments to aid the reader's understanding of the review when needed.

## Results

### Description of studies

See Characteristics of included studies and Characteristics of excluded studies.

#### Results of the search

See PRISMA flow diagram (Figure 1).

**1:**
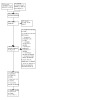
Flow diagram

We searched electronic databases up to 31 March 2022 and identified a total of 8328 records. We also screened a further 397 records that were referenced by the included trials, using SpiderCite. We removed 84 duplicates, leaving 8641 records for further assessment.

On initial assessment of the titles and abstracts of these 8641 records, we excluded 8481 records as irrelevant. Of the remaining 160 records, we excluded 120 studies (135 records) after screening the full text against eligibility criteria (see Excluded studies for further details). Four studies appeared to meet the inclusion criteria but did not report sufficient data to allow a decision on eligibility to be made (jRCTs041180163; NCT00618358; NCT00652314; NCT04083807); details are given in Studies awaiting classification).

We identified 24 potentially eligible trials, two ongoing trials (ChiCTR1900023323; NCT04803747) and 22 completed trials that were eligible for inclusion (Bajardi 2009; Bochicchio 2015; Chetter 2017; Clagett 1995; Czerny 2000; EUCTR2016‐003661‐26‐PL; Giovanacci 2002; Joseph 2004; Leijdekkers 2006; Lethagen 1991; Milne 1996; Minkowitz 2019; Monaco 2020; NCT02094885; Nenezic 2019; O'Donnell 2010 (abstract only); POISE‐3 2022; Qerimi 2013; Ranaboldo 1997; Robinson 2000; Taylor 2003; Weaver 2002).

#### Included studies

We included 22 RCTs, summarised in Table 3 with more detail for each trial in the Characteristics of included studies.  One trial with 69 participants was reported in abstract form only without sufficient data to include in any of our analyses. The remaining 21 trials reported a total of 3324 participants analysed.  The total randomised is unknown, as many trials did not explicitly report it.

**1:** Overview of included studies

**Study ID**	**Country (centres); dates**	**No randomised (analysed) by intervention vs control**	**Population**	**% Elective**	**Intervention**	**Control**	**Comparison**
**Bajardi 2009**	Italy (1); June 2007 to June 2008	10 (10) vs 10 (10)	Patients undergoing replacement of infra‐renal AAA. Indirectness: no 85% male Mean age: 72.7 years (range 63 to 82)	100%	TachoSil	Usual care	Topical drugs: thrombin + fibrin/collagen sponge vs usual care
**Bochicchio 2015**	Netherlands (6), UK (10), USA (9); May 2012 to April 2013	118 (117) vs 58 (58)	Patients undergoing arterial bypass 78%, arteriovenous graft formation for haemodialysis access 11%, carotid endarterectomy 9%, other 2%. Indirectness: no 68.8% male Mean age 65.6 years	NR	Fibrocaps liquid	Gelatin sponge	Topical drugs: fibrin sealant vs gelatin sponge
**Chetter 2017**	Canada (5), Spain (7), UK (7); March 2013 to December 2015	111 (110) vs 57 (57)	Patients undergoing open arterial surgery Indirectness: "20.9% of fibrin sealant arm and 19.3% of usual care arm underwent carotid endarterectomy with patch angioplasty." 79.8% male Mean age: 65.5 years	100%	Fibrin sealant (Grifols)	Usual care (manual compression with gauze)	Topical drugs: fibrin sealant vs usual care
**Clagett 1995**	USA (1); NR	43 (43) vs 48 (48)	Patients undergoing infra‐renal aortic aneurysm repair or aortofemoral bypass surgery. Indirectness: all patients were male 100% male Mean age: 63 years	100%	Desmopressin	Placebo	Systemic drugs: desmopressin
**Czerny 2000**	Austria (1), Germany (2); NR	NR (30) vs NR (30)	Patients undergoing vascular reconstruction surgery with PTFE prostheses. Indirectness: no 73% male Mean age: 65.8 years	100%	TachoComb H	Usual care (manual compression)	Topical drugs: fibrin/collagen sponge vs usual care
**EUCTR2016‐003661‐26‐PL**	Bosnia and Herzegovina, Croatia, Poland, UK, Serbia (16 centres total); NR	NR (36) vs NR (18)	Patients undergoing a planned open liver/soft tissue surgery, vascular surgery or spine surgery. Indirectness: no, vascular subgroup reported 78% male Mean age: 68.1 years	100%	Peprostat soaked gelatin sponge	Saline soaked gelatin sponge	Topical drugs: novel agent/gelatin sponge vs oxidised cellulose
**Giovanacci 2002**	Switzerland (3) July 1998 to January 2001	NR (79) vs NR (81)	Patients undergoing femoral artery surgery with inguinal access. Indirectness: no 59% male Mean age: 71 years	69%	Fibrin glue	Usual care	Topical drugs: fibrin sealant vs usual care
**Joseph 2004**	UK (3) NR	12 (11) vs 12 (11)	Patients undergoing femoral anastomosis and femoral or carotid patch angioplasty with PTFE grafts. Indirectness: no % male NR Mean age: 68.4 years (range 51 to 86)	NR	Tachocomb H	Usual care	Topical drugs: thrombin + fibrin/collagen sponge vs usual care
**Leijdekkers 2006**	Netherlands (1); June 1996 to July 2001	16 (16) vs 19 (19)	Patients undergoing repair of an asymptomatic infrarenal aortic aneurysm. Indirectness: no 80% male Median age: 68 years	100%	Aprotinin	Placebo	Systemic drugs: aprotinin
**Lethagen 1991**	Sweden (1); NR	25 (22) vs 25 (22)	Patients undergoing aorto‐iliac surgery. Indirectness: no 74% male Mean age: NR	100%	Desmopressin	Placebo	Systemic drugs: desmopressin
**Milne 1996**	Scotland (1); NR	21 (21) vs 18 (18)	Patients undergoing either arterial bypass surgery with a PTFE bypass graft or aortic aneurysm repair with a woven Dacron graft. Indirectness: no 77% male Median age: Fibrin sealant: 73 years; Usual care: 70 years	NR	Fibrin sealant (fibrinogen and thrombin, which are mixed in the presence of factor XI11 and calcium to produce insoluble fibrin)	Usual care	Topical drugs: fibrin sealant vs usual care
**Minkowitz 2019**	USA (20); January 2014 to November 2015	NR (20) vs NR (11)	Adult and paediatric patients undergoing non‐laparoscopic, non endovascular surgical procedure involving a native artery graft end to side proximal anastomosis. Indirectness: yes 17.1% vascular; vascular subgroup reported. Paediatric patients also included. Numbers NR 38% male Mean age: 55.8 years	100%	Human thrombin on Gelfoam sponge	Bovine thrombin on Gelfoam sponge	Topical drugs: fibrin sealant vs usual care
**Monaco 2020**	Italy (1); March 2015 to October 2017	50 (50) vs 50 (50)	Patients undergoing surgical repair for AAA. Indirectness: no 93% male Median age: TXA: 69 years; Placebo: 71 years	100%	Tranexamic acid	Placebo	Systemic drugs: TXA
**NCT02094885**	China (9) NR	125 (125) vs 127 (127)	Patients undergoing elective vascular procedures. Indirectness: no 73% male Mean age: 56.9 SD 12.6	100%	Bioseal (porcine derived fibrin sealant)	Usual care (manual compression)	Topical drugs: fibrin sealant vs usual care
**Nenezic 2019**	Hungary, Russian Federation, Serbia, USA (35 centres in total) August 2012 to December 2015	109 (109) vs 57 (57)	Patients undergoing peripheral vascular procedures. Indirectness: 12.8% (fibrin sealant) and 17.5% (usual care) of participants underwent upper extremity vascular access procedures (not target population) 64.4% male Median age: Fibrin sealant: 64; Usual care: 61 (range 22 to 84)	100%	Fibrin sealant (Grifols)	Usual care (manual compression with gauze)	Topical drugs: fibrin sealant vs usual care
**O'Donnell 2010**	USA (NR); NR	NR (NR) vs NR (NR) [Total 69]	Patients undergoing vascular surgery with anastomotic suture line bleeding. Indirectness: no % male NR Mean age NR	NR	Vascular sealant (no further details)	Gelfoam/Thrombin	Topical drugs: unspecified sealant vs usual care
**POISE‐3 2022**	Australia (17), Austria (1), Belgium (2), Brazil (2), Canada (12), Chile (2), China (4), Denmark (3), France (1), Germany (4), Hong Kong (1), India (14), Italy (4), Malaysia (5), Netherlands (2), New Zealand (3), Pakistan (2), Poland (3), Russian Federation (7), South Africa (3), Spain (7), UK (2), USA (13) (114 centres total); June 2018 to July 2021	699 (699; 684 PPA for safety) vs 700 (700; 676 PPA for safety)	Patients undergoing major vascular surgery, at risk of developing bleeding or cardiovascular complications. (vascular subgroup of a larger trial of non‐cardiac surgery). Indirectness: no % male NR Mean age NR	NR	Tranexamic acid	Placebo	Systemic drugs: TXA
**Qerimi 2013**	Germany (1); February 2009 to July 2009	8 (7) vs 8 (8)	Patients undergoing vascular reconstruction due to peripheral vascular disease with suture hole bleeding of peripheral arterial bypass anastomosis using PTFE graft prosthesis. Indirectness: no 69% male Mean age: Lyostypt: men mean 69.7 SD 7.0 women 80.0; Surgicel: men mean 70.5 SD 2.9 women mean 70.5 SD 8.2	100%	Lyostypt	Surgicel	Topical drugs: collagen dressing vs oxidised cellulose
**Ranaboldo 1997**	UK (1); NR	NR (66) vs NR (62)	Patients undergoing elective aortic reconstruction surgery. Indirectness: no 75.8% male Median age: Aprotinin: 68 years; Placebo: 70 years	100%	Aprotinin	Placebo	Systemic drugs: aprotinin
**Robinson 2000**	UK (9); December 1994 to June 1998	NR (38) vs NR (39)	Patients undergoing emergency repair for AAA. Indirectness: no 86% male Median age: Aprotinin: 74 years; Placebo: 73 years (range 52 to 88)	0%	Aprotinin	Placebo	Systemic drugs: aprotinin
**Taylor 2003**	USA (26); NR	NR (101) vs NR (99)	Patients undergoing elective PTFE grafting including at least one end‐to‐side anastomosis of a PTFE graft to the common femoral artery. Indirectness: no 62.5% male Mean age: 64.0 years	100%	Beriplast (fibrin sealant)	Thrombin‐soaked gelatin sponge	Topical drugs: fibrin sealant vs thrombin/gelatin sponge
**Weaver 2002**	USA (4); NR	43 (43) vs 46 (46)	Patients undergoing reconstructive vascular surgery or arteriovenous access procedures. Indirectness: no % male: NR Mean age: 65.4 years	NR	FloSeal (glutaraldehyde cross linked gelatin with thrombin)	Gelfoam thrombin	Topical drugs: synthetic sealant vs thrombin/gelatin sponge

AAA ‐ Abdominal aortic aneurysm; NR ‐ not reported; PPA ‐ per protocol analysis; PTFE ‐ polytetrafluoroethylene; TXA ‐ tranexamic acid

##### Trial design

All of the included studies were two‐arm parallel randomised controlled trials. Six were multinational trials (Bochicchio 2015; Chetter 2017; Czerny 2000; EUCTR2016‐003661‐26‐PL; Nenezic 2019; POISE‐3 2022); seven single‐country multicentre trials (Giovanacci 2002; Joseph 2004; Minkowitz 2019; NCT02094885; Robinson 2000; Taylor 2003; Weaver 2002), and eight of the trials were single centre trials (Bajardi 2009; Clagett 1995; Leijdekkers 2006; Lethagen 1991; Milne 1996; Monaco 2020; Qerimi 2013; Ranaboldo 1997). O'Donnell 2010 is published as an abstract only, and it is unclear if this was a single or multicentre trial.

##### Trial size

The numbers of participants enrolled in all trials, and relevant for this review, ranged between 16 (Qerimi 2013) and 1399 (POISE‐3 2022); POISE‐3 2022 was a multinational trial comparing tranexamic acid to placebo for higher risk surgical procedures conducted in 22 countries, which recruited 9535 people in total. There were nine trials that included more than 100 participants (Bochicchio 2015; Chetter 2017; Giovanacci 2002; Minkowitz 2019; NCT02094885; Nenezic 2019; POISE‐3 2022; Ranaboldo 1997; Taylor 2003) (Table 3). 

##### Participants

Most of the trials only included people undergoing elective procedures (Table 3). Robinson 2000 was conducted in participants undergoing emergency repair of ruptured abdominal aortic aneurysms, and Giovanacci 2002 included 31% of participants who were undergoing urgent or emergency femoral artery surgery. Six trials did not specify the proportion of elective or urgent surgery (Bochicchio 2015; Joseph 2004; Milne 1996; O'Donnell 2010; POISE‐3 2022; Weaver 2002).

##### Setting

Most of the trials were only conducted in high income countries according to the World Bank classification (Table 3). POISE‐3 2022 was conducted in middle‐ (India and Pakistan) and upper‐middle‐income countries (Brazil, China, Malaysia, Russian Federation and South Africa) as well as high‐income countries. EUCTR2016‐003661‐26‐PL and Nenezic 2019 were conducted in upper‐middle‐income countries (Serbia and Bosnia EUCTR2016‐003661‐26‐PL; Serbia and Russian Federation Nenezic 2019) as well as high‐income countries. NCT02094885 was conducted in China.

##### Intervention

Of the 22 included RCTs: 

- seven assessed systemic drug treatments versus placebo:
  - three aprotinin (Leijdekkers 2006; Ranaboldo 1997; Robinson 2000);
  - two desmopressin (Clagett 1995; Lethagen 1991);
  - two tranexamic acid (Monaco 2020; POISE‐3 2022).
- 15 assessed topical drugs, dressings or glues:
  - nine compared an intervention versus usual care: five assessed fibrin sealant (Chetter 2017; Giovanacci 2002; Milne 1996; NCT02094885; Nenezic 2019); two assessed thrombin plus fibrin/collagen sponge (Bajardi 2009; Joseph 2004); one assessed fibrin/collagen sponge (Czerny 2000); and one assessed an unspecified sealant (O'Donnell 2010);
  - six compared two different interventions: human thrombin gelatin sponge versus bovine thrombin gelatin sponge (Minkowitz 2019); collagen dressing versus oxidised cellulose (Qerimi 2013); fibrin sealant versus thrombin gelatin sponge (Taylor 2003); fibrin sealant versus gelatin sponge (Bochicchio 2015); synthetic sealant versus thrombin gelatin sponge (Weaver 2002); and a new agent (Prepostat) that polymerises fibrinogen versus gelatin sponge (EUCTR2016‐003661‐26‐PL).

We did not identify any completed or ongoing trials that assessed the following interventions: ε‐aminocaproic acid (EACA); prothrombin complex concentrate (PCC); recombinant factor VII (rFVII); factor XIII (FXIII); fibrinogen concentrate. No systemic drugs were compared against each other.

##### Outcomes

One trial did not report any of this review's outcomes (O'Donnell 2010). Many of the trials only reported a limited number of this review's outcomes. Two of these trials were vascular subgroups of larger trials with a mixed surgical population, so there was limited outcome data for the vascular subgroup (EUCTR2016‐003661‐26‐PL; POISE‐3 2022). Table 4 summarises the outcomes reported by the trials.

**2:** Reporting of review endpoints

**Trial ID**	**Max FU**	**RBCu**	**M30**	**M90**	**AABT**	**PRC**	**FFP**	**PLT**	**Redo**	**TE**	**MI**	**CVA**	**DVT**	**PE**	**SAE**	**LoHS**
**Systemic drugs**																
**Aprotinin vs placebo**																
**Leijdekkers 2006**	In‐hospital	Y	Y^a^	*‐*	*‐*	N^b^	N^b^	N^b^	Y	*‐*	*‐*	*‐*	*‐*	*‐*	Y	*‐*^c^
**Ranaboldo 1997**	30 days	≈	Y	*‐*	*‐*	*‐*	*‐*	*‐*	*‐*	*‐*	Y	Y	Y	Y	≈	*‐*
**Robinson 2000**	30 days	≈	Y	*‐*	*‐*	*‐*	N^b^	N^b^	*‐*	*‐*	Y	Y	*‐*	*‐*	Y	≈
**Desmopressin vs placebo**																
**Clagett 1995**	30 days	Y	Y	*‐*	*‐*	Y	N	N	*‐*	Y	Y	N	Y	Y	*‐*	*‐*
**Lethagen 1991**	Unclear	Y	*‐*	*‐*	*‐*	*‐*	*‐*	*‐*	*‐*	≈	≈	*‐*	*‐*	*‐*	≈	*‐*
**Tranexamic acid vs placebo**																
**Monaco 2020**	28 days/1 year	*‐*	Y	N	Y^d^	Y	Y^d^	*‐*	Y	≈	Y	*‐*	Y	Y	*‐*	Y
**POISE‐3 2022**	30 days	*‐*	*‐*^e^	*‐*	*‐*	*‐*^e^	*‐*	*‐*	*‐*^e^	Y	*‐*^e^	*‐*^e^	*‐*^e^	*‐*^e^	*‐*^e^	*‐*^e^
**Trial ID**	**Max FU**	**RBCu**	**M30**	**M90**	**AABT**	**PRC**	**FFP**	**PLT**	**Redo**	**TE**	**MI**	**CVA**	**DVT**	**PE**	**SAE**	**LoHS**
**Topical drugs**																
**Fibrin/collagen sponge vs usual care**																
**Czerny 2000**	Intraoperative (90 days AEs)	*‐*	*‐*	*‐*	Y^d^	*‐*	*‐*	*‐*	*‐*	≈	*‐*	*‐*	*‐*	*‐*	≈	≈
**Thrombin+fibrin/collagen sponge vs usual care**																
**Bajardi 2009**	Unclear	Y^f^	*‐*	*‐*	*‐*	*‐*	*‐*	*‐*	*‐*	≈	Y	*‐*	*‐*	*‐*	*‐*	*‐*
**Joseph 2004**	Intraoperative	*‐*	Y^a^	*‐*	Y^d^	*‐*	*‐*	*‐*	*‐*	Y	*‐*	*‐*	*‐*	*‐*	Y	*‐*
**Human thrombin/gelatin sponge vs bovine thrombin/gelatin sponge**																
**Minkowitz 2019**	Unclear	*‐*	Y^a^	Y	*‐*	*‐*	*‐*	*‐*	*‐*	*‐*	*‐*	*‐*	*‐*	*‐*	N^e^	*‐*
**Collagen dressing vs oxidised cellulose**																
**Qerimi 2013**	30 days	*‐*	Y	*‐*	*‐*	*‐*	*‐*	*‐*	*‐*	Y	Y	Y	Y	Y	Y	*‐*
**Novel agent (Peprostat) vs placebo/gelatin sponge**																
**EUCTR2016‐003661‐26‐PL**	30 days	*‐*	Y	*‐*	*‐*	*‐*	*‐*	*‐*	*‐*	*‐*	*‐*	*‐*	*‐*	Y	Y	*‐*
**Trial ID**	**Max FU**	**RBCu**	**M30**	**M90**	**AABT**	**PRC**	**FFP**	**PLT**	**Redo**	**TE**	**MI**	**CVA**	**DVT**	**PE**	**SAE**	**LoHS**
**Fibrin sealant vs usual care**																
**Chetter 2017**	24 hours	*‐*	Y	‐	*‐*	*‐*	‐	*‐*	*‐*	*‐*	*‐*	*‐*	*‐*	*‐*	≈	*‐*
**Giovanacci 2002**	Unclear	*‐*	*‐*	*‐*	*‐*	*‐*	*‐*	*‐*	Y	*‐*	*‐*	*‐*	*‐*	*‐*	*‐*	*‐*
**Milne 1996**	26 weeks	*‐*	*‐*	*‐*	*‐*	*‐*	*‐*	*‐*	*‐*	Y	*‐*	*‐*	*‐*	*‐*	*‐*	*‐*
**NCT02094885**	30 days	*‐*	Y	*‐*	*‐*	*‐*	*‐*	*‐*	*‐*	*‐*	Y	Y	Y	Y	Y	*‐*
**Nenezic 2019**	7 months	*‐*	Y	*‐*	*‐*	*‐*	*‐*	*‐*	*‐*	*‐*	*‐*	*‐*	*‐*	*‐*	Y^g^	*‐*
**Fibrin sealant vs gelatin sponge**																
**Bochicchio 2015**	29 days	*‐*	Y	*‐*	*‐*	Y	*‐*	*‐*	Y	*‐*	*‐*	*‐*	*‐*	*‐*	Y	*‐*
**Fibrin sealant vs thrombin/gelatin sponge**																
**Taylor 2003**	30 days	*‐*	Y	*‐*	*‐*	*‐*	*‐*	*‐*	*‐*	*‐*	*‐*	*‐*	*‐*	*‐*	*‐*	*‐*
**Synthetic sealant vs thrombin/gelatin sponge**																
**Weaver 2002**	6 to 8 weeks	*‐*	Y	*‐*	*‐*	*‐*	*‐*	*‐*	Y	Y	*‐*	*‐*	*‐*	*‐*	*‐*	*‐*
**Unspecified sealant vs usual care**																
**O'Donnell 2010**	30 days	*‐*	*‐*	*‐*	*‐*	*‐*	*‐*	*‐*	*‐*	*‐*	*‐*	*‐*	*‐*	*‐*	*‐*	*‐*
**Key**																
Max FU	maximum follow‐up															
**Primary outcomes (review)**																
RBCu	red cell transfusions (units per participant) at up to 30 days post surgery															
M30	all‐cause mortality at up to 30 days;															
M90	all‐cause mortality between 31 to 90 days															
**Secondary outcomes (review)**																
AABT	risk of receiving any allogeneic blood product at up to 30 days post surgery (PRC, FFP, PLT composite)															
PRC	packed red cells (PRC)															
FFP	fresh frozen plasma (FFP)															
PLT	platelets (PLTs)															
Redo	risk of reoperation or repeat procedure for bleeding within 7 days															
TE	risk of a thrombotic/thromboembolic event (MI, CVA, DVT, PE composite)															
MI	myocardial Infarction at up to 30 days															
CVA	cerebrovascular attack at up to 30 days															
DVT	deep vein thrombosis at up to 90 days															
PE	pulmonary embolus at up to 90 days															
SAE	risk of a serious adverse event at up to 30 days post surgery															
LoHS	length of hospital stay (days)															
**Reporting of outcome**																
Y	Reported in a form which can be meta‐analysed															
≈	Reported in a form which cannot be meta‐analysed															
I	Measured but not reported															
‐	Not measured															

^a^ Follow‐up not clearly defined ^b^ Units reported ^c^ Length of stay in intensive care unit reported ^d^ Intraoperative only ^e^ Vascular subgroup not reported ^f^ “Peri‐operative” (undefined) ^g^ At six weeks

##### Funding source

Funding sources and statements of independence of authors are summarised in Table 5.

**3:** Industry funding and independence of authors

**Trial ID**	**Industry funded?**	**Statement of independence of authors?**	**Industry employees or consultants on authorship?**
**Bajardi 2009**	NR	^‐^	‐
**Bochicchio 2015**	Yes	No	Yes
**Chetter 2017**	Yes	No	Yes
**Clagett 1995**	NR	‐	‐
**Czerny 2000**	Yes	No	Yes
**EUCTR2016‐003661‐26‐PL**	Yes	Trial registration; no publication available	
**Giovanacci 2002**	Yes	No	No
**Joseph 2004**	Yes	No	No
**Leijdekkers 2006**	Yes	No	No
**Lethagen 1991**	No	NA	NA
**Milne 1996**	No	NA	NA
**Minkowitz 2019**	Yes	No	Yes
**Monaco 2020**	Supported by an unrestricted grant, no direct funding or sponsor control.		
**NCT02094885**	Yes	Trial registration; no publication available	
**Nenezic 2019**	Yes	No	Yes
**O'Donnell 2010**	NR	‐	‐
**POISE‐3 2022**	No	NA	NA
**Qerimi 2013**	Yes	No	Yes
**Ranaboldo 1997**	Yes	No	Yes
**Robinson 2000**	Yes	No	No
**Taylor 2003**	Yes	No	Yes
**Weaver 2002**	Yes	No	No

NR ‐ not reported; NA ‐ not applicable; ‐ Unanswerable

All but three trials were funded by the drug manufacturer (Lethagen 1991; Milne 1996; POISE‐3 2022), or provided no details of funding source (Bajardi 2009; Clagett 1995). One of these trials was funded by an unrestricted grant (Monaco 2020), which implies independence of the authors. Three industry‐sponsored trials did not provide any details of the sponsor's involvement in the trial process (EUCTR2016‐003661‐26‐PL; NCT02094885; O'Donnell 2010). 

Thirteen industry‐funded trials did not include a statement of authorial independence (Bochicchio 2015; Chetter 2017; Czerny 2000; Giovanacci 2002; Joseph 2004; Leijdekkers 2006; Minkowitz 2019; Nenezic 2019; Qerimi 2013; Ranaboldo 1997; Robinson 2000; Taylor 2003; Weaver 2002). Eight trials included employees of the manufacturer on the author list (Bochicchio 2015; Chetter 2017; Czerny 2000; Minkowitz 2019; Nenezic 2019; Qerimi 2013; Ranaboldo 1997; Taylor 2003). Six trials received additional material support from the manufacturer such as grants to some of the authors, or the services of medical writers (Bochicchio 2015; Chetter 2017; Minkowitz 2019; Nenezic 2019; Robinson 2000; Taylor 2003).

##### Ongoing studies

We identified two ongoing trials (Characteristics of ongoing studies). One compares fibrin sealant to an unspecified control and plans to recruit 500 participants (ChiCTR1900023323). The primary outcomes specified are death due to arterial disease and reintervention rates.

The second trial, TRACTION (NCT04803747), compares tranexamic acid against placebo and plans to recruit 8320 participants undergoing a range of different surgeries, including vascular procedures. It was registered in March 2021 and the primary outcomes are the proportion of participants requiring red blood cell transfusion and incidence of venous thromboembolism (DVT or PE). It plans to complete recruitment in April 2023.

#### Excluded studies

We excluded 135 records of 120 studies at the full‐text screening stage. Of these, 26 studies (26 records) were an ineligible intervention. One study (3 records) used a blood product control, and was excluded as it had an ineligible comparator (Morrison 2019), and 59 studies (71 records) had an ineligible population, including those studies where there was a mixed surgical population with no vascular subgroup available. 

Twenty‐eight studies (28 records) were excluded as having an ineligible study design. Where this may be ambiguous, these have been listed in Characteristics of excluded studies (Brunkwall 2007; Koncar 2008; Koncar 2011; Pilon 2010; Sauer 2016; Shimamura 1998). We deemed Clagett 1996 and Thompson 2013 not relevant as they were reviews.

Of the studies we excluded as having an ineligible participant population, nine were excluded at a late stage because they contained mixed populations, with less than 80% of participants meeting our eligibility requirements (Chalmers 2010; Develle 2020; Glickman 2002; Lumsden 2006; NCT00439309; NCT01500135; Saha 2012; Stone 2012; Verhoef 2015). Of these, five studies included more than 20% of the participants receiving arterio‐venous access for haemodialysis, which is not an eligible intervention in this review, and no subgroup of eligible participants was available. The studies were: Chalmers 2010  (33% haemodialysis access); Glickman 2002 (28% haemodialysis); Lumsden 2006 (54% haemodialysis); Saha 2012 (44% haemodialysis) and Stone 2012 (22.7% haemodialysis). We excluded four of the nine studies as we could not establish how many people in each group received arterio‐venous access for dialysis, and subgroup information was not available (Develle 2020; NCT00439309; NCT01500135; Verhoef 2015).

See table of Excluded studies for further details.

##### Studies awaiting classification

Four studies appeared to meet the inclusion criteria but did not report sufficient data to allow a decision on eligibility to be made (jRCTs041180163; NCT00618358; NCT00652314; NCT04083807); details are given in Studies awaiting classification. 

### Risk of bias in included studies

The primary risk of bias identified in this group of studies is the lack of blinding in the trials of topical drug treatments, which is unavoidable with most of these interventions. There were also concerns over the independence of the authors for trials funded by the manufacturer of the product.

Overall, the trials were of reasonable quality, with around a third having a low risk of bias and only around 10% having a high risk of bias for reasons other than a lack of blinding or non‐independence from the funder.

Figure 2 is a summary risk of bias chart for the whole review, The risk of bias assessment for each individual trial is described in detail in the trial summaries (Characteristics of included studies) and Figure 3.

**2:**
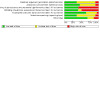
Risk of bias graph: review authors' judgements about each risk of bias item presented as percentages across all included studies.

**3:**
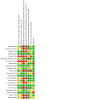
Risk of bias summary: review authors' judgements about each risk of bias item for each included study.

Only two trials were at low risk of bias across all domains (Monaco 2020; POISE‐3 2022).

#### Allocation

Two trials were at high risk of selection bias due to issues with both random sequence generation and allocation concealment (Czerny 2000; Giovanacci 2002).

Twelve trials were at unclear risk of selection bias because the trials gave little or no information about how randomisation was done, or the allocation concealed, or both (Bajardi 2009; Bochicchio 2015; EUCTR2016‐003661‐26‐PL; Joseph 2004; Lethagen 1991; Milne 1996; Minkowitz 2019; NCT02094885; Nenezic 2019; O'Donnell 2010; Ranaboldo 1997; Taylor 2003; Weaver 2002).

Seven trials were at low risk of selection bias for random sequence generation and allocation concealment (Chetter 2017; Clagett 1995; Leijdekkers 2006; Monaco 2020; POISE‐3 2022; Qerimi 2013; Robinson 2000).

#### Blinding

All seven trials that assessed a systemic intervention were at low risk of performance and detection bias due to the use of a matched placebo (Clagett 1995; Leijdekkers 2006; Lethagen 1991; Monaco 2020; POISE‐3 2022; Ranaboldo 1997; Robinson 2000).

Two trials that compared two different topical interventions were at low risk of performance and detection bias (EUCTR2016‐003661‐26‐PL; Minkowitz 2019).

Around 70% of trials that compared two different topical interventions could not be blinded to healthcare workers performing the procedure because of the distinctive appearance of the topical treatments being compared; for example topical liquids were compared to a sponge. However, in these trials there was no description of how participants or outcomes assessors were blinded to the intervention. We classified 11 trials as at high risk of performance and detection bias because there was no evidence of independent outcome assessors who were blinded to the intervention (Bajardi 2009; Bochicchio 2015; Chetter 2017; Czerny 2000; Giovanacci 2002; Milne 1996; NCT02094885; O'Donnell 2010; Qerimi 2013; Taylor 2003; Weaver 2002).

Joseph 2004 and Nenezic 2019 included independent outcome assessors, but there was no information on blinding of participants; we classified these as high risk for performance bias and unclear risk of bias for detection bias.

#### Incomplete outcome data

Fourteen trials were at low risk of attrition bias due to little or no loss to follow‐up (Bajardi 2009; Bochicchio 2015; Clagett 1995; Joseph 2004; Leijdekkers 2006; Milne 1996; Monaco 2020; NCT02094885; Nenezic 2019; POISE‐3 2022; Qerimi 2013; Robinson 2000; Taylor 2003; Weaver 2002).

We only considered one trial to be at high risk of attrition bias, due to the proportion who had been randomised but did not receive the allocated intervention (EUCTR2016‐003661‐26‐PL). The other trials were at unclear risk of attrition bias due to some loss to follow‐up, but this appeared to be balanced between arms (Chetter 2017; Czerny 2000; Giovanacci 2002; Lethagen 1991; Minkowitz 2019; O'Donnell 2010; Ranaboldo 1997).

#### Selective reporting

Around 90% of trials reported all their stated primary and secondary outcomes, although only half had a published protocol or trial registration for verification that these outcomes were prespecified.

Two trials were at high risk of selective reporting bias (Czerny 2000; Milne 1996). In Czerny 2000 the authors stated in their methods section that adverse events were classified as serious or not, but there was no reporting of serious adverse events ‐ only all adverse events. In Milne 1996, the methods stated that they were going to report outcomes, but no numerical data were provided in the results.

Twelve trials were at unclear risk of selective reporting bias (Bajardi 2009; Chetter 2017; Clagett 1995; Giovanacci 2002; Leijdekkers 2006; Lethagen 1991; Minkowitz 2019; O'Donnell 2010; Ranaboldo 1997; Robinson 2000; Taylor 2003; Weaver 2002). In one study this was because serious adverse events were not fully described (Minkowitz 2019). In one study the trial registration referenced is incorrect and we were unable to locate any registration or protocol for the study (Chetter 2017). For one study, the only report available was an abstract with insufficient information to make a judgement about selective outcome reporting (O'Donnell 2010). In the remaining nine studies there was no available trial registration or published protocol (Bajardi 2009; Clagett 1995; Giovanacci 2002; Leijdekkers 2006; Lethagen 1991; Ranaboldo 1997; Robinson 2000; Taylor 2003; Weaver 2002).

Eight studies were at low risk for selective reporting bias (Bochicchio 2015; EUCTR2016‐003661‐26‐PL;Joseph 2004; Monaco 2020; NCT02094885; Nenezic 2019; POISE‐3 2022; Qerimi 2013).

#### Other potential sources of bias

We identified a high risk of bias for 'other' reasons in three trials (Joseph 2004; Minkowitz 2019; Robinson 2000), of which two were because of early stopping decisions that were not fully explained (Joseph 2004; Robinson 2000), and one was because of concerns about the effectiveness of the masking used (Minkowitz 2019). Risk of bias was unclear in the 'other' domain for four trials (O'Donnell 2010; Qerimi 2013; Ranaboldo 1997; Weaver 2002), of which two were because of insufficient information about baseline characteristics (O'Donnell 2010; Weaver 2002). In one trial there was a change in protocol during the study (Ranaboldo 1997), and in one very small trial (16 participants) there was a large baseline imbalance by sex with an unclear impact on outcomes (Qerimi 2013). The remaining studies were at low risk of other bias (Bajardi 2009; Bochicchio 2015; Chetter 2017; Clagett 1995; Czerny 2000; EUCTR2016‐003661‐26‐PL; Giovanacci 2002; Leijdekkers 2006; Lethagen 1991; Milne 1996; Monaco 2020; NCT02094885; Nenezic 2019; POISE‐3 2022; Taylor 2003).

### Effects of interventions

See: Table 1; Table 2

We were able to obtain only very limited data from the trials identified, due to small sample sizes, a lack of reporting of the outcomes we are interested in, and the large number of treatment comparisons (13) relative to the number of trials (22), limiting the amount of information that could be pooled. Network meta‐analysis was not possible and most of the data sought for the pairwise meta‐analyses were unavailable (see Data extraction and management).

One trial did not report any of our review outcomes (O'Donnell 2010), so 21 trials are included in the quantitative analysis.

POISE‐3 2022 was a multinational trial comparing tranexamic acid to placebo for higher risk surgical procedures, which included 1399 people undergoing higher risk vascular surgeries. Of our prespecified review outcomes, only thromboembolic events were reported for the vascular subgroup in this trial.

All the other included trials were small or very small, ranging from 16 to 252 randomised participants, and many did not report our prespecified outcomes. Table 4 summarises the data available for each outcome, grouped by treatment comparison, noting where trials did not measure the outcome or did not report it in a form suitable for meta‐analysis.

The 15 trials of topical dressings or sealants, in particular, were unlikely to report our clinical outcomes of interest. These trials focused on intraoperative outcomes, such as time to haemostasis, with some reporting of adverse events in the weeks following surgery but rarely any postoperative transfusion requirements.

The seven trials of systemic drug treatments were more likely to report transfusion‐related outcomes, although some reported RBC units as medians instead of means and so could not be meta‐analysed. The transfusion‐related outcomes reported by the trials of systemic drug treatments are summarised in Table 6, including information which could not be included in the forest plots.

**4:** Transfusion outcomes for trials of systemic drugs

**Trial ID**	**Red cell transfusions (units per participant) up to 30 days post surgery**	**Risk of receiving any allogeneic blood product at up to 30 days post surgery**			
		**All**	**Red blood cells**	**Fresh frozen plasma (FFP)**	**Platelets**
**Aprotinin vs placebo**					
**Leijdekkers 2006**	**Intraoperative** Aprotinin Mean (SD) 4.1 (3.1) vs Placebo Mean (SD) 4.1 (2.9) P = 0.95 **Postoperative** Aprotinin mean (SD) 0.44 (0.7) vs Placebo mean (SD) 2.0 (7.9) P = 0.43 **Total** NR	NR	NR	NR	**Intraoperative** “In both groups, only one patient received 1 unit of platelets.” Implied intraoperatively. **Postoperative** NR **Total** NR
**Ranaboldo 1997**	**Intraoperative** NR **Postoperative** < 24 hr: Aprotinin median (IQR) 1 (0 to 2) vs Placebo median 1 (0 to 2) > 24 hr: Aprotinin median (IQR) 0 (0 to 0) vs Placebo median (IQR) 0 (0 to 0) **Total** Aprotinin median (IQR) 3 (2 to 5) vs Placebo median (IQR) 3 (2 to 5) See footnote^a^	NR	NR	NR	NR
**Robinson 2000**	**Perioperative** Aprotinin median (range) 7 (0 to 20) vs Placebo median (range) 10 (2 to 32) **Postoperative** Aprotinin median (range) 1 (0 to 14) vs Placebo median (range) 3 (0 to 13) P = 0.02 **Total** Aprotinin median (range) 10 (2 to 29) vs Placebo median (range) 14 (4 to 38) P = 0.053	NR	NR	**Perioperative** NR (units only)	**Perioperative** NR (units only)
**Desmopressin vs placebo**					
**Clagett 1995** ** **	**Intraoperative** Desmopressin mean (SD) 1.9 (2.2) vs Placebo mean (SD) 1.7 (1.8) **Postoperative** Desmopressin mean (SD) 1.2 (1.6) vs Placebo mean (SD) 1.0 (2.0) **Total** Desmopressin mean (SD) 3.1 (3.0) vs Placebo mean (SD) 2.7 (3.0)	NR	**Intraoperative** NR **Postoperative** NR **Total** Desmopressin 33/43 vs Placebo 35/48	NR	NR
**Lethagen 1991**	Converted to units using 280 mL per unit to provide the values for this study in Analysis 1.1 **Intraoperative** Desmopressin mean (SD) 600 (124) vs Placebo mean (SD) 960 (168) **Postoperative** Desmopressin mean (SD) 454 (97) vs Placebo mean (SD) 228 (70) **Total** Desmopressin mean (SD) 1054 (147) vs Placebo mean (SD) 1186 (178)	NR	NR	NR	NR
**Tranexamic acid (TXA) vs placebo**					
**Monaco 2020** ** **	NR	**Intraoperative** TXA 2/50 vs Placebo 3/50 **Postoperative** NR **Total** NR	**Intraoperative** TXA 1/50 vs Placebo 2/50 **Postoperative** TXA 6/50 vs Placebo 10/50 **Total** TXA 7/50 vs Placebo 12/50	**Intraoperative** TXA 1/50 vs Placebo 1/50 **Postoperative** NR **Total** NR	NR
**POISE‐3 2022**	Blood outcomes not reported for vascular subgroup				

IQR ‐ interquartile range; NR ‐ not reported; SD: standard deviation; TXA ‐ tranexamic acid ^a^Numbers in text inconsistent with table referred to in the text: “Aprotinin‐treated patients received a mean of 2.2 (range 1‐12, median 2) units of blood, compared with 1.9 (range 1‐7, median 2) units for control patients. No differences were observed between the aprotinin‐ and placebo‐treated patients for transfusion requirements after operation or for the total amount of blood transfused (Table 3).”

Given the very limited amount of data available, we have only summarised two of the thirteen treatment comparisons in summary of findings tables; tranexamic acid vs placebo (Table 1) because it includes the only trial with a reasonably large sample size, and fibrin sealant vs usual care (Table 2) because it includes more trials (5) than any other comparison.

The data we were able to obtain for each outcome are summarised below.

#### Primary outcomes

##### Red cell transfusions (units per participant) at up to 30 days post surgery

###### Systemic drug treatments

Three trials, one of aprotinin (Leijdekkers 2006) and two of desmopressin (Clagett 1995; Lethagen 1991), reported this outcome in a form that can be meta‐analysed (Analysis 1.1). Two others (Ranaboldo 1997; Robinson 2000) reported medians with range or interquartile range (Table 6).

There is very limited evidence for this outcome for any of the systemic drugs included in this review.

###### Topical drug treatments

One trial of topical treatments reported this outcome (Bajardi 2009). This trial compared thrombin + fibrin/collagen sponge versus usual care for the (undefined) "peri‐operative" period only (Analysis 2.1).

There is very limited evidence for this outcome for any of the topical treatments included in this review.

##### All‐cause mortality at up to 30 days; and between 31 to 90 days

Our other primary outcome, mortality at 30 days and up to 90 days, was reported by a larger proportion of trials than any other outcome, although few had follow‐up beyond 30 days.

###### Systemic drug treatments

Five trials of systemic drugs reported all‐cause mortality up to 30 days, with Leijdekkers 2006 having only in‐hospital follow‐up (Analysis 1.2). No trials reported all‐cause mortality at 90 days; Monaco 2020 reported mortality at one year. Mortality rates were low and all of the trials reporting this endpoint are small, and so the confidence intervals are very wide.

There is very limited evidence for this outcome for any of the systemic drugs included in this review.

###### Topical drug treatments

Ten trials of topical treatments (Analysis 2.2) reported all‐cause mortality up to 30 days, with Joseph 2004 having only in‐hospital follow‐up. Only one trial reported all‐cause mortality up to 90 days (Minkowitz 2019). Mortality rates were low and all of the trials are small, and so the confidence intervals are very wide.

There is very limited evidence for this outcome for any of the topical treatments included in this review.

#### Secondary outcomes

##### Risk of receiving any allogeneic blood product at up to 30 days post surgery

###### Systemic drug treatments

One trial of systemic drugs reported this outcome (Monaco 2020) (Analysis 1.3).

There is very limited evidence for this outcome for any of the systemic drugs included in this review.

###### Topical drug treatments

Two trials of topical treatments reported this outcome for the intraoperative period only (Czerny 2000; Joseph 2004) (Analysis 2.3).

There is no evidence for this outcome for any of the topical treatments included in this review.

##### Risk of reoperation or repeat procedure for bleeding within 7 days

###### Systemic drug treatments

Two trials of systemic drugs reported the need for reoperation (Leijdekkers 2006; Monaco 2020) (Analysis 1.4). Reintervention rates were low, and both trials were small, so the confidence intervals are very wide.

There is very limited evidence for this outcome for any of the systemic drugs included in this review.

###### Topical drug treatments

Three trials of topical treatments reported the need for reoperation (Bochicchio 2015; Giovanacci 2002; Weaver 2002) (Analysis 2.4). Reintervention rates were low and all of the trials were small, so the confidence intervals are very wide.

There is very limited evidence for this outcome for any of the topical treatments included in this review.

##### Risk of a thrombotic/thromboembolic event

###### Systemic drug treatments

Our secondary composite outcome of thromboembolic events (any of MI, CVA/stroke, DVT, PE; Analysis 1.5) was reported by two trials of systemic drugs (Clagett 1995; POISE‐3 2022), with three others (Monaco 2020; Ranaboldo 1997; Robinson 2000) reporting some or all of these events separately (Analysis 1.6; Analysis 1.7; Analysis 1.8; Analysis 1.9).

Thromboembolic events were more common in the trials of systemic drugs, all of which included only major vascular procedures; six of them only included aortic aneurysms. There was no evidence that there was a higher risk of thromboembolic events associated with any of these drugs, but the sample sizes were all too small to rule out clinically important differences. This was the only endpoint available for the vascular subgroup of the large POISE‐3 trial, comparing tranexamic acid with placebo (Table 1).

There is very limited evidence for this outcome for any of the systemic drugs included in this review.

###### Topical drug treatments

Our secondary composite outcome of thromboembolic events (any of MI, CVA/stroke, DVT, PE; (Analysis 2.5) was reported by six trials of topical treatments (Bajardi 2009; Czerny 2000; Joseph 2004; Milne 1996; Qerimi 2013; Weaver 2002), with three other trials reporting some or all of these events separately (EUCTR2016‐003661‐26‐PL; Milne 1996; NCT02094885) (Analysis 2.6; Analysis 2.7; Analysis 2.8; Analysis 2.9).

The reported event rates in trials of topical treatments, which tended to include lower risk surgical procedures than the trials of systemic drugs, were generally very low, with many of these small trials reporting that none or very few events were observed.

There is very limited evidence for this outcome for any of the topical treatments included in this review.

##### Risk of a serious adverse event (SAE) at up to 30 days post surgery

Reporting of adverse events was variable, with some trials not distinguishing between adverse events and serious adverse events, some reporting the number of events rather than the number of people, and some not reporting events split by arm. The data we were able to extract is included in the trial summaries (Characteristics of included studies) and the reporting of this outcome summarised in Table 4. All the reported SAE results are summarised in Table 7.

**5:** Serious adverse events results

**Trial ID**	**SAE**
**Systemic drugs: aprotinin vs placebo**	
**Leijdekkers 2006**	Reports “complications” as 2/16 vs 5/19 OR: 0.4 (95% CI 0.07 to 2.41), P = 0.31 Includes deaths: “In the placebo group, one patient was reoperated for persistent bleeding, who then was resuscitated and developed adult respiratory distress syndrome (ARDS). A second patient was reoperated for persistent bleeding and eventually died due to multiple organ failure. Two patients developed pneumonia, and one developed a wound infection. In the aprotinin group, one patient was reoperated for persistent bleeding due to back‐bleeding from lumbar arteries. He developed colonic ischemia, necessitating a left‐sided hemicolectomy. One patient developed respiratory insufficiency and a fascial dehiscence. One patient died in the aprotinin group due to sepsis after pneumonia.”
**Ranaboldo 1997**	Reported as “complications”, not all serious. Per event, not per person. (Table 4 in published paper)
**Robinson 2000**	Reports “complications” not SAEs, most serious.
**Topical drugs: fibrin/collagen sponge vs usual care**	
**Czerny 2000**	Reports number of events, not number of people. Number of people given in text but for all adverse events: “Throughout the entire study a total of 30 adverse events (31 episodes) were reported in 16 patients in the [TachoComb H] group (including one preoperative adverse event). In the [Control] group 41 adverse events (47 episodes) in 18 patients were reported.”
**Joseph 2004**	2/11 vs 2/11
**Minkowitz 2019**	NR for vascular subgroup
**Topical drugs:collagen dressing vs oxidised cellulose**	
**Qerimi 2013**	1/8 vs 0/8
**Topical drugs: novel agent (Peprostat) vs placebo/gelatin sponge**	
**EUCTR2016‐003661‐26‐PL**	6/36 vs 1/18
**Topical drugs:fibrin sealant vs usual care**	
**Chetter 2017**	Pooled with a non‐randomised exploratory study: 47/187 vs 9/52
**NCT02094885**	13/125 vs 11/127
**Nenezic 2019**	At 6 weeks 21/109 vs 11/57
**Topical drugs: fibrin sealant vs gelatin sponge**	
**Bochicchio 2015**	25/117 vs 12/58

CI ‐ confidence interval; NR ‐ not reported; OR ‐ odds ratio: SAE ‐ serious adverse events

There is very limited evidence for this outcome for any of the treatments included in this review.

##### Length of hospital stay (days)

Only one trial reported means and standard deviation for hospital stay (Monaco 2020). Two other trials reported medians only (Czerny 2000; Robinson 2000), and one reported stay in ICU only (Leijdekkers 2006). The data we were able to extract are included in the trial summaries (Characteristics of included studies) and in Table 8.

**6:** Length of hospital stay results

**Study name**	**Length of hospital stay**	**Number of participants**
**Systemic drugs**		
**Aprotinin vs placebo**		
**Leijdekkers 2006**	Reported length of stay in ICU Aprotinin mean (SD) 44 (61) vs Placebo mean (SD) 120 (228) 95% CI (‐43 to 196) P = 0.20 Aprotinin median (range or IQR unspecified) 22 (19 to 269) vs Placebo median (range or IQR unspecified) 24 (12 to 792) P = 0.55	35 (aprotinin 16; placebo 19)
**Robinson 2000**	Aprotinin median 12 days vs Placebo median 15 days No range or IQR reported. (3 vs 3.5 days in ICU)	77 (aprotinin 38; placebo 39)
**TXA vs placebo**		
**Monaco 2020**	TXA mean (SD) 6 (1.5) vs Placebo mean (SD) 6 (1.2)	100 (TXA 50; placebo 50)
**Topical drugs**		
**Fibrin/collagen sponge vs usual care**		
**Czerny 2000**	Fibrin/collagen sponge mean 10 vs Usual care mean 10.5 SD not reported	60 (fibrin/collagen sponge: 30; usual care: 30)

CI ‐ confidence interval; ICU ‐ intensive care unit; IQR ‐ interquartile range; SD ‐ standard deviation; TXA ‐ tranexamic acid

There is very limited evidence for this outcome for any of the systemic drugs included in this review.

## Discussion

We identified 24 eligible trials, two ongoing trials and 22 completed trials with results available.

### Summary of main results

We included 22 completed trials within this review with 3393 participants analysed. One trial was not included within the quantitative analysis because it did not report any usable data (O'Donnell 2010), leaving 3324 participants analysed for at least one of our outcomes.

The majority of the trials (15/22) were of bioabsorbable dressings or glues and many reported few or no postoperative outcomes. The seven trials of systemic drugs were somewhat more likely to report outcomes of interest for this review.

Apart from the one outcome for the vascular subgroup reported by the POISE‐3 2022 trial, for tranexamic acid compared to placebo, all the review outcomes were of very low‐certainty evidence due to the small size of the trials and the limited reporting of this review's outcomes (Table 4).

The POISE‐3 2022 trial reported thromboembolic events up to 30 days in its vascular subgroup (1399 participants). There may be no difference in the risk of experiencing a thromboembolic event up to 30 days in those that received TXA compared to placebo, with the reported result for the vascular subgroup being a hazard ratio (HR) of 1.10 (95% CI 0.87 to 1.40), consistent with the whole‐trial result for all 9182 participants having high‐risk surgeries: HR 1.02 (0.92 to 1.14).

POISE‐3 2022 did not report any of our bleeding‐related outcomes for the vascular subgroup but did report subgroups by type of surgery for their primary outcome, a composite of life‐threatening, major, and critical organ bleeding. For the vascular subgroup, the hazard ratio (HR) was 0.86 (95% CI 0.64 to 1.13), consistent with the overall result of 0.76 (95% CI 0.67 to 0.87), with little heterogeneity between subgroups defined by the type of surgery.

There are two ongoing trials planning to recruit 500 and 8320 participants respectively. The smaller of these trials is comparing fibrin sealant versus usual care in participants undergoing abdominal aortic aneurysm repair (ChiCTR1900023323). It lists death due to arterial disease and reintervention rates as primary outcomes. The larger trial, TRACTION NCT04803747), is comparing tranexamic acid to placebo in participants undergoing major non‐cardiac surgery that has at least a 5% risk of requiring a red cell transfusion, and has primary outcomes of proportion transfused with red blood cells and incidence of venous thromboembolism (DVT or PE).

Trials with target sample sizes in the thousands and outcomes related to blood transfusion and thromboembolic events are required, and we look forward to seeing the results of these trials, particularly TRACTION (NCT04803747), in the near future. TRACTION is expected to complete recruitment in April 2023.

### Overall completeness and applicability of evidence

This review provides the most up‐to‐date assessment of the effectiveness and safety of drugs to reduce the need for blood transfusion in major open vascular or endovascular surgery.

However, very little data were reported for our primary and secondary outcomes (Table 4). When units of red blood cells were reported, this was not always in a form suitable for meta‐analysis, with around half of these results being reported as medians rather than means.

The majority of trials (15/22) were of bioabsorbable dressings or glues and their primary focus was on intraoperative endpoints, such as time to haemostasis. We rejected this outcome during protocol development as it is prone to bias and of limited clinical relevance. The seven trials of systemic drug treatment, aprotinin (3), desmopressin (2) and tranexamic acid (2) were somewhat more likely to report our outcomes of interest, but there are still substantial gaps and the trials were small.

The lack of clinically relevant outcomes in these trials is likely due to three factors.

1. Some of the included procedures may be considered relatively low risk for postoperative bleeding and so these outcomes were not considered by the trialists. The seven trials involving aortic aneurysms, which includes six of the trials of systemic drugs, were somewhat more likely to report outcomes related to postoperative transfusion requirements.

2. Postoperative follow‐up is logistically challenging and costly, particularly in cases where surgeons may have limited postoperative contact which may be the case after lower‐risk procedures.

3. Most of the included trials were funded by the manufacturers, often with an explicit regulatory or marketing purpose. Clinically relevant outcomes are less likely to be included in trials of this sort because they are of little value to the funder (Svensson 2013).

Even when trials did report clinically relevant outcomes, in all cases their sample sizes were too small to reliably estimate clinically relevant treatment differences. Even for the most widely reported outcome, 30‐day mortality, the confidence intervals were very wide.

### Quality of the evidence

Overall, we rated the certainty of the evidence as very low, according to GRADE methodology, for all but one result across all 13 treatment comparisons identified for this review. This was due to trials being at high or unclear risk of bias, and wide confidence intervals due to small sample sizes. We have formally summarised the GRADE outcome for one systemic drug (Table 1) and one topical sealant (Table 2).

### Potential biases in the review process

We conducted a comprehensive search; searching data sources (including multiple databases, and clinical trial registries) to ensure that all relevant trials would be captured. There were no restrictions for the language in which the paper was originally published. The relevance of each paper was carefully assessed and all screening and data extractions were performed in duplicate. We prespecified all outcomes and subgroups prior to analysis. There were insufficient numbers of included trials within the meta‐analyses for us to use a funnel plot to examine the risk of publication bias.

However, a large proportion of the potentially eligible trials we identified from the literature search included vascular surgery as one of a mixture of surgical procedures, or a mix of eligible and ineligible vascular surgeries. As specified in the protocol, we only included these trials if they reported the eligible vascular subgroup(s) separately (sample size and at least one review outcome), or if at least 80% of surgeries were eligible vascular procedures.

The usefulness of our review was limited by lack of clinically relevant outcome reporting. Once available, we aim to update this review with data from the currently ongoing studies in order to provide better certainty evidence (ChiCTR1900023323; NCT04803747). Where currently we have consulted with vascular surgeons during the review process, we also plan to include a vascular surgeon in the author team for any updates.

### Agreements and disagreements with other studies or reviews

We are not aware of any previous reviews that cover this broad topic of reducing the need for blood transfusion in vascular surgery.

This review is one of a group of five reviews, four of them funded by NIHR and NHSBT, looking at drugs for bleeding due to various causes. The other four focus on cardiac surgery (Beverly 2019), hip and knee replacement (Gibbs 2019a), long bone trauma (Gibbs 2019b), and blunt or penetrating trauma (Erasu 2022). We will consider the similarities and differences between the findings of these reviews, in terms of results, sample sizes and quality of the trials, in joint publications when all the reviews are completed and published.

## Authors' conclusions

**Implications for practice**

We did not identify any evidence which can reliably inform clinical practice. We were able to obtain only very limited data from the trials identified, due to small sample sizes, a lack of reporting of the outcomes we are interested in, and a large number of treatment comparisons (13) relative to the number of trials (22), limiting the amount of information which could be pooled. Network meta‐analysis was not possible and most of the data for the pairwise meta‐analyses were unavailable.

Because of a lack of data, we are uncertain whether any systemic or topical treatments used to reduce bleeding due to major vascular surgery have an effect on all‐cause mortality up to 30 days; risk of requiring a repeat procedure due to bleeding, or the requirement for blood transfusion; number of red cells transfused per participant up to 30 days or the number of participants requiring an allogeneic blood transfusion up to 30 days.

There is no evidence that tranexamic acid increases the risk of thromboembolic events may be no effect of tranexamic acid on the risk of thromboembolic events up to 30 days, an important concern for vascular surgeons. This is important as there has been concern that this risk may be increased.

Trials with sample size targets of thousands of participants and clinically relevant outcomes are needed, and we look forward to seeing the results of the ongoing trials in the future.

**Implications for research**

Vascular surgeons need to do much bigger trials, with follow‐up in the postoperative period. Outcomes related to postoperative transfusion requirements are of interest to the individual, clinicians, and blood services. There is an urgent need for bigger trials in this area with sufficient follow‐up to report clinically relevant endpoints, which are: the need for a red cell transfusion; all‐cause mortality; thromboembolism (venous and arterial); risk of graft thrombosis or occlusion; serious adverse events; length of hospital stay; and need for reoperation. There is one ongoing trial (TRACTION, NCT04803747) that meets both of these requirements, and we look forward to seeing the results. Future trials should also report on specific subgroups that have a higher risk of bleeding, such as patients on antiplatelet agents and anticoagulations; patients who are on cardiopulmonary bypass and are made hypothermic during the procedure; and duration of aortic cross‐clamping.

## History

Protocol first published: Issue 6, 2020

## Appendices

### Appendix 1. Search strategies

#### CENTRAL (The Cochrane Library)

#1 MeSH descriptor: [Vascular Surgical Procedures] explode all trees #2 MeSH descriptor: [Amputation] explode all trees #3 MeSH descriptor: [Blood Vessel Prosthesis Implantation] this term only #4 MeSH descriptor: [Vascular Diseases] explode all trees and with qualifier(s): [surgery ‐ SU] #5 MeSH descriptor: [Iliac Artery] this term only and with qualifier(s): [surgery ‐ SU] #6 MeSH descriptor: [Femoral Artery] this term only and with qualifier(s): [surgery ‐ SU] #7 MeSH descriptor: [Popliteal Artery] this term only and with qualifier(s): [surgery ‐ SU] #8 MeSH descriptor: [Aneurysm] explode all trees and with qualifier(s): [surgery ‐ SU] #9 MeSH descriptor: [Aortic Diseases] explode all trees and with qualifier(s): [surgery ‐ SU] #10 MeSH descriptor: [Arterial Occlusive Diseases] explode all trees and with qualifier(s): [surgery ‐ SU] #11 MeSH descriptor: [Arteriovenous Malformations] explode all trees and with qualifier(s): [surgery ‐ SU] #12 MeSH descriptor: [Diabetic Angiopathies] explode all trees and with qualifier(s): [surgery ‐ SU] #13 MeSH descriptor: [Peripheral Vascular Diseases] explode all trees and with qualifier(s): [surgery ‐ SU] #14 MeSH descriptor: [Venous Insufficiency] explode all trees and with qualifier(s): [surgery ‐ SU] #15 MeSH descriptor: [Radiology, Interventional] this term only #16 (interventional radiolog* or surgical radiolog*) #17 ((vascular or vessel* or aneurysm or aortic or aorta or AAA or TAA or TAAA or aortofemoral or artery or arterial or interarterial or arterioarterial or atheroma or carotid or vein or venous or endovascular* or intravascular*) near/6 (surg* or operat* or reconstruct* or repair* or resection* or repair* or bypass* or graft* or homograft* or transplant* or bypass* or transpos* or implant* or reimplant* or clamp* or ligat* or crossover* or cross‐over*)) #18 ((endovascular or intravascular or laparoscop* or angioscop*) near/3 (surg* or operat* or reconstruct* or repair* or resection* or repair* or bypass* or graft* or homograft* or transplant* or bypass* or transpos* or implant* or reimplant* or procedure*)) #19 (bypass graft* or by‐pass graft* or bypass surgery or by‐pass surgery or enarterectom* or (minimally invasive near/1 surg*) or teflon graft* or dacron graft*) #20 (arterial dilation* or endoluminal repair*) #21 ((venous or arterial) near/3 catheteri?ation) #22 ((aort* or iliac or femoral or popliteal or femoropop* or fempop* or crural) near/3 (surg* or operat* or bypass or graft or reconstruct* or revascular*)) #23 (angiosurg* or aneurysmectom* or aneurysm clipping* or aortopexy or aortoplast* or arteriotom* or arterioplast* or artery plast* or artery stripping or venostom* or portacaval anastomoses or revasculari?ation or devasculari?ation) #24 ((femoral* or iliac* or aorta* or aortic* or infrapopliteal or popliteal or infra‐pop*) near/3 (stent* or angioplast*)) #25 (endovascular near/6 (dissection* or stent*)) #26 (EVAR or FEVAR or TEVAR or embolectom* or thrombectom* or endarterectom* or thromboendarterectom* or thromboembolectom* or atherectom*) #27 (mechanical* near/3 (thrombolysis or clot disruption*)) #28 (axillo bifemoral bypass graft* or axillo femoral bypass graft* or axillobifemoral bypass graft* or axillofemoral bypass graft*) #29 ((ilio femor* or ilio femor* or infrarenal or infra‐renal or infrainguinal or infra‐inguinal) next (bypass* or by‐pass*)) #30 ((pedal or tibial) near/3 (angio* or bypass* or by‐pass*)) #31 ((lower limb* or knee* or leg* or foot or feet or lower extremit* or hindquarter*) near/5 (salvag* or saving or save* or angioplast* or graft* or bypass* or by‐pass* or revascula* or reconstruct* or amputat*)) #32 ((blood vessel* or vascular or endovascular or vein or venous or arterial or artery or arteriovenous or arterio‐venous or brescia cimino or venoarterial or veno‐arterial or arterioportal) near/3 (anastomosis or shunt*)) #33 #1 or #2 or #3 or #4 or #5 or #6 or #7 or #8 or #9 or #10 or #11 or #12 or #13 or #14 or #15 or #16 or #17 or #18 or #19 or #20 or #21 or #22 or #23 or #24 or #25 or #26 or #27 or #28 or #29 or #30 or #31 or #32 #34 MeSH descriptor: [Antifibrinolytic Agents] this term only #35 MeSH descriptor: [Tranexamic Acid] this term only #36 MeSH descriptor: [Aminocaproic Acid] explode all trees #37 (antifibrinolytic* or anti‐fibrinolytic* or antifibrinolysin* or antiplasmin* or plasmin inhibitor* or tranexamic or tranhexamic or cyclohexanecarboxylic acid* or amcha or t‐amcha or amca or transamin or amchafibrin or anvitoff or spotof or cyklokapron or femstrual or ugurol):ti,ab #38 (AMCHA or amchafibrin or amikapron or amstat or antivoff or caprilon or cl65336 or cyclocapron or cyclokapron or cyklocapron or cyklokapron or exacyl or frenolyse or fibrinon or hemostan or hexacapron):ti,ab #39 (hexakapron or kalnex or lysteda or rikaparin or ronex or theranex or tranexam or tranexanic or tranexic or "trans achma" or transexamic or trenaxin or TXA):ti,ab #40 (fibrinolysis near/2 inhibitor*):ti,ab #41 (Agretax or Bio‐Stat or Capiloc or Capitrax or Clip Inj or Clot‐XL or Clotawin‐T or Coastat or Cuti or Cymin or Dubatran or Espercil or Examic or Existat or Extam or Fibran or Gynae‐Pil or Hemstate or Kapron or Menogia or Monitex or Nestran or Nexamic or Nexi‐500 or Nexmeff or Nicolda or Nixa‐500 or Pause or Rheonex or Sylstep TX or Synostat or T‐nex or T Stat or T Stat or Tanmic or Temsyl‐T or Texakind or Texanis or Texapar or Texid or Thams or Tonopan or Traklot or Tramic or Tramix or Tranarest or Trance Inj or Tranecid or Tranee or Tranemic or Tranex or Tranexa or Tranfib or Tranlok or Transtat or Transys or Transcam or Tranxi or Trapic or Traxage or Traxamic or Traxyl or Trenaxa or Trexamic or Trim Inj or Tx‐1000 or Tx 500 or Wistran or X‐Tran or Xamic):ti,ab #42 (ecapron or ekaprol or epsamon or epsicaprom or epsicapron or epsilcapramin or epsilon amino caproate or epsilon aminocaproate or epsilonaminocaproic or epsilonaminocapronsav or ethaaminocaproic or ethaaminocaproich or emocaprol or hepin or ipsilon or neocaprol or resplamin or tachostyptan):ti,ab #43 (lederle or acikaprin or afibrin or amicar or caprocid or capracid or capramol or caprogel or caprolest or caprolisin* or caprolysin* or capromol or epsikapron or hemocaprol or caproamin or EACA or caprolest or capralense or hexalense or hamostat or hemocid):ti,ab #44 (aminohexanoic or aminocaproic or aminohexanoic or amino caproic or amino‐caproic or amino‐n‐hexanoic):ti,ab #45 #34 or #35 or #36 or #37 or #38 or #39 or #40 or #41 or #42 or #43 or #44 #46 MeSH descriptor: [Aprotinin] this term only #47 (antagosan or antilysin* or aprotimbin or apronitin* or aprotinin* or bayer a128 or contrical or contrycal or contrykal or dilmintal or frey inhibitor or kontrycal or Kunitz inhibitor or gordox or haemoprot or kallikrein‐trypsin inactivator or iniprol or kontrikal or kontrykal or kunitz pancreatic trypsin inhibitor or midran or pulmin or tracylol or trascolan or trasilol or tra?ylol or traskolan or zymofren or pancreas antitrypsin or protinin or riker 52g or Rivilina zymofren):ti,ab #48 #46 or #47 #49 MeSH descriptor: [Factor VIIa] this term only #50 (factor viia or factor 7a or rfviia or fviia or novoseven* or novo seven* or aryoseven or acset or eptacog* or proconvertin):ti,ab #51 (activated near/1 (factor seven or factor vii or rfvii or fvii)):ti,ab #52 (factor seven or factor vii or factor 7):ti #53 #49 or #50 or #51 or #52 #54 MeSH descriptor: [Fibrinogen] this term only #55 ("fibrinogen concentrate" or "factor I" or Haemocomplettan* or Riastap* or Fibryga* or Fibryna*):ti,ab #56 #54 or #55 #57 MeSH descriptor: [Deamino Arginine Vasopressin] this term only #58 (desmopressin* or vasopressin deamino or D amino D arginine vasopressin or deamino 8 d arginine vasopressin or vasopressin desamino 8 arginine or desmotabs or DDAVP or desmogalen or adin or adiuretin or concentraid or d void or dav ritter or deamino 8 dextro arginine vasopressin or deamino 8d arginine vasopressin or deamino dextro arginine vasopressin or deaminovasopressin or defirin or defirin melt or desmirin pr desmomelt or desmopresina or desmospray or desmotab* or desurin or emosint or enupresol or minirin or minirinette or minirinmelt or minrin or minurin or miram or nictur or noctisson or nocturin or nocutil or nordurine or novidin or nucotil or octim or octostim or presinex or stimate or wetirin):ti,ab #59 #57 or #58 #60 MeSH descriptor: [Factor XIII] explode all trees #61 (factor xiii or fxiii or fibrin stabili?ing factor* or Tretten* or Catridecacog):ti,ab #62 #60 or #61 #63 MeSH descriptor: [Tissue Adhesives] explode all trees #64 MeSH descriptor: [Collagen] explode all trees and with qualifier(s): [therapeutic use ‐ TU] #65 MeSH descriptor: [Thrombin] explode all trees and with qualifier(s): [therapeutic use ‐ TU] #66 MeSH descriptor: [Gelatin] explode all trees and with qualifier(s): [therapeutic use ‐ TU] #67 MeSH descriptor: [Gelatin Sponge, Absorbable] this term only #68 ((fibrin* or collagen or cellulose or gelatin or gel or thrombin* or albumin or hemostatic* or haemostatic*) next (glu* or seal* or adhesive* or topical* or local* or matrix or matrices or spong* or fleece* or foam* or scaffold* or patch* or sheet* or bandag* or aerosol* or dressing* or paste or powder*)):ti,ab #69 ((nonfibrin* or non‐fibrin* or synthetic* or non‐biological* or nonbiological* or biological*) near/3 (glue* or seal* or adhesive*)):ti,ab #70 (surgical* near/3 (glue* or sealant* or adhesive*)):ti,ab #71 ((fibrin* or collagen or cellulose or gelatin or thrombin) near/3 (hemosta* or haemosta*)):ti,ab #72 (8Y or Aafact or Actif‐VIII or Advate or Artiss or Bioglue or Biocol or Collaseal or Omrixil or Transglutine or Raplixa or Evarrest or Aleviate or Alphanate or Amofil or Beriate or Beriplast or Biostate or Bolheal or Cluvot or Conco‐Eight‐HT or Crosseel or Crosseal or Crosseight or Emoclot or Evarrest or Evicel or Factane or Fanhdi or Fibrogammin P or Green VIII or Green VIII Factor or Greengene or Greenmono or Greenplast or Haemate or Haemate P or Haemate P or Haemate P500 or Haemate‐P or Haemoctin or Haemoctin SDH or Haemoctin‐SDH or Hemaseel or Hemaseal or Hemofil M or Hemoraas or Humaclot or Humafactor‐8 or Humate‐P or Immunate or Innovate or Koate or Koate‐DVI or Kogenate Bayer or Kogenate FS or Monoclate‐P or NovoThirteen or Octafil or Octanate or Octanate or Optivate or Quixil or Talate or Tisseel or Tisseal or Tissel or Tissucol or Tricos or Vivostat or Voncento or Wilate or Wilnativ or Wilstart or Xyntha):ti,ab #73 (Glubran or Gluetiss or Ifabond or Indermil or LiquiBand or TissuGlu or Evithrom or Floseal or Hemopatch or Gel‐Flow or Gelfoam or Gelfilm or Recothrom or Surgifoam or Surgiflo* or "rh Thrombin" or Thrombi‐Gel or Thrombi‐Pad or Thrombin‐JMI or Thrombinar or Thrombogen or Thrombostat):ti,ab #74 (porcine gelatin or bovine collagen or bovine gelatin or nu‐knit or arista or hemostase or vita sure or thrombin‐jmi or thrombinjmi or avicel or vivagel or lyostypt or tabotamp or arterx or omnex or veriset):ti,ab #75 (polysaccharide next (sphere* or hemostatic powder)):ti,ab #76 MeSH descriptor: [Chitosan] this term only #77 MeSH descriptor: [Polyethylene Glycols] this term only and with qualifier(s): [therapeutic use ‐ TU] #78 MeSH descriptor: [Hydrogel, Polyethylene Glycol Dimethacrylate] explode all trees and with qualifier(s): [therapeutic use ‐ TU] #79 MeSH descriptor: [Polyurethanes] explode all trees and with qualifier(s): [pharmacology ‐ PD, adverse effects ‐ AE, toxicity ‐ TO, administration & dosage ‐ AD, therapeutic use ‐ TU] #80 ((polymer‐derived elastic* or polymer tissue adhesive* or elastic hydrogel* or glutaraldehyde or PEG‐based or polyurethane‐based tissue or polyethylene glycol* or polyvinyl alcohol‐based tissue or PVA‐based tissue or natural biopolymer* or polypeptide‐based or protein‐based or polysaccharide‐based or chitosan or poliglusam or cyanoacrylic or cyanoacrylate or cyacrin or dextran‐based or chondroitin sulfate‐based or mussel‐inspired elastic* or glycol hydrogel or polymer‐based) next (glu* or seal* or adhesive* or topical* or local* or matrix or matrices or spong* or fleece* or foam* or scaffold* or patch* or sheet* or bandag* or aerosol* or dressing* or paste* or powder*)):ti,ab #81 MeSH descriptor: [Cellulose, Oxidized] this term only #82 (absorbable cellulose or resorbable cellulose or oxidi?ed cellulose or carboxycellulose or oxycellulose or cellulosic acid or oxycel or oxidi?ed regenerated cellulose):ti,ab #83 (BioGlue or Progel or Duraseal or Coseal or FocalSeal or ADAL‐1 or AdvaSeal or Pleuraseal or Angio‐Seal or Avitene or Instat or Helitene or Helistat or TDM‐621 or Dermabond or Tissueseal or PolyStat or Raplixa or Spongostan or Surgicel or Surgilux or Tachosil or Traumstem):ti,ab #84 (collagen‐thrombin or thrombin‐collagen or gelatin‐fibrinogen or fibrinogen‐gelatin or gelatin‐thrombin or thrombin‐gelatin or fibrinogen‐thrombin or thrombin‐fibrinogen or collagen‐fibrinogen or fibrinogen‐collagen or microfibrillar collagen or CoStasis or "GRF Glue" or GR‐Dial or Algosterile or TraumaStat or HemCon or ChitoFlex or Celox or QuikClot or WoundStat or Vitagel or TachSeal or TachoComb or Cryoseal):ti,ab #85 #63 or #64 or #65 or #66 or #67 or #68 or #69 or #70 or #71 or #72 or #73 or #74 or #75 or #76 or #77 or #78 or #79 or #80 or #81 or #82 or #83 or #84 #86 MeSH descriptor: [Waxes] explode all trees #87 (bonewax* or bone wax* or bone putty or hemasorb or ostene):ti,ab #88 #86 or #87 #89 MeSH descriptor: [Blood Coagulation Factors] this term only #90 (prothrombin near/5 (complex* or concentrate*)) #91 (PCC* or 3F‐PCC* or 4F‐PCC* or Beriplex* or Feiba* or Autoplex* or Ocplex* or Octaplex* or Kcentra* or Cofact or Prothrombinex* or "Proplex‐T" or Prothroraas* or Haemosolvex* or Prothromplex* or "HT Defix" or Facnyne* or Kaskadil* or Kedcom* or Confidex* or PPSB or Profil?ine* or Pronativ* or Proplex* or Prothar* or ProthoRAAS* or Protromplex* or "Pushu Laishi" or "Uman Complex") #92 #89 or #90 or #91 #93 (((haemosta* or hemosta* or antihaemorrhag* or antihemorrhag* or anti haemorrhag* or anti‐hemorrhag*) next (drug* or agent* or treat* or therap*)) or ((coagulat* or clotting) next factor*)):ti,ab #94 #45 or #48 or #53 or #56 or #59 or #62 or #85 or #88 or #92 or #93 #95 #33 and #94 [In Trials]

#### MEDLINE (Ovid)

1. Vascular Surgical Procedures/  2. Endarterectomy/  3. exp Endovascular Procedures/  4. Axillofemoral Bypass Grafting/  5. Embolectomy/  6. Limb Salvage/  7. exp Amputation/  8. exp Thrombectomy/  9. Vascular Grafting/  10. Arteriovenous Shunt, Surgical/  11. Blood Vessel Prosthesis Implantation/  12. Vascular Diseases/su  13. Femoral Artery/su or Iliac Artery/su or Popliteal Artery/su  14. exp Aneurysm/su  15. exp Aortic Diseases/su  16. exp Arterial Occlusive Diseases/su  17. exp Arteriovenous Malformations/su  18. Diabetic Angiopathies/su  19. exp Peripheral Vascular Diseases/su  20. exp Spinal Cord Vascular Diseases/su  21. Vascular System Injuries/su  22. exp Venous Insufficiency/su  23. or/1‐22  24. Radiology, Interventional/  25. (interventional radiolog* or surgical radiolog*).tw,kf.  26. ((vascular or vessel* or aneurysm or aortic or aorta or AAA or TAA or TAAA or aortofemoral or artery or arterial or interarterial or arterioarterial or atheroma or carotid or vein or venous or endovascular* or intravascular*) adj6 (surg* or operat* or reconstruct* or repair* or resection* or repair* or bypass* or graft* or homograft* or transplant* or bypass* or transpos* or implant* or reimplant* or clamp* or ligat* or crossover* or cross‐over*)).tw,kf.  27. ((endovascular or intravascular or laparoscop* or angioscop*) adj3 (surg* or operat* or reconstruct* or repair* or resection* or repair* or bypass* or graft* or homograft* or transplant* or bypass* or transpos* or implant* or reimplant* or procedure*)).tw,kf.  28. (bypass graft* or by‐pass graft* or bypass surgery or by‐pass surgery or enarterectom* or (minimally invasive adj1 surg*) or teflon graft* or dacron graft*).tw,kf.  29. (arterial dilation* or endoluminal repair*).tw,kf.  30. ((venous or arterial) adj3 catheteri?ation).tw,kf.  31. ((aort* or iliac or femoral or popliteal or femoropop* or fempop* or crural) adj3 (surg* or operat* or bypass or graft or reconstruct* or revascular*)).tw,kf.  32. (angiosurg* or aneurysmectom* or aneurysm clipping* or aortopexy or aortoplast* or arteriotom* or arterioplast* or artery plast* or artery stripping or venostom* or portacaval anastomoses or revasculari?ation or devasculari?ation).tw,kf.  33. ((femoral* or iliac* or aorta* or aortic* or infrapopliteal or popliteal or infra‐pop*) adj3 (stent* or angioplast*)).tw,kf.  34. (endovascular adj6 (dissection* or stent*)).tw,kf.  35. (EVAR or FEVAR or TEVAR or embolectom* or thrombectom* or endarterectom* or thromboendarterectom* or thromboembolectom* or atherectom*).tw,kf.  36. (mechanical* adj3 (thrombolysis or clot disruption*)).tw,kf.  37. (axillo bifemoral bypass graft* or axillo femoral bypass graft* or axillobifemoral bypass graft* or axillofemoral bypass graft*).tw,kf.  38. ((ilio femor* or ilio femor* or infrarenal or infra‐renal or infrainguinal or infra‐inguinal) adj (bypass* or by‐pass*)).tw,kf.  39. ((pedal or tibial) adj3 (angio* or bypass* or by‐pass*)).tw,kf.  40. ((lower limb* or knee* or leg* or foot or feet or lower extremit* or hindquarter*) adj5 (salvag* or saving or save* or angioplast* or graft* or bypass* or by‐pass* or revascula* or reconstruct* or amputat*)).tw,kf. 41. ((blood vessel* or vascular or endovascular or vein or venous or arterial or artery or arteriovenous or arterio‐venous or brescia cimino or venoarterial or veno‐arterial or arterioportal) adj3 (anastomosis or shunt*)).tw,kf.  42. or/24‐41  43. 23 or 42  44. Antifibrinolytic Agents/  45. Tranexamic Acid/  46. Aminocaproic Acid/  47. (antifibrinolytic* or anti‐fibrinolytic* or antifibrinolysin* or antiplasmin* or plasmin inhibitor* or tranexamic or tranhexamic or cyclohexanecarboxylic acid* or amcha or trans‐4‐aminomethyl‐cyclohexanecarboxylic acid* or t‐amcha or amca or "kabi 2161" or transamin or amchafibrin or anvitoff or spotof or cyklokapron or cyclo‐F or femstrual or ugurol or aminomethylcyclohexanecarbonic acid or aminomethylcyclohexanecarboxylic acid or AMCHA or amchafibrin or amikapron or aminomethyl cyclohexane carboxylic acid or aminomethyl cyclohexanecarboxylic acid or aminomethylcyclohexane carbonic acid or aminomethylcyclohexane carboxylic acid or aminomethylcyclohexanecarbonic acid or aminomethylcyclohexanecarboxylic acid or aminomethylcyclohexanocarboxylic acid or aminomethylcyclohexanoic acid or amstat or antivoff or caprilon or cl?65336 or cl65336 or cyclocapron or cyclokapron or cyklocapron or cyklokapron or exacyl or frenolyse or fibrinon or hemostan or hexacapron or hexakapron or kalnex or lysteda or rikaparin or ronex or theranex or tranexam or tranexanic or tranexic or trans achma or transexamic or trenaxin or TXA or (fibrinolysis adj2 inhibitor*)).tw,kf.  48. (Agretax or Bio‐Stat or Capiloc or Capitrax or Clip Inj or Clot‐XL or Clotawin‐T or Coastat or Cuti or Cymin or Dubatran or Espercil or Examic or Existat or Extam or Fibran or Gynae‐Pil or Hemstate or Kapron or Menogia or Monitex or Nestran or Nexamic or Nexi‐500 or Nexmeff or Nicolda or Nixa‐500 or Pause or Rheonex or Sylstep TX or Synostat or T‐nex or T Stat or T Stat or Tanmic or Temsyl‐T or Texakind or Texanis or Texapar or Texid or Thams or Tonopan or Traklot or Tramic or Tramix or Tranarest or Trance Inj or Tranecid or Tranee or Tranemic or Tranex or Tranexa or Tranfib or Tranlok or Transtat or Transys or Transcam or Tranxi or Trapic or Traxage or Traxamic or Traxyl or Trenaxa or Trexamic or Trim Inj or Tx‐1000 or Tx 500 or Wistran or X‐Tran or Xamic).tw,kf.  49. (6‐aminohexanoic or amino?caproic or amino?hexanoic or amino caproic or amino‐caproic or amino‐n‐hexanoic or cy‐116 or cy116 or lederle or acikaprin or afibrin or amicar or caprocid or capracid or capramol or caprogel or caprolest or caprolisin* or caprolysin* or capromol or epsikapron or hemocaprol or caproamin or EACA or caprolest or capralense or hexalense or hamostat or hemocid or cl 10304 or cl10304 or ecapron or ekaprol or epsamon or epsicaprom or epsicapron or epsilcapramin or epsilon amino caproate or epsilon aminocaproate or epsilonaminocaproic or epsilonaminocapronsav or etha?aminocaproic or ethaaminocaproich or emocaprol or hepin or ipsilon or jd?177or neocaprol or nsc?26154 or resplamin or tachostyptan).tw,kf.  50. or/44‐49  51. Aprotinin/  52. (antagosan or antilysin* or aprotimbin or apronitin* or aprotinin* or bayer a128 or contrical or contrycal or contrykal or dilmintal or frey inhibitor or kontrycal or Kunitz inhibitor or gordox or haemoprot or kallikrein‐trypsin inactivator).tw,kf.  53. (iniprol or kontrikal or kontrykal or kunitz pancreatic trypsin inhibitor or midran or pulmin or tracylol or trascolan or trasilol or tra?ylol or traskolan or zymofren or pancreas antitrypsin or protinin or riker 52g or Rivilina zymofren).tw,kf.  54. or/51‐53  55. Factor VIIa/  56. (factor viia or factor 7a or rfviia or fviia or novoseven* or novo seven* or aryoseven or acset or eptacog* or proconvertin).tw,kf.  57. ((activated adj2 factor seven) or (activated adj2 factor vii) or (activated adj3 rfvii) or (activated adj2 fvii)).tw,kf.  58. (factor seven or factor vii or factor 7).ti.  59. 55 or 56 or 57 or 58  60. Fibrinogen/ad, ae, de, sd, tu, th, to  61. *Fibrinogen/  62. (fibrinogen concentrate* or factor I or Haemocomplettan* or Riastap* or Fibryga* or Fibryna*).tw,kf.  63. 60 or 61 or 62  64. Deamino Arginine Vasopressin/  65. (desmopressin* or vasopressin deamino or D‐amino D‐arginine vasopressin or deamino‐8‐d‐arginine vasopressin or vasopressin 1‐desamino‐8‐arginine or desmotabs or DDAVP or desmogalen or adin or adiuretin or concentraid or d‐void or dav ritter or deamino 8 dextro arginine vasopressin or deamino 8d arginine vasopressin or deamino dextro arginine vasopressin or deaminovasopressin or defirin or defirin melt or desmirin or desmomelt or desmopresina or desmospray or desmotab* or desurin or emosint or enupresol or minirin or minirinette or minirinmelt or minrin or minurin or miram or nictur or noctisson or nocturin or nocutil or nordurine or novidin or nucotil or octim or octostim or presinex or stimate or wetirin).tw,kf.  66. 64 or 65  67. exp Factor XIII/  68. (factor XIII or fXIII or fibrin stabili?ing factor* or Tretten* or Catridecacog).tw,kf.  69. 67 or 68  70. exp Tissue Adhesives/  71. *Adhesives/  72. Collagen/tu  73. Thrombin/tu  74. Gelatin/tu  75. Gelatin Sponge, Absorbable/  76. ((fibrin* or collagen or cellulose or gelatin or gel or thrombin* or albumin or hemostatic* or haemostatic*) adj3 (glu* or seal* or adhesive* or topical* or local* or matrix or matrices or spong* or fleece* or foam* or scaffold* or patch* or sheet* or bandag* or aerosol* or dressing* or paste or powder*)).tw,kf.  77. ((nonfibrin* or non‐fibrin* or synthetic* or non‐biological* or nonbiological* or biological*) adj3 (glue* or seal* or adhesive*)).tw,kf.  78. (surgical* adj3 (glue* or sealant* or adhesive*)).tw,kf.  79. ((fibrin* or collagen or cellulose or gelatin or thrombin) adj3 (hemosta* or haemosta*)).tw,kf. 80. (8Y or Aafact or Actif‐VIII or Advate or Artiss or Bioglue or Biocol or Collaseal or Omrixil or Transglutine or Raplixa or Evarrest or Aleviate or Alphanate or Amofil or Beriate or Beriplast or Biostate or Bolheal or Cluvot or Conco‐Eight‐HT or Crosseel or Crosseal or Crosseight or Emoclot or Evarrest or Evicel or Factane or Fanhdi or Fibrogammin P or Green VIII or Green VIII Factor or Greengene or Greenmono or Greenplast or Haemate or Haemate P or Haemate P or Haemate P500 or Haemate‐P or Haemoctin or Haemoctin SDH or Haemoctin‐SDH or Hemaseel or Hemaseal or Hemofil M or Hemoraas or Humaclot or Humafactor‐8 or Humate‐P or Immunate or Innovate or Koate or Koate‐DVI or Kogenate Bayer or Kogenate FS or Monoclate‐P or NovoThirteen or Octafil or Octanate or Octanate or Optivate or Quixil or Talate or Tisseel or Tisseal or Tissel or Tissucol or Tricos or Vivostat or Voncento or Wilate or Wilnativ or Wilstart or Xyntha).tw,kf.  81. (Glubran or Gluetiss or Ifabond or Indermil or LiquiBand or TissuGlu).tw,kf.  82. (Evithrom or Floseal or Hemopatch or Gel‐Flow or Gelfoam or Gelfilm or Recothrom or Surgifoam or Surgiflo* or "rh Thrombin" or Thrombi‐Gel or Thrombi‐Pad or Thrombin‐JMI or Thrombinar or Thrombogen or Thrombostat).tw,kf.  83. (porcine gelatin or bovine collagen or bovine gelatin or nu‐knit or arista or hemostase or vita sure or thrombin‐jmi or thrombinjmi or avicel or vivagel or lyostypt or tabotamp or arterx or omnex or veriset).tw,kf. 84. (polysaccharide adj (sphere* or hemostatic powder)).tw,kf.  85. *Chitosan/  86. *Polyethylene Glycols/  87. *Hydrogel, Polyethylene Glycol Dimethacrylate/  88. Polyurethanes/ad, ae, pd, tu, to  89. ((polymer‐derived elastic* or polymer tissue adhesive* or elastic hydrogel* or glutaraldehyde or PEG‐based or polyurethane‐based tissue or polyethylene glycol* or polyvinyl alcohol‐based tissue or PVA‐based tissue or natural biopolymer* or polypeptide‐based or protein‐based or polysaccharide‐based or chitosan or poliglusam or cyanoacrylic or cyanoacrylate or cyacrin or dextran‐based or chondroitin sulfate‐based or mussel‐inspired elastic* or glycol hydrogel or polymer‐based) adj3 (glu* or seal* or adhesive* or topical* or local* or matrix or matrices or spong* or fleece* or foam* or scaffold* or patch* or sheet* or bandag* or aerosol* or dressing* or paste* or powder*)).tw,kf.  90. Cellulose, Oxidized/  91. (absorbable cellulose or resorbable cellulose or oxidi?ed cellulose or carboxycellulose or oxycellulose or cellulosic acid or oxycel or oxidi?ed regenerated cellulose).tw,kf.  92. (BioGlue or Progel or Duraseal or Coseal or FocalSeal or ADAL‐1 or AdvaSeal or Pleuraseal or Angio‐Seal or Avitene or Instat or Helitene or Helistat or TDM‐621 or Dermabond or Tissueseal or PolyStat or Raplixa or Spongostan or Surgicel or Surgilux or Tachosil or Traumstem).tw,kf.  93. (collagen‐thrombin or thrombin‐collagen or gelatin‐fibrinogen or fibrinogen‐gelatin or gelatin‐thrombin or thrombin‐gelatin or fibrinogen‐thrombin or thrombin‐fibrinogen or collagen‐fibrinogen or fibrinogen‐collagen or microfibrillar collagen or CoStasis or "GRF Glue" or GR‐Dial or Algosterile or TraumaStat or HemCon or ChitoFlex or Celox or QuikClot or WoundStat or Vitagel or TachSeal or TachoComb or Cryoseal).tw,kf. 94. or/70‐93  95. exp Waxes/  96. (bonewax* or bone wax* or bone putty or hemasorb or ostene).tw,kf.  97. 95 or 96  98. Blood Coagulation Factors/  99. (prothrombin adj5 (complex* or concentrate*)).tw,kf.  100. (PCC* or 3F‐PCC* or 4F‐PCC* or Beriplex* or Feiba* or Autoplex* or Ocplex* or Octaplex* or Kcentra* or Cofact or Prothrombinex* or "Proplex‐T" or Prothroraas* or Haemosolvex* or Prothromplex* or "HT Defix" or Facnyne* or Kaskadil* or Kedcom* or Confidex* or PPSB or Profil?ine* or Pronativ* or Proplex* or Prothar* or ProthoRAAS* or Protromplex* or "Pushu Laishi" or "Uman Complex").tw,kf.  101. or/98‐100  102. (((haemosta* or hemosta* or antihaemorrhag* or antihemorrhag* or anti haemorrhag* or anti‐hemorrhag*) adj5 (drug* or agent* or treat* or therap*)) or ((coagulat* or clotting) adj factor*)).tw,kf.  103. 50 or 54 or 59 or 63 or 66 or 69 or 94 or 97 or 101 or 102  104. 43 and 103  105. Meta‐Analysis.pt.  106. Systematic Review.pt.  107. ((meta analy* or metaanaly*) and (trials or studies)).ab.  108. (meta analy* or metaanaly* or evidence‐based).ti.  109. ((systematic* or evidence‐based) adj2 (review* or overview*)).tw,kf.  110. (evidence synthes* or cochrane or medline or pubmed or embase or cinahl or cinhal or lilacs or "web of science" or science citation index or scopus or search terms or literature search or electronic search* or comprehensive search* or systematic search* or published articles or search strateg* or reference list* or bibliograph* or handsearch* or hand search* or manual* search*).ab.  111. Cochrane Database of systematic reviews.jn.  112. (additional adj (papers or articles or sources)).ab.  113. ((electronic* or online) adj (sources or resources or databases)).ab.  114. (relevant adj (journals or articles)).ab.  115. or/105‐114  116. Review.pt.  117. exp Randomized Controlled Trials as Topic/  118. selection criteria.ab. or critical appraisal.tw.  119. (data adj (abstract* or extract* or analys*)).ab.  120. exp Randomized Controlled Trial/  121. or/117‐120  122. 116 and 121  123. 115 or 122  124. Randomized Controlled Trial.pt.  125. Controlled Clinical Trial.pt.  126. (placebo or randomly or groups).ab.  127. (randomi* or trial).tw,kf.  128. exp Clinical Trial as Topic/  129. or/123‐128  130. exp animals/ not humans/  131. 129 not 130  132. 104 and 131

#### Embase (Ovid)

1. artery surgery/ or arteriotomy/ or exp artery anastomosis/ or exp artery ligation/ or artery reconstruction/ or exp atherectomy/ or endarterectomy/  2. vascular surgery/ or exp aneurysm surgery/  3. angioplasty/ or bare metal stenting/ or blunt microdissection/ or exp laser angioplasty/ or patch angioplasty/ or percutaneous transluminal angioplasty/ or transluminal coronary angioplasty/  4. exp blood vessel shunt/  5. exp blood vessel transplantation/  6. devascularization/ or exp embolectomy/ or limb salvage/ or microvascular surgery/  7. exp endovascular surgery/ or exp thrombectomy/  8. exp vein surgery/  9. exp aortic surgery/  10. exp vascular disease/su  11. exp leg amputation/  12. foot amputation/  13. exp thoracic artery/su  14. exp leg artery/su  15. exp artery disease/su  16. or/1‐15  17. interventional radiology/  18. (interventional radiolog* or surgical radiolog*).tw.  19. ((vascular or vessel* or aneurysm or aortic or aorta or AAA or TAA or TAAA or aortofemoral or artery or arterial or interarterial or arterioarterial or atheroma or carotid or vein or venous or endovascular* or intravascular*) adj6 (surg* or operat* or reconstruct* or repair* or resection* or repair* or bypass* or graft* or homograft* or transplant* or bypass* or transpos* or implant* or reimplant* or clamp* or ligat* or crossover* or cross‐over*)).tw.  20. ((endovascular or intravascular or laparoscop* or angioscop*) adj3 (surg* or operat* or reconstruct* or repair* or resection* or repair* or bypass* or graft* or homograft* or transplant* or bypass* or transpos* or implant* or reimplant* or procedure*)).tw.  21. (bypass graft* or by‐pass graft* or bypass surgery or by‐pass surgery or enarterectom* or (minimally invasive adj1 surg*) or teflon graft* or dacron graft*).tw.  22. (arterial dilation* or endoluminal repair*).tw.  23. ((venous or arterial) adj3 catheteri?ation).tw.  24. ((aort* or iliac or femoral or popliteal or femoropop* or fempop* or crural) adj3 (surg* or operat* or bypass or graft or reconstruct* or revascular*)).tw.  25. (angiosurg* or aneurysmectom* or aneurysm clipping* or aortopexy or aortoplast* or arteriotom* or arterioplast* or artery plast* or artery stripping or venostom* or portacaval anastomoses or revasculari?ation or devasculari?ation).tw.  26. ((femoral* or iliac* or aorta* or aortic* or infrapopliteal or popliteal or infra‐pop*) adj3 (stent* or angioplast*)).tw.  27. (endovascular adj6 (dissection* or stent*)).tw.  28. (EVAR or FEVAR or TEVAR or embolectom* or thrombectom* or endarterectom* or thromboendarterectom* or thromboembolectom* or atherectom*).tw.  29. (mechanical* adj3 (thrombolysis or clot disruption*)).tw.  30. (axillo bifemoral bypass graft* or axillo femoral bypass graft* or axillobifemoral bypass graft* or axillofemoral bypass graft*).tw.  31. ((ilio femor* or ilio femor* or infrarenal or infra‐renal or infrainguinal or infra‐inguinal) adj (bypass* or by‐pass*)).tw.  32. ((pedal or tibial) adj3 (angio* or bypass* or by‐pass*)).tw.  33. ((lower limb* or knee* or leg* or foot or feet or lower extremit* or hindquarter*) adj5 (salvag* or saving or save* or angioplast* or graft* or bypass* or by‐pass* or revascula* or reconstruct* or amputat*)).tw. 34. ((blood vessel* or vascular or endovascular or vein or venous or arterial or artery or arteriovenous or arterio‐venous or brescia cimino or venoarterial or veno‐arterial or arterioportal) adj3 (anastomosis or shunt*)).tw.  35. or/17‐34  36. 16 or 35  37. Antifibrinolytic Agent/  38. Tranexamic Acid/  39. Aminocaproic Acid/  40. (antifibrinolytic* or anti‐fibrinolytic* or antifibrinolysin* or antiplasmin* or plasmin inhibitor* or tranexamic or tranhexamic or cyclohexanecarboxylic acid* or amcha or trans‐4‐aminomethyl‐cyclohexanecarboxylic acid* or t‐amcha or amca or "kabi 2161" or transamin or amchafibrin or anvitoff or spotof or cyklokapron or cyclo‐F or femstrual or ugurol or aminomethylcyclohexanecarbonic acid or aminomethylcyclohexanecarboxylic acid or AMCHA or amchafibrin or amikapron or aminomethyl cyclohexane carboxylic acid or aminomethyl cyclohexanecarboxylic acid or aminomethylcyclohexane carbonic acid or aminomethylcyclohexane carboxylic acid or aminomethylcyclohexanecarbonic acid or aminomethylcyclohexanecarboxylic acid or aminomethylcyclohexanocarboxylic acid or aminomethylcyclohexanoic acid or amstat or antivoff or caprilon or cl?65336 or cl65336 or cyclocapron or cyclokapron or cyklocapron or cyklokapron or exacyl or frenolyse or fibrinon or hemostan or hexacapron or hexakapron or kalnex or lysteda or rikaparin or ronex or theranex or tranexam or tranexanic or tranexic or trans achma or transexamic or trenaxin or TXA or (fibrinolysis adj2 inhibitor*)).tw.  41. (Agretax or Bio‐Stat or Capiloc or Capitrax or Clip Inj or Clot‐XL or Clotawin‐T or Coastat or Cuti or Cymin or Dubatran or Espercil or Examic or Existat or Extam or Fibran or Gynae‐Pil or Hemstate or Kapron or Menogia or Monitex or Nestran or Nexamic or Nexi‐500 or Nexmeff or Nicolda or Nixa‐500 or Pause or Rheonex or Sylstep TX or Synostat or T‐nex or T Stat or T Stat or Tanmic or Temsyl‐T or Texakind or Texanis or Texapar or Texid or Thams or Tonopan or Traklot or Tramic or Tramix or Tranarest or Trance Inj or Tranecid or Tranee or Tranemic or Tranex or Tranexa or Tranfib or Tranlok or Transtat or Transys or Transcam or Tranxi or Trapic or Traxage or Traxamic or Traxyl or Trenaxa or Trexamic or Trim Inj or Tx‐1000 or Tx 500 or Wistran or X‐Tran or Xamic).tw.  42. (6‐aminohexanoic or amino?caproic or amino?hexanoic or amino caproic or amino‐caproic or amino‐n‐hexanoic or cy‐116 or cy116 or lederle or acikaprin or afibrin or amicar or caprocid or capracid or capramol or caprogel or caprolest or caprolisin* or caprolysin* or capromol or epsikapron or hemocaprol or caproamin or EACA or caprolest or capralense or hexalense or hamostat or hemocid or cl 10304 or cl10304 or ecapron or ekaprol or epsamon or epsicaprom or epsicapron or epsilcapramin or epsilon amino caproate or epsilon aminocaproate or epsilonaminocaproic or epsilonaminocapronsav or etha?aminocaproic or ethaaminocaproich or emocaprol or hepin or ipsilon or jd?177or neocaprol or nsc?26154 or resplamin or tachostyptan).tw. 43. or/37‐42  44. Aprotinin/  45. (antagosan or antilysin* or aprotimbin or apronitin* or aprotinin* or bayer a128 or contrical or contrycal or contrykal or dilmintal or frey inhibitor or kontrycal or Kunitz inhibitor or gordox or haemoprot or kallikrein‐trypsin inactivator).tw.  46. (iniprol or kontrikal or kontrykal or kunitz pancreatic trypsin inhibitor or midran or pulmin or tracylol or trascolan or trasilol or tra?ylol or traskolan or zymofren or pancreas antitrypsin or protinin or riker 52g or Rivilina zymofren).tw.  47. or/44‐46  48. Blood Clotting Factor 7a/  49. (factor viia or factor 7a or rfviia or fviia or novoseven* or novo seven* or aryoseven or acset or eptacog* or proconvertin).tw.  50. ((activated adj2 factor seven) or (activated adj2 factor vii) or (activated adj3 rfvii) or (activated adj2 fvii)).tw.  51. (factor seven or factor vii or factor 7).ti.  52. 48 or 49 or 50 or 51  53. Fibrinogen Concentrate/  54. (fibrinogen concentrate* or factor I or Haemocomplettan* or Riastap* or Fibryga* or Fibryna*).tw.  55. 53 or 54  56. Desmopressin/  57. (desmopressin* or vasopressin deamino or D‐amino D‐arginine vasopressin or deamino‐8‐d‐arginine vasopressin or vasopressin 1‐desamino‐8‐arginine or desmotabs or DDAVP or desmogalen or adin or adiuretin or concentraid or d‐void or dav ritter or deamino 8 dextro arginine vasopressin or deamino 8d arginine vasopressin or deamino dextro arginine vasopressin or deaminovasopressin or defirin or defirin melt or desmirin or desmomelt or desmopresina or desmospray or desmotab* or desurin or emosint or enupresol or minirin or minirinette or minirinmelt or minrin or minurin or miram or nictur or noctisson or nocturin or nocutil or nordurine or novidin or nucotil or octim or octostim or presinex or stimate or wetirin).tw.  58. 56 or 57  59. Blood Clotting Factor 13/  60. (factor xiii or fxiii or fibrin stabili?ing factor* or Tretten* or Catridecacog).tw.  61. 59 or 60  62. exp Tissue Adhesive/  63. *Adhesive Agent/  64. *Hemostatic Agent/  65. ((fibrin* or collagen or cellulose or gelatin or gel or thrombin* or albumin or hemostatic* or haemostatic*) adj3 (glu* or seal* or adhesive* or topical* or local* or matrix or matrices or spong* or fleece* or foam* or scaffold* or patch* or sheet* or bandag* or aerosol* or dressing* or paste or powder*)).tw.  66. ((nonfibrin* or non‐fibrin* or synthetic* or non‐biological* or nonbiological* or biological*) adj3 (glue* or seal* or adhesive*)).tw.  67. (surgical* adj3 (glue* or sealant* or adhesive*)).tw.  68. ((fibrin* or collagen or cellulose or gelatin or thrombin) adj3 (hemosta* or haemosta*)).tw.  69. (8Y or Aafact or Actif‐VIII or Advate or Artiss or Raplixa or Evarrest or Aleviate or Alphanate or Amofil or Beriate or Beriplast or Biostate or Bolheal or Cluvot or Conco‐Eight‐HT or Crosseel or Crosseal or Crosseight or Emoclot or Evarrest or Evicel or Factane or Fanhdi or Fibrogammin P or Green VIII or Green VIII Factor or Greengene or Greenmono or Greenplast or Haemate or Haemate P or Haemate P or Haemate P500 or Haemate‐P or Haemoctin or Haemoctin SDH or Haemoctin‐SDH or Hemaseel or Hemaseal or Hemofil M or Hemoraas or Humaclot or Humafactor‐8 or Humate‐P or Immunate or Innovate or Koate or Koate‐DVI or Kogenate Bayer or Kogenate FS or Monoclate‐P or NovoThirteen or Octafil or Octanate or Octanate or Optivate or Quixil or Talate or Tisseel or Tisseal or Tissel or Tissucol or Tricos or Vivostat or Voncento or Wilate or Wilnativ or Wilstart or Xyntha).tw. 70. (Glubran or Gluetiss or Ifabond or Indermil or LiquiBand or TissuGlu).tw.  71. Collagen Sponge/ or Collagen Dressing/  72. Gelatin Sponge/ or Gelfoam/  73. (Evithrom or Floseal or Hemopatch or Gel‐Flow or Gelfoam or Gelfilm or Recothrom or Surgifoam or Surgiflo* or "rh Thrombin" or Thrombi‐Gel or Thrombi‐Pad or Thrombin‐JMI or Thrombinar or Thrombogen or Thrombostat).tw.  74. *Chitosan/  75. Hydrogel Dressing/  76. Fibrinogen plus Thrombin/  77. Polyvinyl Alcohol Sponge/  78. (porcine gelatin or bovine collagen or bovine gelatin or nu‐knit or arista or hemostase or vita sure or thrombin‐jmi or thrombinjmi or avicel or vivagel or lyostypt or tabotamp or arterx or omnex or veriset).tw. 79. (polysaccharide adj (sphere* or hemostatic powder)).tw.  80. ((polymer‐derived elastic* or polymer tissue adhesive* or elastic hydrogel* or glutaraldehyde or PEG‐based or polyurethane‐based tissue or polyethylene glycol* or polyvinyl alcohol‐based tissue or PVA‐based tissue or natural biopolymer* or polypeptide‐based or protein‐based or polysaccharide‐based or chitosan or poliglusam or cyanoacrylic or cyanoacrylate or cyacrin or dextran‐based or chondroitin sulfate‐based or mussel‐inspired elastic* or glycol hydrogel or polymer‐based) adj3 (glu* or seal* or adhesive* or topical* or local* or matrix or matrices or spong* or fleece* or foam* or scaffold* or patch* or sheet* or bandag* or aerosol* or dressing* or paste* or powder*)).tw.  81. Oxidized Cellulose/  82. Oxidized Regenerated Cellulose/  83. Recombinant Thrombin/  84. Tachocomb/  85. (absorbable cellulose or resorbable cellulose or oxidi?ed cellulose or carboxycellulose or oxycellulose or cellulosic acid or oxycel or oxidi?ed regenerated cellulose).tw.  86. (BioGlue or Progel or Duraseal or Coseal or FocalSeal or ADAL‐1 or AdvaSeal or Pleuraseal or Angio‐Seal or Avitene or Instat or Helitene or Helistat or TDM‐621 or Dermabond or Tissueseal or PolyStat or Raplixa or Spongostan or Surgicel).tw.  87. (Tachosil or Traumstem or CoStasis or "GRF Glue" or GR‐Dial or Algosterile or TraumaStat or HemCon or ChitoFlex or Celox or QuikClot or WoundStat or Vitagel or TachSeal or TachoComb or Cryoseal).tw.  88. (collagen‐thrombin or thrombin‐collagen or gelatin‐fibrinogen or fibrinogen‐gelatin or gelatin‐thrombin or thrombin‐gelatin or fibrinogen‐thrombin or thrombin‐fibrinogen or collagen‐fibrinogen or fibrinogen‐collagen or microfibrillar collagen).tw.  89. or/62‐88  90. Bone Wax/  91. (bonewax* or bone wax* or bone putty or hemasorb or ostene).tw.  92. or/90‐91  93. prothrombin complex/  94. (prothrombin adj5 (complex* or concentrate*)).tw.  95. (PCC* or 3F‐PCC* or 4F‐PCC* or Beriplex* or Feiba* or Autoplex* or Ocplex* or Octaplex* or Kcentra* or Cofact or Prothrombinex* or "Proplex‐T" or Prothroraas* or Haemosolvex* or Prothromplex* or "HT Defix" or Facnyne* or Kaskadil* or Kedcom* or Confidex* or PPSB or Profil?ine* or Pronativ* or Proplex* or Prothar* or ProthoRAAS* or Protromplex* or "Pushu Laishi" or "Uman Complex").tw.  96. or/93‐95  97. (((haemosta* or hemosta* or antihaemorrhag* or antihemorrhag* or anti haemorrhag* or anti‐hemorrhag*) adj5 (drug* or agent* or treat* or therap*)) or ((coagulat* or clotting) adj factor*)).tw. 98. 43 or 47 or 52 or 55 or 58 or 61 or 89 or 92 or 96 or 97  99. meta analysis/  100. (meta analy* or metaanaly* or evidence‐based).ti.  101. ((meta analy* or metaanaly*) and (trials or studies)).ab.  102. systematic review/  103. ((systematic* or evidence‐based) adj2 (review* or overview*)).tw.  104. (evidence synthes* or cochrane or medline or pubmed or embase or cinahl or cinhal or lilacs or "web of science" or science citation index or scopus or search terms or literature search or electronic search* or comprehensive search* or systematic search* or published articles or search strateg* or reference list* or bibliograph* or handsearch* or hand search* or manual* search*).ab.  105. ((electronic* or online) adj (sources or resources or databases)).ab.  106. ((additional adj (papers or articles or sources)) or (relevant adj (journals or articles))).ab.  107. or/99‐106  108. Review.pt.  109. (data extraction or selection criteria).ab.  110. 108 and 109  111. 107 or 110  112. Editorial.pt.  113. 111 not 112  114. crossover‐procedure/ or double‐blind procedure/ or randomized controlled trial/ or single‐blind procedure/  115. (random* or factorial* or crossover* or cross over* or cross‐over* or placebo* or doubl* blind* or singl* blind* or assign* or allocat* or volunteer*).mp.  116. 113 or 114 or 115  117. (exp animal/ or nonhuman/) not exp human/  118. 116 not 117  119. 36 and 98 and 118

#### CINAHL (EBSCOhost)

S1 (MH "Vascular Surgery+") S2 (MH "Graft Occlusion, Vascular") S3 (MH "Limb Salvage") S4 (MH "Amputation+") S5 (MH "Vascular Diseases+/SU") S6 (MH "Femoral Artery") OR (MH "Iliac Artery") OR (MH "Popliteal Artery/SU") S7 TX (interventional radiolog* or surgical radiolog*) S8 TI ( ((vascular or vessel* or aneurysm or aortic or aorta or AAA or TAA or TAAA or aortofemoral or artery or arterial or interarterial or arterioarterial or atheroma or carotid or vein or venous or endovascular* or intravascular*) N6 (surg* or operat* or reconstruct* or repair* or resection* or repair* or bypass* or graft* or homograft* or transplant* or bypass* or transpos* or implant* or reimplant* or clamp* or ligat* or crossover* or cross‐over*)) ) OR AB ( ((vascular or vessel* or aneurysm or aortic or aorta or AAA or TAA or TAAA or aortofemoral or artery or arterial or interarterial or arterioarterial or atheroma or carotid or vein or venous or endovascular* or intravascular*) N6 (surg* or operat* or reconstruct* or repair* or resection* or repair* or bypass* or graft* or homograft* or transplant* or bypass* or transpos* or implant* or reimplant* or clamp* or ligat* or crossover* or cross‐over*)) ) S9 TI ( ((endovascular or intravascular or laparoscop* or angioscop*) N3 (surg* or operat* or reconstruct* or repair* or resection* or repair* or bypass* or graft* or homograft* or transplant* or bypass* or transpos* or implant* or reimplant* or procedure*)) ) OR AB ( ((endovascular or intravascular or laparoscop* or angioscop*) N3 (surg* or operat* or reconstruct* or repair* or resection* or repair* or bypass* or graft* or homograft* or transplant* or bypass* or transpos* or implant* or reimplant* or procedure*)) ) S10 TI ( (bypass graft* or by‐pass graft* or bypass surgery or by‐pass surgery or enarterectom* or (minimally invasive N1 surg*) or teflon graft* or dacron graft*) ) OR AB ( (bypass graft* or by‐pass graft* or bypass surgery or by‐pass surgery or enarterectom* or (minimally invasive N1 surg*) or teflon graft* or dacron graft*) ) S11 TI ( (arterial dilation* or endoluminal repair*) ) OR AB ( (arterial dilation* or endoluminal repair*) ) S12 TI ( ((venous or arterial) N3 catheteri?ation) ) OR AB ( ((venous or arterial) N3 catheteri?ation) ) S13 TI ( ((aort* or iliac or femoral or popliteal or femoropop* or fempop* or crural) N3 (surg* or operat* or bypass or graft or reconstruct* or revascular*)) ) AND AB ( ((aort* or iliac or femoral or popliteal or femoropop* or fempop* or crural) N3 (surg* or operat* or bypass or graft or reconstruct* or revascular*)) ) S14 TI ( (angiosurg* or aneurysmectom* or aneurysm clipping* or aortopexy or aortoplast* or arteriotom* or arterioplast* or artery plast* or artery stripping or venostom* or portacaval anastomoses or revasculari?ation or devasculari?ation) ) OR AB ( (angiosurg* or aneurysmectom* or aneurysm clipping* or aortopexy or aortoplast* or arteriotom* or arterioplast* or artery plast* or artery stripping or venostom* or portacaval anastomoses or revasculari?ation or devasculari?ation) ) S15 TI ( ((femoral* or iliac* or aorta* or aortic* or infrapopliteal or popliteal or infra‐pop*) N3 (stent* or angioplast*)) ) OR AB ( ((femoral* or iliac* or aorta* or aortic* or infrapopliteal or popliteal or infra‐pop*) N3 (stent* or angioplast*)) ) S16 TI ( (endovascular N6 (dissection* or stent*)) ) OR AB ( (endovascular N6 (dissection* or stent*)) ) S17 TI ( (EVAR or FEVAR or TEVAR or embolectom* or thrombectom* or endarterectom* or thromboendarterectom* or thromboembolectom* or atherectom*) ) OR AB ( (EVAR or FEVAR or TEVAR or embolectom* or thrombectom* or endarterectom* or thromboendarterectom* or thromboembolectom* or atherectom*) ) S18 TI ( (mechanical* N3 (thrombolysis or clot disruption*)) ) OR AB ( (mechanical* N3 (thrombolysis or clot disruption*)) ) S19 TI ( (axillo bifemoral bypass graft* or axillo femoral bypass graft* or axillobifemoral bypass graft* or axillofemoral bypass graft*) ) OR AB ( (axillo bifemoral bypass graft* or axillo femoral bypass graft* or axillobifemoral bypass graft* or axillofemoral bypass graft*) ) S20 TI ( ((ilio femor* or ilio femor* or infrarenal or infra‐renal or infrainguinal or infra‐inguinal) NEXT (bypass* or by‐pass*)) ) OR AB ( ((ilio femor* or ilio femor* or infrarenal or infra‐renal or infrainguinal or infra‐inguinal) NEXT (bypass* or by‐pass*)) ) S21 TI ( ((ilio femor* or ilio femor* or infrarenal or infra‐renal or infrainguinal or infra‐inguinal) W1 (bypass* or by‐pass*)) ) OR AB ( ((ilio femor* or ilio femor* or infrarenal or infra‐renal or infrainguinal or infra‐inguinal) W1 (bypass* or by‐pass*)) ) S22 TI ( ((pedal or tibial) N3 (angio* or bypass* or by‐pass*)) ) OR AB ( ((pedal or tibial) N3 (angio* or bypass* or by‐pass*)) ) S23 TI ( ((lower limb* or knee* or leg* or foot or feet or lower extremit* or hindquarter*) N5 (salvag* or saving or save* or angioplast* or graft* or bypass* or by‐pass* or revascula* or reconstruct* or amputat*)) ) OR AB ( ((lower limb* or knee* or leg* or foot or feet or lower extremit* or hindquarter*) N5 (salvag* or saving or save* or angioplast* or graft* or bypass* or by‐pass* or revascula* or reconstruct* or amputat*)) ) S24 TI ( ((blood vessel* or vascular or endovascular or vein or venous or arterial or artery or arteriovenous or arterio‐venous or brescia cimino or venoarterial or veno‐arterial or arterioportal) N3 (anastomosis or shunt*)) ) OR AB ( ((blood vessel* or vascular or endovascular or vein or venous or arterial or artery or arteriovenous or arterio‐venous or brescia cimino or venoarterial or veno‐arterial or arterioportal) N3 (anastomosis or shunt*)) ) S25 S1 OR S2 OR S3 OR S4 OR S5 OR S6 OR S7 OR S8 OR S9 OR S10 OR S11 OR S12 OR S13 OR S14 OR S15 OR S16 OR S17 OR S18 OR S19 OR S20 OR S21 OR S22 OR S23 OR S24 S26 (MH "Antifibrinolytic Agents") OR (MH "Aminocaproic Acids") OR (MH "Tranexamic Acid") S27 TI ( (antifibrinolytic* or anti‐fibrinolytic* or antifibrinolysin* or antiplasmin* or plasmin inhibitor* or tranexamic or tranhexamic or cyclohexanecarboxylic acid* or amcha or trans‐4‐aminomethyl‐cyclohexanecarboxylic acid* or t‐amcha or amca or "kabi 2161" or transamin or amchafibrin or anvitoff or spotof or cyklokapron or cyclo‐F or femstrual or ugurol or aminomethylcyclohexanecarbonic acid or aminomethylcyclohexanecarboxylic acid or AMCHA or amchafibrin or amikapron or aminomethyl cyclohexane carboxylic acid or aminomethyl cyclohexanecarboxylic acid or aminomethylcyclohexane carbonic acid or aminomethylcyclohexane carboxylic acid or aminomethylcyclohexanecarbonic acid or aminomethylcyclohexanecarboxylic acid or aminomethylcyclohexanocarboxylic acid or aminomethylcyclohexanoic acid or amstat or antivoff or caprilon or cl?65336 or cl65336 or cyclocapron or cyclokapron or cyklocapron or cyklokapron or exacyl or frenolyse or fibrinon or hemostan or hexacapron or hexakapron or kalnex or lysteda or rikaparin or ronex or theranex or tranexam or tranexanic or tranexic or trans achma or transexamic or trenaxin or TXA or (fibrinolysis N2 inhibitor*)) ) OR AB ( (antifibrinolytic* or anti‐fibrinolytic* or antifibrinolysin* or antiplasmin* or plasmin inhibitor* or tranexamic or tranhexamic or cyclohexanecarboxylic acid* or amcha or trans‐4‐aminomethyl‐cyclohexanecarboxylic acid* or t‐amcha or amca or "kabi 2161" or transamin or amchafibrin or anvitoff or spotof or cyklokapron or cyclo‐F or femstrual or ugurol or aminomethylcyclohexanecarbonic acid or aminomethylcyclohexanecarboxylic acid or AMCHA or amchafibrin or amikapron or aminomethyl cyclohexane carboxylic acid or aminomethyl cyclohexanecarboxylic acid or aminomethylcyclohexane carbonic acid or aminomethylcyclohexane carboxylic acid or aminomethylcyclohexanecarbonic acid or aminomethylcyclohexanecarboxylic acid or aminomethylcyclohexanocarboxylic acid or aminomethylcyclohexanoic acid or amstat or antivoff or caprilon or cl?65336 or cl65336 or cyclocapron or cyclokapron or cyklocapron or cyklokapron or exacyl or frenolyse or fibrinon or hemostan or hexacapron or hexakapron or kalnex or lysteda or rikaparin or ronex or theranex or tranexam or tranexanic or tranexic or trans achma or transexamic or trenaxin or TXA or (fibrinolysis N2 inhibitor*)) ) S28 TI ( (6‐aminohexanoic or amino?caproic or amino?hexanoic or amino caproic or amino‐caproic or amino‐n‐hexanoic or cy‐116 or cy116 or lederle or acikaprin or afibrin or amicar or caprocid or capracid or capramol or caprogel or caprolest or caprolisin* or caprolysin* or capromol or epsikapron or hemocaprol or caproamin or EACA or caprolest or capralense or hexalense or hamostat or hemocid or cl 10304 or cl10304 or ecapron or ekaprol or epsamon or epsicaprom or epsicapron or epsilcapramin or epsilon amino caproate or epsilon aminocaproate or epsilonaminocaproic or epsilonaminocapronsav or etha?aminocaproic or ethaaminocaproich or emocaprol or hepin or ipsilon or jd?177or neocaprol or nsc?26154 or resplamin or tachostyptan) ) OR AB ( (6‐aminohexanoic or amino?caproic or amino?hexanoic or amino caproic or amino‐caproic or amino‐n‐hexanoic or cy‐116 or cy116 or lederle or acikaprin or afibrin or amicar or caprocid or capracid or capramol or caprogel or caprolest or caprolisin* or caprolysin* or capromol or epsikapron or hemocaprol or caproamin or EACA or caprolest or capralense or hexalense or hamostat or hemocid or cl 10304 or cl10304 or ecapron or ekaprol or epsamon or epsicaprom or epsicapron or epsilcapramin or epsilon amino caproate or epsilon aminocaproate or epsilonaminocaproic or epsilonaminocapronsav or etha?aminocaproic or ethaaminocaproich or emocaprol or hepin or ipsilon or jd?177or neocaprol or nsc?26154 or resplamin or tachostyptan) ) S29 S26 OR S27 OR S28 S30 (MH "Aprotinin") S31 TI ( (antagosan or antilysin* or aprotimbin or apronitin* or aprotinin* or bayer a128 or contrical or contrycal or contrykal or dilmintal or frey inhibitor or kontrycal or Kunitz inhibitor or gordox or haemoprot or kallikrein‐trypsin inactivator) ) OR AB ( (antagosan or antilysin* or aprotimbin or apronitin* or aprotinin* or bayer a128 or contrical or contrycal or contrykal or dilmintal or frey inhibitor or kontrycal or Kunitz inhibitor or gordox or haemoprot or kallikrein‐trypsin inactivator) ) S32 TI ( (iniprol or kontrikal or kontrykal or kunitz pancreatic trypsin inhibitor or midran or pulmin or tracylol or trascolan or trasilol or tra?ylol or traskolan or zymofren or pancreas antitrypsin or protinin or riker 52g or Rivilina zymofren) ) OR AB ( (iniprol or kontrikal or kontrykal or kunitz pancreatic trypsin inhibitor or midran or pulmin or tracylol or trascolan or trasilol or tra?ylol or traskolan or zymofren or pancreas antitrypsin or protinin or riker 52g or Rivilina zymofren) ) S33 S30 OR S31 OR S32 S34 TX ( (factor viia or factor 7a or rfviia or fviia or novoseven* or novo seven* or aryoseven or acset or eptacog* or proconvertin) ) OR TX ( ((activated N2 factor seven) or (activated N2 factor vii) or (activated N3 rfvii) or (activated N2 fvii)) ) S35 TX (factor seven or factor vii or factor 7) S36 S34 OR S35 S37 (MH "Fibrinogen") S38 TX (fibrinogen concentrate* or factor I or Haemocomplettan* or Riastap* or Fibryga* or Fibryna*) S39 S37 OR S38 S40 (MH "Desmopressin") S41 TI ( (desmopressin* or vasopressin deamino or D‐amino D‐arginine vasopressin or deamino‐8‐d‐arginine vasopressin or vasopressin 1‐desamino‐8‐arginine or desmotabs or DDAVP or desmogalen or adin or adiuretin or concentraid or d‐void or dav ritter or deamino 8 dextro arginine vasopressin or deamino 8d arginine vasopressin or deamino dextro arginine vasopressin or deaminovasopressin or defirin or defirin melt or desmirin or desmomelt or desmopresina or desmospray or desmotab* or desurin or emosint or enupresol or minirin or minirinette or minirinmelt or minrin or minurin or miram or nictur or noctisson or nocturin or nocutil or nordurine or novidin or nucotil or octim or octostim or presinex or stimate or wetirin) ) OR AB ( (desmopressin* or vasopressin deamino or D‐amino D‐arginine vasopressin or deamino‐8‐d‐arginine vasopressin or vasopressin 1‐desamino‐8‐arginine or desmotabs or DDAVP or desmogalen or adin or adiuretin or concentraid or d‐void or dav ritter or deamino 8 dextro arginine vasopressin or deamino 8d arginine vasopressin or deamino dextro arginine vasopressin or deaminovasopressin or defirin or defirin melt or desmirin or desmomelt or desmopresina or desmospray or desmotab* or desurin or emosint or enupresol or minirin or minirinette or minirinmelt or minrin or minurin or miram or nictur or noctisson or nocturin or nocutil or nordurine or novidin or nucotil or octim or octostim or presinex or stimate or wetirin) ) S42 S40 OR S41 S43 TX (factor XIII or fXIII or fibrin stabili?ing factor* or Tretten* or Catridecacog) S44 (MH "Tissue Adhesives") S45 (MH "Fibrin Tissue Adhesive") S46 (MH "Collagen/TU") S47 (MH "Thrombin/TU") S48 (MH "Surgical Sponges") S49 TI ( ((fibrin* or collagen or cellulose or gelatin or gel or thrombin* or albumin or hemostatic* or haemostatic*) N3 (glu* or seal* or adhesive* or topical* or local* or matrix or matrices or spong* or fleece* or foam* or scaffold* or patch* or sheet* or bandag* or aerosol* or dressing* or paste or powder*)) ) OR AB ( ((fibrin* or collagen or cellulose or gelatin or gel or thrombin* or albumin or hemostatic* or haemostatic*) N3 (glu* or seal* or adhesive* or topical* or local* or matrix or matrices or spong* or fleece* or foam* or scaffold* or patch* or sheet* or bandag* or aerosol* or dressing* or paste or powder*)) ) S50 TI ( ((nonfibrin* or non‐fibrin* or synthetic* or non‐biological* or nonbiological* or biological*) N3 (glue* or seal* or adhesive*)) ) OR AB ( ((nonfibrin* or non‐fibrin* or synthetic* or non‐biological* or nonbiological* or biological*) N3 (glue* or seal* or adhesive*)) ) S51 TI ( (surgical* N3 (glue* or sealant* or adhesive*)) ) OR AB ( (surgical* N3 (glue* or sealant* or adhesive*)) ) S52 TI ( ((fibrin* or collagen or cellulose or gelatin or thrombin) N3 (hemosta* or haemosta*)) ) OR AB ( ((fibrin* or collagen or cellulose or gelatin or thrombin) N3 (hemosta* or haemosta*)) ) S53 TI ( (8Y or Aafact or Actif‐VIII or Advate or Artiss or Bioglue or Biocol or Collaseal or Omrixil or Transglutine or Raplixa or Evarrest or Aleviate or Alphanate or Amofil or Beriate or Beriplast or Biostate or Bolheal or Cluvot or Conco‐Eight‐HT or Crosseel or Crosseal or Crosseight or Emoclot or Evarrest or Evicel or Factane or Fanhdi or Fibrogammin P or Green VIII or Green VIII Factor or Greengene or Greenmono or Greenplast or Haemate or Haemate P or Haemate P or Haemate P500 or Haemate‐P or Haemoctin or Haemoctin SDH or Haemoctin‐SDH or Hemaseel or Hemaseal or Hemofil M or Hemoraas or Humaclot or Humafactor‐8 or Humate‐P or Immunate or Innovate or Koate or Koate‐DVI or Kogenate Bayer or Kogenate FS or Monoclate‐P or NovoThirteen or Octafil or Octanate or Octanate or Optivate or Quixil or Talate or Tisseel or Tisseal or Tissel or Tissucol or Tricos or Vivostat or Voncento or Wilate or Wilnativ or Wilstart or Xyntha) ) OR AB ( (8Y or Aafact or Actif‐VIII or Advate or Artiss or Bioglue or Biocol or Collaseal or Omrixil or Transglutine or Raplixa or Evarrest or Aleviate or Alphanate or Amofil or Beriate or Beriplast or Biostate or Bolheal or Cluvot or Conco‐Eight‐HT or Crosseel or Crosseal or Crosseight or Emoclot or Evarrest or Evicel or Factane or Fanhdi or Fibrogammin P or Green VIII or Green VIII Factor or Greengene or Greenmono or Greenplast or Haemate or Haemate P or Haemate P or Haemate P500 or Haemate‐P or Haemoctin or Haemoctin SDH or Haemoctin‐SDH or Hemaseel or Hemaseal or Hemofil M or Hemoraas or Humaclot or Humafactor‐8 or Humate‐P or Immunate or Innovate or Koate or Koate‐DVI or Kogenate Bayer or Kogenate FS or Monoclate‐P or NovoThirteen or Octafil or Octanate or Octanate or Optivate or Quixil or Talate or Tisseel or Tisseal or Tissel or Tissucol or Tricos or Vivostat or Voncento or Wilate or Wilnativ or Wilstart or Xyntha) ) S54 TI ( (Glubran or Gluetiss or Ifabond or Indermil or LiquiBand or TissuGlu) ) OR AB ( (Glubran or Gluetiss or Ifabond or Indermil or LiquiBand or TissuGlu) ) S55 TI ( (Evithrom or Floseal or Hemopatch or Gel‐Flow or Gelfoam or Gelfilm or Recothrom or Surgifoam or Surgiflo* or "rh Thrombin" or Thrombi‐Gel or Thrombi‐Pad or Thrombin‐JMI or Thrombinar or Thrombogen or Thrombostat) ) OR AB ( (Evithrom or Floseal or Hemopatch or Gel‐Flow or Gelfoam or Gelfilm or Recothrom or Surgifoam or Surgiflo* or "rh Thrombin" or Thrombi‐Gel or Thrombi‐Pad or Thrombin‐JMI or Thrombinar or Thrombogen or Thrombostat) ) S56 TI ( (porcine gelatin or bovine collagen or bovine gelatin or nu‐knit or arista or hemostase or vita sure or thrombin‐jmi or thrombinjmi or avicel or vivagel or lyostypt or tabotamp or arterx or omnex or veriset) ) OR AB ( (porcine gelatin or bovine collagen or bovine gelatin or nu‐knit or arista or hemostase or vita sure or thrombin‐jmi or thrombinjmi or avicel or vivagel or lyostypt or tabotamp or arterx or omnex or veriset) ) S57 TX (polysaccharide NEXT (sphere* or hemostatic powder)) S58 (MM "Polyethylene Glycols") S59 (MH "Hydrogel Dressings") S60 (MH "Polyurethanes/AD/AE/TU/ST/DE") S61 TI ( ((polymer‐derived elastic* or polymer tissue adhesive* or elastic hydrogel* or glutaraldehyde or PEG‐based or polyurethane‐based tissue or polyethylene glycol* or polyvinyl alcohol‐based tissue or PVA‐based tissue or natural biopolymer* or polypeptide‐based or protein‐based or polysaccharide‐based or chitosan or poliglusam or cyanoacrylic or cyanoacrylate or cyacrin or dextran‐based or chondroitin sulfate‐based or mussel‐inspired elastic* or glycol hydrogel or polymer‐based) N3 (glu* or seal* or adhesive* or topical* or local* or matrix or matrices or spong* or fleece* or foam* or scaffold* or patch* or sheet* or bandag* or aerosol* or dressing* or paste* or powder*)) ) OR AB ( ((polymer‐derived elastic* or polymer tissue adhesive* or elastic hydrogel* or glutaraldehyde or PEG‐based or polyurethane‐based tissue or polyethylene glycol* or polyvinyl alcohol‐based tissue or PVA‐based tissue or natural biopolymer* or polypeptide‐based or protein‐based or polysaccharide‐based or chitosan or poliglusam or cyanoacrylic or cyanoacrylate or cyacrin or dextran‐based or chondroitin sulfate‐based or mussel‐inspired elastic* or glycol hydrogel or polymer‐based) N3 (glu* or seal* or adhesive* or topical* or local* or matrix or matrices or spong* or fleece* or foam* or scaffold* or patch* or sheet* or bandag* or aerosol* or dressing* or paste* or powder*)) ) S62 (MH "Cellulose/TU") S63 TI ( (absorbable cellulose or resorbable cellulose or oxidi?ed cellulose or carboxycellulose or oxycellulose or cellulosic acid or oxycel or oxidi?ed regenerated cellulose) ) OR AB ( (absorbable cellulose or resorbable cellulose or oxidi?ed cellulose or carboxycellulose or oxycellulose or cellulosic acid or oxycel or oxidi?ed regenerated cellulose) ) S64 TI ( (BioGlue or Progel or Duraseal or Coseal or FocalSeal or ADAL‐1 or AdvaSeal or Pleuraseal or Angio‐Seal or Avitene or Instat or Helitene or Helistat or TDM‐621 or Dermabond or Tissueseal or PolyStat or Raplixa or Spongostan or Surgicel or Surgilux or Tachosil or Traumstem) ) OR AB ( (BioGlue or Progel or Duraseal or Coseal or FocalSeal or ADAL‐1 or AdvaSeal or Pleuraseal or Angio‐Seal or Avitene or Instat or Helitene or Helistat or TDM‐621 or Dermabond or Tissueseal or PolyStat or Raplixa or Spongostan or Surgicel or Surgilux or Tachosil or Traumstem) ) S65 TI ( (collagen‐thrombin or thrombin‐collagen or gelatin‐fibrinogen or fibrinogen‐gelatin or gelatin‐thrombin or thrombin‐gelatin or fibrinogen‐thrombin or thrombin‐fibrinogen or collagen‐fibrinogen or fibrinogen‐collagen or microfibrillar collagen or CoStasis or "GRF Glue" or GR‐Dial or Algosterile or TraumaStat or HemCon or ChitoFlex or Celox or QuikClot or WoundStat or Vitagel or TachSeal or TachoComb or Cryoseal) ) OR AB ( (collagen‐thrombin or thrombin‐collagen or gelatin‐fibrinogen or fibrinogen‐gelatin or gelatin‐thrombin or thrombin‐gelatin or fibrinogen‐thrombin or thrombin‐fibrinogen or collagen‐fibrinogen or fibrinogen‐collagen or microfibrillar collagen or CoStasis or "GRF Glue" or GR‐Dial or Algosterile or TraumaStat or HemCon or ChitoFlex or Celox or QuikClot or WoundStat or Vitagel or TachSeal or TachoComb or Cryoseal) ) S66 S43 OR S44 OR S45 OR S46 OR S47 OR S48 OR S49 OR S50 OR S51 OR S52 OR S53 OR S54 OR S55 OR S56 OR S57 OR S58 OR S59 OR S60 OR S61 OR S62 OR S63 OR S64 OR S65 S67 (MH "Waxes/TU") S68 TI ( (bonewax* or bone wax* or bone putty or hemasorb or ostene) ) OR AB ( (bonewax* or bone wax* or bone putty or hemasorb or ostene) ) S69 S67 OR S68 S70 (MH "Blood Coagulation Factors") S71 TI ( (prothrombin N5 (complex* or concentrate*)) ) OR AB ( (prothrombin N5 (complex* or concentrate*)) ) S72 TI ( (PCC* or 3F‐PCC* or 4F‐PCC* or Beriplex* or Feiba* or Autoplex* or Ocplex* or Octaplex* or Kcentra* or Cofact or Prothrombinex* or "Proplex‐T" or Prothroraas* or Haemosolvex* or Prothromplex* or "HT Defix" or Facnyne* or Kaskadil* or Kedcom* or Confidex* or PPSB or Profil?ine* or Pronativ* or Proplex* or Prothar* or ProthoRAAS* or Protromplex* or "Pushu Laishi" or "Uman Complex") ) OR AB ( (PCC* or 3F‐PCC* or 4F‐PCC* or Beriplex* or Feiba* or Autoplex* or Ocplex* or Octaplex* or Kcentra* or Cofact or Prothrombinex* or "Proplex‐T" or Prothroraas* or Haemosolvex* or Prothromplex* or "HT Defix" or Facnyne* or Kaskadil* or Kedcom* or Confidex* or PPSB or Profil?ine* or Pronativ* or Proplex* or Prothar* or ProthoRAAS* or Protromplex* or "Pushu Laishi" or "Uman Complex") ) S73 S70 OR S71 OR S72 S74 TI ( (((haemosta* or hemosta* or antihaemorrhag* or antihemorrhag* or anti haemorrhag* or anti‐hemorrhag*) N5 (drug* or agent* or treat* or therap*)) or ((coagulat* or clotting) NEXT factor*)) ) OR AB ( (((haemosta* or hemosta* or antihaemorrhag* or antihemorrhag* or anti haemorrhag* or anti‐hemorrhag*) N5 (drug* or agent* or treat* or therap*)) or ((coagulat* or clotting) NEXT factor*)) ) S75 S29 OR S33 OR S36 OR S39 OR S42 OR S66 OR S69 OR S73 OR S74 S76 (MH Clinical Trials+) S77 PT Clinical Trial S78 TI ((controlled trial*) or (clinical trial*)) OR AB ((controlled trial*) or (clinical trial*)) S79 TI ((singl* blind*) OR (doubl* blind*) OR (trebl* blind*) OR (tripl* blind*) OR (singl* mask*) OR (doubl* mask*) OR (tripl* mask*)) OR AB ((singl* blind*) OR (doubl* blind*) OR (trebl* blind*) OR (tripl* blind*) OR (singl* mask*) OR (doubl* mask*) OR (tripl* mask*)) S80 TI randomi* OR AB randomi* S81 MH RANDOM ASSIGNMENT S82 TI ((phase three) or (phase III) or (phase three)) or AB ((phase three) or (phase III) or (phase three)) S83 ( TI (random* N2 (assign* or allocat*)) ) OR ( AB (random* N2 (assign* or allocat*)) ) S84 MH PLACEBOS S85 MH META ANALYSIS S86 MH SYSTEMATIC REVIEW S87 TI ("meta analys*" OR metaanalys* OR "systematic review" OR "systematic overview" OR "systematic search*") OR AB ("meta analys*" OR metaanalys* OR "systematic review" OR "systematic overview" OR "systematic search*") S88 TI ("literature review" OR "literature overview" OR "literature search*") OR AB ("literature review" OR "literature overview" OR "literature search*") S89 TI (cochrane OR embase OR cinahl OR cinhal OR lilacs OR BIDS OR science AND citation AND index OR cancerlit) OR AB (cochrane OR embase OR cinahl OR cinhal OR lilacs OR BIDS OR science AND citation AND index OR cancerlit) S90 TI placebo* OR AB placebo* S91 MH QUANTITATIVE STUDIES S92 S76 or S77 or S78 or S79 or S80 or S81 or S82 or S83 or S84 or S85 or S86 or S87 or S88 or S89 or S90 or S91 S93 S25 AND S75 AND S92

#### TRANSFUSION EVIDENCE LIBRARY

Clinical Specialty: Cardiovascular Surgery AND Subject Areas: Alternatives to Blood/Antifibrinolytics OR Alternatives to Blood/Fractionated Blood Proucts OR Alternatives to Blood/Recombinant Coagulation Factors

#### ClinicalTrials.gov

1. Other Terms: (interventional radiology OR vascular surgery OR embolectomy OR interarterial OR intravascular OR arteriovenous OR arterial OR endovascular OR revascularization OR iliac OR femoral OR popliteal OR EVAR or FEVAR or TEVAR OR vascular graft) AND Intervention/Treatment: hemostatic OR antifibrinolytic OR tranexamic OR EACA OR aminocaproic OR aprotinin OR desmopressin OR DDAVP OR factor viia OR novoseven OR aryoseven OR fibrinogen OR haemocomplettan OR Riastap OR Fibryna OR Fibryga OR factor XIII OR Tretten OR Other Terms: (thrombectomy OR venous catheterization OR aortic surgery OR aneurysm surgery OR angiosurgery OR devascularization OR angioplasty OR endarterectomy OR atherectomy OR limb salvage OR amputation OR anastomosis) AND Intervention/Treatment: hemostatic OR antifibrinolytic OR tranexamic OR EACA OR aminocaproic OR aprotinin OR desmopressin OR DDAVP OR factor viia OR novoseven OR aryoseven OR fibrinogen OR haemocomplettan OR Riastap OR Fibryna OR Fibryga OR factor XIII OR Tretten

2. Other Terms: (interventional radiology OR vascular surgery OR embolectomy OR interarterial OR intravascular OR arteriovenous OR arterial OR endovascular OR revascularization OR iliac OR femoral OR popliteal OR EVAR or FEVAR or TEVAR OR vascular graft) AND Intervention/Treatment: sealant OR adhesive OR collagen OR cellulose OR gelatin OR glue OR matrix OR sponge OR fleece OR foam OR scaffold OR patch OR sheet OR gelfoam OR chitosan OR hydrogel OR polyethylene glycol OR tachocomb OR BioGlue OR Surgicel OR Veriset OR Other Terms: (thrombectomy OR venous catheterization OR aortic surgery OR aneurysm surgery OR angiosurgery OR devascularization OR angioplasty OR endarterectomy OR atherectomy OR limb salvage OR amputation OR anastomosis) AND Intervention/Treatment: sealant OR adhesive OR collagen OR cellulose OR gelatin OR glue OR matrix OR sponge OR fleece OR foam OR scaffold OR patch OR sheet OR gelfoam OR chitosan OR hydrogel OR polyethylene glycol OR tachocomb OR BioGlue OR Surgicel OR Veriset

3. Other Terms: (interventional radiology OR vascular surgery OR embolectomy OR interarterial OR intravascular OR arteriovenous OR arterial OR endovascular OR revascularization OR iliac OR femoral OR popliteal OR EVAR or FEVAR or TEVAR OR vascular graft) AND Intervention/Treatment: Evithrom OR Floseal OR Tachosil OR Cryoseal OR Hemopatch OR Progel OR Duraseal OR Coseal OR FocalSeal OR Algosterile OR TraumaStat OR HemCon OR ChitoFlex OR Celox OR QuikClot OR WoundStat OR Vitagel OR TachSeal OR bonewax OR hemasorb OR ostene OR Other Terms: (thrombectomy OR venous catheterization OR aortic surgery OR aneurysm surgery OR angiosurgery OR devascularization OR angioplasty OR endarterectomy OR atherectomy OR limb salvage OR amputation OR anastomosis) AND Intervention/Treatment: Evithrom OR Floseal OR Tachosil OR Cryoseal OR Hemopatch OR Progel OR Duraseal OR Coseal OR FocalSeal OR Algosterile OR TraumaStat OR HemCon OR ChitoFlex OR Celox OR QuikClot OR WoundStat OR Vitagel OR TachSeal OR bonewax OR hemasorb OR ostene

4. Other Terms: (interventional radiology OR vascular surgery OR embolectomy OR interarterial OR intravascular OR arteriovenous OR arterial OR endovascular OR revascularization OR iliac OR femoral OR popliteal OR EVAR or FEVAR or TEVAR OR vascular graft) AND Intervention/Treatment: iniprol or kontrikal OR CloSys OR Glubran OR Gluetiss OR Ifabond OR Indermil OR LiquiBand OR Octafil OR Octanate OR Optivate OR Quixil OR Tisseel OR Tissucol OR TissuGlu OR Thrombi‐Gel OR Vivostat OR Voncento OR Wilate OR Wilnativ OR Wilstart OR Other Terms: (thrombectomy OR venous catheterization OR aortic surgery OR aneurysm surgery OR angiosurgery OR devascularization OR angioplasty OR endarterectomy OR atherectomy OR limb salvage OR amputation OR anastomosis) AND Intervention/Treatment: iniprol or kontrikal OR CloSys OR Glubran OR Gluetiss OR Ifabond OR Indermil OR LiquiBand OR Octafil OR Octanate OR Optivate OR Quixil OR Tisseel OR Tissucol OR TissuGlu OR Thrombi‐Gel OR Vivostat OR Voncento OR Wilate OR Wilnativ OR Wilstart

5. Other Terms: (interventional radiology OR vascular surgery OR embolectomy OR interarterial OR intravascular OR arteriovenous OR arterial OR endovascular OR revascularization OR iliac OR femoral OR popliteal OR EVAR or FEVAR or TEVAR OR vascular graft) AND Intervention/Treatment: "prothrombin complex concentrate" OR Beriplex OR Feiba OR Ocplex OR Kcentra OR Prothrombinex OR Other Terms: (thrombectomy OR venous catheterization OR aortic surgery OR aneurysm surgery OR angiosurgery OR devascularization OR angioplasty OR endarterectomy OR atherectomy OR limb salvage OR amputation OR anastomosis) AND Intervention/Treatment: "prothrombin complex concentrate" OR Beriplex OR Feiba OR Ocplex OR Kcentra OR Prothrombinex

6. Other Terms: (interventional radiology OR vascular surgery OR embolectomy OR interarterial OR intravascular OR arteriovenous OR arterial OR endovascular OR revascularization OR iliac OR femoral OR popliteal OR EVAR or FEVAR or TEVAR OR vascular graft) AND Title: hemostasis OR hemostatic OR antifibrinolytic OR clotting factor OR coagulation factor OR fibrinogen OR thrombin OR collagen OR gelatin OR cellulose OR Other Terms: (thrombectomy OR venous catheterization OR aortic surgery OR aneurysm surgery OR angiosurgery OR devascularization OR angioplasty OR endarterectomy OR atherectomy OR limb salvage OR amputation OR anastomosis) AND Title: hemostasis OR hemostatic OR antifibrinolytic OR clotting factor OR coagulation factor OR fibrinogen OR thrombin OR collagen OR gelatin OR cellulose

7. Other Terms: (interventional radiology OR vascular surgery OR embolectomy OR interarterial OR intravascular OR arteriovenous OR arterial OR endovascular OR revascularization OR iliac OR femoral OR popliteal OR EVAR or FEVAR or TEVAR OR vascular graft) AND Condition: bleeding OR hemorrhage OR blood loss OR bloodloss OR Other Terms: (thrombectomy OR venous catheterization OR aortic surgery OR aneurysm surgery OR angiosurgery OR devascularization OR angioplasty OR endarterectomy OR atherectomy OR limb salvage OR amputation OR anastomosis) AND Condition: bleeding OR hemorrhage OR blood loss OR bloodloss

8. 1 OR 2 OR 3 OR 4 OR 5 OR 6 OR 7 [N.B. combined and de‐duplicated in EndNote]

#### WHO ICTRP

1. Title OR Condition: interventional radiology OR vascular surgery OR embolectomy OR interarterial OR intravascular OR arteriovenous OR arterial OR endovascular OR revascularization OR iliac OR femoral OR popliteal OR EVAR or FEVAR or TEVAR OR vascular graft AND Intervention: hemostatic OR antifibrinolytic OR tranexamic OR EACA OR aminocaproic OR aprotinin OR desmopressin OR DDAVP OR factor viia OR novoseven OR aryoseven OR fibrinogen OR haemocomplettan OR Riastap OR Fibryna OR Fibryga OR factor XIII OR Tretten OR Title OR Condition: thrombectomy OR venous catheterization OR aortic surgery OR aneurysm surgery OR angiosurgery OR devascularization OR angioplasty OR endarterectomy OR atherectomy OR limb salvage OR amputation OR anastomosis AND Intervention: hemostatic OR antifibrinolytic OR tranexamic OR EACA OR aminocaproic OR aprotinin OR desmopressin OR DDAVP OR factor viia OR novoseven OR aryoseven OR fibrinogen OR haemocomplettan OR Riastap OR Fibryna OR Fibryga OR factor XIII OR Tretten

2. Title OR Condition: interventional radiology OR vascular surgery OR embolectomy OR interarterial OR intravascular OR arteriovenous OR arterial OR endovascular OR revascularization OR iliac OR femoral OR popliteal OR EVAR or FEVAR or TEVAR OR vascular graft AND Intervention: sealant OR adhesive OR collagen OR cellulose OR gelatin OR glue OR matrix OR sponge OR fleece OR foam OR scaffold OR patch OR sheet OR gelfoam OR chitosan OR hydrogel OR polyethylene glycol OR tachocomb OR BioGlue OR Surgicel OR Veriset OR Title OR Condition: thrombectomy OR venous catheterization OR aortic surgery OR aneurysm surgery OR angiosurgery OR devascularization OR angioplasty OR endarterectomy OR atherectomy OR limb salvage OR amputation OR anastomosis AND Intervention: sealant OR adhesive OR collagen OR cellulose OR gelatin OR glue OR matrix OR sponge OR fleece OR foam OR scaffold OR patch OR sheet OR gelfoam OR chitosan OR hydrogel OR polyethylene glycol OR tachocomb OR BioGlue OR Surgicel OR Veriset

3. Title OR Condition: interventional radiology OR vascular surgery OR embolectomy OR interarterial OR intravascular OR arteriovenous OR arterial OR endovascular OR revascularization OR iliac OR femoral OR popliteal OR EVAR or FEVAR or TEVAR OR vascular graft AND Intervention: Evithrom OR Floseal OR Tachosil OR Cryoseal OR Hemopatch OR Progel OR Duraseal OR Coseal OR FocalSeal OR Algosterile OR TraumaStat OR HemCon OR ChitoFlex OR Celox OR QuikClot OR WoundStat OR Vitagel OR TachSeal OR bonewax OR hemasorb OR ostene OR  Title OR Condition: thrombectomy OR venous catheterization OR aortic surgery OR aneurysm surgery OR angiosurgery OR devascularization OR angioplasty OR endarterectomy OR atherectomy OR limb salvage OR amputation OR anastomosis AND Intervention: Evithrom OR Floseal OR Tachosil OR Cryoseal OR Hemopatch OR Progel OR Duraseal OR Coseal OR FocalSeal OR Algosterile OR TraumaStat OR HemCon OR ChitoFlex OR Celox OR QuikClot OR WoundStat OR Vitagel OR TachSeal OR bonewax OR hemasorb OR ostene

4. Title OR Condition: interventional radiology OR vascular surgery OR embolectomy OR interarterial OR intravascular OR arteriovenous OR arterial OR endovascular OR revascularization OR iliac OR femoral OR popliteal OR EVAR or FEVAR or TEVAR OR vascular graft AND Intervention: iniprol or kontrikal OR CloSys OR Glubran OR Gluetiss OR Ifabond OR Indermil OR LiquiBand OR Octafil OR Octanate OR Optivate OR Quixil OR Tisseel OR Tissucol OR TissuGlu OR Thrombi‐Gel OR Vivostat OR Voncento OR Wilate OR Wilnativ OR Wilstart OR Title OR Condition: thrombectomy OR venous catheterization OR aortic surgery OR aneurysm surgery OR angiosurgery OR devascularization OR angioplasty OR endarterectomy OR atherectomy OR limb salvage OR amputation OR anastomosis AND Intervention: iniprol or kontrikal OR CloSys OR Glubran OR Gluetiss OR Ifabond OR Indermil OR LiquiBand OR Octafil OR Octanate OR Optivate OR Quixil OR Tisseel OR Tissucol OR TissuGlu OR Thrombi‐Gel OR Vivostat OR Voncento OR Wilate OR Wilnativ OR Wilstart ‐ 5. Title OR Condition: interventional radiology OR vascular surgery OR embolectomy OR interarterial OR intravascular OR arteriovenous OR arterial OR endovascular OR revascularization OR iliac OR femoral OR popliteal OR EVAR or FEVAR or TEVAR OR vascular graft AND Intervention: prothrombin complex OR prothrombin concentrate OR Beriplex OR Feiba OR Ocplex OR Kcentra OR Prothrombinex OR Title OR Condition: thrombectomy OR venous catheterization OR aortic surgery OR aneurysm surgery OR angiosurgery OR devascularization OR angioplasty OR endarterectomy OR atherectomy OR limb salvage OR amputation OR anastomosis AND Intervention: prothrombin complex OR prothrombin concentrate OR Beriplex OR Feiba OR Ocplex OR Kcentra OR Prothrombinex

6. Title OR Condition: interventional radiology OR vascular surgery OR embolectomy OR interarterial OR intravascular OR arteriovenous OR arterial OR endovascular OR revascularization OR iliac OR femoral OR popliteal OR EVAR or FEVAR or TEVAR OR vascular graft AND Title OR Intervention: hemostasis OR hemostatic OR antifibrinolytic OR factor OR fibrinogen OR thrombin OR collagen OR gelatin OR cellulose OR Title OR Condition: thrombectomy OR venous catheterization OR aortic surgery OR aneurysm surgery OR angiosurgery OR devascularization OR angioplasty OR endarterectomy OR atherectomy OR limb salvage OR amputation OR anastomosis AND Title OR Intervention: hemostasis OR hemostatic OR antifibrinolytic OR factor OR fibrinogen OR thrombin OR collagen OR gelatin OR cellulose

7. 1 OR 2 OR 3 OR 4 OR 5 OR 6 [N.B. combined and de‐duplicated in EndNote]

## Data and analyses

**Comparison 1:** Systemic drug treatments

Outcome or subgroup title	No. of studies	No. of participants	Statistical method	Effect size
1.1 Number of red blood cells (units) transfused per participant up to 30 days post surgery	3		Mean Difference (IV, Random, 95% CI)	Subtotals only
1.1.1 Aprotinin vs placebo	1	35	Mean Difference (IV, Random, 95% CI)	‐1.56 [‐5.65, 2.53]
1.1.2 Desmopressin vs placebo	2	135	Mean Difference (IV, Random, 95% CI)	‐0.23 [‐0.87, 0.42]
1.2 All‐cause mortality at up to 30 days	5		Risk Ratio (M‐H, Random, 95% CI)	Subtotals only
1.2.1 Aprotinin vs placebo	3	244	Risk Ratio (M‐H, Random, 95% CI)	0.96 [0.61, 1.52]
1.2.2 Desmopressin vs placebo	1	91	Risk Ratio (M‐H, Random, 95% CI)	5.57 [0.27, 112.85]
1.2.3 Tranexamic acid vs placebo	1	100	Risk Ratio (M‐H, Random, 95% CI)	Not estimable
1.3 Risk of receiving any allogeneic blood product at up to 30 days post surgery	1		Peto Odds Ratio (Peto, Fixed, 95% CI)	Totals not selected
1.3.1 Tranexamic acid vs placebo	1		Peto Odds Ratio (Peto, Fixed, 95% CI)	Totals not selected
1.4 Risk of reoperation or repeat procedure for bleeding within 7 days	2		Risk Ratio (M‐H, Random, 95% CI)	Totals not selected
1.4.1 Aprotinin vs placebo	1		Risk Ratio (M‐H, Random, 95% CI)	Totals not selected
1.4.2 Tranexamic acid vs placebo	1		Risk Ratio (M‐H, Random, 95% CI)	Totals not selected
1.5 Risk of a thrombotic/thromboembolic event (MI, CVA, DVT, PE) up to 30‐day follow‐up	2		Risk Ratio (M‐H, Random, 95% CI)	Totals not selected
1.5.1 Desmopressin vs placebo	1		Risk Ratio (M‐H, Random, 95% CI)	Totals not selected
1.5.2 Tranexamic acid vs placebo	1		Risk Ratio (M‐H, Random, 95% CI)	Totals not selected
1.6 Risk of myocardial infarction up to 30 days	4		Peto Odds Ratio (Peto, Fixed, 95% CI)	Subtotals only
1.6.1 Aprotinin vs placebo	2	209	Peto Odds Ratio (Peto, Fixed, 95% CI)	0.41 [0.09, 1.82]
1.6.2 Desmopressin vs placebo	1	91	Peto Odds Ratio (Peto, Fixed, 95% CI)	0.55 [0.11, 2.88]
1.6.3 Tranexamic acid vs placebo	1	100	Peto Odds Ratio (Peto, Fixed, 95% CI)	1.00 [0.14, 7.32]
1.7 Risk of CVA or stroke up to 30 days	2		Peto Odds Ratio (Peto, Fixed, 95% CI)	Subtotals only
1.7.1 Aprotinin vs placebo	2	209	Peto Odds Ratio (Peto, Fixed, 95% CI)	0.36 [0.05, 2.62]
1.8 Risk of deep vein thrombosis up to 90 days	2		Peto Odds Ratio (Peto, Fixed, 95% CI)	Totals not selected
1.8.1 Aprotinin vs placebo	1		Peto Odds Ratio (Peto, Fixed, 95% CI)	Totals not selected
1.8.2 Desmopressin vs placebo	1		Peto Odds Ratio (Peto, Fixed, 95% CI)	Totals not selected
1.9 Risk of pulmonary embolism up to 90 days	3		Peto Odds Ratio (Peto, Fixed, 95% CI)	Totals not selected
1.9.1 Aprotinin vs placebo	1		Peto Odds Ratio (Peto, Fixed, 95% CI)	Totals not selected
1.9.2 Desmopressin vs placebo	1		Peto Odds Ratio (Peto, Fixed, 95% CI)	Totals not selected
1.9.3 Tranexamic acid vs placebo	1		Peto Odds Ratio (Peto, Fixed, 95% CI)	Totals not selected

**Comparison 2:** Topical drug treatments

Outcome or subgroup title	No. of studies	No. of participants	Statistical method	Effect size
2.1 Number of red blood cells (units) transfused per participant up to 30 days	1		Mean Difference (IV, Random, 95% CI)	Subtotals only
2.1.1 Thrombin+fibrin/collagen sponge vs usual care	1	20	Mean Difference (IV, Random, 95% CI)	‐0.10 [‐0.80, 0.60]
2.2 All‐cause mortality at up to 30 days	10		Risk Ratio (M‐H, Random, 95% CI)	Subtotals only
2.2.1 Thrombin+fibrin/collagen sponge vs usual care	1	22	Risk Ratio (M‐H, Random, 95% CI)	3.00 [0.14, 66.53]
2.2.2 Human thrombin/gelatin sponge vs bovine thrombin/gelatin sponge	1	31	Risk Ratio (M‐H, Random, 95% CI)	Not estimable
2.2.3 Collagen dressing vs oxidised cellulose	1	16	Risk Ratio (M‐H, Random, 95% CI)	3.00 [0.14, 64.26]
2.2.4 Novel agent/gelatin sponge vs placebo/gelatin sponge	1	54	Risk Ratio (M‐H, Random, 95% CI)	Not estimable
2.2.5 Fibrin sealant vs usual care	3	585	Risk Ratio (M‐H, Random, 95% CI)	0.44 [0.11, 1.76]
2.2.6 Fibrin sealant vs gelatin sponge	1	176	Risk Ratio (M‐H, Random, 95% CI)	2.46 [0.29, 20.55]
2.2.7 Fibrin sealant vs thrombin/gelatin sponge	1	199	Risk Ratio (M‐H, Random, 95% CI)	0.50 [0.09, 2.64]
2.2.8 Synthetic sealant vs thrombin/gelatin sponge	1	89	Risk Ratio (M‐H, Random, 95% CI)	0.10 [0.01, 1.71]
2.3 Risk of receiving any allogeneic blood product at up to 30 days post surgery	2		Risk Ratio (M‐H, Random, 95% CI)	Subtotals only
2.3.1 Fibrin/collagen sponge vs usual care	1	60	Risk Ratio (M‐H, Random, 95% CI)	0.33 [0.12, 0.92]
2.3.2 Thrombin+fibrin/collagen sponge vs usual care	1	22	Risk Ratio (M‐H, Random, 95% CI)	0.20 [0.01, 3.74]
2.4 Risk of reoperation or repeat procedure for bleeding within 7 days	3		Risk Ratio (M‐H, Random, 95% CI)	Subtotals only
2.4.1 Fibrin sealant vs usual care	1	160	Risk Ratio (M‐H, Random, 95% CI)	1.03 [0.31, 3.40]
2.4.2 Fibrin sealant vs gelatin sponge	1	175	Risk Ratio (M‐H, Random, 95% CI)	0.17 [0.01, 4.03]
2.4.3 Synthetic sealant vs thrombin/gelatin sponge	1	89	Risk Ratio (M‐H, Random, 95% CI)	0.36 [0.01, 8.51]
2.5 Risk of a thrombotic/thromboembolic event (MI, CVA, DVT, PE) up to 30‐day follow‐up	6		Peto Odds Ratio (Peto, Fixed, 95% CI)	Totals not selected
2.5.1 Fibrin/collagen sponge vs usual care	1		Peto Odds Ratio (Peto, Fixed, 95% CI)	Totals not selected
2.5.2 Thrombin+fibrin/collagen sponge vs usual care	2		Peto Odds Ratio (Peto, Fixed, 95% CI)	Totals not selected
2.5.3 Collagen dressing vs oxidised cellulose	1		Peto Odds Ratio (Peto, Fixed, 95% CI)	Totals not selected
2.5.4 Fibrin sealant vs usual care	1		Peto Odds Ratio (Peto, Fixed, 95% CI)	Totals not selected
2.5.5 Synthetic sealant vs thrombin/gelatin sponge	1		Peto Odds Ratio (Peto, Fixed, 95% CI)	Totals not selected
2.6 Risk of a myocardial infarction up to 30 days	4		Peto Odds Ratio (Peto, Fixed, 95% CI)	Totals not selected
2.6.1 Thrombin+fibrin/collagen sponge vs usual care	1		Peto Odds Ratio (Peto, Fixed, 95% CI)	Totals not selected
2.6.2 Collagen dressing vs oxidised cellulose	1		Peto Odds Ratio (Peto, Fixed, 95% CI)	Totals not selected
2.6.3 Fibrin sealant vs usual care	2		Peto Odds Ratio (Peto, Fixed, 95% CI)	Totals not selected
2.7 Risk of a CVA or stroke up to 30 days	3		Peto Odds Ratio (Peto, Fixed, 95% CI)	Totals not selected
2.7.1 Collagen dressing vs oxidised cellulose	1		Peto Odds Ratio (Peto, Fixed, 95% CI)	Totals not selected
2.7.2 fibrin sealant vs usual care	2		Peto Odds Ratio (Peto, Fixed, 95% CI)	Totals not selected
2.8 Risk of a deep vein thrombosis up to 90 days	2		Peto Odds Ratio (Peto, Fixed, 95% CI)	Totals not selected
2.8.1 Collagen dressing vs oxidised cellulose	1		Peto Odds Ratio (Peto, Fixed, 95% CI)	Totals not selected
2.8.2 Fibrin sealant vs usual care	1		Peto Odds Ratio (Peto, Fixed, 95% CI)	Totals not selected
2.9 Risk of a pulmonary embolism up to 90 days	3		Peto Odds Ratio (Peto, Fixed, 95% CI)	Totals not selected
2.9.1 Collagen dressing vs oxidised cellulose	1		Peto Odds Ratio (Peto, Fixed, 95% CI)	Totals not selected
2.9.2 Novel agent (Peprostat) vs placebo/gelatin sponge	1		Peto Odds Ratio (Peto, Fixed, 95% CI)	Totals not selected
2.9.3 Fibrin sealant vs usual care	1		Peto Odds Ratio (Peto, Fixed, 95% CI)	Totals not selected

## Characteristics of studies

### Characteristics of included studies [ordered by study ID]

**Table:** Bajardi 2009

***Study characteristics***		
Methods	**Design:** RCT (parallel group) **Anticoagulation cessation:** NR **Antiplatelet cessation:** NR **Power calculation reached:** NR **Prophylactic or therapeutic randomisation:** therapeutic: "Sealed code envelope method was used to randomize patients to either treatment with TachoSil use or to standard compression with surgical swabs. The randomization code envelopes were opened just before the application of haemostatic measures. The nature of the treatments precluded blinding of the study." **Recruitment end date:** June 2008 **Recruitment start date:** June 2007 **Stopping rule:** NR **Strategy for analysis (ITT/PPA):** ITT **Transfusion strategy protocol:** NR **Transfusion threshold in trial:** NA **Trial stopped early:** no	
Participants	**Inclusion criteria:** "20 patients with intact infra‐renal abdominal aortic aneurysm (AAA) were randomized in the same centre." Mean transverse diameter was 7.1 +/‐ 1.2 cm (range 5.6 to 9 cm) in Group I and 6.8 +/‐1 cm (range 5.5 to 8.6 cm) in Group II. **Exclusion criteria:** "Allergy to any component of TachoSil was considered for exclusion prior the randomization. Patients, who did not require additional haemostatic measures on releasing the clamps, were also excluded from the study. Likewise patients with alteration of clotting parameters or liver diseases were excluded from the study." **Group differences:** there were no significant baseline differences in age, gender, smoking history, hypertension DM, COPD, CAD, CVA, renal failure or median aneurysm size. **Indirectness:** no **Protocol deviations:** no participants were excluded after the randomisation. **Reasons for dropouts:** no participants were excluded after the randomisation. **Number of participants randomised:** Tachosil: 10; Usual care: 10 **Number of participants receiving treatment:** Tachosil: 10; Usual care: 10 **Number of participants analysed:** Tachosil: 10; Usual care: 10 **Number of participants dropping out:** Tachosil: 0; Usual care: 0 **Male gender (%):** Tachosil: 80%; Usual care: 90% **Age (years):** Tachosil: mean 72.8 range 63 to 80; Usual care: mean 72.6 range 67 to 82 **Weight (kg):** NR **Ethnicity:** NR **Preoperative haemoglobin (g/L):** NR **Perioperative antiplatelet use (excluding aspirin) (%)** **Perioperative aspirin use (%):** NR **Perioperative anticoagulants (%):** NR **Revision surgery (%):** NR **Elective surgery (%):** Tachosil: 100%; Usual care: 100% **Risk score (system used & score):** NR **Cross clamp use (%):** Tachosil: 100%; Usual care: 100% **Cross clamp duration (mins):** NR **Duration of surgery (mins):** Tachosil: mean 117 SD 15; Usual care: mean 119.9 SD 20.7 **Surgical approach/es:** OSR **Surgical pathology/ies:** replacement of infra‐renal AAA **Anatomical region/s:** infra‐renal	
Interventions	**Standardised name:** TachoSil: collagen matrix coated with fibrinogen and thrombin (TachoSil); Usual care: manual compression with gauze **Description in text:** TachoSil: "TachoSil® patches were applied in Group I after moistening with physiological saline after clamp removing. Dosage (the number of patches used) depended on the size of the area to be covered. One patch of TachoSil® is 9.5 × 4.8× 0.5 cm in size. It contains a fixed combination of a collagen matrix coated with the coagulation factors, human fibrinogen (5.5 mg/cm^2^) and human thrombin (2.0 IU/cm^2^)."; Usual care: "patients in the control arm received compression with 10 × 10 cm surgical swabs."	
Outcomes	**Red cell transfusions at up to 30 days postsurgery:** TachoSil: mean 1.3 SD 0.8 **All‐cause mortality at up to 30 days; and at between 31 and 90 days:** NR **Risk of receiving any allogeneic blood product (composite) at up to 30 days postsurgery:** NR **Risk of receiving any allogeneic packed red cells at up to 30 days postsurgery:** NR **Risk of receiving any allogeneic platelets at up to 30 days postsurgery:** NR **Risk of receiving any allogeneic fresh frozen plasma at up to 30 days postsurgery:** NR **Risk of reoperation or repeat procedure for bleeding within 7 days:** NR **Risk of a thrombotic/thromboembolic event (any composite measured in study) (n/N):** Tachosil 0/10; Usual care: 0/10 **Risk of cerebrovascular attack (CVA/stroke) at up to 30 days: NR** **Risk of myocardial infarction (MI) at up to 30 days:** Tachosil 0/10; Usual care: 1/10 **Risk of deep vein thrombosis (DVT) at up to 90 days:** NR **Risk of pulmonary embolus (PE) at up to 90 days:** NR **Risk of a serious adverse event (SAE) at up to 30 days:** NR **Length of hospital stay (days):** NR **Cost data presented:** no	
Notes	**Sponsorship source:** NR **Country:** Italy **Setting:** single‐centre **Trial primary outcome:** primary outcome was time to achieve haemostasis at the suture line. Haemostasis was defined as the time when the abdominal or inguinal wound was completely dry or sufficiently dry to complete the operation without additional haemostatic measures. **Reference type:** full text **Translation into English required:** no **Trial registration number:** NR **Trial registration timing:** NA	
***Risk of bias***		
**Bias**	**Authors' judgement**	**Support for judgement**
Random sequence generation (selection bias)	Unclear risk	"Between June 2007 and June 2008 a total of 20 patients with intact infrarenal abdominal aortic aneurysm (AAA) were randomized in the same center." Method of sequence generation for randomisation not described.
Allocation concealment (selection bias)	Low risk	"Sealed code envelope method was used to randomize patients to either treatment with TachoSil ® use or to standard compression with surgical swabs. The randomization code envelopes were opened just before the application of haemostatic measures." Sealed code method acceptable, though does not specify that envelopes were sealed, opaque and sequentially numbered etc.
Blinding of participants and personnel (performance bias) All outcomes	High risk	"The nature of the treatments precluded blinding of the study." Subjective outcomes ‐ high risk of bias due to inadequate personnel blinding because comparator is usual care rather than placebo. Objective outcomes ‐ low risk of bias.
Blinding of outcome assessment (detection bias) All outcomes	High risk	Subjective outcomes ‐ high risk of bias due to absent outcome assessor blinding. Objective outcomes ‐ low risk of bias.
Incomplete outcome data (attrition bias) All outcomes	Low risk	Outcome data set reported with denominators and numerators and with no dropouts reported.
Selective reporting (reporting bias)	Unclear risk	No available prospective protocol or trial registration.
Other bias	Low risk	No other concerns such as early stopping or imbalanced study arms.

**Table:** Bochicchio 2015

***Study characteristics***		
Methods	**Design:** RCT (parallel group) **Anticoagulation cessation:** anticoagulant was not an exclusion criterion. Anticoagulant agents included direct factor Xa inhibitors, synthetic pentasaccharide inhibitors of factor X, direct thrombin inhibitors, heparin, low molecular weight heparin, and vitamin K antagonists. **Antiplatelet cessation:** antiplatelet use was not an exclusion criterion. Antiplatelet agents included adenosine diphosphate receptor inhibitors, cyclooxygenase inhibitors, glycoprotein IIb/IIIa inhibitors (intravenous use only), and phosphodiesterase inhibitors. **Power calculation reached:** yes, "Assuming 86% of patients achieved hemo‐stasis within 5 minutes and a hazard ratio of 1.70, a sample size of 168 patients undergoing a vascular procedure (Fibrocaps: 112; Gelatin sponge: 56) provided 85% power to detect a 70% difference between treatment arms (two‐sided, overall alpha level of.05)." **Prophylactic or therapeutic randomisation:** prophylactic **Recruitment end date:** April 2013 **Recruitment start date:** May 2012 **Stopping rule:** no **Strategy for analysis (ITT/PPA):** ITT for adverse events. PPA for all other outcomes. **Transfusion strategy protocol:** ITT for adverse events. "Of 230 screened patients, 176 were randomized to treatment with Fibrocaps plus gelatin sponge (n=118) or gelatin sponge alone (n=58), 175 patients were treated (n=117; n=58), and 163 completed the study (n=107/118 [91%]; n=56/58 [97%];Fig 1). One patient randomized to Fibrocaps plus gelatin sponge was not treated because of lack of an appropriate target bleeding site. Death (Fibrocaps, n=5; Gelatin sponge, n=1) and lost to follow‐up (n=4, n=0) were the most common reasons for discontinuing from the study." **Transfusion threshold in trial:** NA **Trial stopped early:** no	
Participants	**Inclusion criteria:** "Has signed an institutional review board/independent ethics committee (IRB/IEC)‐approved informed consent document; is undergoing one of the 4 surgical procedures described; Is at least 18 years old at time of consent; if female and of child‐bearing potential, has negative pregnancy test during screening and is not breast‐feeding; if able to reproduce, agrees to use a medically accepted form of contraception from the time of consent to completion of all follow‐up study visits when engaging in heterosexual intercourse; has not received blood transfusion between screening and study treatment; has mild to moderate surgical bleeding; does not have intra‐operative complications; has not used a topical hemostat containing thrombin prior to study treatment; has an approximate bleeding site surface area of less than or equal to 100 cm^2^; The trial included patients undergoing procedures in one of four different surgical indications (spinal, hepatic, vascular, or soft tissue dissection). The subset described herein included patients who underwent vascular surgical procedures, with mild to moderate surgical bleeding." **Exclusion criteria:** "Has known antibodies or hypersensitivity to thrombin or other coagulation factors; has history of heparin‐induced thrombocytopenia (only for vascular subjects where heparin use is required); has known allergy to gelatin sponge; Is unwilling to receive blood products; has liver enzymes appropriate for the study, considering their disease; has appropriate level of platelets per liter (PLT/L) during screening; has any clinically‐significant coagulation disorder that may interfere with the assessment of efficacy or pose a safety risk to the subject according to the Investigator, or baseline abnormalities during screening that are not explained by current drug treatment (e.g., warfarin, heparin)" **Group differences:** no. "Demographic and surgical characteristics were comparable between treatment arms." **Indirectness:** population also included carotid endarterectomy and AV graft for haemodialysis formation, otherwise no. **Protocol deviations:** "Of 230 screened patients, 176 were randomized to treatment with Fibrocaps plus gelatin sponge (n=118) or gelatin sponge alone (n=58), 175 patients were treated (n=117; n=58), and 163 completed the study (n=107/118 [91%]; n=56/58 [97%]; Fig 1). One patient randomized to Fibrocaps plus gelatin sponge was not treated because of lack of an appropriate target bleeding site." **Reasons for dropouts:** "Death (Fibrocaps, n=5; Gelatin sponge, n=1) and lost to follow‐up (n=4, n=0) were the most common reasons for discontinuing from the study" **Number of participants randomised:** Fibrocaps: 118; Gelatin sponge: 58 **Number of participants receiving treatment:** Fibrocaps: 117; Gelatin sponge: 58 **Number of participants analysed:** Fibrocaps: 117; Gelatin sponge: 58 **Number of participants dropping out:** Fibrocaps: 1; Gelatin sponge: 0 **Male gender (%):** Fibrocaps: 70%; Gelatin sponge: 66% **Age (years):** Fibrocaps: mean 65.1 SD 10.5; Gelatin sponge: mean 66.7 SD 9.9 **Weight (kg):** NR **Ethnicity:** Fibrocaps: White 89%, Black/ African American 7%, Asian, other or NR 4%; Gelatin sponge: White 90%, Black/ African American 5% Asian/other 5% **Preoperative haemoglobin (g/L):** NR **Perioperative antiplatelet use (excluding aspirin) (%):** Fibrocaps: antiplatelet only, 20%, anticoagulant and antiplatelet 37%; Gelatin sponge: antiplatelet only, 17%, anticoagulant and antiplatelet 38% **Perioperative aspirin use (%):** NR **Perioperative anticoagulants (%):** Fibrocaps: 24; Gelatin sponge: 26 **Revision surgery (%):** NR **Elective surgery (%):** NR **Risk score (system used & score):** NR **Cross clamp use (%):** NR **Cross clamp duration (mins):** NR **Duration of surgery (mins):** Fibrocaps: median 168 range 54 to 486; Gelatin sponge: median 168 range 54 to 354 **Surgical approach/es:** open **Surgical pathology/ies:** Fibrocaps: arterial bypass 78%, arteriovenous graft formation for haemodialysis access 11%, carotid endarterectomy 9%, other 2%; Gelatin sponge: arterial bypass 88%, arteriovenous graft formation for haemodialysis access 5%, carotid endarterectomy 7% **Anatomical region/s:** NR	
Interventions	**Standardised name:** Fibrocaps: Fibrocaps liquid (human fibrinogen and thrombin) followed by a gelatin sponge; Gelatin sponge: gelatin sponge **Description in text:** Fibrocaps: "Up to three vials of Fibrocaps (1.0 g each) could be applied to the target bleeding site; each vial contained 79 mg of human‐plasma‐derived fibrinogen and 726 IU of human‐plasma‐derived thrombin (each was sourced from donors at U.S. Foodand Drug Administration [FDA]‐approved collection centers)"; Gelatin sponge: "Spongostan absorbable gelatin sponges (Ethicon, Inc, Somerville, NJ) were used at European sites and Gelfoam absorbable gelatin sponges (Pfizer, New York, NY) were used at U.S. sites; sponges were used according to the manufacturers’ instructions"	
Outcomes	**Red cell transfusions at up to 30 days postsurgery:** NR **All‐cause mortality at up to 30 days:** Fibrocaps: 5; Gelatin sponge: 1 **and at between 31 and 90 days:** NR **Risk of receiving any allogeneic blood product (composite) at up to 30 days postsurgery:** NR **Risk of receiving any allogeneic packed red cells at up to 30 days postsurgery:** Fibrocaps: 11; Gelatin sponge: 4 **Risk of receiving any allogeneic platelets at up to 30 days postsurgery:** NR **Risk of receiving any allogeneic fresh frozen plasma at up to 30 days post‐surgery:** NR **Risk of reoperation or repeat procedure for bleeding within 7 days:** Fibrocaps: 0; Gelatin sponge: 1 **Risk of a thrombotic/thromboembolic event (any composite measured in study) (n/N):** NR **Risk of cerebrovascular attack (CVA/stroke) at up to 30 days:** NR **Risk of myocardial infarction (MI) at up to 30 days:** NR **Risk of deep vein thrombosis (DVT) at up to 90 days:** NR **Risk of pulmonary embolus (PE) at up to 90 days:** NR **Risk of a serious adverse event (SAE) at up to 30 days:** Fibrocaps: 25; Gelatin sponge: 12 **Length of hospital stay (days):** NR **Cost data presented:** no	
Notes	**Sponsorship source:** pharmaceutical (ProFibrix BV, a subsidiary of The medicines Company). Two employees on authorship and consulting fees to three other authors or their departments. **Country:** The Netherlands (6), UK (10), USA (9) **Setting:** multi‐centre (25) **Trial primary outcome:** Endpoints. "As previously described, the primary efficacy endpoint was TTH within the 5‐minute TTH assessment period. The secondary efficacy endpoints were treatment differences in restricted mean TTH over 5 minutes and the probabilities of TTH at 3 and 5 minutes. Safety endpoints included the incidence, severity and relationship to treatment of adverse events, the assessment of clinical laboratory abnormalities, the results of immunogenicity assessments, and the presence of postsurgical bleeding complications." **Reference type:** full text, clinical trial register **Translation into English required:** no **Trial registration number:** NCT01527357 FC‐004; FINISH‐3; EudraCT:2011‐006174‐47 **Trial registration timing:** prospective	
***Risk of bias***		
**Bias**	**Authors' judgement**	**Support for judgement**
Random sequence generation (selection bias)	Unclear risk	The article says "random", but no further details about how it was randomised. Method of sequence generation for randomisation not described.
Allocation concealment (selection bias)	Unclear risk	Method of allocation concealment not described.
Blinding of participants and personnel (performance bias) All outcomes	High risk	"this single‐blind trial" Blinding not possible. Subjective outcomes ‐ low risk of bias due to adequate personnel blinding. Objective outcomes ‐ low risk of bias.
Blinding of outcome assessment (detection bias) All outcomes	High risk	Subjective outcomes ‐ low risk of bias due to adequate outcome assessor blinding. Objective outcomes ‐ low risk of bias.
Incomplete outcome data (attrition bias) All outcomes	Low risk	9% of the Fibrocaps group and 3% of the Gelatin sponge group did not complete the study. However because some of these were due to death, overall, less than 5% drop out rate per arm and low risk of bias. Outcome data set reported with denominators and numerators
Selective reporting (reporting bias)	Low risk	All outcomes planned in the protocol or prospective trial registration were reported.
Other bias	Low risk	No other concerns such as early stopping or imbalanced study arms.

**Table:** Chetter 2017

***Study characteristics***		
Methods	**Design:** RCT (parallel group) **Anticoagulation cessation:** NR **Antiplatelet cessation:** NR **Power calculation reached:** yes, total of 168 calculated, 168 recruited. "The sample size for the Primary Study was calculated to provide at least 80% power to detect a difference between the proportions of patients achieving hemostasis during the 10‐min observational period. Assuming that 45% of the MC group and 70% of the FS Grifols group could be expected to exhibit hemostasis and using a 2‐group continuity‐corrected chi‐squared test at the 5% significance level with a 2:1 randomization ratio, a total of 153 patients (102 treated with FS Grifols and 51 treated with MC) were expected to provide 80% power. Assuming a 10% dropout rate after randomization, a total of 168 patients was required in the Primary Study." **Prophylactic or therapeutic randomisation:** therapeutic **Recruitment end date:** NR **Recruitment start date:** NR **Stopping rule:** NR **Strategy for analysis (ITT/PPA):** modified ITT **Transfusion strategy protocol:** NR **Transfusion threshold in trial:** NA **Trial stopped early:** no	
Participants	**Inclusion criteria:** "Patients were eligible to participate if they required an elective (nonemergency), open arterial surgery listed in the study protocol involving use of either Dacron or PTFE, for which a target bleeding site (TBS) was identified and a topical hemostat was indicated. A TBS was defined as a bleeding site requiring an adjunct treatment to hemostasis because conventional surgical techniques (including suture, ligature, and cautery) were ineffective or impractical. For entry into the study, all patients should meet the following inclusion criteria: hemoglobin > 9.0 g/dL, platelet count > 70 x 10*3/mL, willingness and ability to complete all protocol requirements, and had an intraoperative identified TBS with arterial bleeding. In the Primary Study, the number of patients with each type of graft (Dacron or PTFE) had to be balanced, with no more than approximately 90 patients with either type of graft planned to be enrolled in the Primary Study, and a majority of the TBSs treated were to be of moderate intensity (gradual and steady)." **Exclusion criteria:** "Exclusion criteria included the following: patients with baseline coagulopathy, liver disorder, or uremia; reoperative procedure; infection in the surgical field; history of severe reactions to any blood‐derived product; positive bleeding history; history of alcohol or drug abuse within the previous 12 months; pregnancy or lactation; treatment with any investigational agent in the past 3 months; and patients with intraoperative severe (brisk and forceful) arterial bleeding at the TBS according to the investigators’ judgment." **Number of participants randomised:** Fibrin sealant: 111; Usual care: 57 **Number of participants receiving treatment:** Fibrin sealant: 110; Usual care: 57 **Number of participants analysed:** Fibrin sealant: 110; Usual care: 57 **Number of participants dropping out:** Fibrin sealant: 1; Usual care: 0 **Male gender (%):** Fibrin sealant: 86.4%; Usual care: 68.4% **Age (years):** Fibrin sealant: mean 64.8 SD 8.9; Usual care: mean 67.0 SD 10.3 **Weight (kg):** Fibrin sealant: mean 79.5 SD 20.5; Usual care: mean 74.6 SD 14.1 **Ethnicity:** Fibrin sealant: Caucasian 99.1%; Usual care: Caucasian 100% **Preoperative haemoglobin (g/L):** NR **Perioperative antiplatelet use (excluding aspirin) (%):** NR **Perioperative aspirin use (%):** NR **Perioperative anticoagulants (%):** "100% were taking concurrent antithrombotic medications" **Revision surgery (%):** 0 **Elective surgery (%):** 100% **Risk score (system used & score):** NR **Cross clamp use (%):** 100% **Cross clamp duration (mins):** NR **Duration of surgery (mins):** NR **Surgical approach/es:** open **Surgical pathology/ies:** aneurysm, bypass, resection, other **Anatomical region/s:** carotid, femoral, abdominal	
Interventions	**Standardised name:** Fibrin sealant: fibrin sealant (Grifols); Usual care: usual care (manual compression with gauze) **Description in text:** Fibrin sealant: "Fibrin Sealant Grifols: Combination of 3 mL fibrinogen and 3 mL thrombin, in separate syringes assembled on a syringe holder (6 mL of solution in total), applied topically to the target bleeding site"; Usual care: "direct MC was applied immediately to the TBS using gauze swabs, protocol: Surgicel® is a sterile, absorbable knitted fabric prepared by the controlled oxidation of regenerated cellulose. Up to four Surgicel® sheets applied to the target bleeding site according to Package Insert instructions and the surgeon's usual clinical practice."	
Outcomes	**Red cell transfusions at up to 30 days postsurgery:** NR **All‐cause mortality at up to 30 days;** Fibrin sealant: 2/110; Usual care: 1/57 **and at between 31 and 90 days:** NR **Risk of receiving any allogeneic blood product (composite) at up to 30 days postsurgery:** NR **Risk of receiving any allogeneic packed red cells at up to 30 days postsurgery:** NR **Risk of receiving any allogeneic platelets at up to 30 days postsurgery:** NR **Risk of receiving any allogeneic fresh frozen plasma at up to 30 days postsurgery:** NR **Risk of reoperation or repeat procedure for bleeding within 7 days:** NR **Risk of a thrombotic/thromboembolic event (any composite measured in study) (n/N):** NR **Risk of cerebrovascular attack (CVA/stroke) at up to 30 days:** NR **Risk of myocardial infarction (MI) at up to 30 days:** NR **Risk of deep vein thrombosis (DVT) at up to 90 days:** NR **Risk of pulmonary embolus (PE) at up to 90 days:** NR **Risk of a serious adverse event (SAE) at up to 30 days:** NR **Length of hospital stay (days):** NR **Cost data presented:** no	
Notes	**Sponsorship source:** pharmaceutical (Grifols, S.A). One employee on the authorship and medical writers funded by the manufacturer are acknowledged. **Country:** Canada, Spain, UK **Setting:** multicentre (19) **Trial primary outcome:** proportion of subjects achieving haemostasis by four minutes after treatment start. Secondary outcomes: time to haemostasis (TTH); cumulative proportion of subjects having achieved haemostasis at the target bleeding site by specified time points; prevalence of treatment failures **Reference type:** full text **Translation into English required:** no **Trial registration number:** NCT01754480 ‐ refers to all surgery types not just vascular, but methods quote this protocol. **Trial registration timing:** NA	
***Risk of bias***		
**Bias**	**Authors' judgement**	**Support for judgement**
Random sequence generation (selection bias)	Low risk	"randomization function of the statistics software" Adequate method of sequence generation with computer‐generated random numbers.
Allocation concealment (selection bias)	Low risk	"and communicated using sealed opaque envelopes." Method of allocation concealment likely to be adequate.
Blinding of participants and personnel (performance bias) All outcomes	High risk	"Due to the obvious differences between the 2 treatments, blinding of investigators was not possible following randomization." Subjective outcomes ‐ high risk of bias due to absent personnel blinding. Objective outcomes ‐ low risk of bias regardless.
Blinding of outcome assessment (detection bias) All outcomes	High risk	Subjective outcomes ‐ high risk of bias due to absent outcome assessor blinding. Objective outcomes ‐ low risk of bias.
Incomplete outcome data (attrition bias) All outcomes	Unclear risk	Outcome data set reported of both randomised and non‐randomised participants pooled together.
Selective reporting (reporting bias)	Unclear risk	The prospective protocol or trial registration mentioned in the article is not correct, and is from another study, so the trial protocol is unknown.
Other bias	Low risk	No other concerns such as early stopping or imbalanced study arms.

**Table:** Clagett 1995

***Study characteristics***		
Methods	**Design:** RCT (parallel group) **Anticoagulation cessation:** NR **Antiplatelet cessation:** yes, "Patients with the following conditions were ineligible: aspirin ingestion within 7 days of operation" **Power calculation reached:** no power calculation reported **Prophylactic or therapeutic randomisation:** prophylactic **Recruitment end date:** NR **Recruitment start date:** NR **Stopping rule:** NR **Strategy for analysis (ITT/PPA):** ITT **Transfusion strategy protocol:** NR **Transfusion threshold in trial:** NA **Trial stopped early:** no	
Participants	**Inclusion criteria:** "Patients undergoing elective infra‐renal aortic aneurysm repair or aorto‐femoral bypass for occlusive disease were eligible for entrance into this study" **Exclusion criteria:** "Patients with the following conditions were ineligible: aspirin ingestion within 7 days of operation, acquired or congenital haemorrhagic diathesis, emergency operation, creatinine > 3 mg/dl, thoraco‐ abdominal reconstruction, aortorenal or visceral by‐pass, or refusal to join the study." **Group differences:** "All patients were male, and no significant differences were seen between groups in age, body habitus, smoking history, hypertension, diabetes mellitus, chronic obstructive pulmonary disease, elevated cholesterol, coronary artery disease, and renal insufficiency (creatinine between 1.8 and 3.0 mg/dl). Slightly more than one half the patients in each group underwent operation for aortic aneurysm, and no significant difference was seen between groups in aneurysm size." **Indirectness:** all patients were male. **Protocol deviations:** NA, none. **Reasons for dropouts:** NA, none. **Number of participants randomised:** Desmopressin: 43; Placebo: 48 **Number of participants receiving treatment:** Desmopressin: 43; Placebo: 48 **Number of participants analysed:** Desmopressin: 43; Placebo: 48 **Number of participants dropping out:** 0 **Male gender (%):** Desmopressin: 100%; Placebo: 100% **Age (years):** Desmopressin: mean 62 SD 9; Placebo: mean 64 SD 8 **Weight (kg):** Desmopressin: mean 77 SD 18; Placebo: mean 82 SD 17 **Ethnicity:** NR **Preoperative haemoglobin (g/L):** Desmopressin: % haematocrit 39.7 SD 4.6; Placebo: % haematocrit 40.6 SD 5.1 **Perioperative antiplatelet use (excluding aspirin) (%):** 0 **Perioperative aspirin use (%):** 0 **Perioperative anticoagulants (%):** NR **Revision surgery (%):** NR **Elective surgery (%):** 100% **Risk score (system used & score):** NR **Cross clamp use (%):** 100% **Cross clamp duration (mins):** Desmopressin: mean 83 SD 43; Placebo: mean 82 SD 30 **Duration of surgery (mins):** Desmopressin: mean 235 SD 73; Placebo: mean 252 SD 76 **Surgical approach/es:** open **Surgical pathology/ies:** infrarenal aortic aneurysm and aortofemoral bypass **Anatomical region/s:** infra‐renal	
Interventions	**Standardised name:** Desmopressin: desmopressin 20 mcg iv bolus to patient at aortic clamp placement; Placebo: placebo NS EV **Description in text:** Desmopressin: "Patients were randomized by drawing a sealed envelope that contained a prescription for either 20 ug DDAVP (Rorer Pharmaceuticals, Fort Washington, Pa.) or placebo. DDAVP or placebo was administered intravenously for 15 minutes immediately after intravenous heparinization with 100 U/kg was performed and just before aortic cross‐clamp application was done"; Placebo: "or placebo"	
Outcomes	**Red cell transfusions at up to 30 days postsurgery: intraoperative and up to 72 hours postoperative:** Desmopressin: mean 3.1 SD 3, n = 43; Placebo: mean 2.7 SD 3, n = 48 **All‐cause mortality at up to 30 days;** Desmopressin: 2/43; Placebo: 0/48; **and at between 31 and 90 days:** NR **Risk of receiving any allogeneic blood product (composite) at up to 30 days postsurgery:** NR **Risk of receiving any allogeneic packed red cells at up to 30 days postsurgery:** Desmopressin: 33/43; Placebo: 35/48 **Risk of receiving any allogeneic platelets at up to 30 days postsurgery:** NR **Risk of receiving any allogeneic fresh frozen plasma at up to 30 days postsurgery:** NR **Risk of reoperation or repeat procedure for bleeding within 7 days:** NR **Risk of a thrombotic/thromboembolic event (any composite measured in study) (n/N):** Desmopressin: 6/43; Placebo: 7/48. **Risk of cerebrovascular attack (CVA/stroke) at up to 30 days:** NR **Risk of myocardial infarction (MI) at up to 30 days:** Desmopressin: 2/43; Placebo: 4/48 **Risk of deep vein thrombosis (DVT) at up to 90 days:** Desmopressin: 1/43; Placebo: 0/48 **Risk of pulmonary embolus (PE) at up to 90 days:** Desmopressin: 1/43; Placebo: 0/48 **Risk of a serious adverse event (SAE) at up to 30 days:** NR **Length of hospital stay (days):** NR **Cost data presented:** no	
Notes	**Sponsorship source:** Source of funding not stated but manufacturer may have been involved **Country:** USA **Setting:** single‐centre **Trial primary outcome:** "Major end‐points for analysis included blood loss, blood transfusion requirements, and arterial and venous thromboembolic complications. Blood loss was monitored during operation according to standard techniques that included sponge weights, measurement of amounts collected in drainage canisters (total minus field irrigation with normal saline solutions), and nurses' and anesthesiologists' estimates. The operative blood loss was assessed at three time periods: (1) from skin incision to heparinization and application of aortic cross clamps, (2) the period during which aortic and vascular cross‐clamps were in place and the patients were given a full course of heparin and (3) the period from reversal of heparin with protamine sulfate to completion of operation. Blood transfusion requirements were monitored during operation and for 72 hours in the postoperative period. In addition, colloid, electrolyte solutions, and blood component therapy (fresh‐frozen plasma and platelet concentrate) were also recorded during these intervals." **Reference type:** full text **Translation into English required:** no **Trial registration number:** NR **Trial registration timing:** NA	
***Risk of bias***		
**Bias**	**Authors' judgement**	**Support for judgement**
Random sequence generation (selection bias)	Low risk	"Randomization was carried out in blocks of 10 and was stratified for repair of abdominal aortic aneurysm or aortofemoral bypass for occlusive disease. Patients were randomized by drawing a sealed envelope that contained a prescription for either 20 txg DDAVI' (Rorer Pharmaceuticals, Fort Washington, Pa.) or placebo." "Patients were randomized by drawing a sealed envelope that contained a prescription for either ...." Method of sequence generation for randomization not described.
Allocation concealment (selection bias)	Low risk	"The only person to have knowledge of treatment assignment was the pharmacist who kept records and prepared DDAVP or placebo in identical‐appearing plastic bags of 50 ml normal saline solution. DDAVP or placebo was administered intravenously for 15 minutes immediately after intravenous heparinization with 100 U/kg was performed and just before aortic cross‐clamp application was done." "Patients were randomized by drawing a sealed envelope that contained a prescription for either ...." Adequate method of central allocation concealment by pharmacy.
Blinding of participants and personnel (performance bias) All outcomes	Low risk	"The only person to have knowledge of the treatment assignment was the pharmacist who kept records and prepared DDAVP or placebo in identical ‐appearing plastic bags of 50ml normal saline solution." Subjective outcomes ‐ low risk of bias due to adequate personnel blinding. Objective outcomes ‐ low risk of bias. The article says "double blind" and a placebo was used. Subjective outcomes ‐ low risk of bias due to adequate personnel blinding. Objective outcomes ‐ low risk of bias.
Blinding of outcome assessment (detection bias) All outcomes	Low risk	"The only person to have knowledge of the treatment assignment was the pharmacist who kept records and prepared DDAVP or placebo in identical ‐appearing plastic bags of 50ml normal saline solution." Subjective outcomes ‐ low risk of bias due to adequate outcome assessor blinding. Objective outcomes ‐ low risk of bias.
Incomplete outcome data (attrition bias) All outcomes	Low risk	Outcome data set reported with denominators and numerators showing no dropouts or exclusions.
Selective reporting (reporting bias)	Unclear risk	No available prospective protocol or trial registration.
Other bias	Low risk	No other concerns such as early stopping or imbalanced study arms.

**Table:** Czerny 2000

***Study characteristics***		
Methods	**Design:** RCT (parallel group) **Anticoagulation cessation:** NR **Antiplatelet cessation:** NR **Power calculation reached:** no power calculation **Prophylactic or therapeutic randomisation:** therapeutic **Recruitment end date:** NR **Recruitment start date:** NR **Stopping rule:** NR **Strategy for analysis (ITT/PPA):** implicitly ITT but not stated and so little detail of randomisation given it is impossible to be sure **Transfusion strategy protocol:** NR **Transfusion threshold in trial:** NR **Trial stopped early:** cannot tell	
Participants	**Inclusion criteria:** "Vascular reconstruction surgery with PTFE prostheses; age of at least 18 years; existence of suture hole bleeding" **Exclusion criteria:** "Pregnant and breastfeeding women; PTFE patch longer than 8 cm; use of any kind of fibrin glue or local haemostyptic; no requirement for haemostyptic measurements; known or suspected allergies or hypersensitivities to any of the components of TachoComb H." **Group differences:** "Very large differences in type of reconstructive surgery given that this was a stratification factor for the randomisation (no details of randomisation procedure reported). Differences in condition of arterial wall, number of puncture hold bleedings, PTFE patch length and local scarring from previous operations." "There were differences between the groups concerning type and size of reconstruction as well as the condition of the artery wall. In the TCH‐group more patients underwent patch angioplasties which tend to have more needle puncture bleedings. In addition, the condition of the artery wall (extent of arteriosclerotic plaques) was worse in the TCH‐group. This, however, was balanced by larger sized grafts and patches, more cicatrisation and a higher number of operations in the anamnesis in the C‐group so that the groups were finally comparable for the extent of expected bleedings" **Indirectness:** no, all patients were vascular surgery patients **Protocol deviations:** NR **Reasons for dropouts:** NR/NA **Number of participants randomised:** NR **Number of participants receiving treatment:** NR **Number of participants analysed:** Fibrin sealant: 30; Usual care: 30 **Number of participants dropping out:** NR **Male gender (%):** Fibrin sealant: 70%; Usual care: 77% **Age (years):** Fibrin sealant: 66.4 ± 9.9; Usual care: 65.1 ± 10.7 **Weight (kg)**: Fibrin sealant: 71.5 ± 11.8; Usual care: 71.0 ± 13.9 **Ethnicity:** NR **Preoperative haemoglobin (g/L):** NR **Perioperative antiplatelet use (excluding aspirin) (%):** NR **Perioperative aspirin use (%):** NR **Perioperative anticoagulants (%):** NR **Revision surgery (%):** NR **Elective surgery (%):** 100% **Risk score (system used & score):** NR **Cross clamp use (%):** NR **Cross clamp duration (mins):** NR **Duration of surgery (mins):** Fibrin sealant: 124; Usual care: 130 **Surgical approach/es:** NR **Surgical pathology/ies:** Fibrin sealant: inguinal anastomoses 12, patch angioplasties 18; Usual care: inguinal anastomoses 17, patch angioplasties 13 **Anatomical region/s:** NR	
Interventions	**Standardised name:** Fibrin sealant: TachoComb H; Usual care: usual care **Description in text: "**Fibrin sealant: TachoComb H is a sponge‐like collagen patch coated with the components of the fibrin adhesive and is coloured yellow on the active side. A patch of 1 cm^2^ in size contains: Collagen from equine tendons 1.6 ‐ 2.6 mg Human fibrinogen 4.3 ‐ 6.7 mg Human thrombin 1.5 ‐ 2.5 IU Bovine aprotinin 0.055 ‐ 0.087 ph.Eur.U.; Usual care: surgical compresses only according to normal procedure to control suture hole bleedings."	
Outcomes	**Red cell transfusions at up to 30 days postsurgery:** NR **All‐cause mortality at up to 30 days; and at between 31 and 90 days:** NR **Risk of receiving any allogeneic blood product (composite) at up to 30 days postsurgery :** Fibrin sealant: 4/30 ; Usual care: 12/30 **Risk of receiving any allogeneic packed red cells at up to 30 days postsurgery:** NR **Risk of receiving any allogeneic platelets at up to 30 days postsurgery:** NR **Risk of receiving any allogeneic fresh frozen plasma at up to 30 days postsurgery:** NR **Risk of reoperation or repeat procedure for bleeding within 7 days:** NR **Risk of a thrombotic/thromboembolic event (any composite measured in study) (n/N): 30 days:** Fibrin sealant: 0/30; Usual care: 3/30 **Up to 90 days:** Fibrin sealant: 2/30; Usual care: 3/30 **Risk of cerebrovascular attack (CVA/stroke) at up to 30 days:** NR **Risk of myocardial infarction (MI) at up to 30 days:** NR **Risk of deep vein thrombosis (DVT) at up to 90 days:** NR **Risk of pulmonary embolus (PE) at up to 90 days:** NR **Risk of a serious adverse event (SAE) at up to 30 days:** NR **Length of hospital stay (days):** Fibrin sealant: mean 10; Usual care: 10.5 **Cost data presented:** no	
Notes	**Sponsorship source:** NR, appears to be pharmaceutical Nycomed Pharma AG. Two authors were employed by the manufacturer and randomisation was done by the sponsor with no further information given. **Country:** Germany, Austria **Setting:** multicentre **Trial primary outcome:** time to haemostasis (period from release of the cross‐clamping to the moment the wound was completely dry or at least sufficiently dry to finalise the operation without additional measures for haemostasis) as assessed by investigators. Secondary endpoints were intraoperative blood loss (measured by weighing the swabs used only in the application area and used from the time TachoComb H/compresses were applied until haemostasis), intraoperative blood substitutes, duration of the operation, duration of the drainage and total drainage volume as well as intraoperative efficacy rating of the study treatment evaluated by the surgeon. All adverse events were recorded from the time the patient signed the informed consent up to 3 months following discharge. The adverse events were divided into two groups 'non‐serious' and 'serious' according to ICH‐GCP criteria. **Reference type:** full text **Translation into English required:** no **Trial registration number:** NA **Trial registration timing:** NA	
***Risk of bias***		
**Bias**	**Authors' judgement**	**Support for judgement**
Random sequence generation (selection bias)	High risk	No meaningful information. "The randomisation was carried out by the sponsor and patients were stratified for the type of reconstruction (anastomosis or patch angioplasty) as the bleeding from patch angioplasty was supposed to be more severe because of longer suture lines."
Allocation concealment (selection bias)	High risk	No information. No details of allocation concealment were available.
Blinding of participants and personnel (performance bias) All outcomes	High risk	The study was unblinded. For objective outcomes (e.g. mortality) the RoB is low.
Blinding of outcome assessment (detection bias) All outcomes	High risk	The study was unblinded. For objective outcomes, the RoB is low.
Incomplete outcome data (attrition bias) All outcomes	Unclear risk	The number of participants randomised was not reported. Unclear if all participants who were randomised were included in the final analysis.
Selective reporting (reporting bias)	High risk	The trial was not preregistered, and no protocol is available, so it is unclear if the investigators collected more data that is not reported here. All the outcomes stated in the methods section were reported in the paper. Methods stated that AEs were classified as serious/not, but there was no reporting of SAEs, only all AEs. Eligibility criteria require bleeding (so presumably intraoperative randomisation, but no information reported about randomisation procedures) but a preoperative adverse event was reported. No sample sizes given for any outcome.
Other bias	Low risk	No additional concerns noted

**Table:** EUCTR2016‐003661‐26‐PL

***Study characteristics***		
Methods	**Design:** RCT (parallel group) **Anticoagulation cessation:** exclusion criteria: "Vascular surgery: Subject is taking dual anti‐platelet treatment or oral anticoagulants within 7 days of surgery. One anti‐platelet agent is allowed perioperatively." **Antiplatelet cessation:** exclusion criteria: "Vascular surgery: Subject is taking dual anti‐platelet treatment or oral anticoagulants within 7 days of surgery. One anti‐platelet agent is allowed perioperatively." **Power calculation reached:** none given (trial registration) **Prophylactic or therapeutic randomisation:** prophylactic (randomisation appears to have occurred before surgery) **Recruitment end date:** 19 July 2017 **Recruitment start date:** 31 March 2017 **Stopping rule:** none stated (trial registration) **Strategy for analysis (ITT/PPA):** per protocol. " Full Analysis Set represents the planned treatments for patients undergoing all surgeries who received [allocated treatment] and experienced mild bleeding during surgery" **Transfusion strategy protocol:** NR **Transfusion threshold in trial:** NR **Trial stopped early:** yes, "240 subjects planned; 169 subjects analysed"	
Participants	**Inclusion criteria:** "Subject is undergoing a planned open liver/soft tissue surgery, vascular surgery or spine surgery. Subjects are able and willing to provide written informed consent to participate in this study. Adult males and females ≥ 18 years of age at screening. Willing and able to comply with all protocol requirements including follow‐up assessments. Male subjects must be willing and able to use adequate contraception from enrollment through to the 30 day follow‐up visit. Women of childbearing potential (WCBP)C have to use highly effective methods of contraception from enrollment through to the 30 day follow‐up visit. Intraoperative: The subject presents an identified target bleeding site with mild or moderate bleeding, which conventional surgical techniques are insufficient to control or are inappropriate and would otherwise be a candidate for standard haemostats. The subject presents no intraoperative complications, other than bleeding, that may interfere with study assessments as judged by the Investigator. The subject presents no contaminated areas of the body, signs of infection or abscess development. Total target bleeding site surface area of ≤ 70 cm^2^, defined within one or two TBSs." **Exclusion criteria:** "Subject is undergoing emergency surgical procedure. Use of study treatment and sponge in closure of skin incisions as the sponge may interfere with the healing of skin edges. Intravascular compartments because of the risk of embolization following sponge application. Recipient of an organ transplant. Haematologic, biochemistry and coagulation panel thresholds at screening: Haemoglobin ≤ 9.0 g/dL. Platelet count ≤ 100,000/mm^3^ (≤ 100 x 109/L). International Normalized Ratio (INR) > 2.0 or activated Partial Thromboplastin Time (aPTT) ratio > 2.0. Fibrinogen level < 1.5 g/L. Aspartate Aminotransferase (AST) or Alanine aminotransferase (ALT) ≥ 3 times the upper limit normal range, except for subjects undergoing liver resection surgery where there is no upper limit for these analytes due to the nature of their disease. Severe renal failure. Any other disease or condition that may affect normal blood clotting, for example thrombocytopenia, as judged by the Investigator. A known history of anaphylaxis or allergic reaction to human albumin, PEGylated proteins, yeast or moulds, porcine products or other components in the study medication or sponge. Participation in another investigational drug or device research study within 30 days before and after enrolment in the current study. Current known or suspected alcohol and/or drug abuse or dependence at the time of screening. Any concurrent medical, surgical, or psychiatric condition that may, in the Investigator's opinion, affect the subject's willingness or ability to meet all study requirements during the study duration. Known HIV, Hepatitis B virus or Hepatitis C Virus infection. During the surgery, subject presents severe bleeding where use of a topical haemostat would be inappropriate. Anti‐platelets/oral anticoagulants treatment: Soft tissue/liver and neurosurgery: Subject is taking anti‐platelet agents or oral anticoagulants within 7 days of surgery, Vascular surgery: Subject is taking dual anti‐platelet treatment or oral anticoagulants within 7 days of surgery. One anti‐platelet agent is allowed perioperatively. Heparin treatment: c. Soft tissue/liver and neurosurgery only: Subject is receiving therapeutic doses of heparin perioperatively. Only prophylactic Low Molecular Weight Heparin is allowed. Pregnant or breast‐feeding subject." **Group differences:** greater proportion of men in the vascular intervention group (6 female, 29 male verus 6 female, 12 male) **Indirectness:** no (vascular subgroup reported) **Protocol deviations:** whole population: "A total of 214 subjects were consented and screened, 203 subjects were randomised and 169 received treatment. 11 subjects were screen failures and 34 were extended screen failures (did not require a haemostat during their surgery)." No information for vascular subgroup. **Reasons for dropouts:** Unclear. Number randomised NR **Number of participants randomised:** NR **Number of participants receiving treatment:** Peprostat: 36; Saline: 18 **Number of participants analysed:** Peprostat: 36; Saline: 18 **Number of participants dropping out:** NR **Male gender (%):** Peprostat: 83%; Saline: 67% **Age (years):** Peprostat: mean 68.2 SD 9.07; Saline: mean 68.0 SD 9.60 **Weight (kg):** NR **Ethnicity:** NR **Preoperative haemoglobin (g/L):** NR **Perioperative antiplatelet use (excluding aspirin) (%):** NR **Perioperative aspirin use (%):** NR **Perioperative anticoagulants (%):** NR **Revision surgery (%):** NR **Elective surgery (%):** 100% (eligibility criteria) **Risk score (system used & score):** NR **Cross clamp use (%):** NR **Cross clamp duration (mins):** NR **Duration of surgery (mins):** NR **Surgical approach/es:** NR **Surgical pathology/ies:** NR **Anatomical region/s:** NR	
Interventions	**Standardised name:** Peprostat: Peprostat soaked gelatin sponge; Saline: Saline soaked gelatin sponge **Description in text:** Peprostat: "The gelatine sponge was soaked with 1 vial; 5 mL 2.5 mg/mL PeproStat (12.5 mg nominal dose), moments before topical application to the target bleeding site during scheduled liver/soft tissue, vascular, or spine surgery. A maximum of 2 PeproStat soaked sponges could be used in each subject as appropriate for the size / number of bleeding sites."; Saline: "The gelatine sponge was soaked with 1 vial; 5 mL of 0.9% saline, moments before topical application to the target bleeding site during scheduled liver/soft tissue, vascular, or spine surgery. A maximum of 2 saline soaked sponges could be used in each subject as appropriate for the size / number of bleeding sites."	
Outcomes	**Red cell transfusions at up to 30 days postsurgery:** NR **All‐cause mortality at up to 30 days;** Peprostat: 0/36; Saline: 0/18 **and at between 31 and 90 days:** NR **Risk of receiving any allogeneic blood product (composite) at up to 30 days postsurgery:** NR **Risk of receiving any allogeneic packed red cells at up to 30 days postsurgery:** NR **Risk of receiving any allogeneic platelets at up to 30 days postsurgery:** NR **Risk of receiving any allogeneic fresh frozen plasma at up to 30 days postsurgery:** NR **Risk of reoperation or repeat procedure for bleeding within 7 days:** NR **Risk of a thrombotic/thromboembolic event (any composite measured in study) (n/N):** NR **Risk of cerebrovascular attack (CVA/stroke) at up to 30 days:** NR **Risk of myocardial infarction (MI) at up to 30 days:** NR **Risk of deep vein thrombosis (DVT) at up to 90 days:** NR **Risk of pulmonary embolus (PE) at up to 90 days:** Peprostat: 0/36; Saline: 0/18 **Risk of a serious adverse event (SAE) at up to 30 days:** Peprostat: 6/36; Saline: 1/18 **Length of hospital stay (days):** NR **Cost data presented:** no	
Notes	**Sponsorship source:** Pharmaceutical ‐ Haemostatix Limited. Full declarations of author COI are not available in the trial registration. **Country:** Bosnia and Herzegovina, Croatia, Poland, United Kingdom, Serbia **Setting:** multicentre (5) **Trial primary outcome:** "The primary endpoint of the study was the efficacy in terms of Time To Haemostasis (TTH) at the primary Target Bleeding Site (TBS)..."all of the secondary parameters were analysed using descriptive and exploratory statistics." "Efficacy: 1. Time to haemostasis 2. Number and amount of haemostatic sponge used 3. Use of alternate haemostatic agents at the TBS 4. Investigator efficacy assessment: global score for efficacy to obtain haemostasis and global score for ease of use of study treatment Safety: 1. Adverse events 2. Clinical laboratory assessments 3. Physical examination 4. Vital signs 5. Pulse oximetry 6. Electrocardiogram 7. Concomitant medications" **Reference type:** trial registration with results **Translation into English required:** no **Trial registration number:** EUDRACT2016‐003661‐26; NCT03131336 **Trial registration timing:** retrospective but within one month of recruitment starting	
***Risk of bias***		
**Bias**	**Authors' judgement**	**Support for judgement**
Random sequence generation (selection bias)	Unclear risk	Only trial registration and results were available to extract. No details of randomisation processes were provided.
Allocation concealment (selection bias)	Unclear risk	Only trial registration and results were available to extract. No details of allocation concealment processes were provided.
Blinding of participants and personnel (performance bias) All outcomes	Low risk	Trial is described as "double blind," with subject, investigator, monitor and data analyst all blinded. No details available on how blinding was carried out, as only trial registration and results are provided.
Blinding of outcome assessment (detection bias) All outcomes	Low risk	Trial is described as "double blind," with subject, investigator, monitor and data analyst all blinded. No details available on how blinding was carried out, as only trial registration and results are provided.
Incomplete outcome data (attrition bias) All outcomes	High risk	Very large number excluded due to not receiving study intervention; randomisation appears to have taken place before surgery, but exclusion from analysis includes not receiving allocated intervention, not just absence of an eligible bleed. "Full Analysis Set represents the planned treatments for patients undergoing all surgeries who received [allocated treatment] and experienced mild bleeding during surgery."
Selective reporting (reporting bias)	Low risk	The primary outcomes were reported in the trial registration, but secondary outcomes were not reported, likely because of lack of space. Adverse event reporting was complete.
Other bias	Low risk	No additional concerns noted.

**Table:** Giovanacci 2002

***Study characteristics***		
Methods	**Design:** RCT (parallel group) **Anticoagulation cessation:** NR **Antiplatelet cessation:** NR **Power calculation reached:** "For an estimated rate of lymphatic complications of 20% a sample size of 108 wounds was required to detect as halving as lymphatic complications (alpha 0.5 and power 80%)" **Prophylactic or therapeutic randomisation:** unclear, randomisation done in the operating theatre, but eligibility criteria are vague. **Recruitment end date:** January 2001 **Recruitment start date:** "over 2.5 years ending in January 2001" implies July 1998. **Stopping rule:** NR **Strategy for analysis (ITT/PPA):** not stated; one cross‐over was excluded, but six participants in each group were lost to follow‐up and not reported despite having intrasurgical results. **Transfusion strategy protocol:** NR **Transfusion threshold in trial:** NR **Trial stopped early:** no	
Participants	**Inclusion criteria:** all patients over the age of 20 years undergoing exposure of the femoral artery **Exclusion criteria:** NR **Group differences:** few baseline characteristics reported (with bilateral group reported separately). More men on control (56% vs 51%), more "urgent" operations on intervention (27% vs 18%) and fewer "redo" operations (9% vs 16%). **Indirectness:** no **Protocol deviations:** one participant in the control group received fibrin glue when they required revision surgery **Reasons for dropouts:** Fibrin sealant: (138 wounds, including the group receiving both intervention and control procedures), 6 cases lost to follow‐up (3 death < 1 week, 3 missing data). Usual care: (141 wounds including the group receiving both intervention and control procedures), 6 cases lost to follow‐up (3 deaths < 1 week, 2 patients transferred out of hospital, 1 missing data). [Cases may mean wounds rather than patients as the totals do not add up.] **Number of participants randomised:** NR **Number of participants receiving treatment:** NR **Number of participants analysed:** Fibrin sealant: 79; Usual care: 81 **Number of participants dropping out:** Fibrin sealant: 6; Usual care: 6 **Male gender (%):** Fibrin sealant: 51%; Usual care: 56% **Age (years):** Fibrin sealant: mean 70; Usual care: mean 72 **Weight (kg):** NR **Ethnicity:** NR **Preoperative haemoglobin (g/L):** NR **Perioperative antiplatelet use (excluding aspirin) (%):** NR **Perioperative aspirin use (%):** NR **Perioperative anticoagulants (%):** NR **Revision surgery (%):** Fibrin sealant: 9%; Usual care: 16% **Elective surgery (%):** Fibrin sealant: NR but Table 1 in publication implies 64.6% if no revision surgeries were also urgent; Usual care: NR but Table 1 in publication implies 66.7% if no revision surgeries were also urgent. **Risk score (system used & score):** NR **Cross clamp use (%):** NR **Cross clamp duration (mins):** NR **Duration of surgery (mins):** NR **Surgical approach/es:** inguinal access to femoral artery 100% **Surgical pathology/ies:** Fibrin sealant: aneurysm 9 (11%), chronic occlusive disease 50 (63%); Usual care: aneurysm 11 (27%); chronic occlusive disease 51 (63%); aneurysm and chronic occlusive disease 1 (1%) **Anatomical region/s:** inguinal access 100%	
Interventions	**Standardised name:** Fibrin sealant: fibrin glue; Usual care: usual care **Description in text:** Fibrin sealant: "1 ml fibrin glue (Tissucol1; Baxter AG, Volketswil). The fibrin glue was spread with a syringe on the largest possible surface of the deep subcutaneous tissue and of the fascia"; Usual care: "The wounds of group A were closed without fibrin glue."	
Outcomes	**Red cell transfusions at up to 30 days postsurgery:** NR **All‐cause mortality at up to 30 days; and at between 31 and 90 days:** NR **Risk of receiving any allogeneic blood product (composite) at up to 30 days postsurgery:** NR **Risk of receiving any allogeneic packed red cells at up to 30 days postsurgery:** NR **Risk of receiving any allogeneic platelets at up to 30 days postsurgery:** NR **Risk of receiving any allogeneic fresh frozen plasma at up to 30 days postsurgery:** NR **Risk of reoperation or repeat procedure for bleeding within 7 days :** Fibrin sealant: 5/79; Usual care: 5/81 **Risk of a thrombotic/thromboembolic event (any composite measured in study) (n/N):** NR **Risk of cerebrovascular attack (CVA/stroke) at up to 30 days:** NR **Risk of myocardial infarction (MI) at up to 30 days:** NR **Risk of deep vein thrombosis (DVT) at up to 90 days:** NR **Risk of pulmonary embolus (PE) at up to 90 days:** NR **Risk of a serious adverse event (SAE) at up to 30 days:** NR **Length of hospital stay (days):** NR **Cost data presented:** no	
Notes	**Sponsorship source:** "This study was carried out with the financial support of Baxter AG, Switzerland." No statement of independence of authors. Unclear how much of the trial was managed by the sponsor. **Country:** Switzerland **Setting:** Not stated but presumably vascular surgery departments (3 listed for the authors) **Trial primary outcome:** "The incidence of lymphatic and non‐lymphatic complications, the amount and gross appearance of fluid collected in the drain and the time to drain removal were recorded prospectively. Lymphorrhoea was defined as perfusion of clear lymphatic fluid at the operative wound site. Lymphatic fistula was defined as the production of more than 30 ml clear fluid per day at the operative wound site lasting more than 3 days following the procedure, or persistent lymphatic leakage for more than 5 days after the procedure regardless of the amount of fluid. Lymphocele was defined as subcutaneous collection of clear fluid without infection or haematoma. If a lymphocele was suspected the groin was examined using ultrasound." **Reference type:** full text **Translation into English required:** no (but there are some language barriers in the reporting) **Trial registration number:** none **Trial registration timing:** NA	
***Risk of bias***		
**Bias**	**Authors' judgement**	**Support for judgement**
Random sequence generation (selection bias)	High risk	No information. Study randomised wounds rather than people.
Allocation concealment (selection bias)	High risk	"For randomisation we had two containers with numbered closed envelopes in the operating room, one for unilateral and one for bilateral procedures. For unilateral operation the envelope contained the instruction if fibrin glue had to be utilised or not. For bilateral operations the envelope contained the instruction about which inguinal wound had to be sealed with fibrin glue. The contralateral wound was assigned to the control group." Unclear how "which inguinal wound" was described in the randomisation and whether surgeon would have the freedom to choose (i.e. if randomisation was first wound vs second wound). No mention of whether envelopes were opaque or audit of order in which envelopes were used.
Blinding of participants and personnel (performance bias) All outcomes	High risk	No blinding reported. Surgical team would not be able to be blinded. For objective outcomes, this domain is low RoB.
Blinding of outcome assessment (detection bias) All outcomes	High risk	No blinding reported. Surgical team would not be able to be blinded; no mention of blinded outcome assessors. For objective outcomes, this domain is low RoB.
Incomplete outcome data (attrition bias) All outcomes	Unclear risk	Six people lost to follow‐up on each arm and one cross‐over on control. 6 vs 7 wounds excluded as a result. Unclear why intrasurgical outcomes not available for these people. Sample sizes not reported but appear to be 136 wounds on control and 130 on intervention. Results keep switching between wounds and people without much clarity.
Selective reporting (reporting bias)	Unclear risk	No trial registration. Protocol is not available. It is unclear if more outcome data were recorded and not reported in the paper.
Other bias	Low risk	No other concerns noted.

**Table:** Joseph 2004

***Study characteristics***		
Methods	**Design:** RCT (parallel group) **Anticoagulation cessation:** NR **Antiplatelet cessation:** NR **Power calculation reached:** no, 24 enrolled (12 per arm) though power calculation planned for 60 (30 per arm) **Prophylactic or therapeutic randomisation:** prophylactic **Recruitment end date:** NR **Recruitment start date:** NR **Stopping rule:** NR **Strategy for analysis (ITT/PPA):** per protocol analysis, "Twenty four patients were randomised to give 12 patients in each treatment group of the study. One patient was withdrawn immediately after randomisation (surgical compression group), and one patient had no assessment of time to haemostasis (Tacho‐Comb H group)." "One patient was excluded prior to randomisation and two more had to be withdrawn because of deviation from study protocol and insufficient data collection, giving a total of 22 patients." **Transfusion strategy protocol:** NR **Transfusion threshold in trial:** NA **Trial stopped early:** yes, "A total sample size of 60 patients (30 in each treatment group) was originally planned after a power calculation but as the effectiveness of TachoComb H was also demonstrated in a similar study, conducted simultaneously, the present study was stopped prematurely and only 24 patients were randomised."	
Participants	**Inclusion criteria**: people undergoing femoral anastomosis and femoral or carotid patch angioplasty with PTFE grafts **Exclusion criteria:** "Patients were excluded if the patch length exceeded 8 cm, if fibrin glues or any local haemostyptics were used during the operation or if the patient was allergic to any of the components of TachoComb H. Patients who did not require additional haemostatic measures on releasing the clamps were also excluded from the study." **Group differences:** unclear as few measured baseline characteristics are presented in the publication, but authors report there were none: "The mean age of patients was 70.6 (range 55 – 86) years in the TachoComb H group and 66.3 (range 51 – 71) years in the surgical control group. There were no significant differences in any patient or procedural characteristics including the use of anti‐platelet agents." **Indirectness:** no **Protocol deviations:** NA, none **Reasons for dropouts:** There were 2 dropouts. This left 11 out of 12 participants in each group for analysis. "Twenty four patients were randomised to give 12 patients in each treatment group of the study. One patient was withdrawn immediately after randomisation (surgical compression group), and one patient had no assessment of time to haemostasis (Tacho‐Comb H group)." **Number of participants randomised:** Tacho‐Comb H: 12; Usual care: 12 **Number of participants receiving treatment:** Tacho‐Comb H: 12; Usual care: 11 **Number of participants analysed:** Tacho‐Comb H: 11; Usual care: 11 **Number of participants dropping out:** Tacho‐Comb H: 1; Usual care: 1 **Male gender (%):** NR **Age (years):** Tacho‐Comb H: mean 70.6 range 55‐86; Usual care: mean 66.3 range 51‐71 **Weight (kg):** NR **Ethnicity:** NR **Preoperative haemoglobin (g/L):** NR **Perioperative antiplatelet use (excluding aspirin) (%):** NR **Perioperative aspirin use (%):** NR **Perioperative anticoagulants (%):** NR **Revision surgery (%):** NR **Elective surgery (%):** NR **Risk score (system used & score):** NR **Cross clamp use (%):** 100% **Cross clamp duration (mins):** NR **Duration of surgery (mins):** Tacho‐Comb H: mean 81 range 51‐110; Usual care: mean 83 range 50 to 144 **Surgical approach/es:** open **Surgical pathology/ies:** anastomoses, angioplasty **Anatomical region/s:** femoral, carotid	
Interventions	**Standardised name:** Tacho‐Comb H: TachoComb H (collagen sponge coated with thrombin/fibrin); Usual care: usual care (standard compression with surgical swabs) **Description in text:** Tacho‐Comb H: "Patients were randomised by the sealed code envelope method to either treatment with TachoCombwH patches or to standard compression with surgical swabs; Usual care: "Patients in the control arm received compression with 10 x 10 cm^2^ surgical swabs. If haemostasis was suboptimal at the end of 10 min then other methods were adopted as necessary."	
Outcomes	**Red cell transfusions at up to 30 days postsurgery:** NR **All‐cause mortality at up to 30 days:** Tacho‐Comb H: 1/11; Usual care: 0/11 **and at between 31 and 90 days:** NR **Risk of receiving any allogeneic blood product (composite) at up to 30 days postsurgery:** Tacho‐Comb H: 0/11; Usual care: 2/11 **Risk of receiving any allogeneic packed red cells at up to 30 days postsurgery:** NR **Risk of receiving any allogeneic platelets at up to 30 days postsurgery:** NR **Risk of receiving any allogeneic fresh frozen plasma at up to 30 days postsurgery:** NR **Risk of reoperation or repeat procedure for bleeding within 7 days:** NR **Risk of a thrombotic/thromboembolic event (any composite measured in study) (n/N):** Tacho‐Comb H: 0/11; Usual care: 0/11 **Risk of cerebrovascular attack (CVA/stroke) at up to 30 days:** NR **Risk of myocardial infarction (MI) at up to 30 days:** NR **Risk of deep vein thrombosis (DVT) at up to 90 days:** NR **Risk of pulmonary embolus (PE) at up to 90 days:** NR **Risk of a serious adverse event (SAE) at up to 30 days:** Tacho‐Comb H: 2/11; Usual care: 2/11 **Length of hospital stay (days):** NR **Cost data presented:** no	
Notes	**Sponsorship source:** pharmaceutical (Nycomed). No statement of independence of authors. Unclear why the trial was stopped early but may have been sponsor‐influenced. **Country:** UK **Setting:** multicentre (3) **Trial Primary outcome:** "Primary outcome was the time to haemostasis. Haemostasis was defined as the time when the wound was completely dry or sufficiently dry to complete the operation without additional haemostasis. Secondary outcomes: Blood loss during the operation, duration of operation, drain volume, requirement for blood transfusion, surgeons rating of efficacy (very good, good, satisfactory or unsatisfactory), and coagulation parameters were also recorded." **Reference type:** full text **Translation into English required:** no **Trial registration number:** NR **Trial registration timing:** NA	
***Risk of bias***		
**Bias**	**Authors' judgement**	**Support for judgement**
Random sequence generation (selection bias)	Unclear risk	"Patients were randomised by the sealed code envelope method to either treatment with TachoComb w H patches or to standard compression with surgical swabs." Method of sequence generation for randomisation not described.
Allocation concealment (selection bias)	Unclear risk	"Patients were randomised by the sealed code envelope method to either treatment with TachoComb w H patches or to standard compression with surgical swabs." Method of allocation concealment not described fully enough. Envelopes not described as sealed, opaque and sequentially numbered.
Blinding of participants and personnel (performance bias) All outcomes	High risk	"The nature of the treatments precluded blinding of the study." Study not blinded. Subjective outcomes ‐ high risk of bias due to unblinded personnel blinding. Objective outcomes ‐ low risk of bias regardless.
Blinding of outcome assessment (detection bias) All outcomes	Unclear risk	"The nature of the treatments precluded blinding of the study. Therefore, an independent nurse recorded all the variables assessed." Study was unblinded however, nurse is described as independent so presumable was unaware of treatment allocation.
Incomplete outcome data (attrition bias) All outcomes	Low risk	Whilst two participants were excluded from analysis due to deviation from study protocol it is unlikely to have introduced bias, because the exclusions in both arms were balanced.
Selective reporting (reporting bias)	Low risk	All outcomes planned in the protocol or prospective trial registration were reported.
Other bias	High risk	"There were no significant differences in any patient or procedural characteristics including the use of anti‐platelet agents." "A total sample size of 60 patients (30 in each treatment group) was originally planned after a power calculation but as the effectiveness of TachoComb w H was also demonstrated in a similar study, conducted simultaneously, the present study was stopped prematurely and only 24 patients were randomised." Study stopped early for ethical reasons (futility) as a different study showed clear efficacy of TachoComb. Although the text in the results suggested that there were no significant differences in the baseline characteristics, there was no table with data backing up this assertion.

**Table:** Leijdekkers 2006

***Study characteristics***		
Methods	**Design:** RCT (parallel group) **Anticoagulation cessation:** Use of anticoagulant drugs prior to surgery was an exclusion criterion. All patients had heparin 1 mg/kg prior to aortic cross clamping. **Antiplatelet cessation:** Use of platelet aggregation inhibitors prior to surgery as an exclusion criterion. **Power calculation reached:** yes, "35 patients would be necessary to show a significant difference in the amount of blood loss (40% reduction with an alpha of 0.05 and beta of 0.2 and suspected S.D of around 800ml)" **Prophylactic or therapeutic randomisation:** prophylactic **Recruitment end date:** July 2001 **Recruitment start date:** June 1996 **Stopping rule:** NR **Strategy for analysis (ITT/PPA):** ITT **Transfusion strategy protocol:** yes **Transfusion threshold in trial:** haemoglobin (< 96.7 g/L) **Trial stopped early:** no	
Participants	**Inclusion criteria:** "Consecutive patients with an asymptomatic infrarenal aortic aneurysm, in who the operation could be performed by infrarenal cross‐clamping of the aorta in insertion of either an aortic tube or bifurcation prosthesis were evaluated for inclusion." **Exclusion criteria:** "Patients were excluded if they had liver dysfunction, coagulation disorders or history of anticoagulant use (e.g. warfarin and platelet aggregation inhibitors) prior to the operation. Patients were also excluded if they had received aprotinin during previous surgery." **Group differences:** "No significant differences between study groups" for all baseline variables measured (age, gender, aortic diameter, cardiac or pulmonary comorbidity or procedure). **Indirectness:** no **Protocol deviations:** NA **Reasons for dropouts:** NA **Number of participants randomised:** Aprotinin: 16; Placebo: 16 **Number of participants receiving treatment:** Aprotinin: 16; Placebo: 16 **Number of participants analysed:** Aprotinin: 16; Placebo: 16 **Number of participants dropping out:** 0 **Male gender (%):** Aprotinin: 88%; Placebo: 74% **Age (years):** Aprotinin: mean 68 SD 9.5; Placebo: mean 68 SD 6.8 **Weight (kg):** NR **Ethnicity:** NR **Preoperative haemoglobin (g/L):** Aprotinin: mean 127.3 SD 16.1; Placebo: mean 120.9 16.1 **Perioperative antiplatelet use (excluding aspirin) (%):** 0 **Perioperative aspirin use (%):** 0 **Perioperative anticoagulants (%):** 0 **Revision surgery (%):** NR **Elective surgery (%):** 100% **Risk score (system used & score):** NR **Cross clamp use (%):** 100% **Cross clamp duration (mins):** Aprotinin: mean 78 SD 71; Placebo: mean 70 SD 29 **Duration of surgery (mins):** Aprotinin: mean 195 SD 98; Placebo: mean 190 SD 87 **Surgical approach/es:** open **Surgical pathology/ies:** aortic aneurysm **Anatomical region/s:** infra‐renal	
Interventions	**Standardised name:** Aprotinin: aprotinin 2 MK iv bolus to patient over 15 minutes plus aprotinin 0.5 MK/hr iv infusion to patient continuously (to a maximum of 2 MK); Placebo: placebo NS EV **Description in text:** NA	
Outcomes	**Red cell transfusions at up to 30 days postsurgery:** Aprotinin: mean 4.1 SD 3.1 n = 16; Placebo: mean 4.1 SD 2.9 n = 19 **All‐cause mortality at up to 30 days:** Aprotinin: 1/16; Placebo: 1/19 **and at between 31 and 90 days:** NR **Risk of receiving any allogeneic blood product (composite) at up to 30 days postsurgery:** NR **Risk of receiving any allogeneic packed red cells at up to 30 days postsurgery:** NR **Risk of receiving any allogeneic platelets at up to 30 days postsurgery:** Aprotinin: 1/16; Placebo: 1/19 **Risk of receiving any allogeneic fresh frozen plasma at up to 30 days postsurgery:** NR **Risk of reoperation or repeat procedure for bleeding within 7 days:** Aprotinin: 1/16; Placebo: 2/19 **Risk of a thrombotic/thromboembolic event (any composite measured in study) (n/N):** NR **Risk of cerebrovascular attack (CVA/stroke) at up to 30 days:** NR **Risk of myocardial infarction (MI) at up to 30 days:** NR **Risk of deep vein thrombosis (DVT) at up to 90 days:** NR **Risk of pulmonary embolus (PE) at up to 90 days:** NR **Risk of a serious adverse event (SAE) at up to 30 days:** NR **Length of hospital stay (days):** NR **Cost data presented:** no	
Notes	**Sponsorship source:** pharmaceutical (Bayer). No statement of independence of authors. **Country:** The Netherlands **Setting:** single‐centre **Trial Primary outcome:** "Primary end points were blood loss and the necessity for red cell infusions. Secondary end points were the duration of stay on ICU, duration of artificial respiration, amount of complications and consumption of coagulation factors" **Reference type:** full‐text, abstract **Translation into English required:** no **Trial registration number:** NR **Trial registration timing:** NA	
***Risk of bias***		
**Bias**	**Authors' judgement**	**Support for judgement**
Random sequence generation (selection bias)	Low risk	"Patients were randomized to receive either placebo or aprotinin, using a standard randomization list stored in the pharmacy department, only to be opened after the study was closed for inclusion." Adequate method of sequence generation with standard randomisation list.
Allocation concealment (selection bias)	Low risk	Allocation concealment performed at the pharmacy, though no further details given of how patient's group allocation was concealed.
Blinding of participants and personnel (performance bias) All outcomes	Low risk	"A double‐blind randomized trial...." "Aprotinin was supplied in bottles of 50ml... the placebo was supplied in the same bottles containing only NaCl..." Subjective outcomes ‐ low risk of bias due to adequate personnel blinding. Objective outcomes ‐ low risk of bias.
Blinding of outcome assessment (detection bias) All outcomes	Low risk	Randomisation list was only opened after study completion. Subjective outcomes ‐ low risk of bias due to adequate outcome assessor blinding. Objective outcomes ‐ low risk of bias.
Incomplete outcome data (attrition bias) All outcomes	Low risk	Outcome data set reported with denominators and numerators and with no dropouts reported.
Selective reporting (reporting bias)	Unclear risk	No available prospective protocol or trial registration.
Other bias	Low risk	No other concerns noted.

**Table:** Lethagen 1991

***Study characteristics***		
Methods	**Design:** RCT (parallel group) **Anticoagulation cessation:** NR **Antiplatelet cessation:** yes, aspirin cessation. "the use of acetyl salicylic acid within 10 days prior to surgery were excluded". **Power calculation reached:** no power calculation reported **Prophylactic or therapeutic randomisation:** prophylactic **Recruitment end date:** NR **Recruitment start date:** NR **Stopping rule:** NR **Strategy for analysis (ITT/PPA):** per protocol analysis. "six patients in whom surgical complications significantly contributed to blood loss and transfusion requirements were identified and excluded." **Transfusion strategy protocol:** yes **Transfusion threshold in trial:** haematocrit (< 30 to 35) **Trial stopped early:** no	
Participants	**Inclusion criteria:** "Fifty patients scheduled for surgery for aorto‐iliac occlusive disease or aneurysms were randomly allocated to receive either desmopressin or a placebo. " "The operations were performed through an intra‐ peritoneal approach." **Exclusion criteria:** "Patients with a history of increased bleeding tendency, prolonged preoperative bleeding time or the use of acetyl salicylic acid within 10 days prior to surgery were excluded." **Group differences:** "The two groups were well matched concerning background factors and operative procedures." (Gender, surgical indication, surgery type, surgical duration) **Indirectness:** no **Protocol deviations:** NA **Reasons for dropouts:** "Before breaking the code, six patients in whom surgical complications significantly contributed to blood loss and transfusion requirements were identified and excluded." "The exclusion of these six patients did not change the conclusions of the study. Forty‐four patients were valid for analysis, 22 in the desmopressin group and 22 in the placebo group." **Number of participants randomised:** Desmopressin: 25; Placebo: 25 **Number of participants receiving treatment:** Desmopressin: 25; Placebo: 25 **Number of participants analysed:** Desmopressin: 22; Placebo: 22 **Number of participants dropping out:** Desmopressin: 3; Placebo: 3 **Male gender (%):** Desmopressin: 76%; Placebo: 72% **Age (years):** NR **Weight (kg):** NR **Ethnicity:** NR **Preoperative haemoglobin (g/L):** NR **Perioperative antiplatelet use (excluding aspirin) (%):** 0 **Perioperative aspirin use (%):** 0 **Perioperative anticoagulants (%):** NR **Revision surgery (%):** 0 **Elective surgery (%):** 100% **Risk score (system used & score):** NR **Cross clamp use (%):** NR **Cross clamp duration (mins):** NR **Duration of surgery (mins):** Desmopressin: mean 197 SD 62; Placebo: mean 215 SD 93 **Surgical approach/es:** open **Surgical pathology/ies:** Desmopressin: aortic aneurysm 52%. Occlusive disease 48%; Placebo: aneurysm repair elective (52%), bypass elective (44%), repair of coarction (4%) **Anatomical region/s:** aorto‐iliac	
Interventions	**Standardised name:** Desmopressin: desmopressin 0.3 mcg/kg intravenous infusion over 10 minutes at induction; Placebo: placebo NS EV **Description in text:** Desmopressin: "Desmopressin 4 μg/ml (Minirin ®, Ferring, Sweden) was diluted in physiological saline to a volume of 10 ml and injected intravenously over 10min at a dose of 0.3 μg/kg body weight and placebo given as physiological saline. Study drugs were administered immediately before the start of the operation"; Placebo: placebo NS EV	
Outcomes	**Red cell transfusions at up to 30 days postsurgery:** Desmopressin: mean 3.76 SD 0.525, n = 22; Placebo: mean 4.235 SD 0.636, n = 22 **All‐cause mortality at up to 30 days; and at between 31 and 90 days:** NR **Risk of receiving any allogeneic blood product (composite) at up to 30 days postsurgery:** NR **Risk of receiving any allogeneic packed red cells at up to 30 days postsurgery:** NR **Risk of receiving any allogeneic platelets at up to 30 days postsurgery:** NR **Risk of receiving any allogeneic fresh frozen plasma at up to 30 days postsurgery:** NR **Risk of reoperation or repeat procedure for bleeding within 7 days:** NR **Risk of a thrombotic/thromboembolic event (any composite measured in study) (n/N):** NR **Risk of cerebrovascular attack (CVA/stroke) at up to 30 days:** NR **Risk of myocardial infarction (MI) at up to 30 days:** NR **Risk of deep vein thrombosis (DVT) at up to 90 days:** NR **Risk of pulmonary embolus (PE) at up to 90 days:** NR **Risk of a serious adverse event (SAE) at up to 30 days:** NR **Length of hospital stay (days):** NR **Cost data presented:** no	
Notes	**Sponsorship source:** non‐pharmaceutical (Swedish Medical Research Council) **Country:** Sweden **Setting:** single‐centre **Trial primary outcome:** primary and secondary outcomes not specified in methods, various lab assays are described. **Reference type:** full text **Translation into English required:** no **Trial registration number:** NR **Trial registration timing:** NA	
***Risk of bias***		
**Bias**	**Authors' judgement**	**Support for judgement**
Random sequence generation (selection bias)	Unclear risk	"Fifty patients scheduled for surgery for aorto‐iliac occlusive disease or aneurysms were randomly allocated to receive either desmopressin or a placebo." Method of sequence generation for randomisation not described.
Allocation concealment (selection bias)	Unclear risk	Method of allocation concealment not described.
Blinding of participants and personnel (performance bias) All outcomes	Low risk	"Desmopressin 4 μg/ml (Minirin ®, Ferring, Sweden) was diluted in physiological saline to a volume of 10 ml and injected intravenously over 10min at a dose of 0.3 μg/kg body weight and placebo given as physiological saline." Study stated to be double‐blind, but method not described in detail. However, a need to "break the code" for some exclusions suggests study was properly blinded. Subjective outcomes ‐ low risk of bias due to adequate personnel blinding. Objective outcomes ‐ low risk of bias.
Blinding of outcome assessment (detection bias) All outcomes	Low risk	Subjective outcomes ‐ low risk of bias due to adequate outcome assessor blinding. Objective outcomes ‐ low risk of bias.
Incomplete outcome data (attrition bias) All outcomes	Unclear risk	Six participant in whom surgical complications occurred were excluded, three in the desmopressin group and three in the placebo group. However, the exclusions were balanced in the arms, and thus it is unclear what the effect would have been on the outcome.
Selective reporting (reporting bias)	Unclear risk	No available prospective protocol or trial registration.
Other bias	Low risk	No other concerns such as early stopping or imbalanced study arms.

**Table:** Milne 1996

***Study characteristics***		
Methods	**Design:** RCT (parallel group) **Anticoagulation cessation:** NR **Antiplatelet cessation:** no, 4 out of 18 in the usual care group used aspirin in the week prior to operation and 7 in 21 in the treatment group **Power calculation reached:** NR **Prophylactic or therapeutic randomisation:** prophylactic **Recruitment end date:** NR **Recruitment start date:** NR **Stopping rule:** NR **Strategy for analysis (ITT/PPA):** ITT **Transfusion strategy protocol:** NR **Transfusion threshold in trial:** NR **Trial stopped early:** no	
Participants	**Inclusion criteria:** patients undergoing either arterial bypass surgery with a polytetrafluoroethylene (PTFE) bypass graft (n = 18) or aortic aneurysm repair with a woven Dacron graft (n = 21) **Exclusion criteria:** patients who were known to be seropositive for anti‐HIV, who had a history of hepatitis or whose liver function tests were outside the normal range were excluded. In addition, patients with a history of adverse reactions to blood products or concurrent disease which might compromise their ability to be retained within the study were also excluded from the trial. **Group differences:** no, though few baselines characteristics are given. **Indirectness:** no **Protocol deviations:** no, however rescue therapies were used for some participants "12 patients in the control group and 1 patient in the treatment group required additional action taken at operation to achieve haemostasis. In the control group this consisted of application of Oxycell (n = 7), combination of Oxycell and Lyostypt (n = 3), combination of Oxycell and heparin reversal (n = 1) and application of collagen fleece n = 1. 1 patient in the fibrin sealant group needed application of Lyostypt and collagen fleece." **Reasons for dropouts:** NA **Number of participants randomised:** Fibrin sealant: 21; Usual care: 18 **Number of participants receiving treatment:** Fibrin sealant: 21; Usual care: 18 **Number of participants analysed:** Fibrin sealant: 21; Usual care: 18 **Number of participants dropping out:** 0 **Male gender (%):** Fibrin sealant: 71%; Usual care: 83% **Age (years):** Fibrin sealant: median 73.0 range 62 to 87; median 69.5 range 47 to 85 **Weight (kg):** NR **Ethnicity:** NR **Preoperative haemoglobin (g/L):** NR **Perioperative antiplatelet use (excluding aspirin) (%):** NR **Perioperative aspirin use (%):** Fibrin sealant: 33%; Usual care: 22% **Perioperative anticoagulants (%):** NR **Revision surgery (%):** NR **Elective surgery (%):** NR **Risk score (system used & score):** NR **Cross clamp use (%):** 100% **Cross clamp duration (mins):** NR **Duration of surgery (mins):** NR **Surgical approach/es:** open **Surgical pathology/ies:** Fibrin sealant: aneurysm repair elective 52%, bypass elective 48%; Usual care: aneurysm repair elective 55%, bypass elective 44% **Anatomical region/s:** NR	
Interventions	**Standardised name:** Fibrin sealant: fibrin sealant (fibrinogen and thrombin, which are mixed in the presence of factor XIII and calcium to produce insoluble fibrin); Usual care: usual care **Description in text:** Fibrin sealant: "In the treatment group, following completion of the vascular anastomosis, fibrin sealant was applied to the suture line using a dual syringe technique. The clamps were released 1 min after application was complete;" Usual care: "In the control group nothing was applied to the suture line."	
Outcomes	**Red cell transfusions at up to 30 days postsurgery:** NR **All‐cause mortality at up to 30 days;** NR **and at between 31 and 90 days:** NR **Risk of receiving any allogeneic blood product (composite) at up to 30 days postsurgery:** NR **Risk of receiving any allogeneic packed red cells at up to 30 days postsurgery:** NR **Risk of receiving any allogeneic platelets at up to 30 days postsurgery:** NR **Risk of receiving any allogeneic fresh frozen plasma at up to 30 days postsurgery:** NR **Risk of reoperation or repeat procedure for bleeding within 7 days:** NR **Risk of a thrombotic/thromboembolic event (any composite measured in study) (n/N):** Fibrin sealant: 1/21; Usual care: 0/18 **Risk of cerebrovascular attack (CVA/stroke) at up to 30 days:** Fibrin sealant: 0/21; Usual care: 0/18 **Risk of myocardial infarction (MI) at up to 30 days:** Fibrin sealant: 0/21; Usual care: 0/18 **Risk of deep vein thrombosis (DVT) at up to 90 days:** Fibrin sealant: 0/21; Usual care: 0/18 **Risk of pulmonary embolus (PE) at up to 90 days:** Fibrin sealant: 0/21; Usual care: 0/18 **Risk of a serious adverse event (SAE) at up to 30 days:** NR **Length of hospital stay (days):** NR **Cost data presented:** no	
Notes	**Sponsorship source:** nonpharmaceutical (Scottish National Blood Transfusion Service grant) **Country:** Scotland **Setting:** single‐centre **Trial primary outcome:** primary and secondary outcomes not listed in methods. **Reference type:** full text **Translation into English required:** no **Trial registration number:** NR **Trial registration timing:** NA	
***Risk of bias***		
**Bias**	**Authors' judgement**	**Support for judgement**
Random sequence generation (selection bias)	Low risk	"Following entry into the trial the patients were randomised in a computer‐generated sequence to treatment or control." Adequate method of sequence generation with computer‐generated code.
Allocation concealment (selection bias)	Unclear risk	Method of allocation concealment not described.
Blinding of participants and personnel (performance bias) All outcomes	High risk	"The study was a prospective, randomised non‐blinded trial." Unblinded study. Subjective outcomes ‐ high risk of bias due to inadequate or absent personnel blinding. Objective outcomes ‐ low risk of bias regardless
Blinding of outcome assessment (detection bias) All outcomes	High risk	Unblinded study. Subjective outcomes ‐ high risk of bias due to inadequate or absent outcome assessor blinding. Objective outcomes ‐ low risk of bias.
Incomplete outcome data (attrition bias) All outcomes	Low risk	No obvious outcome data missing.
Selective reporting (reporting bias)	High risk	Methods state they intend to report a particular outcome (e.g. blood loss, blood product usage) but this is not presented with numerical data in the results.
Other bias	Low risk	No other concerns such as early stopping or imbalanced study arms, however limited baseline characteristic data given

**Table:** Minkowitz 2019

***Study characteristics***		
Methods	**Design:** RCT (parallel group) **Anticoagulation cessation:** no, "For vascular open operations, anticoagulation with heparin before arterial clamping was allowed according to the standard practice." **Antiplatelet cessation:** NR **Power calculation reached:** NR **Prophylactic or therapeutic randomisation:** NR **Recruitment end date:** November 2015 **Recruitment start date:** January 2014 **Stopping rule:** NR **Strategy for analysis (ITT/PPA):** Postrandomisation exclusions due to poorly chosen timing of randomisation. "The following 4 analysis populations were initially defined in the protocol: the intent to treat (ITT) population, defined as all patients enrolled and randomized into the study; the modified ITT (mITT) population included all patients who were randomized into the study and treated with any amount of TTH‐Grifols or BT‐JMI; the per‐protocol population, defined as the mITT population excluding any patients with 1 or more major protocol deviations that could impact the evaluation of efficacy data, as determined at a data review meeting before unblinding; and the safety population, included all patients who received any amount of TTH‐Grifols or BT‐JMI. The efficacy analysis was prespecified in the protocol to be performed using the patients included in the mITT population." **Transfusion strategy protocol:** NR **Transfusion threshold in trial:** NR **Trial stopped early:** no	
Participants	**Inclusion criteria:** "adult and pediatric patients who required an elective (non‐emergency), open (non‐laparoscopic; non‐endovascular) surgical procedure from among the following categories: vascular open operation, which is a surgical procedure involving a native artery graft end to side proximal anastomosis using coated or uncoated polytetrafluoroethylene graft... . Additional inclusion criteria were preoperative hemoglobin >/= 9.0 g/dL and fibrinogen level >/= 150 mg/dL within 24 hours before the surgical procedure." **Exclusion criteria:** "traumatic injury or infective process in the anatomic surgical area, history of severe reactions to any blood‐derived product, and pregnancy." **Group differences:** not reported for vascular subgroup. Overall some large imbalances by sex, weight, TBS bleeding intensity, TBS size. **Indirectness:** yes, 17.1% vascular. Paediatric patients also included. Numbers NR **Protocol deviations:** Not reported separately for vascular subgroup. "A total of 137 patients were randomized to the TTH‐Grifols treatment group, including 17 who were not dosed, resulting in an mITT population of 120 patients. The per‐protocol population included 104 (86.7%) patients from the mITT population after 16 (13.3%) were excluded for at least 1 major protocol deviation that might have had an impact on the primary efficacy assessment. In the BT‐JMI treatment group, 68 patients were randomized, including 7 who were not dosed, resulting in the mITT population of 61 patients. Eleven of 61 (18.0%) patients had at least 1 major protocol deviation (mostly procedural, visit window, investigational product, and informed consent) that might have had an impact on the primary efficacy assessment and were excluded, resulting in the per‐protocol population of 50 (82.0%). Five of 61 (8.2%) patients in the BT‐JMI treatment group were dosed, but prematurely discontinued due to withdrawal of consent (2 patients), lost to follow‐up (2 patients), or an AE (1 patient). Overall, a total of 181 patients of the mITT population in both treatment groups (n = 120 and n = 61) were included in the safety analysis." **Reasons for dropouts:** Human thrombin: screen failures n = 17, excluded from analysis. Within mITT 6 discontinued prematurely but were included in mITT analysis; Bovine thrombin: screen failures n = 7. Within mITT 5 discontinued prematurely but were included in mITT analysis. **Number of participants randomised:** Human thrombin: 137 (number randomised NR in vascular subgroup); Bovine thrombin: 68 (whole population) **Number of participants receiving treatment:** Human thrombin: 120 (20 analysed in vascular subgroup); Bovine thrombin: 61 (whole population) NR for vascular subgroup **Number of participants analysed:** Human thrombin: 120 (20 in vascular subgroup); Bovine thrombin: 61 (whole population) 11 (vascular subgroup) **Number of participants dropping out:** Human thrombin: 6 (whole population); Bovine thrombin: 7 not dosed and not included in mITT 5 discontinued prematurely (whole population) **Male gender (%):** Human thrombin: 41.7% (whole population); Bovine thrombin: 31.1% (whole population) **Age (years):** Human thrombin: mean 55.5 SD 16.0 (whole population); Bovine thrombin: mean 56.4 SD 16.5 (whole population) **Weight (kg):** Human thrombin: mean 82.6 SD 19.3 (whole population); Bovine thrombin: mean 76.6 SD 21.8 (whole population) **Ethnicity:** Human thrombin: White 80.8%; Black or African American 15.8%; Asian 1.7%; American Indian or Alaskan native 0.8%; native Hawaiian/Pacific Islander 0.8%; Other 0 (whole population); Bovine thrombin: White 82.0%; Black or African American 11.5%; Asian 3.3%; American Indian or Alaskan native 1.6%; native Hawaiian/Pacific Islander 0; Other 1.6% (whole population) **Preoperative haemoglobin (g/L):** Human thrombin: ≥ 9.0 g/dL (eligibility criteria); Bovine thrombin: ≥ 9.0 g/dL (eligibility criteria) **Perioperative antiplatelet use (excluding aspirin) (%):** NR **Perioperative aspirin use (%):** NR **Perioperative anticoagulants (%):** NR **Revision surgery (%):** NR **Elective surgery (%):** 100% **Risk score (system used & score):** NR **Cross clamp use (%):** NR **Cross clamp duration (mins):** NR **Duration of surgery (mins):** NR **Surgical approach/es:** non‐laparoscopic; non‐endovascular (eligibility criteria) **Surgical pathology/ies:** NR **Anatomical region/s:** Human thrombin: vascular 16.7%; hepatic 16.7%; soft tissue 19.2%; spinal 47.5%; Bovine thrombin: vascular 18.0%; hepatic 16.4%; soft tissue 19.7%; spinal 45.9%	
Interventions	**Standardised name:** Human thrombin: TTH‐Grifols; Bovine thrombin: BT‐JMI **Description in text:** Human thrombin: "Thrombin purified from human plasma reconstituted as a 1000 IU/mL solution in blinded syringes and applied using Gelfoam® sponges"; Bovine thrombin: "Thrombin of bovine origin reconstituted as a 1000 IU/mL solution in blinded syringes and applied using Gelfoam® sponges"	
Outcomes	**Red cell transfusions at up to 30 days postsurgery:** NR **All‐cause mortality at up to 30 days;** Human thrombin: 0/20; Bovine thrombin: 0/11 **and at between 31 and 90 days:** Human thrombin: 0/20; Bovine thrombin: 1/11 **Risk of receiving any allogeneic blood product (composite) at up to 30 days postsurgery:** NR **Risk of receiving any allogeneic packed red cells at up to 30 days postsurgery:** NR **Risk of receiving any allogeneic platelets at up to 30 days postsurgery:** NR **Risk of receiving any allogeneic fresh frozen plasma at up to 30 days postsurgery:** NR **Risk of reoperation or repeat procedure for bleeding within 7 days:** NR **Risk of a thrombotic/thromboembolic event (any composite measured in study) (n/N):** NR **Risk of cerebrovascular attack (CVA/stroke) at up to 30 days:** NR **Risk of myocardial infarction (MI) at up to 30 days:** NR **Risk of deep vein thrombosis (DVT) at up to 90 days:** NR **Risk of pulmonary embolus (PE) at up to 90 days:** NR **Risk of a serious adverse event (SAE) at up to 30 days:** Human thrombin: 10/120; Bovine thrombin: 6/61 **Length of hospital stay (days):** NR **Cost data presented:** no	
Notes	**Sponsorship source:** "Pharmaceutical (Grifols); regulatory trial "This study was funded by Grifols, the manufacturer of topical thrombin (human) Grifols, Medical writing support was provided by Gines Escolar, MD, PhD, under the direction of the authors, with funding from Grifols. Drs Ayguasanosa and Navarro‐Puerto are employed by Grifols. Dr Villavicencio received research funding from Grifols, paid to his institution. Dr Minkowitz’s institution receives clinical trial grant money from Research Concepts, GP LLC." **Country:** USA **Setting:** multicentre (20) **Trial primary outcome:** proportion of participants achieving haemostasis by five minutes after treatment start at the target bleeding site **Reference type:** full text **Translation into English required:** no **Trial registration number:** NCT02014402 **Trial registration timing:** prospective	
***Risk of bias***		
**Bias**	**Authors' judgement**	**Support for judgement**
Random sequence generation (selection bias)	Unclear risk	Limited information. If blinding was successful unlikely to introduce bias. "Qualified patients were randomized in a 2:1 ratio to the TTH‐Grifols and BT‐JMI treatment groups, stratified by type of operation (vascular, hepatic, soft tissue, and spinal) and subtype of operation in certain cases (vascular, peripheral arterial bypass, and extremity vascular access for hemodialysis) and soft tissue operation (mastopexies and abdominoplasties and non‐mastopexies and non‐abdominoplasties). Randomizations were performed at the study center’s pharmacy using an Interactive Response Technology system, which provided the randomization number and assigned the corresponding thrombin treatment."
Allocation concealment (selection bias)	Low risk	"The assigned study drug was then reconstituted by pharmacy staff at each study center and supplied to the investigators in syringes labeled with coded information to mask the treatment identification. Investigator, study nurses, or testing laboratories were blinded to the treatment group and could not identify the agent being assigned."
Blinding of participants and personnel (performance bias) All outcomes	Low risk	Double‐blind. "The assigned study drug was then reconstituted by pharmacy staff at each study center and supplied to the investigators in syringes labeled with coded information to mask the treatment identification. Investigator, study nurses, or testing laboratories were blinded to the treatment group and could not identify the agent being assigned."
Blinding of outcome assessment (detection bias) All outcomes	Low risk	"The assigned study drug was then reconstituted by pharmacy staff at each study center and supplied to the investigators in syringes labeled with coded information to mask the treatment identification. Investigator, study nurses, or testing laboratories were blinded to the treatment group and could not identify the agent being assigned."
Incomplete outcome data (attrition bias) All outcomes	Unclear risk	Intervention group: 17/137 (12%) participants were not dosed and not analysed. 6 discontinued treatment prematurely but were included in mITT population. Control group: 7/68 (10%) participants were not dosed and not analysed. 5 discontinued treatment prematurely but were included in mITT population. Postrandomisation exclusions due to timing of randomisation; few results reported for vascular subgroup.
Selective reporting (reporting bias)	Unclear risk	All outcomes listed in the protocol were reported. SAEs were counted but not described; the most common treatment‐emergent AEs were listed and fully described. Limited reporting for surgical subgroups, but prespecified outcomes were reported.
Other bias	High risk	This suggests that eligibility was determined after randomisation, with potential for bias if placebo not perfectly masked: "A specific bleeding area/site was defined as the target bleeding site (TBS) for each surgical procedure in patients already randomized when it was determined by the surgeon that control of bleeding by conventional surgical techniques (including suture, ligature, and cautery) was ineffective or impractical and required an adjunct treatment to achieve hemostasis".

**Table:** Monaco 2020

***Study characteristics***		
Methods	**Design:** RCT (parallel group) **Anticoagulation cessation:** "Warfarin was stopped 5 days before surgery, whereas novel oral anticoagulants were suspended 48 h before surgery. Bridging with low weight molecular heparin was considered in patients with increased thromboembolic risk. To prevent deep venous thrombosis and pulmonary thromboembolism, all patients received 4000 units of low molecular weight heparin once a day, from the evening before surgery until 4 weeks after surgery." **Antiplatelet cessation:** "Non‐aspirin antiplatelet agents were discontinued before surgery according to current guidelines, whereas aspirin was continued throughout the perioperative period." **Power calculation reached:** yes, 48 participants per arm calculated and 50 per arm recruited. "Sample size calculations were based on a two‐sided alpha error of 0.05 and 80% power. We expected a reduction in intra‐operative blood losses around 34% in patients treated with tranexamic acid, as reported in the literature in other surgical settings. The mean intra‐operative blood loss for open AAA surgery at our centre was 800 (250‐3100) ml, according to a previous study that covered an 18 yr period. On the basis of these data, a sample size of 48 patients per group was deemed necessary, and we decided to enrol 100 patients in our trial to account for possible protocol deviation". **Prophylactic or therapeutic randomisation:** prophylactic **Recruitment end date:** October 2017 **Recruitment start date:** March 2015 **Stopping rule:** NR **Strategy for analysis (ITT/PPA):** ITT, "All randomised patients received their assigned treatment and were analysed for the primary outcome. All patients were contacted 1 year after randomisation, and none were lost to follow‐up." **Transfusion strategy protocol:** yes **Transfusion threshold in trial:** haemoglobin (< 80 g/L) or haemoglobin (< 100 g/L) if severe hypotension **Trial stopped early:** no	
Participants	**Inclusion criteria:** "Patients older than 50 years undergoing open AAA surgical repair who provided written informed consent were eligible for the trial." **Exclusion criteria:** "Patients undergoing urgent or emergent surgery, non‐collaborating or psychiatric patient, patients with known history of allergy to tranexamic acid, seizures, acute venous or arterial thrombosis, fibrinolytic conditions because of consumptive coagulopathy, severe renal insufficiency (defined as estimated glomerular filtration rate below 30 ml min^‐1^ 1.73m^‐2^), haematuria, and patients with ocular disturbances, including blurred vision, poor sight, or altered colour perception were excluded from the trial." **Group differences:** no, "The two groups were balanced in terms of patient characteristics, revised cardiac risk index, renal function, or perioperative medical therapy including anticoagulation and antiplatelet with the exception of there being more female patients in the tranexamic acid group, which consequently had a lower BMI, and a slightly higher baseline INR in the tranexamic acid group." "The ratio of patients receiving tube and bifurcated grafts did not vary between groups. Use of cell saver was also similar between the two groups." **Indirectness:** no **Protocol deviations:** NA **Reasons for dropouts:** NA **Number of participants randomised:** TXA: 50; Placebo: 50 **Number of participants receiving treatment:** TXA: 50; Placebo: 50 **Number of participants analysed:** TXA: 50; Placebo: 50 **Number of participants dropping out:** 0 **Male gender (%):** TXA: 86%; Placebo: 100% **Age (years):** TXA: median 69 IQR 63 to 75; Placebo: median 71 IQR 65 to 74 **Weight (kg):** TXA: mean 25.3 SD 3.33; Placebo: mean 26.2 SD 2.64 **Ethnicity:** NR **Preoperative haemoglobin (g/L):** preoperative haematocrit: TXA: mean 41 SD 3.8; Placebo: mean 41 SD 3.6 **Perioperative antiplatelet use (excluding aspirin) (%):** TXA: 4%; Placebo: 0% **Perioperative aspirin use (%):** TXA: 58%; Placebo: 68% **Perioperative anticoagulants (%):** TXA: 4%; Placebo: 0% **Revision surgery (%):** NR **Elective surgery (%):** 100% **Risk score (system used & score):** TXA: ASA 1/2 28%; Placebo: ASA 1/2 26% **Cross clamp use (%)** 100% **Cross clamp duration (mins):** TXA: mean 36 SD 13; Placebo: mean 37 SD 14 **Duration of surgery (mins):** TXA: mean 137 SD 52; Placebo: mean 138 SD 49 **Surgical approach/es:** open **Surgical pathology/ies:** elective AAA **Anatomical region/s:** abdominal	
Interventions	**Standardised name:** TXA: TXA 500 mg intravenous loading dose plus TXA 250 mg/hr infusion; Placebo: placebo NS EV **Description in text:** TXA: "tranexamic acid (a loading dose of 500 mg and a continuous infusion of 250 mg h^‐1^)"; Placebo: placebo NS EV	
Outcomes	**Red cell transfusions at up to 30 days postsurgery:** NR **All‐cause mortality at up to 30 days;** TXA: 0/50; Placebo: 0/50 **and at between 31 and 90 days:** only available at 365 days**:** TXA: 0/50; Placebo: 3/50 **Risk of receiving any allogeneic blood product (composite) at up to 30 days postsurgery:** TXA: 2/50; Placebo 3/50 **Risk of receiving any allogeneic packed red cells at up to 30 days postsurgery:** TXA: 7/50; Placebo: 12/50 **Risk of receiving any allogeneic platelets at up to 30 days postsurgery:** NR **Risk of receiving any allogeneic fresh frozen plasma at up to 30 days postsurgery:** TXA: 1/50; Placebo: 1/50 **Risk of reoperation or repeat procedure for bleeding within 7 days:** TXA: 0/50; Placebo: 1/50 **Risk of a thrombotic/thromboembolic event (any composite measured in study) (n/N):** TXA: 0/50; Placebo: 0/50 **Risk of cerebrovascular attack (CVA/stroke) at up to 30 days:** NR **Risk of myocardial infarction (MI) at up to 30 days:** TXA: 2/50; Placebo: 2/50 **Risk of deep vein thrombosis (DVT) at up to 90 days:** NR **Risk of pulmonary embolus (PE) at up to 90 days:** NR **Risk of a serious adverse event (SAE) at up to 30 days:** NR **Length of hospital stay (days):** TXA: mean 6 SD 1.5; Placebo: mean 6 SD 1.2 **Cost data presented:** no	
Notes	**Sponsorship source:** pharmaceutical (Rottapharm Biotech S. r. l.). No statement of independence of authors. **Country:** Italy **Setting:** single‐centre **Trial primary outcome:** The primary outcome was intraoperative blood loss calculated as the sum of blood volume aspired during surgery and blood volume absorbed in gauzes. The secondary outcomes consisted of patients receiving packed red blood cells (during surgery and until hospital discharge), occurrence of thromboembolic events up to 28days after surgery, and mortality 28 days and 1 year after surgery. **Reference type:** full text, abstract, supplementary material **Translation into English required:** no **Trial registration number:** NCT02335359, EudraCT (2014‐001456‐39 **Trial registration timing:** prospective	
***Risk of bias***		
**Bias**	**Authors' judgement**	**Support for judgement**
Random sequence generation (selection bias)	Low risk	"Randomisation sequence was created by permuted block randomisation with a block size of 20 and a 1:1 allocation generated by a computer." Adequate method of sequence generation with computer‐generated random sequence.
Allocation concealment (selection bias)	Low risk	"The allocation sequence was prepared by an independent operator not otherwise involved in the trial and was concealed by opaque sequentially numbered sealed envelopes. Patients were screened for eligibility and were asked to sign an informed consent document to participate in the trial on the day before surgery. Patients were randomised in the trial after they entered the surgical theatre, performed by dedicated study personnel in a separate environment. Patients were randomly allocated to the intervention or the placebo group by assigning them the sequentially numbered envelope with the lowest numeration." Adequate method of central allocation concealment
Blinding of participants and personnel (performance bias) All outcomes	Low risk	"Patients randomised to the intervention group received tranexamic acid (Ugurol; Rottapharm S.p.A., Milan, Italy; 0.5 g 5 ml^‐1^ vial) according to the following protocol: a loading dose of 500 mg of tranexamic acid diluted in 100 ml saline was intravenously infused slowly 20 min before surgery, and a continuous intravenous infusion of tranexamic acid was then administered at a rate of 250 mg h (2.5 ml h ^‐1^, using non‐diluted tranexamic acid contained in the vials) from surgical incision until skin closure. Patients randomised to the control group received placebo (saline) with identical volumes and rates of infusion." "We performed a single‐centre, double‐blinded, parallel‐group, randomised clinical trial"
Blinding of outcome assessment (detection bias) All outcomes	Low risk	Subjective outcomes ‐ low risk of bias due to adequate outcome assessor blinding. Objective outcomes ‐ low risk of bias.
Incomplete outcome data (attrition bias) All outcomes	Low risk	"All randomised patients received their assigned treatment and were analysed for the primary outcome. All patients were contacted 1 yr after randomisation, and none were lost to follow‐up." Outcome data set reported with denominators and numerators, and with no dropouts reported.
Selective reporting (reporting bias)	Low risk	All outcomes planned in the protocol or prospective trial registration are reported.
Other bias	Low risk	No other concerns noted.

**Table:** NCT02094885

***Study characteristics***		
Methods	**Design:** RCT (parallel group) **Anticoagulation cessation:** NR **Antiplatelet cessation:** NR **Power calculation reached:** NR **Prophylactic or therapeutic randomisation:** prophylactic **Recruitment end date:** 1 November 2014 **Recruitment start date:** 1 February 2014 **Stopping rule:** NR **Strategy for analysis (ITT/PPA):** ITT. Results from trial registration; baselines for results match numbers randomised, including 6 deaths (1 + 5) and withdrawal due to age (1 + 0). **Transfusion strategy protocol:** NR **Transfusion threshold in trial:** NA **Trial stopped early:** no	
Participants	**Inclusion criteria:** "Subjects between 18 and 75 years of age; Undergoing elective vascular surgical procedures and with the presence of an appropriate Target Bleeding Site (TBS) requiring an adjunct to achieve hemostasis as identified intra‐operatively by the surgeon; Able and willing to comply with procedures required by protocol; Signed and dated written informed consent prior to any study related procedures." **Exclusion criteria:** "Subjects with any intra‐operative findings that may preclude conducting of the study procedures; Intended use of Fibrin Sealants (including autologous Fibrin Sealants) other than Bioseal on the TBS; Subjects with known intolerance to blood products or to one of the components of the study product or unwilling to receive blood products; Subjects with known allergies to or previously used porcine derived products; Female subjects who are known breastfeeding or pregnant or intend to become pregnant during the clinical study period. The subject, in the opinion of the investigator, would not be suitable for participation in the study. Subjects who participated in another trial within 30 days prior to the planned start of treatment." **Group differences:** no **Indirectness:** no **Protocol deviations:** NA **Reasons for dropouts:** NA **Number of participants randomised:** Fibrin sealant: 125; Usual care: 127 **Number of participants receiving treatment:** Fibrin sealant: 125; Usual care: 127 **Number of participants analysed:** Fibrin sealant: 125; Usual care: 127 **Number of participants dropping out:** Fibrin sealant: 0 (2 did not complete full study, one died and one withdrew due to age); Usual care: 0 (5 did not fully complete study, all 5 died) **Male gender (%):** Fibrin sealant: 72%; Usual care: 74% **Age (years):** Fibrin sealant: mean 57.1 SD 13.0; Usual care: mean 56.8 SD 12.2 **Weight (kg):** Fibrin sealant: mean 67.1 SD 11.6; Usual care: mean 66.3 SD 11.2 **Ethnicity:** NR **Preoperative haemoglobin (g/L):** NR **Perioperative antiplatelet use (excluding aspirin) (%):** NR **Perioperative aspirin use (%):** NR **Perioperative anticoagulants (%):** NR **Revision surgery (%):** NR **Elective surgery (%):** 100% **Risk score (system used & score):** NR **Cross clamp use (%):** NR **Cross clamp duration (mins):** NR **Duration of surgery (mins):** NR **Surgical approach/es:** open **Surgical pathology/ies:** elective vascular surgical procedures **Anatomical region/s:** NR	
Interventions	**Standardised name:** Fibrin sealant: Bioseal; Usual care: Usual care (manual compression) **Description in text:** Fibrin sealant: "Experimental: Bioseal Fibrin Sealant. A porcine‐derived fibrin sealant consisting of thrombin and fibrinogen"; Usual care: "Manual compression (MC) include any active or inactive adjunctive treatment to hemostasis methods currently used based on each surgeon's surgical practice except for the use of other fibrin sealants."	
Outcomes	**Red cell transfusions at up to 30 days postsurgery:** NR **All‐cause mortality at up to 30 days;** Fibrin sealant: 1/125; Usual care: 5/127 **and at between 31 and 90 days:** NR **Risk of receiving any allogeneic blood product (composite) at up to 30 days postsurgery:** NR **Risk of receiving any allogeneic packed red cells at up to 30 days postsurgery:** NR **Risk of receiving any allogeneic platelets at up to 30 days postsurgery:** NR **Risk of receiving any allogeneic fresh frozen plasma at up to 30 days postsurgery:** NR **Risk of reoperation or repeat procedure for bleeding within 7 days:** NR **Risk of a thrombotic/thromboembolic event (any composite measured in study) (n/N):** NR **Risk of cerebrovascular attack (CVA/stroke) at up to 30 days:** Fibrin sealant: 0/125; Usual care: 1/127 **Risk of myocardial infarction (MI) at up to 30 days:** Fibrin sealant: 1/125; Usual care: 1/127 **Risk of deep vein thrombosis (DVT) at up to 90 days:** NR **Risk of pulmonary embolus (PE) at up to 90 days:** NR **Risk of a serious adverse event (SAE) at up to 30 days: :** Fibrin sealant: 13/125; Usual care: 11/127 **Length of hospital stay (days):** NR **Cost data presented:** no	
Notes	**Sponsorship source:** pharmaceutical (Ethicon, Inc and Guangzhou Bioseal Biotechnology Co., Ltd) **Country:** China **Setting:** multicentre (9) **Trial primary outcome:** haemostasis at the target bleeding site at 10 minutes following the completion of treatment application. **Reference type:** trial registration with results **Translation into English required:** no **Trial registration number:** NCT02094885 **Trial registration timing:** retrospectively (however, the study only started 1 to 2 months after trial registration)	
***Risk of bias***		
**Bias**	**Authors' judgement**	**Support for judgement**
Random sequence generation (selection bias)	Unclear risk	Extracted from results in trial protocol. No details of randomisation available. The trial registration says that allocation is "randomized", but no further details. Method of sequence generation for randomisation not described.
Allocation concealment (selection bias)	Unclear risk	Extracted from results in trial protocol. No details of allocation concealment available.
Blinding of participants and personnel (performance bias) All outcomes	High risk	Due to the nature of the intervention and comparison blinding is not possible for personnel. Also, the trial register says that they used "single masking (participant)". Subjective outcomes ‐ high risk of bias due to absent personnel blinding. Objective outcomes ‐ low risk of bias regardless.
Blinding of outcome assessment (detection bias) All outcomes	High risk	Subjective outcomes ‐ high risk of bias due to absent outcome assessor blinding. Objective outcomes ‐ low risk of bias.
Incomplete outcome data (attrition bias) All outcomes	Low risk	Data are available for all participants. 1/125 in the intervention group withdrew due to age. 5/127 in the control group did not complete the study due to death.
Selective reporting (reporting bias)	Low risk	All outcomes specified in the trial protocol reported for all participants. Full description of adverse events is available.
Other bias	Low risk	No other concerns noted

**Table:** Nenezic 2019

***Study characteristics***		
Methods	**Design:** RCT (parallel group) **Anticoagulation cessation:** "Anticoagulation with heparin before arterial clamping was required. The initial heparin dose administered was 100 IU/kg body weight 610% for patients having arterio‐arterial bypass grafts and 50 IU/kg body weight +/‐10% for patients having arteriovenous grafts for hemodialysis. Additional doses of heparin could be administered at the discretion of the investigator if needed. Heparin reversal with protamine, if necessary according to surgeon’s judgment, was only allowed after the primary end point assessment at T_4_." 16.5% of the fibrin sealant group received heparins and 17.5% of the manual compression group received heparins. 7.3% of the fibrin sealant group received vitamin K antagonists and 8.8% of the manual compression group received vitamin K antagonists. **Antiplatelet cessation:** Fibrin sealant: 7.3% received vitamin K antagonists; Usual care: 8.8% received vitamin K antagonists. **Power calculation reached:** no power calculations reported **Prophylactic or therapeutic randomisation:** therapeutic: "The surgical intervention was performed according to the respective institution’s standards. When it was determined by the surgeon that the control of bleeding from the proximal anastomosis by conventional surgical techniques was ineffective or impractical and required an adjunct treatment to achieve hemostasis, this specific bleeding area/site was identified and defined as the TBS." **Recruitment end date:** December 2015 **Recruitment start date:** August 2012 **Stopping rule:** yes, but rules and timing unclear: "A safety evaluation was performed based on adverse events (AEs) evaluation." **Strategy for analysis (ITT/PPA):** ITT for efficacy analysis **Transfusion strategy protocol:** NR **Transfusion threshold in trial:** NA **Trial stopped early:** no	
Participants	**Inclusion criteria:** "Hemoglobin (Hgb) ≥ 8.0 g/dL at Baseline (within 24 hours prior to surgical procedure). Require elective (non‐emergency), primary, open (non‐laparoscopic; non‐endovascular) peripheral vascular surgery. Require one of the following peripheral vascular procedures involving proximal end‐to‐side arterial anastomosis utilizing coated or uncoated Polytetrafluoroethylene grafts: a) Femoral‐femoral bypass grafting, b) Femoral‐popliteal bypass grafting, c) Femoral‐distal bypass grafting, d) Ilio‐iliac bypass grafting, e) Ilio‐femoral bypass grafting, f) Ilio‐popliteal bypass grafting, g) Aorto‐iliac bypass grafting, h) Aorto‐femoral bypass grafting, i) Axillo‐femoral bypass grafting, and j) Upper extremity vascular access for hemodialysis. A target bleeding site can be identified. Target bleeding site has moderate arterial bleeding." **Exclusion criteria:** "Undergoing a re‐operative procedure. Undergoing other vascular procedures during the same surgical session (stenting and/or endarterectomy of the same artery are allowed). Have an infection in the anatomic surgical area. Have a history of severe (e.g. anaphylactic) reactions to blood or to any blood‐derived product. Have previous known sensitivity to any Fibrin Sealant Grifols, heparin, or protamine component. Females who are pregnant or nursing a child at Baseline (within 24 hours prior to surgical procedure). Have undergone a therapeutic surgical procedure within 30 days from the screening visit. Target bleeding site cannot be identified. Target bleeding site has mild or severe arterial bleeding. Occurrence of major intraoperative complications that require resuscitation or deviation from the planned surgical procedure. Intraoperative change in planned surgical procedure, which results in subject no longer meeting preoperative inclusion and/or exclusion criteria." **Group differences:** no obvious differences in age, gender, race, weight, comorbidities (hypertension, peripheral arterial disease, hyperlipidaemia), prior or concomitant medications, or procedure mix, however statistical significance not presented. **Indirectness:** 12.8% (fibrin sealant) and 17.5% (usual care) of participants underwent upper extremity vascular access procedures (not target population). **Protocol deviations:** there were 17 (of 166) protocol deviations in total, 12 (of 109) in the fibrin sealant group and 5 (of 57) in the manual compression group. The reasons for protocol deviations were not listed. **Reasons for dropouts:** 3 of 109 patients in the fibrin sealant group discontinued prematurely (two consent withdrawals and one death) and one of 57 in the manual compression group discontinued prematurely (1 death). **Number of participants randomised:** Fibrin sealant: 109; Usual care: 57 **Number of participants receiving treatment:** Fibrin sealant: 109; Usual care: 57 **Number of participants analysed:** Fibrin sealant: 109; Usual care: 57 **Number of participants dropping out:** Fibrin sealant: 0 at first follow‐up, 3 discontinued prematurely (2 consent withdrawals, one death); Usual care: 0 at first follow‐up; 1 discontinued prematurely due to death **Male gender (%):** 64.5% **Age (years):** Fibrin sealant: median 64 range 44 to 84; Usual care: median 61 range 22 to 82 **Weight (kg):** Fibrin sealant: mean 77.2 SD 17.0; Usual care: mean 73.6 SD 15.0 **Ethnicity:** Fibrin sealant: Caucasian 92.7%; Usual care: Caucasian 86.0% **Preoperative haemoglobin (g/L):** NR **Perioperative antiplatelet use (excluding aspirin) (%):** Fibrin sealant: total platelet aggregation inhibitors 38.5%; Usual care: total platelet aggregation inhibitors 38.6% **Perioperative aspirin use (%):** NR **Perioperative anticoagulants (%):** Fibrin sealant: heparins 16.5%, vitamin K‐antagonists 7.3%; Usual care: heparins 17.5%, vitamin K‐antagonists 8.8% **Revision surgery (%):** 0 **Elective surgery (%):** 100% **Risk score (system used & score):** NR **Cross clamp use (%):** 100% **Cross clamp duration (mins):** NR **Duration of surgery (mins):** NR **Surgical approach/es:** open **Surgical pathology/ies:** peripheral vascular procedures (bypass, vascular access for haemodialysis) **Anatomical region/s:** femoral‐popliteal, femoral‐femoral, axillofemoral, aortofemoral, aortoiliac, femoral‐distal, illioiliac, iliopopliteal	
Interventions	**Standardised name:** Fibrin sealant: fibrin sealant (Grifols); Usual care: usual care (manual compression with gauze) **Description in text:** Fibrin sealant: "Fibrin Sealant Grifols consisting of 3 mL fibrinogen and 3 mL thrombin in separate syringes assembled on a syringe holder (6 mL of solution in total)"; Usual care: "Direct manual compression of target bleeding site with gauze/laparotomy pads. In patients randomized to MC, clamps were removed immediately and direct MC was applied to the TBS completely covering the suture line using gauze pads. The arterial flow was re‐established by releasing the clamps. There was no limitation to the number of gauzes applied to the TBS for achieving hemostasis."	
Outcomes	**Red cell transfusions at up to 30 days postsurgery:** NR **All‐cause mortality at up to 30 days;** Fibrin sealant: 1/109; Usual care: 1/57 **and at between 31 and 90 days:** NR **Risk of receiving any allogeneic blood product (composite) at up to 30 days postsurgery:** NR **Risk of receiving any allogeneic packed red cells at up to 30 days postsurgery:** NR **Risk of receiving any allogeneic platelets at up to 30 days postsurgery:** NR **Risk of receiving any allogeneic fresh frozen plasma at up to 30 days postsurgery:** NR **Risk of reoperation or repeat procedure for bleeding within 7 days:** NR **Risk of a thrombotic/thromboembolic event (any composite measured in study) (n/N):** NR **Risk of cerebrovascular attack (CVA/stroke) at up to 30 days:** NR **Risk of myocardial infarction (MI) at up to 30 days:** NR **Risk of deep vein thrombosis (DVT) at up to 90 days:** NR **Risk of pulmonary embolus (PE) at up to 90 days:** NR **Risk of a serious adverse event (SAE) at up to 30 days:** (reported at 6 weeks time point) Fibrin sealant: 21/109; Usual care: 11/57 **Length of hospital stay (days):** NR **Cost data presented:** no	
Notes	**Sponsorship source:** pharmaceutical. Four pharmaceutical employees on the authorship and two medical writers acknowledged. **Country:** Hungary, Russia, Serbia, USA **Setting:** multicentre (38) **Trial primary outcome:** "Primary outcome: Proportion of Subjects Achieving Hemostasis by Four Minutes After Treatment Start Secondary outcomes: Time to Hemostasis (TTH) Cumulative Proportion of Subjects Having Achieved Hemostasis at the Target Bleeding Site by Specified Time Points Prevalence of Treatment Failures" **Reference type:** full text **Translation into English required:** no **Trial registration number:** NCT01662856 **Trial registration timing:** prospective	
***Risk of bias***		
**Bias**	**Authors' judgement**	**Support for judgement**
Random sequence generation (selection bias)	Unclear risk	"In the primary part of the study, patients were randomized 2:1 into FS Grifols or MC treatment groups." Method of randomisation not described in sufficient detail.
Allocation concealment (selection bias)	Low risk	"All study centers were provided with sealed opaque envelopes with a number printed outside and containing a treatment group. A randomization number was printed on the outside of the envelopes. For each patient undergoing a surgery, the ﬁrst, sequential, available randomization envelope for the appropriate type of procedure (peripheral arterial bypass or upper extremity vascular access or hemodialysis) was taken to the operating room. The envelope was opened only upon identifying a TBS on the proximal anastomosis with moderate arterial bleeding, according to the investigator’s judgment, and the time of the randomization and the treatment group assignment was recorded." Adequate method of central allocation concealment with sequentially numbered sealed opaque envelopes.
Blinding of participants and personnel (performance bias) All outcomes	High risk	"Study design. This clinical study was a pivotal confirmatory phase III, controlled, prospective, single‐blinded, randomized, multicenter clinical trial performed at 42 sites in United States, Hungary, Serbia, and Russia (ClinicalTrials.gov Identiﬁer: NCT01662856)." "Data from patients participating in the primary part of the study, including treatment assignment and accumulating efﬁcacy data, were blinded to the sponsor team members, except for personnel from study drug supply groups." Single blinded study ‐ surgeon was aware which patient had received. Subjective outcomes ‐ high risk of bias due to inadequate or absent personnel blinding. Objective outcomes ‐ low risk of bias regardless.
Blinding of outcome assessment (detection bias) All outcomes	Unclear risk	"Data from patients participating in the primary part of the study, including treatment assignment and accumulating efficacy data, were blinded to the sponsor team members, except for personnel from study drug supply groups." Hard to establish whether outcome assessors were blinded.
Incomplete outcome data (attrition bias) All outcomes	Low risk	"The ITT population consisted of 166 patients recruited in 18 centers. Three of the 109 patients randomized to FS (2.8%) in the primary part discontinued prematurely (two consent withdrawals, one death). One of the 57 patients randomized to MC (1.8%) discontinued (death)." 9% of participants in the fibrin sealant group vs 3% of patients did not complete the study to the end of follow up which could have introduced bias. However, in terms of study completion, only 2.8% and 1.8% did not complete study. Therefore, the risk of bias from incomplete outcomes data is probably low.
Selective reporting (reporting bias)	Low risk	All outcomes planned in the protocol or prospective trial registration are reported.
Other bias	Low risk	No other concerns such as early stopping or imbalanced study arms.

**Table:** O'Donnell 2010

***Study characteristics***		
Methods	**Design:** RCT (parallel group) **Anticoagulation cessation:** NR **Antiplatelet cessation:** NR **Power calculation reached:** NR **Prophylactic or therapeutic randomisation:** NR **Recruitment end date:** NR **Recruitment start date:** NR **Stopping rule:** NR **Strategy for analysis (ITT/PPA):** NR **Transfusion strategy protocol:** NR **Transfusion threshold in trial:** NR **Trial stopped early:** NR	
Participants	**Inclusion criteria:** NR **Exclusion criteria:** NR **Group differences:** NR **Indirectness:** no **Protocol deviations:** NR **Reasons for dropouts:** NR **Number of participants randomised:** NR **Number of participants receiving treatment:** NR **Number of participants analysed:** NR **Number of participants dropping out:** NR **Male gender (%):** NR **Age (years):** NR **Weight (kg):** NR **Ethnicity:** NR **Preoperative haemoglobin (g/L):** NR **Perioperative antiplatelet use (excluding aspirin) (%):** NR **Perioperative aspirin use (%):** NR **Perioperative anticoagulants (%):** NR **Revision surgery (%):** NR **Elective surgery (%):** NR **Risk score (system used & score):** NR **Cross clamp use (%):** NR **Cross clamp duration (mins):** NR **Duration of surgery (mins):** NR **Surgical approach/es:** NR **Surgical pathology/ies:** NR **Anatomical region/s:** NR	
Interventions	**Standardised name:** Vascular sealant: no details reported; Gelfoam: GELFOAM/Thrombin **Description in text:** No further details reported	
Outcomes	**Red cell transfusions at up to 30 days postsurgery:** NR **All‐cause mortality at up to 30 days:** NR **and at between 31 and 90 days:** NR **Risk of receiving any allogeneic blood product (composite) at up to 30 days postsurgery:** NR **Risk of receiving any allogeneic packed red cells at up to 30 days postsurgery:** NR **Risk of receiving any allogeneic platelets at up to 30 days postsurgery:** NR **Risk of receiving any allogeneic fresh frozen plasma at up to 30 days postsurgery:** NR **Risk of reoperation or repeat procedure for bleeding within 7 days:** NR **Risk of a thrombotic/thromboembolic event (any composite measured in study) (n/N):** NR **Risk of cerebrovascular attack (CVA/stroke) at up to 30 days:** NR **Risk of myocardial infarction (MI) at up to 30 days:** NR **Risk of deep vein thrombosis (DVT) at up to 90 days:** NR **Risk of pulmonary embolus (PE) at up to 90 days:** NR **Risk of a serious adverse event (SAE) at up to 30 days:** NR **Length of hospital stay (days):** NR **Cost data presented:** no	
Notes	**Sponsorship source:** NR **Country:** USA **Setting:** single‐centre **Trial primary outcome:** NR **Reference type:** abstract **Translation into English required:** no **Trial registration number:** NR **Trial registration timing:** NA	
***Risk of bias***		
**Bias**	**Authors' judgement**	**Support for judgement**
Random sequence generation (selection bias)	Unclear risk	No information (abstract only)
Allocation concealment (selection bias)	Unclear risk	No information (abstract only)
Blinding of participants and personnel (performance bias) All outcomes	High risk	Open label, easily distinguishable treatments. Subjective outcomes ‐ unclear risk of bias due to lack of personnel blinding. Objective outcomes ‐ low risk of bias.
Blinding of outcome assessment (detection bias) All outcomes	High risk	Open label, easily distinguishable treatments. Primary endpoints assessed intraoperatively. Subjective outcomes ‐ unclear risk of bias due to unclear adequacy of outcome assessor blinding. Objective outcomes ‐ low risk of bias.
Incomplete outcome data (attrition bias) All outcomes	Unclear risk	Abstract only.
Selective reporting (reporting bias)	Unclear risk	Abstract only and no available prospective protocol or trial registration.
Other bias	Unclear risk	Abstract only; no further information

**Table:** POISE‐3 2022

***Study characteristics***		
Methods	**Design:** RCT (parallel group) **Anticoagulation cessation:** NR **Antiplatelet cessation:** NR **Power calculation reached:** No **Prophylactic or therapeutic randomisation:** prophylactic **Recruitment end date:** July 2021 **Recruitment start date:** June 2018 **Stopping rule:** no formal stopping rule. "The Data Monitoring Committee (DMC) will provide oversight of patients’ safety throughout the trial by reviewing unblinded aggregate data (including all reported study outcome events and SAEs) by treatment group at regular intervals throughout the duration of the trial and as defined in the DMC Charter." **Strategy for analysis (ITT/PPA):** ITT for effectiveness, PPA for safety (non‐inferiority). **Transfusion strategy protocol:** NR **Transfusion threshold in trial:** NR **Trial stopped early:** yes, "Owing to a financial deficit resulting from slowed recruitment during the coronavirus disease 2019 (Covid‐19) pandemic, the steering committee stopped recruitment on July 15, 2021, after at least 9500 patients had undergone randomization. This decision was made without knowledge of the trial results but with knowledge that the incidences of the aggregate composite bleeding and composite cardiovascular outcome events were higher than originally estimated. We estimated that a sample of 9500 patients would provide the trial with 90% power to detect a hazard ratio of 0.80 or less (two‐sided alpha level of 0.05) for tranexamic acid as compared with placebo, assuming an incidence of composite bleeding outcome events of 9.0% in the placebo group. Stopping the trial at 9500 patients also provided the trial with 98% power for a noninferiority margin, expressed as a hazard ratio of 1.125 (one‐sided alpha level of 0.025), under the assumption of an incidence of composite cardiovascular outcome events of 14.0% in the placebo group and an expected hazard ratio of 0.90 and with adjustment for the partial factorial design."	
Participants	**Inclusion criteria:** "Eligible patients were 45 years of age or older, were undergoing inpatient noncardiac surgery, and were at risk for bleeding and cardiovascular complications according to criteria previously associated with perioperative bleeding and cardiovascular complications (e.g., known atherosclerotic disease, undergoing major surgery, an age of ≥ 70 years, and a serum creatinine level of > 175 μmol per liter [2.0 mg per deciliter])." **Exclusion criteria:** "Patients were excluded if they were undergoing cardiac surgery or intracranial neurosurgery, if a physician planned to administer systemic tranexamic acid during surgery, or if the patient had a creatinine clearance of less than 30 ml per minute (Cockcroft–Gault equation) or was receiving long‐term dialysis." **Group differences:** baseline characteristics not reported separately for vascular subgroup but overall the trial is well‐balanced. **Indirectness:** no (vascular subgroup reported) **Protocol deviations:** NR for vascular subgroup (39 missing from PPA for safety) **Reasons for dropouts:** NR for vascular subgroup **Number of participants randomised:** TXA: 699; Placebo: 700 **Number of participants receiving treatment:** NR **Number of participants analysed:** TXA: 699; (684 PPA for safety); Placebo: 700; (676 PPA for safety) **Number of participants dropping out:** NR **Male gender (%):** NR **Age (years):** NR **Weight (kg):** NR **Ethnicity:** NR **Preoperative haemoglobin (g/L):** NR **Perioperative antiplatelet use (excluding aspirin) (%):** NR **Perioperative aspirin use (%):** NR **Perioperative anticoagulants (%):** NR **Revision surgery (%):** NR **Elective surgery (%):** NR **Risk score (system used & score):** NR **Cross clamp use (%):** NR **Cross clamp duration (mins):** NR **Duration of surgery (mins):** NR **Surgical approach/es:** NR **Surgical pathology/ies:** NR **Anatomical region/s:** NR	
Interventions	**Standardised name:** TXA: Tranexamic acid; Placebo: NS EV **Description in text:** TXA: "1g bolus before and after surgery"; Placebo: "intravenous placebo (0.9% normal saline) at a loading dose of 1g over 10 minutes, with a second 1g bolus given at the end of surgery when closing the wound. Other Name: saline"	
Outcomes	**Red cell transfusions at up to 30 days postsurgery:** NR **All‐cause mortality at up to 30 days:** NR**; and at between 31 and 90 days:** NR **Risk of receiving any allogeneic blood product (composite) at up to 30 days postsurgery:** NR **Risk of receiving any allogeneic packed red cells at up to 30 days postsurgery:** NR **Risk of receiving any allogeneic platelets at up to 30 days postsurgery:** NR **Risk of receiving any allogeneic fresh frozen plasma at up to 30 days postsurgery:** NR **Risk of reoperation or repeat procedure for bleeding within 7 days:** NR **Risk of a thrombotic/thromboembolic event (any composite measured in study) (n/N):** TXA: 140/684; Placebo: 126/676 **Risk of cerebrovascular attack (CVA/stroke) at up to 30 days:** NR **Risk of myocardial infarction (MI) at up to 30 days:** NR **Risk of deep vein thrombosis (DVT) at up to 90 days:** NR **Risk of pulmonary embolus (PE) at up to 90 days:** NR **Risk of a serious adverse event (SAE) at up to 30 days:** NR **Length of hospital stay (days):** NR **Cost data presented:** no	
Notes	**Sponsorship source:** public sector ("Supported by a Foundation Grant (FDN‐143302, to Dr. Devereaux) from the Canadian Institutes of Health Research, a Project Grant (1162362) from the Australian National Health and Medical Research Council, and a grant from General Research Fund 14104419, Research Grant Council, Hong Kong, and by the Population Health Research Institute." "No external funder had a role in the design or conduct of the trial, collection or analysis of the data, or preparation of the manuscript." **Country:** 22 countries: Canada, USA, Australia, Belgium, Brazil, Chile, China, Denmark, France, Germany, Hong Kong, India, Italy, Malaysia, Netherlands, New Zealand, Pakistan, Poland, Russian Federation, South Africa, Spain, United Kingdom, **Setting:** multicentre (114) **Trial primary outcome:** composite of life‐threatening, major, and critical organ bleeding at 30 days after randomisation. The primary safety outcome is a composite of myocardial injury after noncardiac surgery (MINS) non‐hemorrhagic stroke, peripheral arterial thrombosis, and symptomatic proximal venous thromboembolism at 30 days after randomisation. The secondary outcomes at 30 days after randomisation are as follows: (1) bleeding independently associated with mortality after noncardiac surgery (BIMS); (2) life‐threatening bleeding; (3) major bleeding; (4) critical organ bleeding; (5) MINS; MINS not fulfilling the universal definition of myocardial infarction (7) myocardial infarction; and (8) the composite of vascular death, bleeding (i.e. non‐fatal life‐threatening, major, or critical organ), MINS, stroke, peripheral arterial thrombosis, and symptomatic proximal venous thromboembolism (i.e. a net risk‐benefit outcome). **Reference type:** full text **Translation into English required:** no **Trial registration number:** NCT03505723 **Trial registration timing:** prospective (NCT registration April 2018, start date June 2018)	
***Risk of bias***		
**Bias**	**Authors' judgement**	**Support for judgement**
Random sequence generation (selection bias)	Low risk	"randomization was performed by means of a central computerized system with the use of block randomization, with stratification according to center"
Allocation concealment (selection bias)	Low risk	"randomization was performed by means of a central computerized system with the use of block randomization, with stratification according to center. Patients were assigned in a 1:1 ratio to receive tranexamic acid (1‐g intravenous bolus) or placebo at the start and end of surgery and, in a 1:1 ratio with the use of a partial factorial design, to a hypotension‐avoidance strategy or a hypertension‐avoidance strategy. Patients, health care providers, data collectors, and outcome adjudicators were unaware of the trial‐group assignments."
Blinding of participants and personnel (performance bias) All outcomes	Low risk	"Patients, health care providers, data collectors, and outcome adjudicators were unaware of the trial‐group assignments."
Blinding of outcome assessment (detection bias) All outcomes	Low risk	"Patients, health care providers, data collectors, and outcome adjudicators were unaware of the trial‐group assignments."
Incomplete outcome data (attrition bias) All outcomes	Low risk	Unclear for vascular subgroup. ITT analysis for effectiveness, PPA for safety. Limited reporting of vascular subgroup but 1399 (all individuals randomised) for effectiveness, 1360 for safety.
Selective reporting (reporting bias)	Low risk	All primary and secondary outcomes were reported.
Other bias	Low risk	No other concerns

**Table:** Qerimi 2013

***Study characteristics***		
Methods	**Design:** RCT (parallel group) **Anticoagulation cessation:** NR **Antiplatelet cessation:** NR **Power calculation reached:** unclear whether study aimed to recruit 28 participants in total or 28 per arm **Prophylactic or therapeutic randomisation:** therapeutic **Recruitment end date:** July 2009 **Recruitment start date:** February 2009 **Stopping rule:** yes, "The trial was to be stopped for success if the null hypothesis was rejected with a one‐sided p‐value lower than alpha_1_, and for futility if it was higher than.50. For the final analysis, applying the approach of Bauer and Kohne, the level of significance was set at alpha_2_= 00380/p_1_, p_1_ being the p‐value from the one‐sided test of the primary endpoint in the interim analysis. The significance level was maintained at.025 and with a power of 80%, values obtained by simulation based on 300 replicates". **Strategy for analysis (ITT/PPA):** ITT **Transfusion strategy protocol:** NR **Transfusion threshold in trial:** NA **Trial stopped early:** yes, for stopped for efficacy	
Participants	**Inclusion criteria:** "Patients with an indication for a peripheral vascular reconstruction due to peripheral vascular disease (PVD) including femoro‐femoral, femoro‐popliteal and femoro‐crural reconstructions or the need of a crossover including femoro‐femoral or ilaco‐femoro reconstruction. Suture hole bleeding of peripheral arterial bypass anastomosis using PTFE graft prosthesis. Written informed consent" **Exclusion criteria:** "Emergency surgery; Patients with coagulopathy or uremia; Reoperation within one month at the same location; Pregnant and Breastfeeding Women; Known or suspected allergies or hypersensitivity to any of the used devices (e.g. to material of bovine origin); Severe comorbidity (ASA ≥ 4); Life expectancy less than 12 months; Current immunosuppressive therapy (more than 40 mg of corticoid per day or ezathioprin); Chemotherapy within last 4 weeks; Radiotherapy on the treated region within the last 2 months; Severe psychiatric or neurologic diseases; Lack of compliance" **Group differences:** no: "In the 4 groups, there was no significant difference in age and sex and American Society of Anesthesiologists status." **Indirectness:** no **Protocol deviations:** no **Reasons for dropouts:** NA **Number of participants randomised:** Lyostypt: 8; Surgicel: 8 **Number of participants receiving treatment:** Lyostypt: 8; Surgicel: 8 **Number of participants analysed:** Lyostypt: 7; Surgicel: 8 **Number of participants dropping out:** 1 patient who received Lyostypt died from MI. **Male gender (%):** Lysotypt: 88%; Surgicel: 50% **Age (years):** Lyostypt: men mean 69.7 SD 7.0 women 80.0; Surgicel: men mean 70.5 SD 2.9 women mean 70.5 SD 8.2 **Weight (kg):** Lyostypt: mean BMI 26.8 SD 4.2; Surgicel: mean BMI 25.5 SD 3.5 **Ethnicity:** NR **Preoperative haemoglobin (g/L):** NR **Perioperative antiplatelet use (excluding aspirin) (%):** NR **Perioperative aspirin use (%):** NR **Perioperative anticoagulants (%):** NR **Revision surgery (%):** 0 **Elective surgery (%):** 100% **Risk score (system used & score):** Lyostypt: ASA PS 3 = 87.5%; Surgicel: ASA P3 = 62.5% **Cross clamp use (%):** 100% **Cross clamp duration (mins):** NR **Duration of surgery (mins):** NR **Surgical approach/es:** open **Surgical pathology/ies:** peripheral vascular disease **Anatomical region/s:** femoro‐femoral, femoro‐popliteal and femoro‐crural or ilaco‐femoro	
Interventions	**Standardised name:** Lyostypt: microfibrillar collagen (Lyostypt); Surgicel: oxidized cellulose (Surgicel) **Description in text:** Lyostypt: "Lyostypt is a gamma‐sterilized, absorbable, wet‐stable hemostat made of collagen from cattle skin"; Surgicel: "Surgicel (Johnson & Johnson Medical Ltd, North Yorkshire, UK), an oxidized cellulose hemostat, in stopping suture hole bleeding after arterial bypass surgery"	
Outcomes	**Red cell transfusions at up to 30 days postsurgery:** NR **All‐cause mortality at up to 30 days:** Lyostypt: 1/8; Surgicel: 0/8 **and at between 31 and 90 days:** NR **Risk of receiving any allogeneic blood product (composite) at up to 30 days postsurgery:** NR **Risk of receiving any allogeneic packed red cells at up to 30 days postsurgery:** NR **Risk of receiving any allogeneic platelets at up to 30 days postsurgery:** NR **Risk of receiving any allogeneic fresh frozen plasma at up to 30 days postsurgery:** NR **Risk of reoperation or repeat procedure for bleeding within 7 days:** NR **Risk of a thrombotic/thromboembolic event (any composite measured in study) (n/N):** Lyostypt: 1/8; Surgicel: 0/8 **Risk of cerebrovascular attack (CVA/stroke) at up to 30 days:** NR **Risk of myocardial infarction (MI) at up to 30 days:** Lyostypt: 1/8; Surgicel: 0/8 **Risk of deep vein thrombosis (DVT) at up to 90 days:** NR **Risk of pulmonary embolus (PE) at up to 90 days:** NR **Risk of a serious adverse event (SAE) at up to 30 days:** NR **Length of hospital stay (days):** NR **Cost data presented:** no	
Notes	**Sponsorship source:** pharmaceutical (Aesculap). Two authors are employees of the manufacturer. **Country:** Germany **Setting:** single‐centre **Trial primary outcome:** "The primary endpoint was the time to hemostasis, which was determined from the release of the cross‐clamp, to the moment the wound was completely or sufficiently dry as judged by a single investigator. To assess the safety of the hemostat, complications (e.g., recurrence of bleeding, infection and occlusion of the PTFE prosthesis, stenosis, thrombosis of the leg artery, reoperation, wound infections, and healing disorders) within 30 +/‐ 10 days after surgery were used as secondary endpoints." **Reference type:** full text, trial registration **Translation into English required:** no **Trial registration number:** NCT00837954 **Trial registration timing:** prospective	
***Risk of bias***		
**Bias**	**Authors' judgement**	**Support for judgement**
Random sequence generation (selection bias)	Low risk	"Sequentially numbered (1–32), opaque, sealed envelopes were used to allocate the patients according to a randomization list prepared by a statistician to receive either microﬁbrillar collagen (COLL) or oxidized cellulose (ORC)." Adequate method of sequence generation
Allocation concealment (selection bias)	Low risk	"Sequentially numbered (1–32), opaque, sealed envelopes were used to allocate the patients according to a randomization list prepared by a statistician to receive either microﬁbrillar collagen (COLL) or oxidized cellulose (ORC)." Adequate method of allocation concealment.
Blinding of participants and personnel (performance bias) All outcomes	High risk	"An untreated control group was not included because of the nature of the study and because animal studies have clearly shown that the time to hemostasis increases if no hemostat is used. 8–12 The products make blinding impossible." Subjective outcomes ‐ high risk of bias due to inadequate personnel blinding because comparator is usual care rather than placebo. Objective outcomes ‐ low risk of bias.
Blinding of outcome assessment (detection bias) All outcomes	High risk	Subjective outcomes ‐ high risk of bias due to inadequate or absent personnel blinding. Objective outcomes ‐ low risk of bias regardless
Incomplete outcome data (attrition bias) All outcomes	Low risk	No obvious outcome data missing.
Selective reporting (reporting bias)	Low risk	All outcomes planned in the protocol or prospective trial registration are reported.
Other bias	Unclear risk	The percentage male and disease severity seem quite dissimilar. However, only eight participants in each arm were recruited. It is not clear whether these imbalances in study arms would have affected outcomes.

**Table:** Ranaboldo 1997

***Study characteristics***		
Methods	**Design:** RCT (parallel group) **Anticoagulation cessation:** NR **Antiplatelet cessation:** "Patients were excluded... if any antiplatelet agents had been taken during the preceding week." **Power calculation reached:** no power calculation reported **Prophylactic or therapeutic randomisation:** prophylactic **Recruitment end date:** NR **Recruitment start date:** NR **Stopping rule:** NR **Strategy for analysis (ITT/PPA):** not entirely modified ITT, because apart from the "4 patients who were found at operation not to be suitable", primary data analysis was only performed on "patients surviving to the first week". "Four deaths occurred within 7 days of surgery, and thus these 4 were also excluded from primary analysis. In the end primary analysis was performed on 128 out of 136 randomised patients." **Transfusion strategy protocol:** yes **Transfusion threshold in trial:** haemoglobin (< 100 g/L) **Trial stopped early:** no	
Participants	**Inclusion criteria:** "A consecutive series of 136 patients having elective aortic surgery were recruited." **Exclusion criteria:** "Patients were excluded if there was a history of previous exposure to aprotinin, hepatic or renal impairment, or if any antiplatelet agents had been taken during the preceding week." **Group differences:** no significant differences on any recorded baseline variables. **Indirectness:** no **Protocol deviations:** yes "Four patients were found at operation not to be suitable for the planned reconstructive surgery: two had inflammatory aneurysms, one had unsuspected disseminated malignancy and one had a calcified aorta that could not safely be cross‐clamped. These patients did not complete the protocol and are not considered in the subsequent analysis." The intravenous bolus of aprotinin to patient (2MK) stayed the same, but the continuous intravenous infusion was changed after 80 participants had been randomised, from 0.5 MK/h to 1 MK/h. In total 128 participants were analysed, 66 in the aprotinin group and 62 in the placebo group. So, about two thirds of the participants received 2 MK bolus plus 0.5 MK/h continuous infusion and about one third 2 MK bolus plus 1.0 MK/h continuous infusion. According to the text, the protocol deviation did not affect outcomes when analysed separately. **Reasons for dropouts:** 8 participants dropped out, 4 dropped out because the planned operation was not suitable and 4 were not used in the primary analysis (2 in each group) because they died within 7 days of surgery. "Patients surviving to the first week were included in the primary data analysis but clinical follow‐up continued to the 30th day". "Some 136 patients were randomized. Four deaths occurred within 7 days of surgery (two in each group): one following massive haemorrhage, two from myocardial infarction and one from a pulmonary embolus. Four patients were found at operation not to be suitable for the planned reconstructive surgery: two had inflammatory aneurysms, one had unsuspected disseminated malignancy and one had a calcified aorta that could not safely be cross‐clamped. These patients did not complete the protocol and are not considered in the subsequent analysis. " **Number of participants randomised:** Aprotinin: NR; Placebo: NR **Number of participants receiving treatment:** Aprotinin: 68; Placebo: 64 **Number of participants analysed:** Aprotinin: 66; Placebo: 62 **Number of participants dropping out:** NR **Male gender (%):** Aprotinin: 83%; Placebo: 73% **Age (years):** Aprotinin: median 68; Placebo: median 70 **Weight (kg):** Aprotinin: median 72; Placebo: median 71 **Ethnicity:** NR **Preoperative haemoglobin (g/L):** NR **Perioperative antiplatelet use (excluding aspirin) (%):** NR **Perioperative aspirin use (%):** 0 **Perioperative anticoagulants (%):** 0 **Revision surgery (%):** NR **Elective surgery (%):** 100% **Risk score (system used & score):** NR **Cross clamp use (%):** 100% **Cross clamp duration (mins):** NR **Duration of surgery (mins):** Aprotinin: median 100; Placebo: median 104 **Surgical approach/es:** open **Surgical pathology/ies:** Aprotinin: aneurysm repair elective (54%), bypass elective (26%), other 20%; Usual care: aneurysm repair elective (40%), bypass elective (32%), other 28% **Anatomical region/s:** NR (aortic)	
Interventions	**Standardised name:** Aprotinin: aprotinin; Placebo: placebo **Description in text:** Aprotinin: "aprotinin 0.05 MK before induction plus aprotinin 2 MK iv bolus to patient over 20 minutes at induction plus aprotinin 0.5 MK/h intravenous infusion to patient continuously (infusion was changed to 1 MK/h after 80 randomised patients)"; Placebo: placebo NS EV	
Outcomes	**Red cell transfusions at up to 30 days postsurgery:** Aprotinin: median 3 IQR 2 to 5, n = 66; Placebo: median 3 IQR 2 to 5, n = 62 **All‐cause mortality at up to 30 days;** Aprotinin: 4/68; Placebo: 6/64 **and at between 31 and 90 days:** NR **Risk of receiving any allogeneic blood product (composite) at up to 30 days postsurgery:** NR **Risk of receiving any allogeneic packed red cells at up to 30 days postsurgery:** NR **Risk of receiving any allogeneic platelets at up to 30 days postsurgery:** NR **Risk of receiving any allogeneic fresh frozen plasma at up to 30 days postsurgery:** NR **Risk of reoperation or repeat procedure for bleeding within 7 days:** NR **Risk of a thrombotic/thromboembolic event (any composite measured in study) (n/N):** NR **Risk of cerebrovascular attack (CVA/stroke) at up to 30 days:** Aprotinin: 1/68; Placebo: 0/64 **Risk of myocardial infarction (MI) at up to 30 days:** Aprotinin: 1/68; Placebo: 3/64 **Risk of deep vein thrombosis (DVT) at up to 90 days:** Aprotinin: 1/68; Placebo: 1/64 **Risk of pulmonary embolus (PE) at up to 90 days:** Aprotinin: 2/68; Placebo: 1/64 **Risk of a serious adverse event (SAE) at up to 30 days:** NR **Length of hospital stay (days):** NR **Cost data presented:** no	
Notes	**Sponsorship source:** pharmaceutical (Bayer). Two authors are employees of the manufacturer. **Country:** UK **Setting:** single‐centre **Trial primary outcome:** NR. However, lab data, blood losses in theatre and postoperatively, and adverse outcomes were reported. **Reference type:** full text **Translation into English required:** no **Trial registration number:** NR **Trial registration timing:** NA	
***Risk of bias***		
**Bias**	**Authors' judgement**	**Support for judgement**
Random sequence generation (selection bias)	Unclear risk	"prospective, randomized, double‐blind, placebo‐controlled trial." "Some 136 patients were randomized to receive either aprotinin, given as a loading dose of 2 X 10^6^ kallikrein inactivator (KI) units followed by 0.5 X 10^6^ KI units/h or equal volumes of 0.9 per cent saline." Method of sequence generation for randomisation not described.
Allocation concealment (selection bias)	Unclear risk	Method of allocation concealment not described.
Blinding of participants and personnel (performance bias) All outcomes	Low risk	"Randomization, in groups of four, was blinded from the investigators by the use of identical coded bottles containing active drug or placebo (0.9 per cent saline)." It is likely blinding was done adequately, because a placebo was used and the bottles was identical. However, not all details regarding blinding are clear. Subjective outcomes ‐ low risk of bias due to adequate personnel blinding. Objective outcomes ‐ low risk of bias.
Blinding of outcome assessment (detection bias) All outcomes	Low risk	It is likely blinding was done adequately, because a placebo was used and the bottles was identical. However, not all details regarding blinding are clear. Subjective outcomes ‐ low risk of bias due to adequate outcome assessor blinding. Objective outcomes ‐ low risk of bias.
Incomplete outcome data (attrition bias) All outcomes	Unclear risk	"Patients surviving to the first week were included in the primary data analysis but clinical follow‐up continued to the 30th day." "Four deaths occurred within 7 days of surgery (two in each group): one following massive haemorrhage, two from myocardial infarction and one from a pulmonary embolus." People who died within 7 days of surgery were excluded, which could have affected outcome. However, the distribution of these 4 excluded people was balanced (2 in each arm), so it is unclear whether or not this would have affected outcomes.
Selective reporting (reporting bias)	Unclear risk	No available prospective protocol or trial registration.
Other bias	Unclear risk	"After 80 patients had been randomized an independent analysis of the plasma levels achieved, without the investigators receiving code‐breaking information, suggested that the maintenance infusion dose should be increased to 1 X 10^6^ KI units/h in an effort to ensure that plasma levels were similar to those observed in cardiac studies." "Independent assay of the plasma levels of aprotinin achieved dictated a protocol change to reach levels similar to those observed in cardiac studies. The maintenance infusion dose was therefore doubled from 0.5 x 10^6^ KI units/ml/h (standard dose) to 1 X 10^6^ KI units/ml/h (high dose) to achieve this. The median intraoperative aprotinin plasma level was 93 (range 51‐222) KI unit/ml for those treated with the standard regimen and 146 (range 95‐334) KI units/ml for those who received the higher infusion dose (z = 4.15, P < 0.001). Patients treated with the initial standard aprotinin infusion or the higher dose were compared separately with controls; the results were similar to those seen with the whole cohort of patients but there was a dose‐related effect for blood loss on to swabs and for the early postoperative period." There was a change in the intervention to increase the infusion rate, and it is unclear whether this might have introduced bias.

**Table:** Robinson 2000

***Study characteristics***		
Methods	**Design:** RCT (parallel group) **Anticoagulation cessation:** NR **Antiplatelet cessation:** NR **Power calculation reached:** no, 50 in each arm calculated and 38/39 in each arm recruited. **Prophylactic or therapeutic randomisation:** prophylactic **Recruitment end date:** June 1998 **Recruitment start date:** December 1994 **Stopping rule:** NR **Strategy for analysis (ITT/PPA):** ITT: "No patient was withdrawn from the trial after surgery on the basis of any pre‐existing cardiac, renal or respiratory disease" **Transfusion strategy protocol:** no: "Most blood loss during surgery is related to obtaining control of the aorta and the subsequent anastomoses, while transfusion given after the patient reaches the intensive care unit is largely governed by physiological parameters and blood results. These are judged by individual anaesthetists and surgeons, and could not be prescribed within a trial protocol." **Transfusion threshold in trial:** NA **Trial stopped early:** yes, recruitment problems. "Because of the difficulty in obtaining informed consent, several local ethics committees refused to sanction the trial, which was begun before the introduction of the Multicentre Regional Ethics Committee (MREC) system".	
Participants	**Inclusion criteria:** "The inclusion criteria for the trial were the clinical diagnosis of a ruptured AAA, i.e. hypotensive collapse associated with a pulsatile abdominal mass, and the acquisition of written, informed consent when possible." **Exclusion criteria:** "Patients were excluded from the study if they had any previous exposure to aprotinin, known renal failure with a serum creatinine level greater than 150mmol/l, or known respiratory or cardiac failure requiring hospital admission within the previous year. Patients were to be withdrawn from the study if the AAA was not ruptured at laparotomy or if any significant pre‐existing renal, cardiac or respiratory failure was subsequently discovered." **Group differences:** no, "The incidence of risk factors in the two groups was similar." **Indirectness:** no **Protocol deviations:** NA, "Two were withdrawn at laparotomy because the AAA was found not to have ruptured" as per exclusion criteria. **Reasons for dropouts:** NA, Two were withdrawn because the AAA was found not to have ruptured. The latter criterion was part of the prespecified exclusion criteria. **Number of participants randomised:** Aprotinin: NR; Placebo: NR **Number of participants receiving treatment:** Aprotinin: 38; Placebo: 39 **Number of participants analysed:** Aprotinin: 38; Placebo: 39 **Number of participants dropping out:** 0 **Male gender (%):** Aprotinin: 79; Placebo: 92 **Age (years):** Aprotinin: median 74 range 56 to 88; Placebo: median 73 range 52 to 86 **Weight (kg):** NR **Ethnicity:** NR **Preoperative haemoglobin (g/L):** NR **Perioperative antiplatelet use (excluding aspirin) (%):** NR **Perioperative aspirin use (%):** NR **Perioperative anticoagulants (%):** NR **Revision surgery (%):** 0 **Elective surgery (%):** 0 **Risk score (system used & score):** NR **Cross clamp use (%):** 100% **Cross clamp duration (mins):** NR **Duration of surgery (mins):** NR **Surgical approach/es:** open **Surgical pathology/ies:** aneurysm repair urgent or emergent 100% **Anatomical region/s:** abdominal aortic	
Interventions	**Standardised name:** aprotinin: aprotinin 2MK to bolus to patient at induction plus aprotinin 1MK/hr iv infusion during surgery up to a maximum of 4MK; Placebo: placebo NS EV **Description in text:** aprotinin: "An intravenous bolus of aprotinin 2 x 10^6^ units (or similar volume of placebo) was administered as soon as possible after the patient reached theatre and a further dose of 0.5 x 10^6^ units (or placebo) was given every 30 min during the operation, to a maximum total dose of 4 x 10^6^ units"; Placebo: placebo NS EV	
Outcomes	**Red cell transfusions at up to 30 days postsurgery:** Aprotinin: median 10; range 2 to 29, n = 38; Placebo: median 14, range 4 to 38, n = 39 **All‐cause mortality at up to 30 days;** Aprotinin: 17/38; Placebo 17/39 **and at between 31 and 90 days:** NR **Risk of receiving any allogeneic blood product (composite) at up to 30 days postsurgery:** NR **Risk of receiving any allogeneic packed red cells at up to 30 days postsurgery:** NR **Risk of receiving any allogeneic platelets at up to 30 days postsurgery:** NR **Risk of receiving any allogeneic fresh frozen plasma at up to 30 days postsurgery:** NR **Risk of reoperation or repeat procedure for bleeding within 7 days:** NR **Risk of a thrombotic/thromboembolic event (any composite measured in study) (n/N):** NR **Risk of cerebrovascular attack (CVA/stroke) at up to 30 days:** aprotinin: 0/38; placebo: 3/39 **Risk of myocardial infarction (MI) at up to 30 days:** Aprotinin: 1/38; Placebo: 2/39 **Risk of deep vein thrombosis (DVT) at up to 90 days:** NR **Risk of pulmonary embolus (PE) at up to 90 days:** NR **Risk of a serious adverse event (SAE) at up to 30 days:** NR **Length of hospital stay (days):** Aprotinin: median 12; Placebo: median 15 **Cost data presented:** no	
Notes	**Sponsorship source:** pharmaceutical (Bayer supplied the study drug free of charge) **Country:** UK **Setting:** multicentre (9) **Trial primary outcome:** NR, Methods state: "The number of units of blood, fresh frozen plasma and platelets administered during the operation encompassing time of admission to completion of surgery) and in the first 12h following operation were recorded. The incidence of postoperative complications, including respiratory failure defined as requiring ventilation beyond 48h or reintubation, renal failure defined as requiring haemofiltration or haemodialysis, or death within 30 days was noted, as were the length of intensive care unit and hospital stay." **Reference type:** full text **Translation into English required:** no **Trial registration number:** NR **Trial registration timing:** NA	
***Risk of bias***		
**Bias**	**Authors' judgement**	**Support for judgement**
Random sequence generation (selection bias)	Low risk	"The randomization was in permuted blocks of ten, ensuring approximately equal numbers of each treatment within individual centres. Aprotinin or placebo was administered according to the randomization list drawn up by the pharmacist; the code for determining the contents of each bottle remained with the pharmacist until the end of the trial." Exact method of sequence generation unclear but probably low risk as conducted centrally at pharmaceutical company using code and permuted blocks of 10
Allocation concealment (selection bias)	Low risk	"Bayer PLC (Newbury, UK) supplied the aprotinin and a single pharmacist undertook the preparation of all samples in the sterile production facility at the Royal Hallamshire Hospital (Sheffield, UK). Bottles were prepared containing either 2 3 10 6 units of aprotinin in 200 ml saline or 0´5 3 10 6 units of aprotinin in 50 ml saline. Identical bottles of sterile 0´9 per cent saline were also prepared. Bottles were numbered and sent to the participating centres, where they were kept in the theatre fridge. The randomization was in permuted blocks of ten, ensuring approximately equal numbers of each treatment within individual centres. Aprotinin or placebo was administered according to the randomization list drawn up by the pharmacist; the code for determining the contents of each bottle remained with the pharmacist until the end of the trial." Adequate method of central allocation concealment by pharmacy.
Blinding of participants and personnel (performance bias) All outcomes	Low risk	Study report does not state that it was double‐blind, but methodology described suggests that it was adequately blinded. Subjective outcomes ‐ low risk of bias due to adequate personnel blinding. Objective outcomes ‐ low risk of bias.
Blinding of outcome assessment (detection bias) All outcomes	Low risk	Subjective outcomes ‐ low risk of bias due to adequate outcome assessor blinding. Objective outcomes ‐ low risk of bias.
Incomplete outcome data (attrition bias) All outcomes	Low risk	No obvious outcome data missing.
Selective reporting (reporting bias)	Unclear risk	No available prospective protocol or trial registration.
Other bias	High risk	The trial was stopped before the number of participants in each trial was reached, and the article does not really explain why this was done.

**Table:** Taylor 2003

***Study characteristics***		
Methods	**Design:** RCT (parallel group) **Anticoagulation cessation:** "Patients received heparin (70 IU/kg) before arterial clamping." **Antiplatelet cessation:** no, aspirin used preoperatively. "All subjects were given or continued to receive preoperative aspirin therapy". **Power calculation reached:** yes, "The primary study end point, that is, hemostasis within4 minutes of randomization, and all secondary efficacy endpoints were analyzed with the intention‐to‐treat method. The analysis was limited to subjects included in the main study. A sample size of 200 randomized subjects was chosen at the investigators’ planning meeting, based on an estimated treatment effect of 25%. The two‐sided 95% CI for the common odds ratio stratified by surgeon for treatment success, that is, hemostasis achieved within 4 minutes, and the corresponding P value were obtained by exact inference using the statistical package StatXact 4.0." **Prophylactic or therapeutic randomisation:** prophylactic **Recruitment end date:** NR **Recruitment start date:** NR **Stopping rule:** NR **Strategy for analysis (ITT/PPA):** modified‐ITT "Data for 1 of 201 randomized subjects was excluded from intention‐to‐ treat analysis because surgery was aborted before completion of the vascular graft." **Transfusion strategy protocol:** NR **Transfusion threshold in trial:** NA **Trial stopped early:** no	
Participants	**Inclusion criteria: "**Male or non pregnant, non lactating female patients older than 18 years were eligible for study inclusion if they were scheduled to undergo elective PTFE grafting including at least one end‐to‐side anastomosis of a PTFE graft to the common femoral artery and could undergo heparinization during the period of arterial occlusion." "All subjects were given or continued to receive preoperative aspirin therapy." "Patients received heparin (70 IU/kg) before arterial clamping." "For operations with more than one anastomosis, the study anastomosis was the last one completed." **Exclusion criteria:** NR **Group differences:** no, "Demographic characteristics of the randomized subjects are compared in Table I. There were no significant differences between groups." **Indirectness:** no **Protocol deviations:** NA **Reasons for dropouts:** "Data for 1 of 201 randomized subjects was excluded from intention‐to‐ treat analysis because surgery was aborted before completion of the vascular graft." **Number of participants randomised:** Fibrin sealant: NR; Gelatin sponge: NR **Number of participants receiving treatment:** Fibrin sealant: 101; Gelatin sponge: 99 **Number of participants analysed:** Fibrin sealant: 101; Gelatin sponge: 99 **Number of participants dropping out:** NR **Male gender (%):** Fibrin sealant: 63%; Gelatin sponge: 62% **Age (years):** Fibrin sealant: mean 63; Gelatin sponge: mean 65 **Weight (kg):** Fibrin sealant: mean 78; Gelatin sponge: mean 74 **Ethnicity:** Fibrin sealant: White 87%; Gelatin sponge: White 82% **Preoperative haemoglobin (g/L):** NR **Perioperative antiplatelet use (excluding aspirin) (%):** NR **Perioperative aspirin use (%):** Fibrin sealant: 84%; Gelatin sponge: 87% **Perioperative anticoagulants (%):** Fibrin sealant: heparin 99; Gelatin sponge: heparin 99 **Revision surgery (%):** NR **Elective surgery (%):** 100% **Risk score (system used & score):** NR **Cross clamp use (%):** 100% **Cross clamp duration (mins):** NR **Duration of surgery (mins):** NR **Surgical approach/es:** open **Surgical pathology/ies:** extruded polytetrafluoroethylene graft (PRFE) after anastomosis to the femoral artery **Anatomical region/s:** femoral	
Interventions	**Standardised name:** Fibrin sealant: Fibrin sealant Beriplast: fibrinogen, thrombin, fibrin stabilising factor XIII aprotinin and calcium chloride; Gelatin sponge: thrombin‐soaked gelatin sponge (TSG) **Description in text:** Fibrin sealant: "The fibrin sealant Beriplast P (FSBP; AventisBehring, Strasbourg, France) includes fibrinogen, thrombin, fibrin stabilizing factor XIII, aprotinin, and calcium chloride; Gelatin sponge: thrombin‐soaked gelatin sponge (TSG)"; Gelatin sponge: "thrombin‐soaked gelatin sponge (TSG)"	
Outcomes	**Red cell transfusions at up to 30 days postsurgery:** NR **All‐cause mortality at up to 30 days;** Fibrin sealant: 2/100; Gelatin sponge: 4/99 **and at between 31 and 90 days:** NR **Risk of receiving any allogeneic blood product (composite) at up to 30 days postsurgery:** NR **Risk of receiving any allogeneic packed red cells at up to 30 days postsurgery:** NR **Risk of receiving any allogeneic platelets at up to 30 days postsurgery:** NR **Risk of receiving any allogeneic fresh frozen plasma at up to 30 days postsurgery:** NR **Risk of reoperation or repeat procedure for bleeding within 7 days:** NR **Risk of a thrombotic/thromboembolic event (any composite measured in study) (n/N):** NR **Risk of cerebrovascular attack (CVA/stroke) at up to 30 days:** NR **Risk of myocardial infarction (MI) at up to 30 days:** NR **Risk of deep vein thrombosis (DVT) at up to 90 days:** NR **Risk of pulmonary embolus (PE) at up to 90 days:** NR **Risk of a serious adverse event (SAE) at up to 30 days:** NR **Length of hospital stay (days):** NR **Cost data presented:** no	
Notes	**Sponsorship source:** pharmaceutical (Avenis‐Behring) Employee of the manufacturer on authorship and consulting fees to first author. **Country:** USA **Setting:** multicentre (26) **Trial primary outcome:** "time from randomisation (not time from treatment application or time from clamp release) to haemostasis." **Reference type:** full text **Translation into English required:** no **Trial registration number:** NR **Trial registration timing:** NA	
***Risk of bias***		
**Bias**	**Authors' judgement**	**Support for judgement**
Random sequence generation (selection bias)	Unclear risk	"This was a prospective multicenter randomized (FSBP‐ TSG ratio, 1:1) single‐blinded therapeutic trial conducted at 26 medical centers in the United States." "Because this was a single‐blinded study (study personnel could not be blinded to the nature of the experimental vs control treatment), randomization was delayed until all possible study procedures were completed." Method of sequence generation for randomisation not described.
Allocation concealment (selection bias)	Unclear risk	"The arterial clamps were then reapplied, and the randomization envelope was opened" Envelopes were used, but no further details supplied.
Blinding of participants and personnel (performance bias) All outcomes	High risk	"Because this was a single‐blinded study (study personnel could not be blinded to the nature of the experimental vs control treatment), randomization was delayed until all possible study procedures were completed." Unblinded study. Subjective outcomes ‐ high risk of bias due to inadequate personnel blinding. Objective outcomes ‐ low risk of bias regardless.
Blinding of outcome assessment (detection bias) All outcomes	High risk	Subjective outcomes ‐ high risk of bias due to inadequate outcome assessor blinding. Objective outcomes ‐ low risk of bias.
Incomplete outcome data (attrition bias) All outcomes	Low risk	No obvious outcome data missing, except for one participant randomised to fibrin sealant.
Selective reporting (reporting bias)	Unclear risk	No available prospective protocol or trial registration.
Other bias	Low risk	No other concerns such as early stopping or imbalanced study arms.

**Table:** Weaver 2002

***Study characteristics***		
Methods	**Design:** RCT (parallel group) **Anticoagulation cessation:** no, 74/89 received intraoperative heparin. Floseal: 28/43 received either anticoagulation or antiplatelet; Gelfoam: 31/46 received either anticoagulation or antiplatelet **Antiplatelet cessation:** no, Floseal: 28/43 received either anticoagulation or antiplatelet; Gelfoam: 31/46 received either anticoagulation or antiplatelet **Power calculation reached:** NR **Prophylactic or therapeutic randomisation:** therapeutic **Recruitment end date:** NR **Recruitment start date:** NR **Stopping rule:** NR **Strategy for analysis (ITT/PPA):** ITT **Transfusion strategy protocol:** NR **Transfusion threshold in trial:** NA **Trial stopped early:** no	
Participants	**Inclusion criteria:** "patients 21 years or older who were undergoing reconstructive vascular surgery or arteriovenous access procedures and who were willing and able to complete all follow‐up visits" **Exclusion criteria:** "patients were excluded if they were pregnant or had a known sensitivity to any components of bovine thrombin preparations and/or any material of bovine origin." **Group differences:** no obvious differences in characteristics reported (age, antiplatelet or anticoagulant use, heparin or protamine doses), though surgical mix has some differences, for example, more femoral bypass than carotid endarterectomy or AV fistula formation in Gelfoam thrombin group vs FloSeal Matric. **Indirectness:** no **Protocol deviations:** NA **Reasons for dropouts:** NA **Number of participants randomised:** Floseal: 43; Gelfoam: 46 **Number of participants receiving treatment:** Floseal: 43; Gelfoam: 46 **Number of participants analysed:** Floseal: 43; Gelfoam: 46 **Number of participants dropping out:** 0 **Male gender (%):** NR **Age (years):** Floseal: mean 68; Gelfoam: mean 63 **Weight (kg):** NR **Ethnicity:** NR **Preoperative haemoglobin (g/L):** NR **Perioperative antiplatelet use (excluding aspirin) (%):** Floseal: 65% were on an anticoagulant and or antiplatelet drug; Gelfoam: 67% were on an anticoagulant and or antiplatelet drug **Perioperative aspirin use (%):** Floseal: 65% were on an anticoagulant and or antiplatelet drug; Gelfoam: 67% were on an anticoagulant and or antiplatelet drug **Perioperative anticoagulants (%):** Floseal: 65% were on an anticoagulant and or antiplatelet drug; Gelfoam: 67% were on an anticoagulant and or antiplatelet drug **Revision surgery (%):** NR **Elective surgery (%):** NR **Risk score (system used & score):** NR **Cross clamp use (%):** NR **Cross clamp duration (mins):** NR **Duration of surgery (mins):** NR **Surgical approach/es:** open **Surgical pathology/ies:** bypass, endarterectomy, aneurysm **Anatomical region/s:** various anatomical regions	
Interventions	**Standardised name:** Floseal: FloSeal (glutaraldehyde cross‐linked gelatin with thrombin); Gelfoam: Gelfoam soaked in thrombin **Description in text:** Floseal: "FloSeal (glutaraldehyde cross linked gelatin with thrombin)"; Gelfoam: NA	
Outcomes	**Red cell transfusions at up to 30 days postsurgery:** NR **All‐cause mortality at up to 30 days:** Floseal: 0/43; Gelfoam: 5/46 **and at between 31 and 90 days:** NR **Risk of receiving any allogeneic blood product (composite) at up to 30 days postsurgery:** NR **Risk of receiving any allogeneic packed red cells at up to 30 days postsurgery:** NR **Risk of receiving any allogeneic platelets at up to 30 days postsurgery:** NR **Risk of receiving any allogeneic fresh frozen plasma at up to 30 days postsurgery:** NR **Risk of reoperation or repeat procedure for bleeding within 7 days:** Floseal: 0/43; Gelfoam: 1/46 **Risk of a thrombotic/thromboembolic event (any composite measured in study) (n/N):** Floseal: 3/43; Gelfoam: 9/46 **Risk of cerebrovascular attack (CVA/stroke) at up to 30 days:** NR **Risk of myocardial infarction (MI) at up to 30 days:** NR **Risk of deep vein thrombosis (DVT) at up to 90 days:** NR **Risk of pulmonary embolus (PE) at up to 90 days:** NR **Risk of a serious adverse event (SAE) at up to 30 days:** NR **Length of hospital stay (days):** NR **Cost data presented:** no	
Notes	**Sponsorship source:** pharmaceutical (Fusion Medical Technology) No statement of independence of authors **Country:** USA **Setting:** multicentre (4) **Trial primary outcome:** cessation of bleeding within 10 minutes; secondary end point: outcomes of additional treated bleeding sites and time to cessation of bleeding **Reference type:** full‐text **Translation into English required:** no **Trial registration number:** NR **Trial registration timing:** NA	
***Risk of bias***		
**Bias**	**Authors' judgement**	**Support for judgement**
Random sequence generation (selection bias)	Unclear risk	The article says "randomised", but gives no further details. Method of sequence generation for randomisation not described.
Allocation concealment (selection bias)	Unclear risk	Method of allocation concealment not described.
Blinding of participants and personnel (performance bias) All outcomes	High risk	Blinding of personnel to the arms not possible. Subjective outcomes ‐ high risk of bias due to inadequate or absent personnel blinding. Objective outcomes ‐ low risk of bias regardless.
Blinding of outcome assessment (detection bias) All outcomes	High risk	Subjective outcomes ‐ unclear risk of bias due to unclear adequacy of outcome assessor blinding. Objective outcomes ‐ low risk of bias.
Incomplete outcome data (attrition bias) All outcomes	Low risk	No obvious outcome data missing.
Selective reporting (reporting bias)	Unclear risk	No available prospective protocol or trial registration.
Other bias	Unclear risk	Only a few baseline characteristics provided. Insufficient information to assess whether an important risk of bias exists.

AAA ‐ abdominal aortic aneurysm; AE ‐ adverse event; AV ‐ arteriovenous; CAD ‐ coronary artery disease; COPD ‐ chronic obstructive pulmonary disease; CVA ‐ cerebrovascular event; DDAVP ‐ Desmopressin acetate 1‐deamino‐8‐D‐arginine vasopressin; DM ‐ diabetes mellitis; DVT ‐ deep vein thrombosis; ITT ‐ intention‐to‐treat; MI ‐ myocardial infarction; mITT ‐ modified intention‐to‐treat; NA ‐ not applicable; NaCL: sodium chloride; NR ‐ not reported; NS EV ‐ normal saline equal volume; OSR ‐ open surgical repair; PE ‐ pulmonary embolism; PPA ‐ per protocol analysis; PTFE ‐ polytetrafluoroethylene; RCT ‐ randomised controlled trial; RoB ‐ risk of bias; SD ‐ standard deviation; TBS ‐ target bleeding site; TTH ‐ time to haemostasis; TXA ‐ tranexamic acid; SAE ‐ serious adverse event;

### Characteristics of excluded studies [ordered by study ID]

**Table:**

Study	Reason for exclusion
Abiko 2022	Ineligible participant population
Amin 2000	Ineligible intervention ‐ femoral vascular access closure device or dressing
Baum 2002	Ineligible intervention
Bedirhan 2001	Ineligible participant population ‐ thoracic surgery
Brunkwall 2007	Ineligible study design ‐ single arm study, so participants were not randomised to interventions
Casati 2002	Ineligible participant population ‐ cardiac surgery
Cebrian 2017	Ineligible intervention
Chalmers 2010	Ineligible participant population ‐ mixed population, < 80% receiving included intervention
Chapman 2001	Ineligible participant population
Chapman 2006	Ineligible participant population ‐ mixed surgical population with no vascular subgroup results available
Chapman 2007	Ineligible participant population ‐ mixed surgical population with no vascular subgroup results provided.
ChiCTR1900021586	Ineligible intervention ‐ femoral artery puncture
Christenson 2004	Ineligible participant population ‐ cardiac surgery
Clagett 1996	Not relevant (review)
Coselli 2003	Ineligible participant population ‐ no vascular subgroup available
Croxtall 2009	Ineligible participant population
CTRI/2013/04/003580	Ineligible participant population
CTRI/2020/05/025285	Ineligible intervention ‐ femoral artery puncture
CTRI/2021/09/036977	Ineligible participant population
Develle 2020	Ineligible participant population ‐ < 80% of patients receiving included intervention; no subgroup information provided
Devereaux 2008	Ineligible intervention
Donker 2012	Ineligible intervention
Doria 2008	Ineligible participant population
Eder 1986	Ineligible participant population
Ehrlich 1998	Ineligible participant population ‐ cardiac surgery
EUCTR‐2007‐004612‐DE	Ineligible participant population
EUCTR‐2013‐003464‐31‐GB	Ineligible participant population ‐ primarily cardiac surgery
EUCTR‐2016‐001126‐33‐GB	Ineligible participant population: no vascular subgroup available
Fletcher 1995	Ineligible intervention ‐ "Patients having femoro‐femoral, axillo‐femoral and femoro‐popliteal bypass with prosthetic vascular graft were randomized to receive either PTFE or Gelsoft with or without rifampicin soaking of the vascular graft prior to insertion."
Glickman 2002	Ineligible participant population ‐ mixed population, < 80% receiving included intervention
Glineur 2018	Ineligible participant population ‐ primarily cardiac with no vascular subgroup available
Hagberg 2004	Ineligible participant population ‐ predominantly cardiac surgery
Hallak 2007	Ineligible intervention ‐ vascular access dressing/device
Hanks 2003	Ineligible participant population ‐ no vascular subgroup available
Hongo 2021	Ineligible intervention
IRCT2017030432858N1	Ineligible participant population
Khoynezhad 2018	Ineligible participant population ‐ cardiac surgery
Kim 2006	Ineligible intervention ‐ collagen plug compared to suture mediated vascular access closure
Klein‐Wiele 2018	Ineligible intervention ‐ clip‐based compared to suture mediated vascular closure for femoral access haemostasis
Koncar 2008	Ineligible study design ‐ used historic controls
Koncar 2011	Ineligible study design ‐ used historic controls
Lumsden 2006	Ineligible participant population ‐ mixed population, < 80% receiving included intervention
Makhija 2013	Ineligible participant population ‐ cardiac surgery
Minato 2009	Ineligible participant population ‐ cardiac surgery
Morita 2013	Ineligible participant population ‐ cardiac surgery
Morita 2020	Ineligible participant population ‐ cardiac surgery
Morrison 2019	Ineligible comparator ‐ comparator is FFP, not placebo or usual care
Nasso 2009	Ineligible participant population ‐ cardiac surgery
NCT00388284	Ineligible participant population ‐ peripheral vascular access
NCT00428155	Ineligible intervention ‐ arterial closure device
NCT00439309	Ineligible participant population ‐ mixed population, < 80% receiving included intervention
NCT00440401	Ineligible participant population ‐ cardiac surgery
NCT00701142	Ineligible participant population
NCT01500135	Ineligible participant population ‐ mixed population, < 80% receiving included intervention
NCT01669382	Ineligible intervention ‐ study compares arteriotomy closure devices used in percutaneous coronary intervention
NCT01681030	Ineligible participant population ‐ cardiac surgery remit
NCT01879475	Ineligible participant population ‐ cardiac surgery
NCT01959503	Ineligible participant population ‐ cardiac surgery
NCT02540434	Ineligible participant population ‐ cardiac surgery
NCT02640235	Ineligible participant population ‐ mixed population with no vascular subgroup available
NCT03173703	Ineligible intervention
NCT03176225	Ineligible intervention and participant population ‐ this study compared XenoSure biologic patch with a polyester patch in cardiac surgery
NCT03369977	Ineligible participant population ‐ cardiac surgery
NCT03426839	Ineligible intervention ‐ this study compared haemostasis strategy guided by conventional coagulation tests with transfusion algorithm guided by viscoelastic POC tests and algorithms.
NCT03444324	Ineligible participant population ‐ spinal surgery
NCT03558243	Ineligible participant population ‐ radial access
NCT03654560	Ineligible participant population ‐ no vascular subgroup available
NCT03917862	Ineligible participant population ‐ cardiac surgery
Okita 1996	Ineligible participant population ‐ cardiac surgery
Park 2005	Ineligible intervention ‐ a study of vascular access devices for percutaneous procedures
Pathan 2021	Ineligible participant population
Pilon 2010	Ineligible study design ‐ this was not a randomised study
Rahe‐Meyer 2009	Ineligible study design and participant population ‐ study appears to be cardiac surgery population and is not randomised
Rahe‐Meyer 2013	Ineligible participant population ‐ cardiac surgery remit
Rahe‐Meyer 2016	Ineligible participant population ‐ cardiac surgery remit
Rastan 2008	Ineligible intervention ‐ vascular access closure devices
Ratnam 2007	Ineligible intervention ‐ vascular access closure devices
Saha 2012	Ineligible participant population ‐ mixed population, < 80% receiving included intervention
Sanborn 1993	Ineligible intervention ‐ vascular access closure devices
Sauer 2016	Ineligible study design ‐ study does not appear to be randomised
Scheinert 2007	Ineligible intervention ‐ vascular access closure surface sealant
Schenk 2003	Ineligible participant population ‐ all participants undergoing vascular access surgery
Schwarz 2009	Ineligible intervention ‐ vascular access closure devices
Shimamura 1998	Ineligible study design ‐ participants not randomised
Sirlak 2003	Ineligible participant population ‐ cardiac surgery
Stone 2012	Ineligible participant population ‐ mixed population, < 80% receiving included intervention
Takaori 1995	Ineligible participant population ‐ cardiac surgery
Tepe 2008	Ineligible intervention ‐ vascular access closure device
Thompson 2013	Not relevant (review article)
Verhoef 2015	Ineligible participant population ‐ mixed population, < 80% receiving included intervention
Vierhout 2014	Ineligible intervention ‐ skin sealant
Wachol‐Drewek 1996	Ineligible intervention
Weaver 2008	Ineligible participant population: 46% of participants undergoing excluded vascular procedures at low risk of bleeding
Zwischenberger 1999	Ineligible participant population

FFP ‐ fresh frozen plasma; POC ‐ point of care; PTFE ‐ polytetrafluoroethylene

### Characteristics of studies awaiting classification [ordered by study ID]

**Table:** jRCTs041180163

Methods	Variously described as RCT and historic control
Participants	**Inclusion criteria:** people who undergo first bifurcated stent graft treatment for abdominal aortic aneurysm and iliac artery aneurysm at Aichi Medical University Hospital **Exclusion criteria:** people who cannot take internal medicine
Interventions	**Tranexamic acid group:** tranexamic acid 250 mg*3/day given on day 1 to 30 after EVAR **Non Tranexamic acid group:** do not give any additional drugs
Outcomes	**Primary outcome:** incidence of type 2 endoleak **Secondary outcome:** changes in the size of aneurysms after EVAR
Notes	Unclear if control participants are randomised, or if historic control is used. Trial registration jRCTs041180163, first posted: 29 March 2019 Actual study start date: 20 May 2017 Estimated primary completion date: NR Estimated study completion date: NR

**Table:** NCT00618358

Methods	RCT, parallel group
Participants	**Inclusion criteria:** Adults Scheduled for elective vascular surgery that entails placement of a PTFE vascular graft including extra‐anatomic, infrainguinal bypass and primary and secondary arteriovenous access procedures Subject is willing and able to comply with all aspects of the treatment and evaluation schedule; Informed of the nature of the study, and has provided written informed consent, approved by the appropriate Institutional Review Board of the respective clinical site Intraoperative inclusion criteria: subjects must meet the following intraoperative inclusion criteria to be eligible for randomisation: Suture line leaks (bleeding) confirmed prior to randomisation **Exclusion criteria:** Preoperative exclusion criteria: subjects who meet any of the following criteria are not eligible for participation in the study: Subject has a known local or systemic infection Subjects with known coagulopathies including haemophilia, factor deficiencies, platelet count < 80,000 u/mL, heparin induced thrombocytopenia or uncorrected INR > 1.5 Subject is participating in a clinical trial that requires treatment with another investigational device or drug Subject is lactating or pregnant, or does not agree to use contraception for the duration of the study Subject has a known hypersensitivity to any components of bovine thrombin preparations and/or material of bovine origin The investigator determines that the subject should not be included in the study for reason(s) not already specified Intraoperative exclusion criteria: subjects who meet any of the following intraoperative exclusion criteria are considered screening failures and are not eligible to be randomised: Incidental finding of any of the preoperative exclusion criteria Subject has obvious contamination or a concurrent systemic infection Investigator determines that participation in the study may jeopardize the safety or welfare of the subject
Interventions	**Experimental:** the Vascular Sealant System is intended for use in vascular reconstructions to achieve adjunctive haemostasis by mechanically sealing areas of leakage **Comparator:** Gelfoam/Thrombin
Outcomes	NR
Notes	Trial terminated by sponsor. No results available Trial registration (NCT00618358), first posted: 20 February 2008 Actual study start date: March 2007 Estimated study completion date: terminated, "(Sponsor ‐ Confluent Surgical terminated study re: surgical techniques Letter dated 4/31/2008)"

**Table:** NCT00652314

Methods	RCT, parallel group
Participants	**Inclusion criteria:** The subject is 18 years of age or older The subject is undergoing an orthopaedic/spinal, general, cardiac, hepatic, or vascular surgical procedure (neurosurgical, ophthalmic or urological procedures must be excluded) The subject is willing and able to provide appropriate informed consent The subject is willing and able to comply with the requirements of the study protocol, including the predefined follow‐up evaluations Inclusion criteria to be determined during the surgical procedure: The subject has an intraoperative bleeding site which the surgeon is unable or unwilling to easily control with conventional methods (cautery, sutures) **Exclusion criteria:** The subject is known or suspected to be pregnant (verified in a manner consistent with institution's standard of care), or is lactating The subject has a known allergy to bovine derived products or any other materials used in the Thrombi‐Gel product The subject has an active infection at the surgical site The use of haemostatic agents are contraindicated for the subject The subject has a known bleeding disorder (including thrombocytopaenia (< 100,000 platelet count), thrombobasthenia, haemophilia, or von Willebrand disease) The subject has received antibiotic solutions/powders at the intended application site The subject has had surgery at the intended application site ≤ 6 months before the current surgical procedure The subject is unavailable for follow‐up The subject is currently participating in another investigational device or drug trial The subject has previously participated in this trial (Protocol 0307) or the Thrombi‐Paste trial (Protocol 0507)
Interventions	**Experimental:** Thrombi‐Gel **Control:** Gelfoam
Outcomes	**Primary outcome:** Time to haemostasis ("If hemostasis was not observed within 10 minutes, the treatment site was to be monitored and the research teams were asked to record the specific number of minutes until hemostasis was observed.") **Secondary outcomes:** Effectiveness: device success (defined as the number of subjects with first bleeding site applications for which haemostasis was obtained within 6 minutes of study device application without the need for adjunctive treatment) (time frame: procedure, up to 6 minutes post procedure) Effectiveness: haemostatic handling characteristics (Surgeon's Questionnaire) (time frame: procedure (application through end of procedure)) Ease of application to bleeding site as assessed by surgeon questionnaire for haemostatic handling characteristics Safety: incidence rate of device‐related adverse events (time frame: procedure, up to 60 days post procedure) Safety: immunological testing for Factor Va antibodies and coagulation parameters (time frame: 0 day, 30 day, and 60 days post procedure)
Notes	Awaiting full publication in case a vascular subgroup can be identified. Trial registration NCT00652314, first posted: 3 April 2008 Actual study start date: March 2008 Actual primary completion date: October 2009 Actual completion date: January 2010

**Table:** NCT04083807

Methods	RCT, parallel group
Participants	**Inclusion criteria:** Signed informed consent Male or female ≥ 18 ages Patients undergoing primary vascular surgery (i.e., conduit placement with an ePTFE graft), including the following: Arterio‐arterial‐bypass Ilio‐femoral bypass Femoro‐femoral bypass Ilio‐popliteal bypass Femoro‐popliteal bypass Femoro‐tibial vessel bypass Arteriovenous shunting for dialysis access in the upper or lower extremity Intraoperative inclusion criterion: Suture line bleeding eligible for study treatment is present after surgical haemostasis **Exclusion criteria:** Concurrent participation in another clinical study treatment with another investigational drug or device within last 30 days Other vascular procedures during the same surgical session Arterio‐arterial bypasses with more than two anastomoses Haemoglobin < 9.0 g/dL at screening Pregnant or lactating women Congenital or acquired coagulation disorders Prior kidney transplantation Heparin‐induced thrombocytopaenia Known prior exposure to aprotinin within the last 12 months Known hypersensitivity to aprotinin, heparin, blood products or other components of the investigational product Unwilling to receive blood products Known severe congenital or acquired immunodeficiency Prior radiation therapy to the operating field Severe local inflammation at the operating field Positive results of any of the following the blood tests: HIV, syphilis, hepatitis B, hepatitis C Emergency surgery Alcohol or drug abuse **Intraoperative exclusion criteria:** Major intraoperative complications that required resuscitation or deviation from the planned surgical procedure Intraoperative change in planned surgical procedure, which resulted in patient no longer meeting preoperative inclusion criteria or having preoperative exclusion criteria
Interventions	**Experimental:** TISSEEL Lyo, applied once intra‐operatively to the study suture line using the DUPLOJECT Fibrin Sealant Preparation and Application System. **Control:** manual compression with surgical gauze pads
Outcomes	**Primary outcome:** number of participants achieving haemostasis at 4 minutes after treatment (time frame: day 0 (4 minutes post‐treatment to closure of surgical wound)) **Secondary outcomes:** Number of participants achieving haemostasis at 6 minutes after treatment (time frame: day 0 (6 minutes post‐treatment to closure of surgical wound)) Number of participants achieving haemostasis at 10 minutes after treatment (time frame: day 0 (10 minutes post‐treatment to closure of surgical wound)) Number of participants with intraoperative re‐bleeding after haemostasis (time frame: day 0 (intraoperative)) Number of participants with postoperative re‐bleeding after haemostasis (time frame: day 1 (postoperative))
Notes	Trial completed October 2019 but no results posted Trial registration NCT04083807, first posted: 10 September 2019 Actual study start date: 10 July 2019 Actual primary completion date: 22 October 2019 Actual study completion date: 22 October 2019

EVAR ‐ endovascular aneurysm repair; HIV ‐ human immunodeficiency disease; INR ‐ international normalised ratio; NR ‐ not reported; PTFE ‐ polytetrafluoroethylene; RCT ‐ randomised controlled trial

### Characteristics of ongoing studies [ordered by study ID]

**Table:** ChiCTR1900023323

Study name	Fibrin Sealant Used in EVAR as Filling Technique Treating Abdominal Aortic Aneurysm
Methods	Single blinded RCT (parallel group)
Participants	Male or female aged from 18 to 80 Diagnosed with abdominal aortic aneurysm Requiring endovascular aneurysm repair
Interventions	Intervention: Fibrin sealant Control: usual care
Outcomes	Arterial disease mortality Reintervention rate
Starting date	3 June 2019
Contact information	Lu Qing‐sheng, Changhai hospital, 168 Changhai Road, Yangpu District, Shanghai, China; luqs@xueguan.net
Notes	Sponsor: self‐funded Recruitment status: not yet recruiting Planned recruitment: 500 Estimated primary completion date: NR Estimated study completion date: NR First posted: 22 May 2019 Last update posted: 22 May 2019 (checked 27 June 2022)

**Table:** NCT04803747

Study name	A Trial of a Hospital Policy of Tranexamic Acid Use to Reduce Transfusion in Major Non‐cardiac Surgery (TRACTION)
Methods	TRACTION is a pragmatic, multicentre, randomised, registry‐based cluster‐crossover trial. Patients, clinicians and investigators are blinded to treatment allocation
Participants	Adults Undergoing major non‐cardiac surgery Inpatient surgeries with an estimated ≥ 5% risk of RBC transfusion, including open surgeries or laparoscopic surgeries with an estimated duration of ≥ 3 hours
Interventions	"TXA 1 gram bolus (2 grams for patients over 100 kg) intravenously (IV) administered within 10 minutes of the first surgical incision, followed by 1 additional gram given intravenously at 2 ‐ 4 hours of surgery or prior to skin closure, at the discretion of the anaesthesiologist (e.g. IV bolus at 2 ‐ 4 hours of surgery, at skin closure, or the 1 additional gram given as a continuous infusion throughout the surgical procedure)." "Placebo (0.9% normal saline (1 gram bolus (2 grams for patients over 100 kg) intravenously (IV) administered within 10 minutes of the first surgical incision, followed by 1 additional gram given intravenously at 2‐4 hours of surgery or prior to skin closure, at the discretion of the anaesthesiologist (e.g. IV bolus at 2‐4 hours of surgery, at skin closure, or the 1 additional gram given as a continuous infusion throughout the surgical procedure)."
Outcomes	Primary outcome measures : Proportion of RBC transfusions Incidence of DVT or PE (collectively called venous thromboembolism (VTE) Secondary outcome measures : Transfused units (number of RBC units transfused (both at cluster level and patient level) Arterial or Venous thrombotic events (secondary safety outcomes include the in‐hospital diagnosis of myocardial infarction, stroke, deep vein thrombosis or pulmonary embolus) Hospital length of stay Number of participants requiring ICU admission 3‐month survival Number of days at home to day 30 Compliance
Starting date	16 February 2022
Contact information	Dayna Solvason, University of Manitoba‐ HSC Campus, 204‐792‐3372, dsolvason@wrha.mb.ca
Notes	Sponsor: University of Manitoba Recruitment status: recruiting Planned recruitment: 8320 Estimated primary completion date: 16 February 2023 Estimated study completion date: 15 April 2023 First posted: 18 March 2021 Last update posted: 7 April 2022 (checked 28 June 2022)

EVAR ‐ endovascular aneurysm repair; NR ‐ not reported; RBC ‐ red blood cells; RCT ‐ randomised controlled trial; TXA ‐ tranexamic acid

## Differences between protocol and review

We had planned to perform a network meta‐analysis (NMA) but were unable to do so due to the large number of treatments with very little data to populate the network. The planned NMA methods are outlined in Beverly 2020.

The protocol states the objective as: "To assess the efficacy and safety of anti‐fibrinolytic and haemostatic drugs and agents in reducing bleeding and transfusion in people undergoing major vascular surgery or vascular procedures with a risk of moderate or severe (> 500 mL) blood loss." This is still our objective, but we have changed "reducing bleeding and transfusion" to "reducing bleeding and the need for blood transfusion" because intra‐surgical bleeding outcomes were rejected at the protocol review stage, and we have not included any direct bleeding outcomes. A large proportion of the trials we identified had 10‐minute follow‐up with intra‐surgical bleeding outcomes only, so we have tightened up the wording to avoid ambiguity over our objectives.

The secondary endpoint of composite thromboembolic events (TE) was reported by very few trials and cannot be reconstructed from reports of specific thromboembolic events (MI, CVA/stroke, DVT, PE) because we cannot tell how many individuals experienced more than one TE. We have reported each of these events separately alongside the composite TE endpoint (where reported).

In the protocol we stated: "We used the International Conference on Harmonisation Good Clinical Practice definition of SAEs (ICH GCP 2018). Where studies reported an SAE outcome using an alternative definition, we recorded what definition of SAE was used and discussed suitability for inclusion in analysis with an expert panel, prior to extracting outcome data." This was overly ambitious. We extracted SAE data as reported by the authors, with very few making any statement about what definition of SAEs they were reporting.

In the protocol we stated: "We will do this after extracting participant, methodology and intervention data but prior to extracting outcome data, to avoid introducing bias. This will therefore be blinded in so far as authors and the panel will not have access to the results of data extraction at this point, though some may have some incidental familiarity with the literature. This two stage approach was devised with experts in the area of complex systematic reviews at the National Institute of Healthcare Research (UK) Complex Review Support Unit. In this way we aim to ensure that any network meta‐analysis of interventions will be meaningful, relevant and feasible." In the event, no network meta‐analysis was possible, and the treatments were grouped after data extraction was completed. This process could not be performed blind to the results, but the subgrouping decisions were based on clinical considerations and the similarity, or otherwise, of the treatments compared, and we do not believe this is an important source of bias.

In the protocol we stated: "For dichotomous variables, we will express the results as odds ratios (OR) with 95% confidence intervals (CI)." The risk ratio (RR) is a more intuitive statistic for reporting the results of RCTs because the meaning of an OR depends on the baseline risk and so is difficult to interpret even for readers who understand what an OR is. We have used RR instead of OR.

In the protocol we stated "If available, we plan to extract and report Hazard Ratios (HRs) for time‐to‐event‐data (mortality or time in hospital). If HRs are not available, we will make every effort to estimate as accurately as possible the HR using the available data and a purpose‐built method based on the Parmar and Tierney approach (Parmar 1998*; *Tierney 2007). If sufficient studies provide HRs, we plan to use these in favour of other reported treatment effects in the meta‐analysis, otherwise we will perform a separate meta‐analysis for all types of reported treatment effects, for example ORs. If the events are rare and the follow‐up times are similar we will consider the perceived similarity of ORs, risk ratios (RRs) and HRs." Only one trial (POISE‐3 2022) reported any HRs, and we did not attempt to estimate any others because it adds little value compared to RR when there is short‐term follow‐up and little loss to follow‐up. Time‐to‐event analysis for in‐hospital follow‐up also has a number of additional complications which are often not recognised by trial authors (McCaw 2022).

We did not contact authors for missing data because most trials with missing data had primarily intraoperative follow‐up and did not measure the outcomes we are interested in.

We changed the inclusion criteria for type of study to make it clearer that elective endovascular procedures were not included.
